# Hydrogen Atom Transfer Driven Enantioselective Minisci Reaction of Alcohols

**DOI:** 10.1002/anie.202200266

**Published:** 2022-04-27

**Authors:** Avene C. Colgan, Rupert S. J. Proctor, David C. Gibson, Padon Chuentragool, Antti S. K. Lahdenperä, Kristaps Ermanis, Robert J. Phipps

**Affiliations:** ^1^ Yusuf Hamied Department of Chemistry University of Cambridge Lensfield Road Cambridge CB2 1EW UK; ^2^ School of Chemistry University of Nottingham University Park Nottingham NG7 2RD UK

**Keywords:** Asymmetric Catalysis, Chiral Phosphoric Acids, Heterocycles, Minisci Reaction, Organocatalysis

## Abstract

Catalytic enantioselective Minisci reactions have recently been developed but all instances so far utilize α‐amino radical coupling partners. We report a substantial evolution of the enantioselective Minisci reaction that enables α‐hydroxy radicals to be used, providing valuable enantioenriched secondary alcohol products. This is achieved through the direct oxidative coupling of two C−H bonds on simple alcohol and pyridine partners through a hydrogen atom transfer (HAT)‐driven approach: a challenging process to achieve due to the numerous side reactions that can occur. Our approach is highly regioselective as well as highly enantioselective. Dicumyl peroxide, upon irradiation with 390 nm light, serves as both HAT reagent and oxidant whilst selectivity is controlled by use of a chiral phosphoric acid catalyst. Computational and experimental evidence provide mechanistic insight as to the origin of selectivity, revealing a stereodetermining deprotonation step distinct from the analogous reaction of amide‐containing substrates.

## Graphical abstract

An enantioselective Minisci reaction between pyridines and prochiral α‐hydroxy radicals has been developed. Hydrogen atom transfer is used to generate the latter from simple alcohols and the reaction formally constitutes the couple of two C−H bonds to form a new C−C bond in a process that is highly enantioselective and regioselective. Computational studies provide insight into the origins of selectivity.

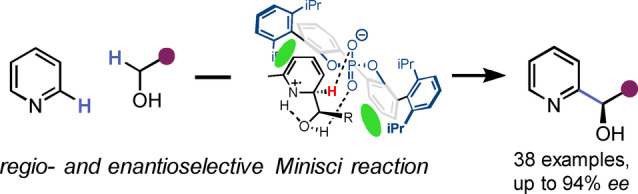

## Introduction

**Figure 1:**
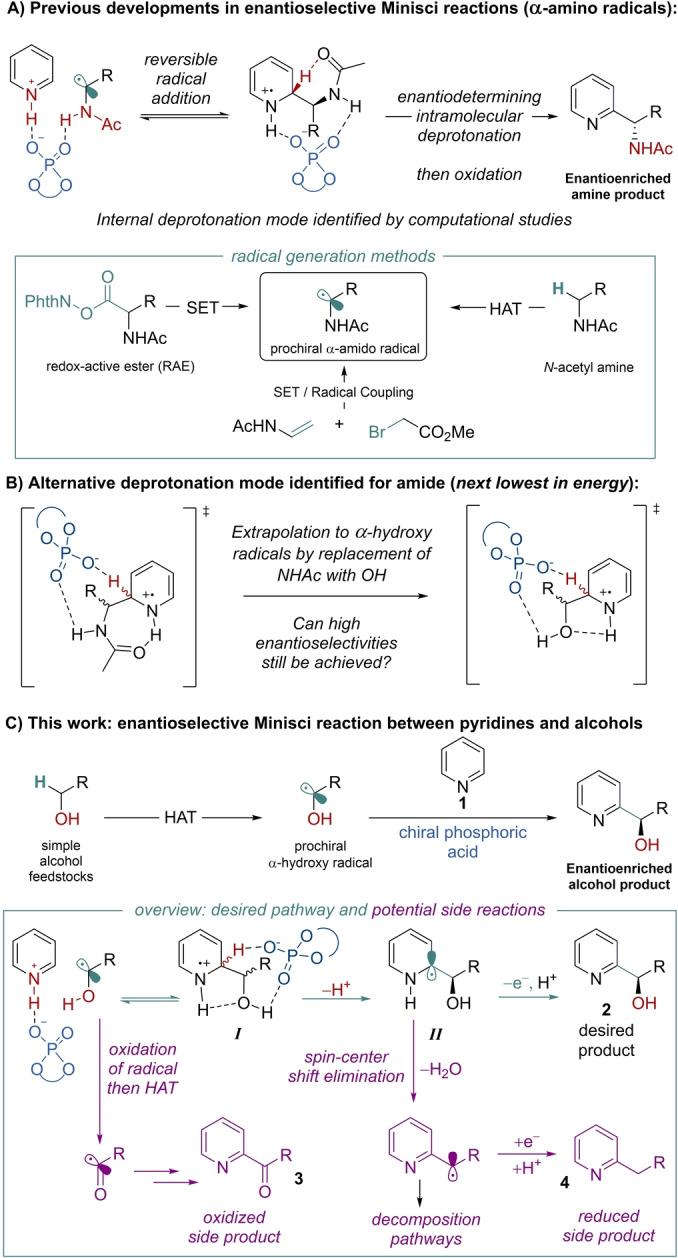
Previous work and the present study.

The development of new methods for the efficient and enantioselective synthesis of chiral compounds containing basic heteroarenes is extremely important due to their prevalence in molecules of medicinal importance.^[1]^ In Minisci‐type reactions, an activated basic heteroarene undergoes addition of a carbon‐centered radical followed by oxidative rearomatization to afford functionalized heteroarene products, constituting a powerful method to significantly increase molecular complexity in a single step.^[2]^ If prochiral radicals are used then a new stereocenter is consequently formed. We recently developed an enantioselective Minisci‐type reaction whereby prochiral *N*‐acetyl α‐amino radicals were oxidatively coupled with basic heteroarenes with high levels of enantiocontrol as well as regiocontrol.^[3]^ This was achieved by the use of a chiral phosphoric acid (CPA) catalyst^[4]^ in combination with photoredox catalysis (Figure 1A).^[5]^ Whilst our original protocol used redox‐active esters (RAEs)^[6]^ as radical precursors and was explored on pyridines and quinolines, further developments of the enantioselective Minisci reaction have been subsequently reported by ourselves and others. These include expansion of the heterocyclic component to isoquinolines^[7]^ and also to diazines,^[8]^ the latter in combination with the development of a predictive model through multivariate statistical analysis. Methods for fragmenting the RAEs that do not require precious metal photocatalysts have also been demonstrated.^[9]^ In terms of the radical generation method, a three component coupling has been developed^[10]^ and we have also recently realized a HAT‐driven protocol that couples simple *N*‐acetylated amines with heteroarenes (Figure 1A, inset box).^[11]^ The latter is particularly attractive as it constitutes an example of a cross‐dehydrogenative coupling (CDC), permitting the formal oxidative coupling of C−H bonds on each reaction partner and so allowing use of the simplest possible starting materials, while still exerting high levels of enantio‐ and regio‐ control.^[12]^

It is conspicuous that all examples of enantioselective Minisci reactions to date feature carbonyl‐protected amines on the prochiral radical. Indeed, early optimization in our system showed that even changing the acetate to a carbamate or trifluoroacetate was highly detrimental to both yield and enantioselectivity.^[3]^ The crucial role played by the acetamide was subsequently elucidated in a detailed computational and experimental investigation into the origin of selectivity in the reaction.^[13]^ Experimental evidence suggested that the deprotonation of the intermediate radical cation was the selectivity‐determining step, leading to high enantioselectivity, as well as excellent regioselectivity for the C2 position of the heteroarene. Whilst initial computational explorations assumed that the chiral phosphate anion itself was performing this deprotonation, the lowest energy pathway by a considerable degree was ultimately obtained by invoking an unexpected mode of “internal” deprotonation enacted by the amide carbonyl, with the assistance of the associated chiral phosphate (Figure 1A). This stereoselectivity model predicted enantioselectivity outcomes for a range of heterocycle substrates very satisfactorily and also accounted for the importance of the acetamide group. In the selectivity‐determining transition state, a network of non‐covalent interactions provides a high degree of organization within the chiral pocket of the bulky phosphoric acid catalyst, leading to very high enantiomeric excesses.^[14]^ This analysis gave us cause for concern that the chemistry may be intrinsically limited to α‐acetamido radicals, with this unique deprotonation mode limiting broader application to other types of valuable radical nucleophiles.

Despite the “internal” deprotonation by the amide being unambiguously favoured, the computational studies suggested that the mode of deprotonation next lowest in energy featured the originally anticipated direct deprotonation by the chiral phosphate, which concurrently forms a hydrogen bond with the amide NH (Figure 1B, left). We hypothesized that an analogous transition state might be viable in which the NHAc is replaced with OH and in which a stabilizing hydrogen bond between the alcohol oxygen and the NH of the radical cation could still be plausible, leading to a high degree of organization (Figure 1B, right). If successful, this would enable a significant expansion of catalytic enantioselective Minisci chemistry. Although asymmetric reduction is established in allowing access to 2‐pyridyl secondary alcohols, this first requires synthesis of the 2‐acyl alcohol in a prior step.^[15]^ Using a Minisci approach increases the expediency with which these motifs, important in both medicinal chemistry and in ligand designs, can be accessed. To generate the requisite α‐hydroxy radicals, we decided to pursue a HAT‐driven approach since this would allow simple primary alcohols to be used and would constitute a CDC‐type process that would be the most synthetically attractive option, removing any requirement for pre‐functionalization.^[12]^ Whereas reports of Minisci‐type reactions involving HAT from ethers are ubiquitous and number in the hundreds, those featuring examples of HAT from alcohols are somewhat fewer.^[16]^ Whilst still well precedented, it is evident from the relatively small number of examples that Minisci‐type hydroxyalkylations pose particular challenges. These can be appreciated by considering the established side reactions that may compete with the desired reaction pathway (Figure 1C, inset box, green arrows vs purple arrows). Firstly, the prochiral α‐hydroxy radical may be susceptible to oxidation to the corresponding aldehyde, which itself can undergo HAT, diminishing the concentration of desired α‐hydroxy radicals and giving an acyl radical which may undergo Minisci‐type addition to ultimately form side product **3**. Second, if the mechanism would proceed analogously to the amide version, after reversible radical addition the resultant radical cation ***I*** would undergo enantiodetermining deprotonation giving neutral radical ***II***. Single electron oxidation of this would give desired product **2**. However, this may be in competition with a spin‐center shift (SCS) process, where the α‐hydroxy group is eliminated as water, giving a benzylic radical which could lead to reduced side‐product **4** in addition to miscellaneous decomposition pathways.^[17]^ Identification of a suitable oxidant/HAT system which would lead to the desired product whilst at the same time exhibiting compatibility with the chiral phosphoric acid catalyst system would be key. We herein describe the realization of this approach through the development of a highly enantioselective Minisci‐type hydroxyalkylation reaction (Figure 1C). With control of both enantioselectivity and regioselectivity in the addition, this reaction, formally a CDC of simple alcohol and pyridine precursors, establishes a direct approach to enantioenriched hydroxyalkylated pyridines. More importantly it demonstrates that that the CPA‐catalysed Minisci reaction is not limited to α‐amino radicals.

## Results and Discussion

Our investigations began with examination of the reaction between 2‐*n*‐pentylpyridine (**1** **a**) and hydrocinnamyl alcohol (**5**). Alongside the challenge of controlling enantioselectivity, each reaction partner poses challenges relating to positional selectivity, specifically site‐selectivity in the HAT event (α‐hydroxy vs benzylic) and regioselectivity in the pyridine functionalization (C2 vs C4). Initially, we tested the conditions that had been successful in our HAT‐driven Minisci reaction of amides, which used diacetyl ((CH_3_CO)_2_) as both oxidant and HAT reagent under irradiation with blue LEDs (Kessil Tuna Blue), together with (*R*)‐**TRIP** as the CPA (Table 1, entry 1).[^11^, ^18^] We were very encouraged to observe formation of the desired C‐6 functionalized product **2** (23 % NMR yield) in 76 % ee. Crucially, the majority of the remaining mass balance could be accounted for as unreacted **1**, suggesting that the potential side reactions outlined in Fig. 1c were not occurring under these particular conditions. We next replaced diacetyl with dicumyl peroxide (DCP) in combination with photocatalyst **Ir‐I** which gave similar results in terms of both yield and ee (entry 2). Use of thioxanthone (**THX**) in place of **Ir‐I**, gave a slightly lower yield (19 %) but retained ee (81 %, entry 3). The effectiveness of **THX** suggested that peroxide cleavage may be occurring through a photosensitization mechanism^[19]^ and accordingly, omission of **THX** resulted only in trace amounts of desired product being formed (entry 4). In an effort to maximize excitation of **THX** (*λ*_max_=377 nm (EtOAc)^[20]^) we switched to irradiation using a 390 nm Kessil lamp, which improved the yield significantly (53 %) whilst retaining comparable enantiomeric excess (74 %, entry 5). Intriguingly, omission of **THX** whilst irradiating with the 390 nm light source furnished the desired product in effectively the same yield (50 %) and slightly improved enantioselectivity (77 %), demonstrating that extraneous photosensitizer is not necessary when using a 390 nm light source (entry 6). To probe this further, irradiation at 390 nm of a solution of dicumyl peroxide in EtOAc showed significant consumption of the peroxide after 1 h (41 %). In contrast, irradiation with Kessil Tuna Blue displayed only 4 % consumption of DCP and under no irradiation, no consumption of dicumyl peroxide was observed (see Supporting Information for full details). On this basis, it appears that direct cleavage of the peroxide is occurring upon irradiation with the lower wavelength lamp.^[21]^ In an effort to improve enantioselectivity, other CPAs were evaluated (entries 7 and 8) with (*R*)‐**DIP**, a variant of **TRIP** where the furthest isopropyl groups have been removed, providing the best outcome (83 % ee). Lowering the reaction temperature was found to improve both yield (62 %) and ee (87 %) (entry 9). Finally, extending the reaction time to 24 h furnished the product in 70 % NMR yield (62 % isolated) and the enantiomeric excess was maintained (entry 10). Carrying out the optimal reaction in the dark resulted in no product formation, highlighting the crucial requirement of light for cleavage of the DCP (entry 11). We found that reduction of the alcohol equivalents below 20 at standard concentration led to unacceptable loss of yield while systematic lowering of alcohol equivalents combined with increasing reaction concentration resulted in lower enantioselectivity. Other solvents and peroxides examined resulted in either reduced yield and/or reduced enantioselectivity (see Supporting Information for full details).

**Table 1:** Optimization of the enantioselective Minisci reaction between alcohols and pyridines.

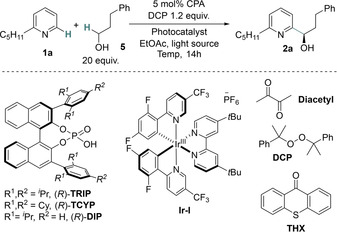						
						
Entry	Photocatalyst	CPA	*T*	Light Source	Yield [%]^[a]^	ee [%]
1^[b]^	–	(*R*)‐TRIP	rt	Tuna blue	23	76
2^[c]^	Ir‐1	(*R*)‐TRIP	rt	Tuna blue	30	81
3^[d]^	THX	(*R*)‐TRIP	rt	Tuna blue	19	81
4	–	(*R*)‐TRIP	rt	Tuna blue	–	–
5^[d]^	THX	(*R*)‐TRIP	rt	Kessil 390	53	74
6	–	(*R*)‐TRIP	rt	Kessil 390	50	77
7	–	(*R*)‐TCYP	rt	Kessil 390	46	81
8	–	(*R*)‐DIP	rt	Kessil 390	40	83
9	–	(*R*)‐DIP	5 °C	Kessil 390	62	87
10^[e]^	–	(*R*)‐DIP	5 °C	Kessil 390	70 (62)	86
11^[f]^	–	(*R*)‐DIP	5 °C	Kessil 390	0	–

[a] Reactions carried out on a 0.05 mmol scale. Yield determined by ^1^H‐NMR analysis with reference to an internal standard; isolated yield in parentheses. [b] Diacetyl (10 equiv) used as oxidant in place of DCP. [c] Ir‐1 2 mol % was used. [d] THX 10 mol % was used. [e] Reaction time of 24 h. [f] Reaction run in the dark.

With optimal conditions in hand, we examined the substrate scope with respect to pyridines with reactions carried out on a 0.2 mmol scale with respect to heteroarene (Table 2). Although some of the yields are moderate this was typically a result of incomplete conversion. Whilst conversion could be increased by adding further peroxide, either from the outset or after a specific time, this did not typically increase product yields, rather resulting in formation of multiple by‐products and complex reaction mixtures. In some cases, purification also proved challenging due to the excess alcohol, and led to a reduction in the isolated yield. Nevertheless, we believe that the moderate yields can be justified by the complexity generated in a single step from simple starting materials and the fact that high levels of control are being exerted over enantioselectivity as well as multiple aspects of site‐selectivity using our developed system. Alongside 2‐*n*‐pentylpyridine (**2** **a**) a number of C‐2 alkylated pyridines were tolerated, including simple 2‐methyl pyridine (**2** **b**), as well as those bearing branched alkyl substituents at C‐2 in the form of isopropyl (**2** **c**) and cyclohexyl (**2** **d**). We were particularly encouraged that deleterious HAT seemed not to occur on the cyclohexyl ring, a feature that had not been tolerated in our HAT‐driven amide Minisci reaction. This was also the case for **2** **e**, featuring a methylene spacer between the cyclohexane ring and the pyridine. Furthermore, alkyl substituents bearing protected amino (**2** **f**) and hydroxy (**2** **g**) groups as well as phenyl (**2** **h**) were effective and gave similar yields to pyridine bearing unfunctionalized alkyl chains. 2,3‐Dimethylpyridine was an effective substrate (**2** **i**) and fused bicyclic substrates with varying ring sizes also performed well (**2** **j**, **2** **k**). There was no problem with the Minisci reaction occurring adjacent to an existing methyl group and indeed in this situation, slightly increased levels of enantioselectivity were obtained (**2** **m**, **2** **n**). In the case of 3‐methyl‐5‐phenyl pyridine very high regioselectivity for attack at the position adjacent to the methyl was observed, as opposed to adjacent to the phenyl (**2** **o**). In the case of 3‐picoline however, a mixture was obtained with an interesting preference for reaction adjacent to the methyl (**2** **p**), contrary to steric considerations. The major isomer also gave superior enantioselectivity and the regioisomers could be successfully separated by column chromatography, albeit in reduced yield. To probe this effect further we also investigated *N*‐Boc‐3‐aminopyridine pyridine which also gave a mixture of regioisomers at the two C2 positions, but in very low yield (see Supporting Information). A Boc‐protected amine could however be incorporated effectively into a related substrate if it was separated from the ring by a methylene unit (**2** **q**). 2‐Methyl‐5‐ethylpyridine is a bulk chemical used in the synthesis of nicotinic acid and we found that Minisci reaction on this occurred adjacent to the ethyl group with high enantioselectivity (**2** **r**). We found that simple unsubstituted pyridine underwent reaction but with reduced ee (**2** **s**, 73 %), although interestingly the minor by‐product arising from diaddition was found to have 95 % ee. Efforts to push the conversion of this reaction to favour the diaddition product unfortunately resulted in a complex reaction mixture. The inclusion of a *tert‐*butyl substituent at the 4‐position was found to improve the enantioselectivity significantly (**2** **t**). We also found that pyridines bearing electron‐withdrawing groups did not give appreciable product yield which is in contrast to the amide Minisci in which an electron‐withdrawing group was required on the pyridine in order to obtain reactivity, illustrating the subtle differences between the characteristics of the two reactions, the origins of which remain unclear at present.

**Table 2:**
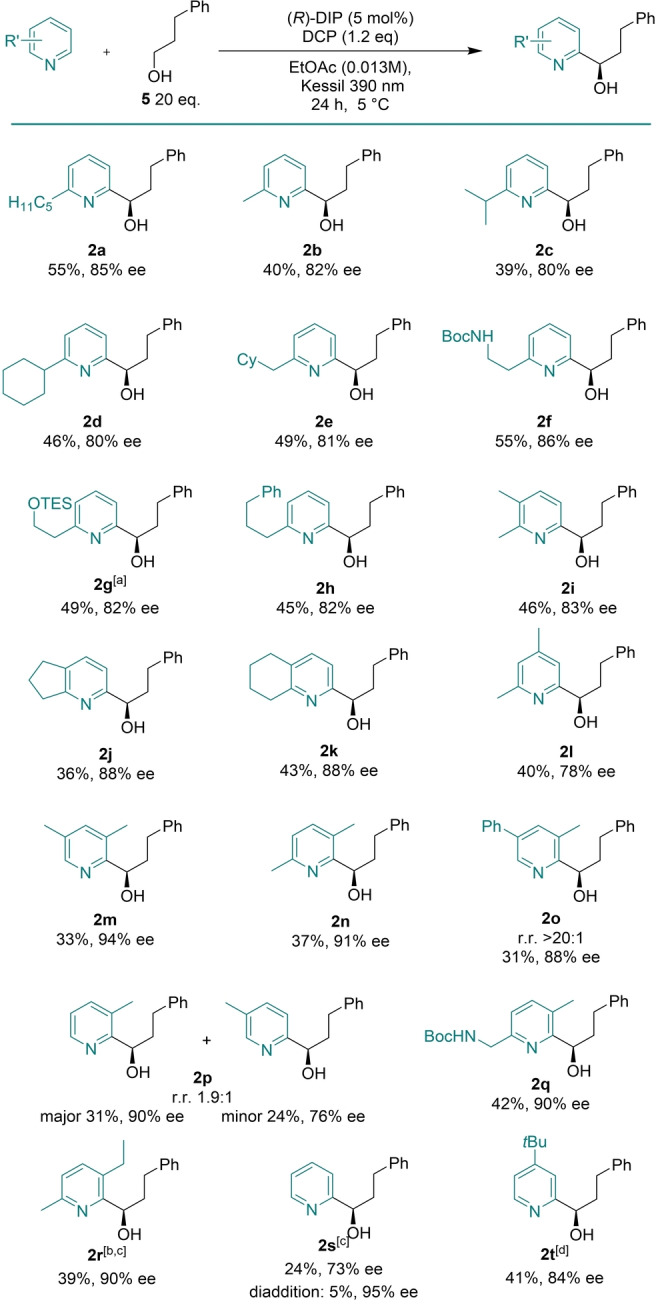
Pyridine scope exploration.

Yields are those of isolated product. [a] Isolated following TES deprotection. [b] DCP 5 equiv was used, 3 h reaction time [c] Isolated following acetyl protection of crude material. [d] Alcohol was acetylated after isolation to facilitate ee determination.

Finally, lepidine was found to give extremely low product yield, alongside low yields of products obtained from competing SCS side reaction pathways outlined in Figure 1C. The mass balance of the reaction was low suggesting that for lepidine other degradation pathways also likely occurred (see Supporting Information for details). Several other heterocycle classes were also tested under the conditions optimized for pyridines but these gave no discernible product formation (see Supporting Information for details). Subjection of hydroxyalkylated pyridines **2** **b** and **2** **k** to Mosher ester analysis enabled the absolute stereochemistry of the products to be assigned as *R* (see Supporting Information for details).^[22]^ Intriguingly, the absolute stereochemistry of the obtained major enantiomer in this work was opposite to that obtained in the analogous amide Minisci reaction, despite use of the same enantiomer of CPA catalyst.

We next evaluated variation of the alcohol component (Table 3). Variously substituted phenylpropanols were effective with incorporation of *ortho*‐bromo (**6** **a**), *ortho‐*methoxy (**6** **b**), *para‐*bromo (**6** **c**) and *ortho*‐fluoro (**6** **d**) substituents well tolerated. Phenylethanols performed well in the reaction and a variety of substituted examples were demonstrated, including *ortho*‐fluoro (**6** **e**), *para*‐bromo (**6** **f**), *ortho*‐methyl (**6** **g**) and *para*‐methoxy (**6** **h**). The *para*‐methoxy example was a welcome addition as the equivalent *N*‐acetyl phenethylamine bearing a *para*‐methoxy substituent performed poorly in the corresponding HAT‐driven amide Minisci reaction, again highlighting subtle differences between the two reactions. The chain length between the alcohol and phenyl ring could be extended with phenylbutanol (**6** **i**). We were pleased to see that the scope of the alcohol component could include simple aliphatic alcohol feedstocks such as *n*‐butanol (**6** **j**). The use of branched alcohols posed no problem and good results were obtained with an alcohol possessing a distal cyclohexane ring **(6** **k)**. In these cases, we were particularly happy that useful yields of the desired product could be achieved through HAT, even in the presence of other potentially abstractable C−H bonds on the alcohol radical precursors. A cyclobutanol‐containing alcohol gave excellent results (**6** **l**), as did alkyne‐containing alcohol radical precursors **6** **m** and **6** **n**. A simple aliphatic alcohol bearing an ester group was tolerated (**6** **o**) although a TBS‐protected alcohol as well as a Boc‐protected amine at the same position saw the ee decrease to 74 % in both cases (**6** **p** and **6** **q**). We also evaluated a secondary alcohol, but this gave no desired Minisci product. For details of this and other alcohols that were tested but gave poor results, see the Supporting Information.

**Table 3:**
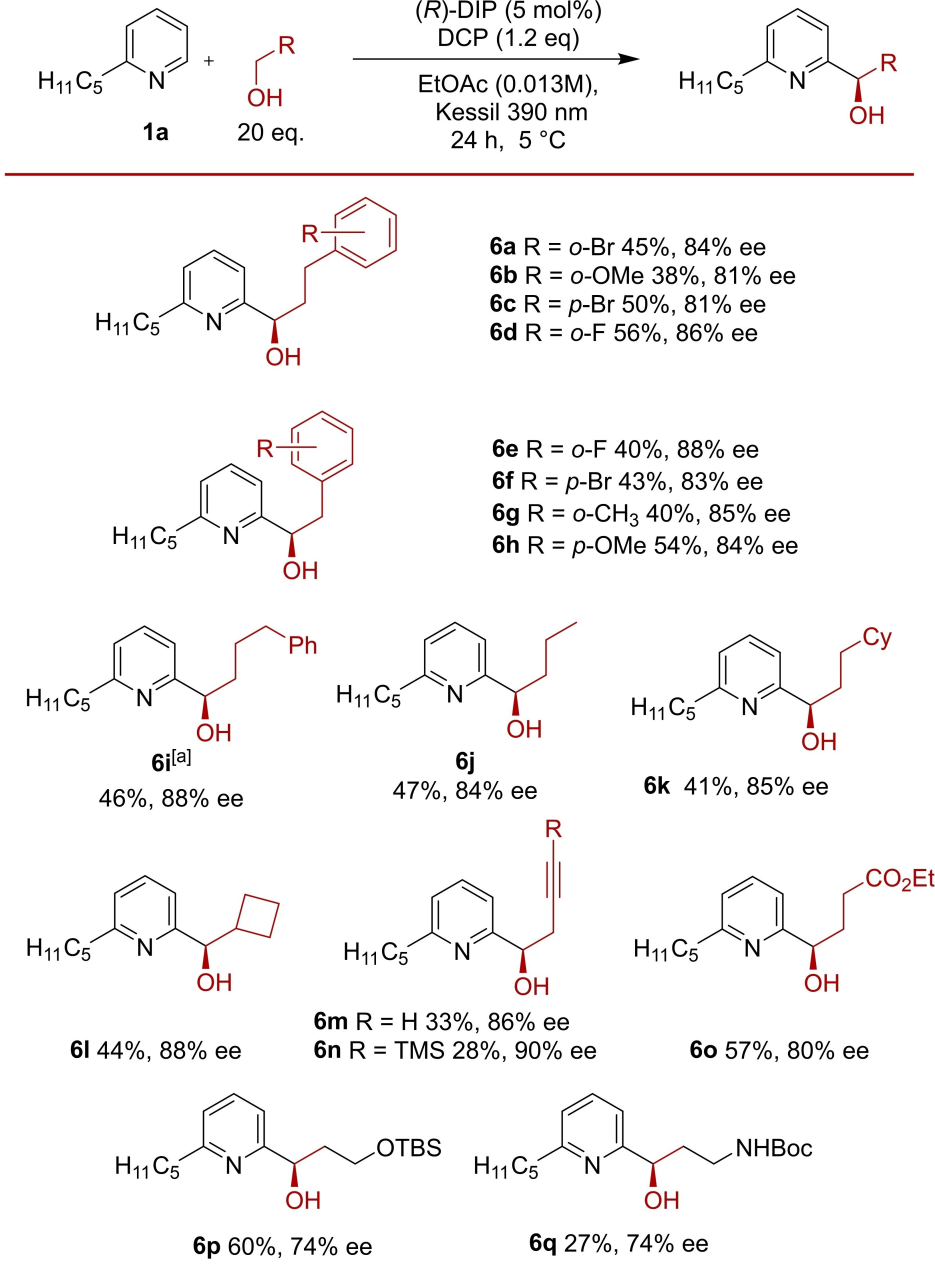
Alcohol scope exploration.

Yields are those of isolated product. [a] Alcohol was acetylated after isolation to facilitate ee determination.

**Scheme 1:**
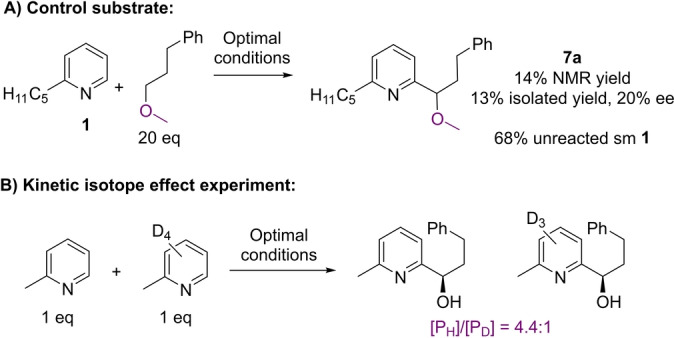
Mechanistic probe experiments.

To test the importance of the free hydroxyl group we next examined a control substrate in which the alcohol was methylated (Scheme 1A). This underwent inefficient reaction in which the product **7** **a** was isolated in very low yield and with very low enantioselectivity, highlighting the importance of the free alcohol for effective reaction in both senses. Finally, the primary kinetic isotope effect (KIE) in an intermolecular competition experiment was determined to be 4.4 (Scheme 1B). This figure, which is in line with previous related experiments,[^13^, ^23^] strongly suggests that in our case the radical addition is reversible, and that the deprotonation of the intermediate radical cation is selectivity‐determining.

**Figure 2:**
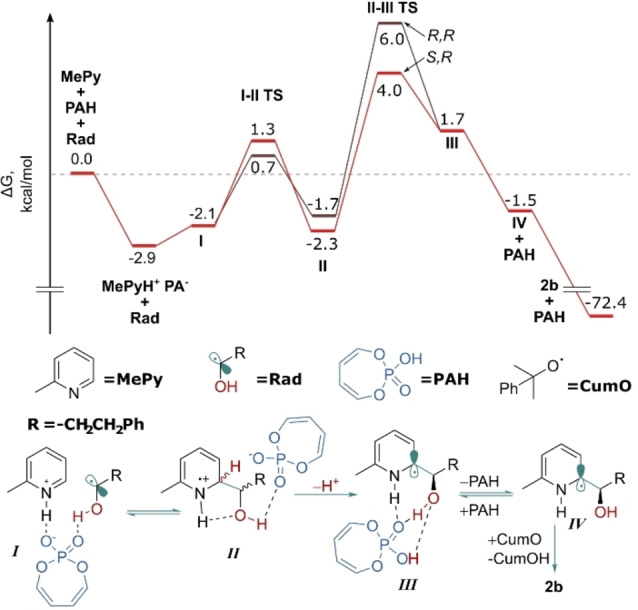
Computational modelling results of the CPA‐catalyzed reaction steps, using a model phosphoric acid. Relative free energies shown in kcal mol^−1^, calculated at B2PLYPD3/def2‐TZVP^[32]^/SMD(ethylacetate)//M06‐2X/6‐31G**/SMD(ethylacetate).

At this stage we sought to investigate the origin of selectivity computationally, to compare and contrast this system with the previously developed enantioselective amide Minisci reaction. To computationally validate the experimental indication that deprotonation is likely enantiodetermining, we first investigated the CPA‐catalyzed steps on a model system using a simplified catalyst. Geometries were optimized at M06‐2X^[27]^/6‐31G**^[25]^/SMD(ethylacetate)^[26]^ level. Single‐point energies were then calculated using either M06‐2X or double‐hybrid B2PLYPD3^[28]^ functionals. In our previous studies of the amide Minisci reaction we encountered a systematic error when using standard DFT methods, which problematically overestimated the stability of delocalized radicals when compared with localized ones.[^13^, ^29^] In the current model system we observed the same effect—M06‐2X results erroneously suggested that radical addition might be the selectivity‐determining step (see Supporting Information, Figure S9 for details). However, energies obtained using the significantly more expensive double‐hybrid B2PLYPD3 functional clearly showed that the deprotonation (***II**–**III***) is the selectivity‐determining step, with a significantly higher barrier than radical addition (***I**–**II***) (Figure 2). This was now in good agreement with the experimentally determined selectivity‐determining step, as suggested by the experimental KIE results. In addition, the DFT‐predicted KIE value^[30]^ for the deprotonation step (7.3) was in good qualitative agreement with the experimental value (4.4), while a KIE of only 1.05 was predicted for the addition step.^[31]^ All this gave us the confidence to proceed with more extensive studies of the deprotonation step as the selectivity‐determining step, using the full catalyst system.

**Figure 3:**
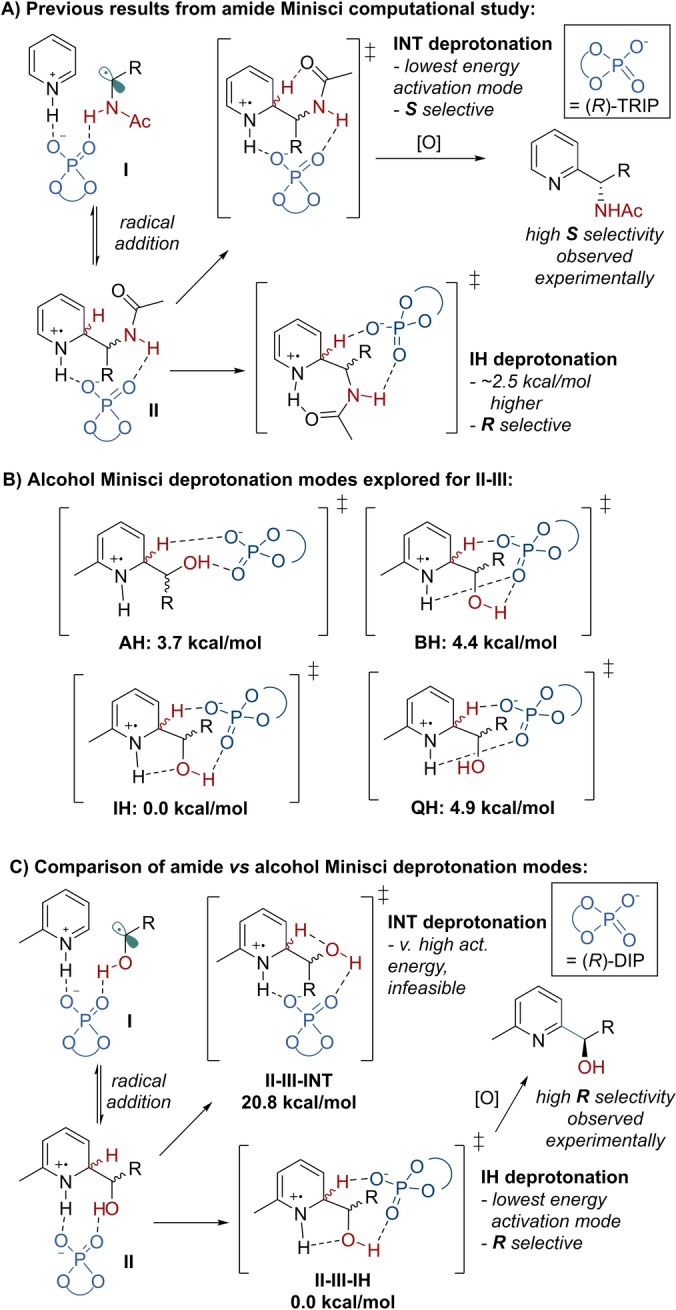
A) Previous results from the amide Minisci computational study. B) Summary of different deprotonation modes explored for the alcohol Minsici reaction, using chiral DIP catalyst. C) Comparison of alcohol vs amide deprotonation modes and stereochemical outcome. Relative free energies shown in kcal mol^−1^, calculated at M06‐2X/def2‐TZVP/SMD(ethylacetate)//B3LYP/6‐31G**.

**Figure 4:**
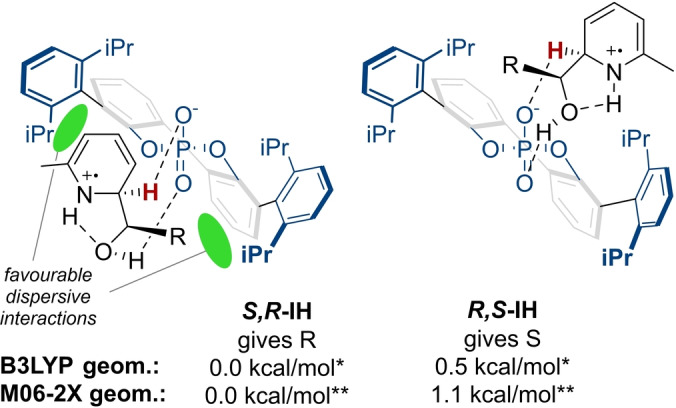
Lowest energy deprotonation TSs for the experimentally major and minor products. * Relative free energies shown calculated at M06‐2X/def2‐TZVP/SMD(ethylacetate)//B3LYP/6‐31G**/SMD(ethylacetate). ** Relative free energies shown calculated at M06‐2X/def2‐TZVP/SMD(ethylacetate)//M06‐2X/6‐31G**/SMD(ethylacetate).

We next interrogated the deprotonation step to identify the lowest‐energy activation mode and probe the origin of enantioinduction (Figure 3). In our previous computational evaluation of the amide Minisci, the second lowest set of transition states, corresponding to activation mode **IH**, featured the phosphate, as opposed to the amide, enacting the crucial deprotonation (Figure 3A). Importantly, this deprotonation mode predicted formation of the *R* enantiomer, which did not match the *S* product that was observed in that reaction, but which does match the analogous major enantiomer obtained in this present study using alcohols. To understand the deprotonation pathways possible in the alcohol Minisci reaction, conformational searches for the full DIP‐catalyzed deprotonation transition states were conducted. Thereafter, 22 carefully selected TS‐like structures across the four possible diastereomers of the radical addition intermediate **II** were optimized using B3LYP^[24]^ functional, and single‐point energies were calculated using M06‐2X. Calculating the single‐point energies at double‐hybrid level using B2PLYPD3 was not possible for the full system due to the immense computational cost at this size. In our previous computational studies of amide Minisci reaction we found M06‐2X single‐point energies in excellent agreement with experimental observations, when focusing just on the selectivity‐determining step.^[13]^ In the current study these computations identified four possible activation modes, **IH**, **AH**, **BH** and **QH** (Figure 3B), all of which feature the phosphate acting as base. The lowest energy of these was **IH**, which features two hydrogen bonds: one between the phosphoryl oxygen and the alcohol and another between the hydroxy oxygen and the pyridinium NH. While the latter is relatively long at 2.3 Å, it is energetically important: the corresponding activation mode without this intramolecular hydrogen bond (**AH**) is 3.7 kcal mol^−1^ higher in energy. The **BH** mode, in which the phosphoryl oxygen hydrogen bonds to both the pyridinium and the hydroxy group concurrently was higher still. Finally, the **QH** activation mode which features only a single hydrogen bond, between the phosphate and the pyridinium, was the highest of the four. All of these activation modes were also analogously identified in the amide Minisci computational study and exhibited the same energetic ordering. Separately, a search was conducted for an **INT**‐like activation mode, analogous to the lowest energy amide deprotonation transition state, where the alcohol would act as an internal base. Whilst the amide **INT** mode featured a six‐membered cyclic TS, the alcohol analogue would necessitate a four‐membered cyclic TS, suggesting significantly higher strain. This was indeed the case, as identified **INT** TS was 20.8 kcal mol^−1^ higher than the lowest **IH** deprotonation TS (Figure 3C). The lowest energy **IH** activation mode transition state energies predicted the alcohol reaction to be *R* selective. Correspondingly, the lowest energy *R*‐producing TS ***S,R*** **‐IH** was found to have an activation free energy 0.5 kcal mol^−1^ lower than the lowest energy *S*‐producing ***R***, ***S*** **‐IH**, which is qualitatively consistent with the observed experimental enantioselectivities. Inspection of the lowest **IH** deprotonation transition states reveals that the substrate conformations are very similar and effectively enantiomers of each other (Figure 4, B3LYP).

**Figure 5:**
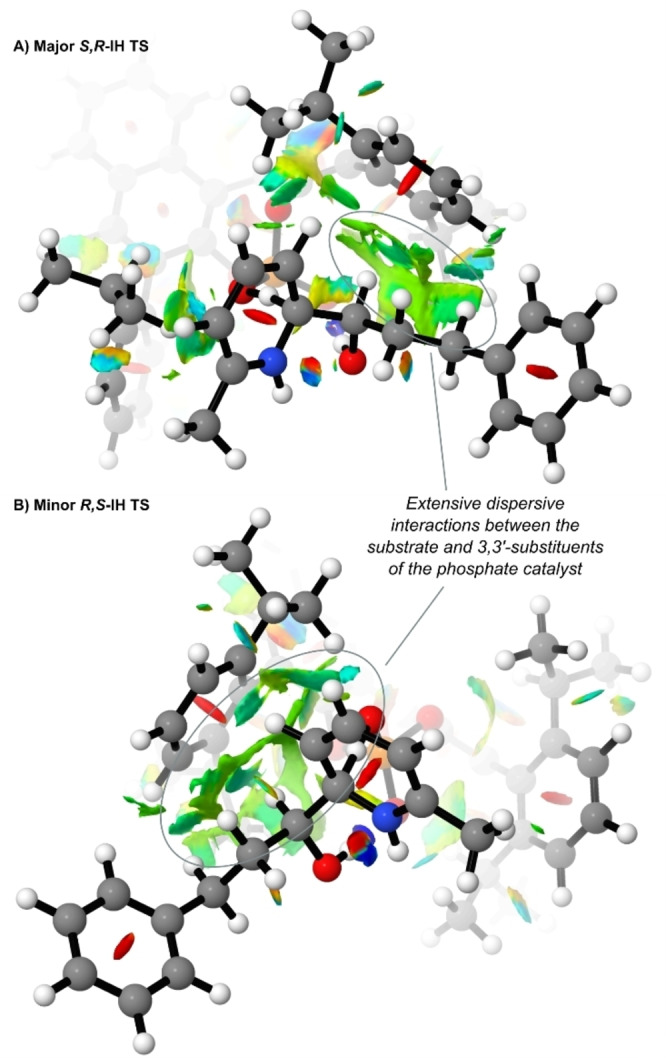
NCI plots of the lowest energy deprotonation TSs for the experimentally major (a) and minor (b) products. Calculated at M06‐2X/def2‐TZVP/SMD(ethylacetate)//M06‐2X/6‐31G**/SMD(ethylacetate).

To probe the possible non‐covalent interactions responsible for enantioinduction, NCI plots were calculated and visualized,^[33]^ revealing several regions of favourable dispersive interactions in the major ***S***, ***R*** **‐IH** TS between the substrate and the 3,3′‐substituents of the catalyst (see Supporting Information—Figure S5).^[34]^ These interactions were largely absent from the minor ***R***, ***S*** **‐IH** transition state, suggesting a plausible basis for rationalizing enantioselectivity. The apparent importance of dispersive interactions in the lowest energy TSs prompted us to reoptimize the B3LYP ***S***, ***R*** **‐IH** and ***R***, ***S*** **‐IH** geometries using M06‐2X/6‐31G**/SMD(ethylacetate), as this would better account for the dispersion interactions than simply calculating single‐point energies with M06‐2X. The minor enantiomeric ***R,S*** **‐IH** TS was now calculated to be 1.1 kcal mol^−1^ higher than ***S***, ***R*** **‐IH**, as opposed to the 0.5 kcal mol^−1^ previously predicted (Figure 4, M06‐2X). This now corresponds to an ee of 77 %, which excellently matches the experimentally observed selectivity (82 % ee). To try to understand the important differences between the competing transition states, a distortion‐interaction study was undertaken (see Table S8 in the Supporting Information).^[35]^ Following the standard approach, single‐point energies (M06‐2X/def2‐TZVP/SMD(ethylacetate)) were calculated for both the chiral phosphate anion and the cationic substrate transition state 3D geometries separately. Then minimum geometries for both components were optimized separately, to determine the “strain” in the TS geometries of each component. Whilst DIP phosphate “strain” was very similar for both TSs, the strain of the cationic partner was calculated to be 16.7 kcal mol^−1^ for ***S***, ***R*** **‐IH** and 18.8 kcal mol^−1^ for ***R***, ***S*** **‐IH**, the significantly higher value for the minor transition state indicating a strong contribution to the observed enantioselectivity. Phosphate‐substrate interactions were found to be −42.5 kcal mol^−1^ for the major ***S***, ***R*** **‐IH** and −43.1 kcal mol^−1^ for the minor ***R***, ***S*** **‐IH**, thus being slightly more favourable in the minor ***R***, ***S*** **‐IH** TS by about 0.6 kcal mol^−1^. Ionic interactions are of course by far the most significant factor in these but should be very similar for both TSs. Overall, this analysis suggests that the major enantiodetermining factor is the substrate activation strain. The favoured ***S***, ***R*** substrate diastereomer fits better in the catalyst pocket, and therefore experiences less strain, thus leading to the high levels of enantioselectivity observed. The extensive dispersive interactions can be clearly seen in the NCI plots of the two final TSs (Figure 5). This outcome echoes other recent studies which emphasize the crucial role that dispersion interactions can play in providing enantioselectivity in other systems.^[36]^

## Conclusion

We have developed an enantioselective Minisci reaction that couples feedstock alcohols with pyridines in what is formally the cross‐dehydrogenative coupling of two C−H bonds, with excellent control over both enantioselectivity and regioselectivity. This new development of the asymmetric Minisci reaction is of particular note because all previous examples have required a protected amine functional group to be present on the prochiral radical, limiting the protocol to the synthesis of chiral amines. By demonstrating that enantioenriched secondary alcohols can now be accessed, the breadth of this approach is significantly widened. Furthermore, computational studies allow us to understand the way in which the phosphoric acid catalyst operates, in comparison with the amide variant, and to rationalize the observed enantioinduction. This assists in building a coherent picture of how the CPA‐catalyzed Minisci reaction functions and will inform future developments to expand the scope of this approach to valuable, enantioenriched heterocyclic compounds.

## Conflict of interest

The authors declare no conflict of interest.

1

## Supporting information

As a service to our authors and readers, this journal provides supporting information supplied by the authors. Such materials are peer reviewed and may be re‐organized for online delivery, but are not copy‐edited or typeset. Technical support issues arising from supporting information (other than missing files) should be addressed to the authors.

Supporting Information

## Data Availability

The data that support the findings of this study are available in the Supporting Information of this article.
