# Supplementary material for: Hydrogen Atom Transfer Driven Enantioselective Minisci Reaction of Alcohols
Source: Angew Chem Int Ed Engl. 2022 Apr 27;61(25):e202200266. doi: 10.1002/anie.202200266 (PMC9321721; doi:10.1002/anie.202200266)

Angewandte Chemie
Eine Zeitschrift der Gesellschaft Deutscher Chemiker

Supporting Information

Hydrogen Atom Transfer Driven Enantioselective Minisci Reaction of Alcohols

A. C. Colgan, R. S. J. Proctor, D. C. Gibson, P. Chuentragool, A. S. K. Lahdenperä, K. Ermanis, R. J. Phipps**

Author Contributions

A.C. Investigation:Lead; Methodology:Equal; Writing – original draft:Supporting; Writing – review & editing:Supporting
R.P. Conceptualization:Equal; Investigation:Equal; Methodology:Equal; Writing – original draft:Supporting; Writing – review & editing:Supporting
D.G. Investigation:Supporting; Methodology:Supporting
P.C. Investigation:Supporting; Methodology:Supporting
K.E. Investigation:Equal; Methodology:Equal; Writing – original draft:Supporting; Writing – review & editing:Supporting

Contents

General Experimental S3
General Procedures S5
General Procedure A S5
Additional Optimization Tables S5
Examination of Other Heterocycles Under Optimized Conditions S7
Examination of Other Alcohols Under Optimized Conditions S7
Examination of a Secondary Alcohol Under Optimized Conditions S8
Evaluation of *N*-Boc-3-Aminopyridine S8
Synthesis of Pyridines S8
Pyridine Scope – Synthesis of Products S10
Reactions of Lepidine S22
Alcohol Scope – Synthesis of Products S24
Mosher Ester Analysis for Determination of Absolute Stereochemistry S33
General Procedure B – Synthesis of Mosher Esters S33
General Procedure C – Mosher Ester Analysis S33
Control Experiments and Experiments to Probe Mechanism S43
Control Reaction with no H-Bond Donor S43
Intermolecular Competition KIE Experiment S44
Computational Investigations S45
Computational methods S45
Model study S45
Non-covalent interaction plots for lowest energy full system deprotonation transition states optimized at B3LYP/6-31G*/SMD(ethylacetate) S47
Summary of the associated computational dataset contents S48
References S52
NMR Spectra S53
SFC Traces S165

General Experimental

Reagents: unless otherwise stated, reagents were used as supplied from commercial sources without further purification. Anhydrous Ethyl acetate was purchased from Acros Organics and sparged with nitrogen for 30 min before use.

Chiral phosphoric acid catalysts (*R*)-TRIP,[1-2] (*R*)-DIP,[3-4] (*R*)-TCYP,[5] were prepared according to known procedures.

The following pyridine starting materials were prepared *via* routes previously reported in the literature: 2-cyclohexylpyridine,[6] 2-(cyclohexylmethyl)pyridine,[7] *tert*-butyl (2-(pyridin-2-yl)ethyl)carbamate, [8] 5-methyl-3-phenylpyridine,[9] and 2-methylpyridine-d_4.[10] The following alcohol starting materials were prepared *via* routes previously reported in the literature 3-(2-bromophenyl)propan-1-ol,[11] 3-(2-methoxyphenyl)propan-1-ol,[12] 3-(2-fluorophenyl)propan-1-ol[13] and 3-(4-bromophenyl)propan-1-ol.[14]

NMR spectra: ^{1}H NMR spectra were recorded on a 500 MHz Bruker Avance III BBO Smart Probe, 400 MHz QNP Cryoprobe. Chemical shifts are reported in parts per million (ppm) and the spectra are calibrated to the resonance resulting from incomplete deuteration of the solvent ($CDCl_3$: 7.26 ppm, CD_3OD: 3.31 ppm, $(CD_3)_2SO$: 2.50 ppm). ^{13}C NMR spectra were recorded the same spectrometer with complete proton decoupling. Chemical shifts are reported in ppm with the solvent resonance as the internal standard ($^{13}CDCl_3$: 77.16 ppm, t; DMSOd_6: 39.51 ppm, s). Data are reported as follows: chemical shift δ/ppm, integration (^{1}H only), multiplicity (s = singlet, d = doublet, t = triplet, q = quartet, br = broad, m = multiplet or combinations thereof; ^{13}C signals are singlets unless otherwise stated), coupling constants *J* in Hz, assignment.

Chromatography: Analytical thin layer chromatography was performed using precoated Merck glass backed silica gel plates (Silicagel 60 F254). Visualisation was by ultraviolet fluorescence (λ = 254 nm) and/or staining with $KMnO_4$ stain. Flash column chromatography was performed using silica gel 60 (0.040-0.063 µm) from Material Harvest.

Chiral SFC analysis: Performed on a Waters ACQUITY UPC2 system with YMC CHIRAL ART SA, SB, SC, SJ (4.6 × 250 mm, 3 µm) or DAICEL CHIRALPAK IE, IG, IH (4.6 × 250 mm, 3 µm) columns in a mixed solvent system of supercritical CO_2 and MeOH. A system backpressure of 138 bar was used in all cases.

Reaction setup: Reactions were carried out in different size vessels according to reaction scale. For 0.05 mmol scale reactions (w.r.t. heteroarene), 4 mL, 15 × 45mm crimp-top vials, containing a stirrer bar, sealed with a crimp seal, evacuated and purged with Nitrogen, were used. For 0.2 mmol scale reactions (w.r.t. heteroarene), 6-dram (22 mL) vials, sealed with a rubber septum, containing a stirrer bar, evacuated and purged with Nitrogen, were used. Unless stated otherwise, reactions were stirred in a cold room, set to 5 °C, under irradiation using a Kessil® PR160L 390 nm light source at 100% intensity for 24 h. For 0.05 mmol scale, the vials were placed in an arc shaped holder at 4 cm distance between the light source and central vials (**Figure 1**, as shown). For 0.2 mmol scale, the vials were placed at 2 cm distance from the light source to the centre of the vial (**Figure 2**, as shown). To offset the heat generated from the light a desk fan was mounted close to the vials.

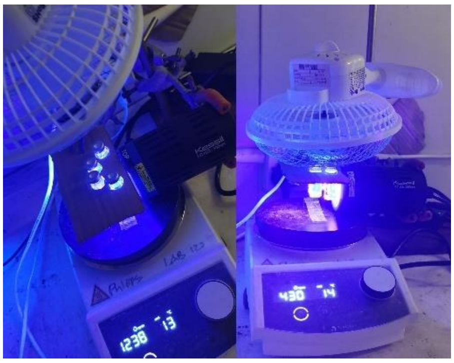

Figure 1: Photo of typical reaction set-up for screening on 0.05 mmol scale @100% intensity

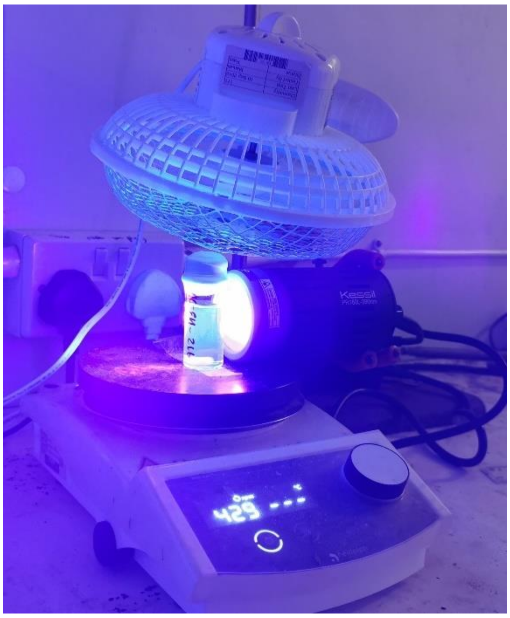

Figure 2: Photo of typical reaction set-up for exploration of scope on 0.2 mmol scale @100% intensity

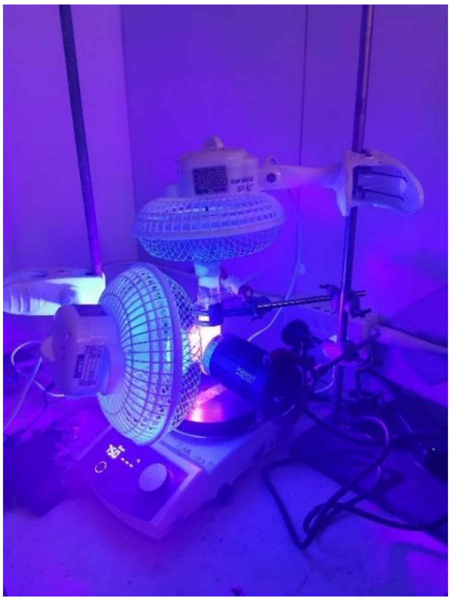

Figure 3: Photo of typical reaction for scope exploration on 0.4 mmol scale @ 100% intensity

General Procedures

General Procedure A

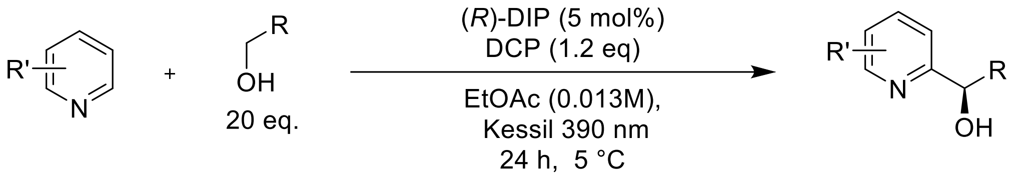

Sequentially, *N*-heteroarene (1 eq), alcohol (20 eq), dicumyl peroxide (1.2 eq) and (*R*)-DIP (0.01 mmol, 5 mol%) were weighed into a 6-dram vial containing a stirrer bar. The vial was sealed with a rubber septum and evacuated and refilled with Nitrogen three times. Anhydrous, freshly nitrogen-sparged ethyl acetate (0.013 M) was added *via* syringe. The reaction was stirred in a cold room, set to 5 °C, under irradiation with a Kessil® PR160L 390 nm light (100% intensity) at 2 cm distance (from the light to the centre of the vial), for 24 h. The heat generated from the light source was offset by mounting a desk fan close to the vial. The reaction was quenched with a few drops of triethylamine, added *via* syringe. The solvent was concentrated *in vacuo* and the crude residue was purified as specified below for each example.

Additional Optimization Tables

Table S1: Peroxide Screen

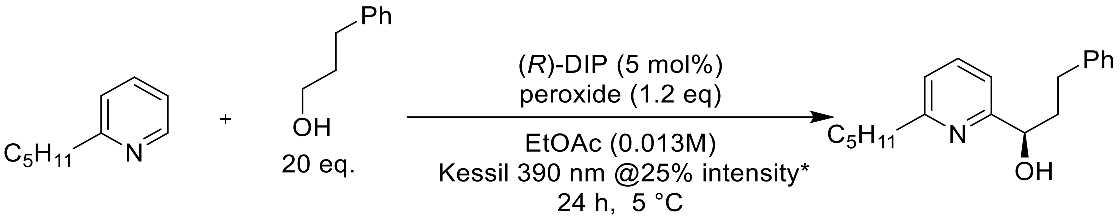

Entry	Peroxide	Product NMR yield (%)[a]	Crude ee (%)
1	Dicumylperoxide	51	85
2	Ditertbutylperoxide	39	85
3	Cumene hydroperoxide	11	87
4	Dilaurylperoxide	6	92
5	Benzoyl peroxide	16	85
6	H_2O_2	7	89
7	tert-Butyl peroxybenzoate	31	86

a. NMR yield determined by ^{1}H NMR with reference to 1,3,5-trimethoxybenzene; * =Light source set to 100% intensity but with vials at 10 cm distance to light source equating to 25% intensity

Table S2: Solvent Screen

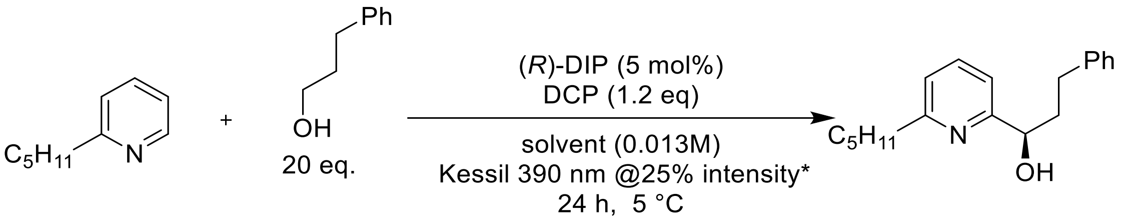

Entry	Peroxide	Product NMR yield (%)[a]	Crude ee (%)
1	EtOAc	55	85
2	tBuOAc	70	82
3	Acetone	33	77
4	MeCN	0	-
5	Trifluorotoluene	40	47
6	DCE	0	-

a. NMR yield determined by ^{1}H NMR with reference to 1,3,5-trimethoxybenzene; * =Light source set to 100% intensity but with vials at 10 cm distance to light source equating to 25% intensity

Table S3: Variation of alcohol equivalents and/or concentration of reaction

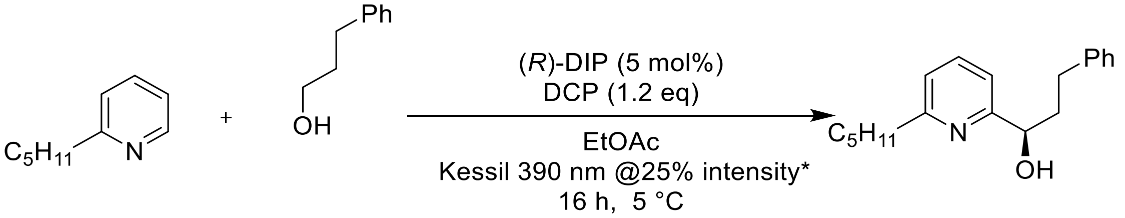

Entry	Alcohol (eq.)	EtOAc (M)	Product NMR yield (%)[a]	Crude ee (%)
1	20	0.013	50	87
2	10	0.025	44	86
3	5	0.05	35	80
4	2.5	0.1	26	80
5	10	0.013	20	90
6	10	0.05	64	84
7	10	0.1	55	80
8	5	0.018	19	86
9	5	0.025	31	83
10	5	0.05	41	81

a. NMR yield determined by ^{1}H NMR with reference to 1,3,5-trimethoxybenzene; * =Light source set to 100% intensity but with vials at 10 cm distance to light source equating to 25% intensity

Irradiation of dicumyl peroxide (DCP) with different light sources

A solution of dicumyl peroxide and 1,3,5-trimethoxybenzene in EtOAc (4 mL, 0.019M w.r.t dicumyl peroxide, 0.056M wrt trimethoxybenzene) was prepared and transferred to four crimp-top vials, containing a stirrer bar, sealed with a crimp seal each containing 1 mL of the solution. One solution was then concentrated and analysed by ^{1}H NMR analysis (Table S4, entry 4). The remaining vials were stirred for 1 h, two of which were irradiated using various light sources (see Table S4, entries 1-3). After 1 h irradiation of the vials was stopped, and the solutions were then concentrated. The consumption of dicumyl peroxide was determined by analysis of the ^{1}H NMR spectrum for each mixture with reference to 1,3,5-trimethoxybenzene as an internal standard.

Table S4: Irradiation of dicumyl peroxide (DCP) with different light sources for 1 h

Entry	Light source	Irradiation time (h)	Temperature (°C)	DCP Consumption (%)[a]
1	Kessil 390 nm	1	5	41
2	Kessil Tuna Blue	1	5	4
3	none	1	RT	0
4	none	0	RT	0

a. determined by ^{1}H NMR with reference to 1,3,5-trimethoxybenzene

Examination of Other Heterocycles Under Optimized Conditions

The following heterocycles showed no appreciable levels of conversion to the desired hydroxyalkylated product when subjected to the Minisci reaction of alcohols.

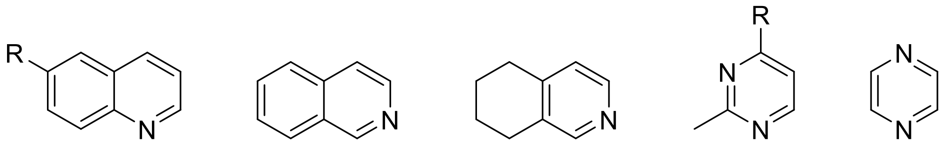

R = OMe
R = CO_2Me

R = H
R = Cl

Examination of Other Alcohols Under Optimized Conditions

The following alcohols showed either no appreciable level of conversion or very low (<10% conversion) to the desired hydroxyalkylated product when subjected to the Minisci reaction on 2-penytlpyridine.

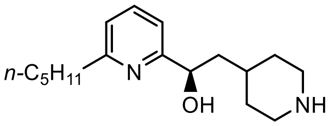

no product observed

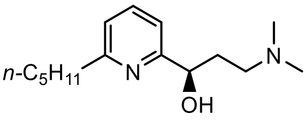

no product observed

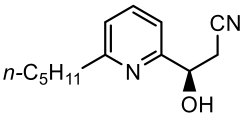

no product observed

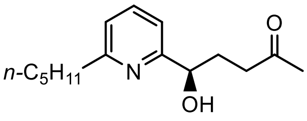

yield of product <10 %

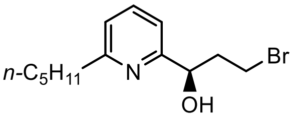

yield of product <10 %

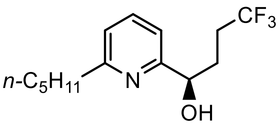

yield of product <10 %

Examination of a Secondary Alcohol Under Optimized Conditions

Racemic 1-Phenyl-2-propanolwas evaluated in the Minisci reaction on 2-pentylpyridine under the optimised conditions but showed no conversion to any desired product.

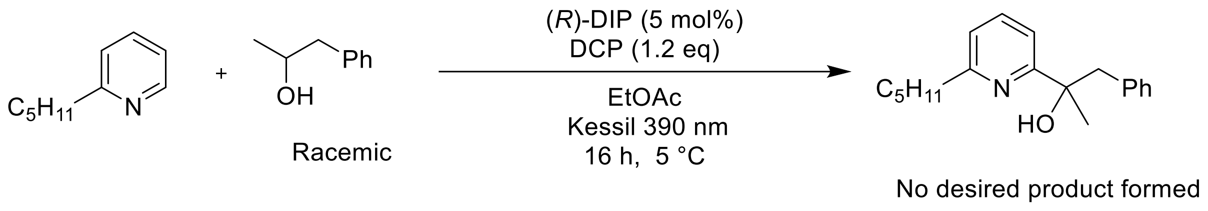

Evaluation of *N*-Boc-3-Aminopyridine

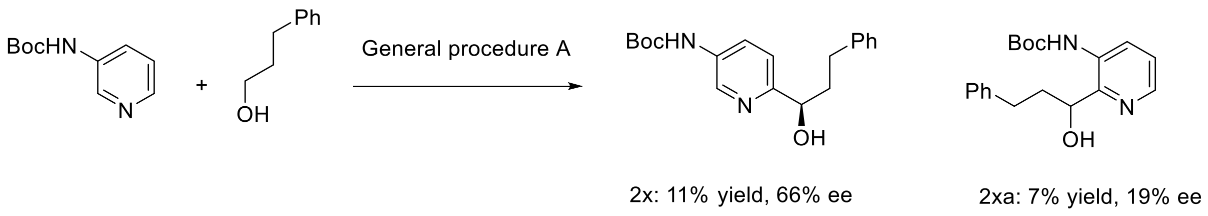

Synthesis of Pyridines

Synthesis of 2-(2-((triethylsilyl)oxy)ethyl)pyridine

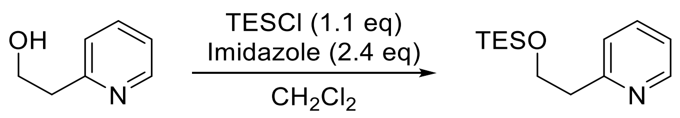

At room temperature, chlorotriethylsilane (0.5 mL, 3.0 mmol) was added *via* syringe to a solution of 2-hydroxyethylpyridine (0.3 mL, 2.7 mmol) and imidazole (436 mg, 6.4 mmol) in CH_2Cl_2 (8 mL). The mixture was stirred overnight and then diluted CH_2Cl_2 (10 mL). The mixture was transferred to a separating funnel and washed with H_2O (10 mL). The aqueous layer was further extracted with CH_2Cl_2 (2 ×10 mL). The combined organic layers were then washed with sat. NaCl (10 mL), dried over Na_2SO_4, filtered and concentrated *in vacuo*. The crude material was subjected to purification *via* flash column chromatography eluting with hexane/EtOAc 100:0 to 50:50 to give the desired product as a pale yellow oil (111 mg, 18% yield).

^{1}H NMR (500 MHz, Chloroform-*d*) δ 8.53 (ddd, *J* = 4.9, 1.9, 0.9 Hz, 1H), 7.58 (td, *J* = 7.6, 1.9 Hz, 1H), 7.20 (dt, *J* = 7.8, 1.1 Hz, 1H), 7.11 (ddd, *J* = 7.6, 4.9, 1.2 Hz, 1H), 3.98 (t, *J* = 6.7 Hz, 2H), 3.01 (t, *J* = 6.7 Hz, 2H), 0.89 (t, *J* = 7.9 Hz, 9H), 0.53 (q, *J* = 7.9 Hz, 6H).**^{13}C NMR** (126 MHz, $CDCl_3$) 159.6, 149.4, 136.2, 124.1, 121.4, 62.7, 42.0, 6.8 (3 × C), 4.5 (3 × C). **HRMS m/z:** $[M+H]^+$ calculated for $[C_{13}H_{24}NOSi]^+$ 238.1622, found: 238.1629.

Synthesis of *tert*-butyl ((5-methylpyridin-2-yl)methyl)carbamate

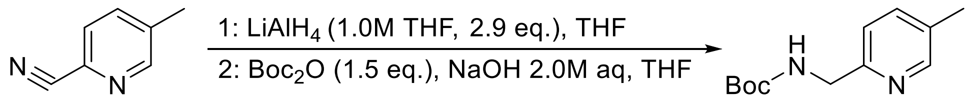

Under nitrogen atmosphere, 5-methyl-pyridine-2-carbonitrile (1.0 g, 8.5 mmol) was dissolved in anhydrous THF (45 mL). The solution was cooled to 0 °C and lithium aluminium hydride (1.0M in THF)(25 mL, 25 mmol, 2.9 eq.) was added dropwise over 20 min. The mixture was stirred at 0 °C for 30 min then sodium sulfate decahydrate was added carefully. When the evolution of gas ceased, the cold bath was removed, sodium sulfate was added and the mixture stirred vigorously at room temperature for 30 min. The suspension was filtered through Celite, and the Celite then washed with EtOAc, CH_2Cl_2 and MeOH. The filtrate was concentrated *in vacuo* to afford crude (5-methyl-pyridin-2-yl)-methylamine which was used without further purification. Crude (5-methyl-pyridin-2-yl)-methylamine was dissolved in THF (40 mL). Sodium hydroxide (2.0M aqueous solution, 12 mL) was then added followed by Boc anhydride (2.77 g, 12.7 mmol, 1.5 eq.). The resulting solution was stirred overnight at room temperature. The solution was then diluted with water (50 mL) and was extracted with ethyl acetate (2 × 150 mL). The organic layers were washed with brine (2 × 50 mL), dried over Na_2SO_4, filtered and concentrated. The crude material was subjected to purification *via* flash column chromatography eluting with petroleum ether (40-60)/EtOAc 50:50 to give the desired product as a pale yellow solid (564 mg, 30% yield).

^{1}H NMR (400 MHz, $CDCl_3$) δ ^{1}H NMR (500 MHz, $CDCl_3$) δ 8.34 (d, *J* = 2.2 Hz, 1H), 7.45 (dd, *J* = 8.1, 2.2 Hz, 1H), 7.15 (d, *J* = 7.9 Hz, 1H), 5.53 (br s, 1H), 4.38 (d, *J* = 5.5 Hz, 2H), 2.30 (s, 3H), 1.45 (s, 9H); **^{13}C NMR** (126 MHz, $CDCl_3$) δ ^{13}C NMR (101 MHz, $CDCl_3$) δ 156.1, 154.7, 149.5, 137.4, 131.7, 121.4, 79.5, 45.6, 28.5 (3 × C), 18.2. **HRMS m/z:** $[M+H]^+$ calculated for $[C_{12}H_{19}N_2O_2]^+$ 223.1441, found: 223.1442.

Pyridine Scope – Synthesis of Products

(*R*)-1-(6-pentylpyridin-2-yl)-3-phenylpropan-1-ol (2a)

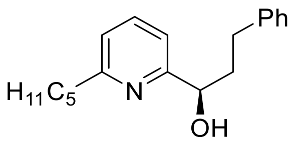

Following General Procedure A, 2-pentylpyridine (29.9 mg, 0.2 mmol), 3-phenyl-1-propanol (545 mg, 4.0 mmol, 20 eq), dicumyl peroxide (64.9 mg, 0.24 mmol, 1.2 eq) and (*R*)-DIP (6.7 mg, 0.01 mmol, 5 mol%) were used. The crude material was purified *via* an initial flash column chromatography eluting with toluene/acetonitrile 95:5, followed by subsequent flash column chromatography eluting with CH_2Cl_2/EtOH 100:0 to 99:1. A final purification by flash column chromatography eluting with hexane/ethyl acetate 80:20 afforded the desired product **2a** as a white solid (31.2 mg, 55% yield, 85% ee).

^{1}H NMR (500 MHz, $CDCl_3$) δ 7.52 (t, J = 7.7 Hz, 1H), 7.20 (t, J = 7.5 Hz, 2H), 7.16 – 7.12 (m, 2H), 7.12 – 7.07 (m, 1H), 6.98 (d, J = 7.8 Hz, 1H), 6.96 (d, J = 7.9 Hz, 1H), 4.79 (br s, 1H), 4.68 (dd, J = 8.2, 3.8 Hz, 1H), 2.78 – 2.64 (m, 4H), 2.07 (dddd, J = 13.9, 10.3, 6.6, 3.8 Hz, 1H), 1.95 – 1.84 (m, 1H), 1.67 (p, J = 7.4 Hz, 2H), 1.28 (tq, J = 9.1, 5.1 Hz, 4H), 0.83 (t, J = 6.9 Hz, 3H); **^{13}C NMR** (101 MHz, $CDCl_3$) δ 161.1, 160.8, 142.4, 137.0, 128.6 (2 × C), 128.5 (2 × C), 125.9, 121.3, 117.4, 71.5, 40.6, 38.1, 31.67, 31.65, 29.4, 22.7, 14.1. **HRMS m/z:** $[M+H]^+$ calculated for $[C_{19}H_{26}NO]^+$ 284.2009, found: 284.2011. **SFC Analysis:** CHIRAL ART SC (CO_2/MeOH = 97/03, 2.5 mL min^{-1}, 40 °C) t_R = 8.0 (minor), 8.5 (major) minutes. **$[\alpha]_D^{25.0}$** = –13.8 (c 1.0, $CHCl_3$).

(*R*)-1-(6-methylpyridin-2-yl)-3-phenylpropan-1-ol (2b)

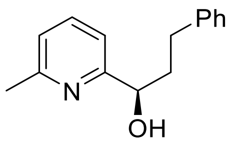

Following General Procedure A, 2-methylpyridine (18.6 mg, 0.2 mmol), 3-phenyl-1-propanol (545 mg, 4.0 mmol, 20 eq), dicumyl peroxide (64.9 mg, 0.24 mmol, 1.2 eq) and (*R*)-DIP (6.7 mg, 0.01 mmol, 5 mol%) were used. The crude material was purified *via* an initial flash column chromatography eluting with toluene/acetonitrile 95:5, followed by subsequent flash column chromatography eluting with CH_2Cl_2/EtOAc 100:0 to 90:10. to afford the desired product **2b** as a white solid (18 mg, 40% yield, 82% ee).

^{1}H NMR (500 MHz, $CDCl_3$) δ 7.55 (t, J = 7.7 Hz, 1H), 7.29 – 7.24 (m, 2H), 7.23 – 7.19 (m, 2H), 7.19 – 7.14 (m, 1H), 7.04 (d, J = 7.6 Hz, 1H), 7.01 (d, J = 8.1 Hz, 1H), 4.77 – 4.69 (m, 1H), 4.63 (br s, 1H), 2.84 – 2.72 (m, 2H), 2.55 (s, 3H), 2.13 (dddd, J = 13.9, 10.4, 6.7, 3.8 Hz, 1H), 1.95 (dddd, J = 13.7, 9.8, 8.2, 5.5 Hz, 1H).; **^{13}C NMR** (126 MHz, $CDCl_3$) δ 161.0, 157.1, 142.4, 137.1, 128.7 (2 × C), 128.5 (2 × C), 125.9, 121.9, 117.2, 71.7, 40.6, 31.8, 24.4. **HRMS m/z:** $[M+H]^+$ calculated for $[C_{15}H_{18}NO]^+$ 228.1383, found: 228.1390. **SFC Analysis:** Chiralpak IG (CO_2/MeOH = 85/15, 2.5 mL min^{-1}, 40 °C, 263 nm) t_R = 4.5 (minor), 4.8 (major) minutes. **$[\alpha]_D^{25.0}$** = –22.0 (c 1.0, $CHCl_3$).

(*R*)-1-(6-isopropylpyridin-2-yl)-3-phenylpropan-1-ol (2c)

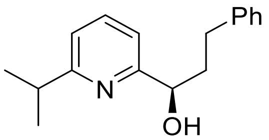

Following General Procedure A, 2-methylpyridine (25.5 mg, 0.21 mmol), 3-phenyl-1-propanol (545 mg, 4.0 mmol, 20 eq), dicumyl peroxide (64.9 mg, 0.24 mmol, 1.2 eq) and (*R*)-DIP (6.7 mg, 0.01 mmol, 5 mol%) were used. The crude material was purified *via* flash column chromatography eluting with hexane/EtOAc 100:0 to 90:10. The resultant residue was dissolved in diethyl ether (5 mL) and shaken with 3 M HCl (5 mL). The two phases were separated, and the aqueous phase was then washed with diethyl ether (1 × 5 mL), neutralised to pH 7 using sat. $NaHCO_3$ solution and extracted with CH_2Cl_2 (3 ×5 mL). The organic phase was dried using Na_2SO_4, filtered and concentrated *in vacuo* which gave the desired product **2c** as a colourless oil (21 mg, 39% yield, 80% ee).

^{1}H NMR (400 MHz, $CDCl_3$) δ 7.53 (t, J = 7.7 Hz, 1H), 7.21 – 7.16 (m, 2H), 7.16 – 7.11 (m, 2H), 7.09 (br t, J = 7.1 Hz, 1H), 7.00 (d, J = 7.6 Hz, 1H), 6.95 (d, J = 7.7 Hz, 1H), 4.94 (br s, 1H), 4.73 – 4.62 (m, 1H), 3.03 (hept, J = 7.0 Hz, 1H), 2.80 – 2.62 (m, 2H), 2.13 – 1.99 (m, 1H), 1.96 – 1.82 (m, 1H), 1.24 (d, J = 6.9 Hz, 6H); **^{13}C NMR** (101 MHz, $CDCl_3$) δ 165.7, 160.5, 142.3, 137.6, 128.7 (2 × C), 128.5 (2 × C), 125.9, 119.4, 117.7, 71.4, 40.5, 35.9, 31.7, 22.6, 22.5. **HRMS m/z:** $[M+H]^+$ calculated for $[C_{17}H_{22}NO]^+$ 256.1696, found: 256.1701. **SFC Analysis:** Chiralpak IE (CO_2/MeOH = 95/05, 2.5 mL min^{-1}, 40 °C, 262 nm) t_R = 6.3 (minor), 6.8 (major) minutes. **$[\alpha]_D^{25.0}$** = –21.8 (c 1.0, $CHCl_3$).

(*R*)-1-(6-cyclohexylpyridin-2-yl)-3-phenylpropan-1-ol (2d)

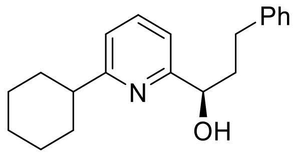

Following General Procedure A, 2-cyclohexylpyridine (32.3 mg, 0.2 mmol), 3-phenyl-1-propanol (545 mg, 4.0 mmol, 20 eq), dicumyl peroxide (64.9 mg, 0.24 mmol, 1.2 eq) and (*R*)-DIP (6.7 mg, 0.01 mmol, 5 mol%) were used. The crude material was purified *via* an initial flash column chromatography eluting with hexane/EtOAc 100:0 to 95:5, followed by subsequent flash column chromatography eluting with CH_2Cl_2/MeOH 100:0 to 99:01. to afford the desired product **2d** as a white solid (27 mg, 46% yield, 80% ee).

^{1}H NMR (400 MHz, $CDCl_3$) δ 7.59 (t, J = 7.7 Hz, 1H), 7.29 – 7.24 (m, 2H), 7.23 – 7.19 (m, 2H), 7.19 – 7.14 (m, 1H), 7.05 (d, J = 7.7 Hz, 1H), 7.01 (d, J = 7.7 Hz, 1H), 5.00 (br s, 1H), 4.79 – 4.71 (m, 1H), 2.87 – 2.68 (m, 3H), 2.19 – 2.09 (m, 1H), 2.01 – 1.91 (m, 3H), 1.90 – 1.82 (m, 2H), 1.80 – 1.72 (m, 1H), 1.55 (qt, J = 12.1, 3.5 Hz, 2H), 1.48 – 1.36 (m, 2H), 1.34 – 1.26 (m, 1H).; **^{13}C NMR** (101 MHz, $CDCl_3$) δ ^{13}C NMR (101 MHz, $CDCl_3$) δ 164.9, 160.5, 142.4, 137.4, 128.7 (2 × C), 128.5 (2 × C), 125.9, 119.7, 117.9, 71.4, 46.1, 40.6, 32.94, 32.85, 31.7, 26.63, 26.62, 26.2. **HRMS m/z:** $[M+H]^+$ calculated for $[C_{20}H_{26}NO]^+$ 296.2009, found: 296.2020. **SFC Analysis:** Chiralpak IE (CO_2/MeOH = 93/07, 2.5 mL min^{-1}, 40 °C, 262 nm) t_R = 9.3 (minor), 10.7 (major) minutes. **$[\alpha]_D^{25.0}$** = –28.5 (c 1.0, $CHCl_3$).

(*R*)-1-(6-(cyclohexylmethyl)pyridin-2-yl)-3-phenylpropan-1-ol (2e)

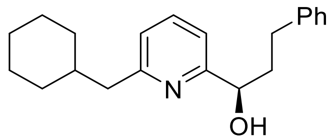

Following General Procedure A, 2-(cyclohexylmethyl)pyridine (36 mg, 0.2 mmol), 3-phenyl-1-propanol (545 mg, 4.0 mmol, 20 eq), dicumyl peroxide (64.9 mg, 0.24 mmol, 1.2 eq) and (*R*)-DIP (6.7 mg, 0.01 mmol, 5 mol%) were used. The crude material was purified *via* flash column chromatography eluting with hexane/EtOAc 100:0 to 80:20. The resultant residue was dissolved in diethyl ether (5 mL) and shaken with 3 M HCl (5 mL). The two phases were separated, and the aqueous phase was then washed with diethyl ether (1 × 5 mL), neutralised to pH 7 using sat. $NaHCO_3$ solution and extracted with CH_2Cl_2 (3 ×5 mL). The organic phase was dried using Na_2SO_4, filtered and concentrated *in vacuo* which gave the desired product **2e** as a white solid (31 mg, 49% yield, 81% ee).

^{1}H NMR (400 MHz, $CDCl_3$) δ 7.59 (t, J = 7.7 Hz, 1H), 7.31 – 7.24 (m, 2H), 7.24 – 7.14 (m, 3H), 7.07 – 6.99 (m, 2H), 4.90 (br s, 1H), 4.76 (dd, J = 8.2, 3.9 Hz, 1H), 2.86 – 2.73 (m, 2H), 2.72 – 2.65 (m, 2H), 2.14 (dddd, J = 13.8, 10.3, 6.7, 3.9 Hz, 1H), 2.05 – 1.91 (m, 1H), 1.86 – 1.74 (m, 1H), 1.73 – 1.59 (m, 5H), 1.30 – 1.15 (m, 3H), 1.08 – 0.94 (m, 2H).; **^{13}C NMR** (126 MHz, $CDCl_3$) δ 160.8, 159.9, 142.4, 136.8, 128.7 (2 × C), 128.5 (2 × C), 126.0, 122.2, 117.3, 71.5, 46.0, 40.7, 38.5, 33.34, 33.25, 31.7, 26.6, 26.37, 26.35. **HRMS m/z:** $[M+H]^+$ calculated for $[C_{21}H_{28}NO]^+$ 310.2165, found: 310.2174. **SFC Analysis:** Chiralpak IE (CO_2/MeOH = 93/07, 2.5 mL min^{-1}, 40 °C, 263 nm) t_R = 9.8 (minor), 10.2 (major) minutes. **$[\alpha]_D^{25.0}$** = –24.0 (c 1.0, $CHCl_3$).

***tert*-butyl (*R*)-(2-(6-(1-hydroxy-3-phenylpropyl)pyridin-2-yl)ethyl)carbamate (2f)**

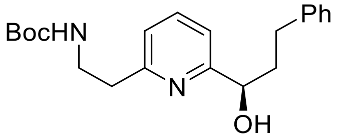

Following General Procedure A, *tert*-butyl (2-(pyridin-2-yl)ethyl)carbamate (47 mg, 0.21 mmol), 3-phenyl-1-propanol (545 mg, 4.0 mmol, 20 eq), dicumyl peroxide (64.9 mg, 0.24 mmol, 1.2 eq) and (*R*)-DIP (6.7 mg, 0.01 mmol, 5 mol%) were used. The crude material was purified *via* flash column chromatography eluting with petroleum ether (40-60)/EtOAc 65:35 to 50:50, to afford the desired product **2f** as a colourless oil (30 mg, 55% yield, 86% ee).

^{1}H NMR (400 MHz, $CDCl_3$) δ 7.61 (t, *J* = 7.7 Hz, 1H), 7.30 – 7.24 (m, 2H), 7.23 – 7.14 (m, 3H), 7.09 (app d, *J* = 7.8 Hz, 2H), 4.85 (br s, 1H), 4.75 (dd, *J* = 8.2, 3.9 Hz, 1H), 4.45 (br s, 1H), 3.55 (q, *J* = 6.5 Hz, 2H), 3.01 (t, *J* = 6.7 Hz, 2H), 2.85 – 2.71 (m, 2H), 2.19 – 2.09 (m, 1H), 2.04 – 1.93 (m, 1H), 1.42 (s, 9H).; **^{13}C NMR** (101 MHz, $CDCl_3$) δ 161.4, 157.9, 156.1, 142.1, 137.6, 128.6 (2 × C), 128.5 (2 × C), 125.9, 122.2, 118.3, 79.4, 71.8, 40.5, 40.0, 37.7, 31.7, 28.5 (3 × C). **HRMS m/z:** $[M+H]^+$ calculated for $[C_{21}H_{29}N_2O_3]^+$ 357.2173, found: 357.2176. **SFC Analysis: SFC Analysis:** CHIRAL ART SC (CO_2/MeOH = 90/10, 2.5 mL min^{-1}, 40 °C, 262 nm) t_R = 7.2 (minor), 7.6 (major) minutes. **$[\alpha]_D^{25.0}$** = –12.3 (*c* 1.0, $CHCl_3$).

(*R*)-1-(6-(2-hydroxyethyl)pyridin-2-yl)-3-phenylpropan-1-ol (2ga)

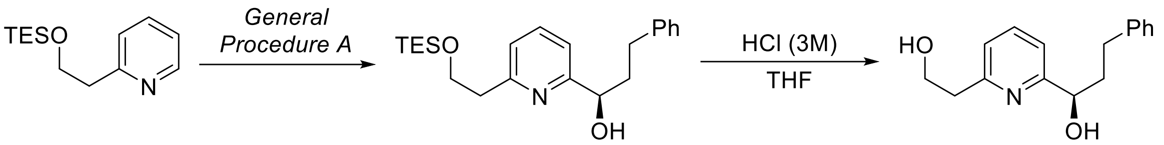

2g
not isolated

2ga

Following General Procedure A, 2-(2-((triethylsilyl)oxy)ethyl)pyridine (46.8 mg, 0.197 mmol), 3-phenyl-1-propanol (545 mg, 4.0 mmol, 20 eq), dicumyl peroxide (64.9 mg, 0.24 mmol, 1.2 eq) and (*R*)-DIP (6.7 mg, 0.01 mmol, 5 mol%) were used. Following triethylamine quench, the reaction mixture was concentrated *in vacuo* and the resulting crude mixture was dissolved in THF (3 mL). Aqueous 3M HCl (3 mL) was added and the mixture was stirred vigorously for 2.5 h. The mixture was then transferred to a separating funnel and extracted with diethyl ether (2 ×3 mL). The aqueous layer was neutralised to pH 7 using sat. $NaHCO_3$ solution and extracted with CH_2Cl_2 (3 ×5 mL). The organic phase was dried using Na_2SO_4, filtered and concentrated *in vacuo*. The residue obtained was subjected to flash column chromatography eluting with EtOAc 100%, to afford the desired product **2g** as a yellow oil (25 mg, 49% yield, 82% ee).

^{1}H NMR (400 MHz, $CDCl_3$) δ 7.57 (t, *J* = 7.6 Hz, 1H), 7.24 – 7.15 (m, 2H), 7.16 – 7.06 (m, 4H), 7.04 (d, *J* = 7.7 Hz, 1H), 4.74 – 4.63 (m, 1H), 3.95 (br t, *J* = 5.4 Hz, 2H), 2.98 (br t, *J* = 5.6 Hz, 2H), 2.71 (app t, *J* = 8.0 Hz, 2H), 2.13 – 2.01 (m, 1H), 2.01 – 1.89 (m, 1H). **^{13}C NMR** (101 MHz, $CDCl_3$) δ 161.6, 158.7, 141.9, 138.0, 128.6 (2 × C), 128.5 (2 × C), 126.0, 122.5, 118.5, 72.3, 61.9, 40.1, 39.4, 31.8. **HRMS m/z:** $[M+H]^+$ calculated for $[C_{16}H_{20}NO_2]^+$ 258.1489, found: 258.1497. **SFC Analysis:** Chiralpak IG (CO_2/MeOH = 80/20, 2.5 mL min^{-1}, 40 °C, 263 nm), t_R = 7.8 (major), 10.0 (minor) minutes. $[\alpha]_D^{25.0}$ = –3.2(*c* 1.0, $CHCl_3$).

(*R*)-3-phenyl-1-(6-(3-phenylpropyl)pyridin-2-yl)propan-1-ol (2h)

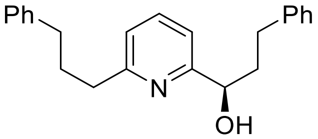

Following General Procedure A, 2-(3-phenylpropyl)pyridine (39.5 mg, 0.2 mmol), 3-phenyl-1-propanol (545 mg, 4.0 mmol, 20 eq), dicumyl peroxide (64.9 mg, 0.24 mmol, 1.2 eq) and (*R*)-DIP (6.7 mg, 0.01 mmol, 5 mol%) were used. The crude material was purified *via* an initial flash column chromatography eluting with toluene/MeCN 95:5, followed by subsequent flash column chromatography eluting with CH_2Cl_2/EtOAc 100:0 to 96:04 to afford the desired product **2h** as a colourless oil (30 mg, 45% yield, 82% ee).

^{1}H NMR (500 MHz, $CDCl_3$) δ 7.50 (t, J = 7.7 Hz, 1H), 7.23 – 7.17 (m, 4H), 7.16 – 7.07 (m, 6H), 6.96 (d, J = 7.7 Hz, 1H, overlapping with resonance δ 6.95 (d, 1H)), 6.95 (d, J = 7.8 Hz, 1H, overlapping with resonance δ 6.96 (d, 1H)), 4.73 – 4.63 (m, 2H), 2.76 (t, J = 7.8 Hz, 2H. overlapping with resonance δ 2.74 – 2.64 (m, 2H)), 2.74 – 2.64 (m, 2H, overlapping with resonance δ 2.76 (t, 2H)), 2.61 (t, J = 7.8 Hz, 2H), 2.11 – 1.97 (m, 3H), 1.87 (dddd, J = 13.4, 10.2, 7.6, 5.3 Hz, 1H); **^{13}C NMR** (126 MHz, $CDCl_3$) δ 160.9, 160.5, 142.3, 142.2, 137.1, 128.7 (2 × C), 128.6 (2 × C), 128.5 (4 × C), 126.0, 125.9, 121.5, 117.6, 71.6, 40.7, 37.5, 35.6, 31.7, 31.2. **HRMS m/z:** $[M+H]^+$ calculated for $[C_{23}H_{26}NO]^+$ 332.2009, found: 332.2017.

SFC Analysis: Chiralpak IE (CO_2/MeOH = 90/10, 2.5 mL min^{-1}, 40 °C, 263 nm) t_R = 10.2 (minor), 10.8 (major) minutes. **[α]$_D^{25.0}$** = –23.4 (c 1.0, $CHCl_3$).

(*R*)-1-(5,6-dimethylpyridin-2-yl)-3-phenylpropan-1-ol (2i)

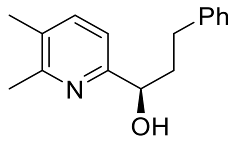

Following General Procedure A, 2,3-dimethylpyridine (21.4 mg, 0.2 mmol), 3-phenyl-1-propanol (545 mg, 4.0 mmol, 20 eq), dicumyl peroxide (64.9 mg, 0.24 mmol, 1.2 eq) and (*R*)-DIP (6.7 mg, 0.01 mmol, 5 mol%) were used. The crude material was purified *via* an initial flash column chromatography eluting with toluene/MeCN 95:05, followed by subsequent flash column chromatography eluting with CH_2Cl_2/EtOAc 100:0 to 95:05 to afford the desired product **2i** as a colourless oil (22 mg, 46% yield, 83% ee).

^{1}H NMR (400 MHz, $CDCl_3$) δ 7.40 (d, J = 7.8 Hz, 1H), 7.31 – 7.22 (m, 2H), 7.24 – 7.12 (m, 3H), 6.96 (d, J = 7.7 Hz, 1H), 4.71 (br s, 1H, overlapping with resonance δ 4.71 (dd, 1H)), 4.71 (dd, J = 8.2, 3.9 Hz, 1H, overlapping with resonance δ 4.71 (s, 1H)), 2.86 – 2.69 (m, 2H), 2.51 (s, 3H), 2.27 (s, 3H), 2.17 – 2.06 (m, 1H), 2.01 – 1.89 (m, 1H). **^{13}C NMR** (101 MHz, $CDCl_3$) δ 158.1, 155.5, 142.4, 138.3, 130.1, 128.7 (2 × C), 128.5 (2 × C), 125.8, 117.7, 71.4, 40.5, 31.7, 22.4, 18.8. **HRMS m/z:** $[M+H]^+$ calculated for $[[C_{16}H_{20}NO]^+$ 242.1539, found: 242.1548. **SFC Analysis:** Chiralpak IE (CO_2/MeOH = 85/15, 2.5 mL min^{-1}, 40 °C, 267 nm) t_R = 4.6 (minor), 4.8 (major) minutes. **[α]$_D^{25.0}$** = –7.5 (c 0.5, $CHCl_3$).

(*R*)-1-(6,7-dihydro-5H-cyclopenta-pyridin-2-yl)-3-phenylpropan-1-ol (2j)

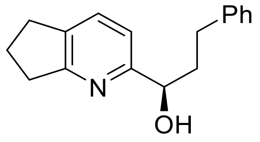

Following General Procedure A, 2,3-cyclopentenopyridine (23.8 mg, 0.2 mmol), 3-phenyl-1-propanol (545 mg, 4.0 mmol, 20 eq), dicumyl peroxide (64.9 mg, 0.24 mmol, 1.2 eq) and (*R*)-DIP (6.7 mg, 0.01 mmol, 5 mol%) were used. The crude material was purified *via* an initial flash column chromatography eluting with CH_2Cl_2/Et_2O 100:0 to 98:02, followed by subsequent flash column chromatography eluting with CH_2Cl_2/MeOH 99:01 The resultant residue was dissolved in diethyl ether (5 mL) and shaken with 3 M HCl (5 mL). The two phases were separated, and the aqueous phase was then washed with diethyl ether (1 × 5 mL), neutralised to pH 7 using sat. $NaHCO_3$ solution and extracted with CH_2Cl_2 (3 ×5 mL). The organic phase was dried using Na_2SO_4, filtered and concentrated *in vacuo* which gave the desired product **2j** as a white solid (18 mg, 36% yield, 88% ee).

^{1}H NMR (400 MHz, $CDCl_3$) δ 7.47 (d, J = 7.8 Hz, 1H), 7.30 – 7.24 (m, 2H), 7.23 – 7.14 (m, 3H), 6.97 (d, J = 7.8 Hz, 1H), 4.74 (dd, J = 8.1, 4.1 Hz, 1H), 3.01 (t, J = 7.7 Hz, 2H), 2.92 (t, J = 7.4 Hz, 2H), 2.82 – 2.73 (m, 2H), 2.20 – 2.07 (m, 3H), 2.05 – 1.93 (m, 1H).; **^{13}C NMR** (101 MHz, $CDCl_3$) δ 164.5, 159.9, 142.3, 135.8, 132.8, 128.7 (2 × C), 128.5 (2 × C), 125.8, 117.7, 72.2, 40.7, 34.1, 31.8, 30.6, 23.3. **HRMS m/z:** $[M+H]^+$ calculated for $[C_{17}H_{20}NO]^+$ 254.1539, found: 254.1542. **SFC Analysis:** Chiralpak IH (CO_2/MeOH= 93/07, 2.5 mL min^{-1}, 40 °C, 273 nm), t_R = 6.1 (major), 7.5 (minor) minutes. **[α]$_D^{25.0}$** = –12.7 (c 1.0, $CHCl_3$).

(*R*)-3-phenyl-1-(5,6,7,8-tetrahydroquinolin-2-yl)propan-1-ol (2k)

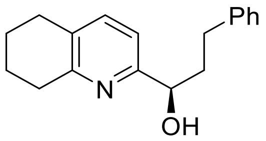

Following General Procedure A, 5,6,7,8-tetrahydroquinoline (26.6 mg, 0.2 mmol), 3-phenyl-1-propanol (545 mg, 4.0 mmol, 20 eq), dicumyl peroxide (64.9 mg, 0.24 mmol, 1.2 eq) and (*R*)-DIP (6.7 mg, 0.01 mmol, 5 mol%) were used. The crude material was purified *via* flash column chromatography eluting with hexane/EtOAc 75:25 to 65:35. The resultant residue was dissolved in diethyl ether (5 mL) and shaken with 3 M HCl (5 mL). The two phases were separated, and the aqueous phase was then washed with diethyl ether (1 × 5 mL), neutralised to pH 7 using sat. $NaHCO_3$ solution and extracted with CH_2Cl_2 (3 ×5 mL). The organic phase was dried using Na_2SO_4, filtered and concentrated *in vacuo* which gave the desired product **2k** as a colourless oil (23 mg, 43% yield, 88% ee).

^{1}H NMR (400 MHz, $CDCl_3$) δ 7.28 (d, J = 7.9 Hz, 1H), 7.22 – 7.17 (m, 2H), 7.16 – 7.06 (m, 3H), 6.89 (d, J = 7.8 Hz, 1H), 4.67 (br s, 1H, overlapping with resonance δ 4.64 (dd, 1H)), 4.64 (dd, J = 8.2, 3.9 Hz, 1H, overlapping with resonance δ 4.67 (s, 1H)), 2.86 (t, J = 6.4 Hz, 2H), 2.79 – 2.61 (m, 4H), 2.05 (dddd, J = 13.5, 9.6, 7.3, 3.7 Hz, 1H), 1.95 – 1.86 (m, 1H), 1.86 – 1.78 (m, 2H), 1.78 – 1.70 (m, 2H).; **^{13}C NMR** (101 MHz, $CDCl_3$) δ 158.4, 155.7, 142.4, 138.1, 131.1, 128.7 (2 × C), 128.5 (2 × C), 125.8, 117.5, 71.4, 40.4, 32.2, 31.8, 28.5, 23.0, 22.8. **HRMS m/z:** $[M+H]^+$ calculated for $[C_{18}H_{22}NO]^+$ 268.1696, found: 268.2697. **SFC Analysis:** CHIRAL ART SC (CO_2/MeOH = 93/07, 2.5 mL min^{-1}, 40 °C, 270 nm) t_R = 11.1 (minor), 11.7 (major) minutes. **$[\alpha]_D^{25.0}$** = –22.8 (c 1.0, $CHCl_3$).

(*R*)-1-(5,6-dimethylpyridin-2-yl)-3-phenylpropan-1-ol (2l)

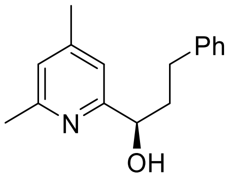

Following General Procedure A, 2,4-dimethylpyridine (23.4 mg, 0.22 mmol), 3-phenyl-1-propanol (545 mg, 4.0 mmol, 20 eq), dicumyl peroxide (64.9 mg, 0.24 mmol, 1.2 eq) and (*R*)-DIP (6.7 mg, 0.01 mmol, 5 mol%) were used. The crude material was purified *via* an initial flash column chromatography eluting with CH_2Cl_2/EtOAc 100:0 to 70:30, followed by subsequent flash column chromatography eluting with hexane/EtOAc 80:20 to afford the desired product **2l** as a colourless oil (21 mg, 40% yield, 78% ee).

^{1}H NMR (700 MHz, $CDCl_3$) δ 7.29 – 7.24 (m, 2H), 7.23 – 7.19 (m, 2H), 7.18 – 7.14 (m, 1H), 6.86 (s, 1H), 6.82 (s, 1H), 4.68 (dd, J = 8.4, 3.7 Hz, 1H, overlapping with resonance δ 4.65 (s, 1H)), 4.65 (br s, 1H, overlapping with resonance δ 4.68 (dd, 1H)), 2.84 – 2.73 (m, 2H), 2.50 (s, 3H), 2.30 (s, 3H), 2.11 (dddd, J = 13.8, 10.3, 6.8, 3.7 Hz, 1H), 1.93 (dddd, J = 13.9, 9.8, 8.2, 5.6 Hz, 1H); **^{13}C NMR** (126 MHz, $CDCl_3$) δ 160.9, 156.7, 148.2, 142.4, 128.7 (2 × C), 128.5 (2 × C), 125.8, 122.9, 118.1, 71.6, 40.6, 31.8, 24.2, 21.1. **HRMS m/z:** $[M+H]^+$ calculated for $[C_{16}H_{20}NO]^+$ 242.1539, found: 242.1538. **SFC Analysis:** Chiralpak IG(CO_2/MeOH = 90/10, 2.5 mL min^{-1}, 40 °C, 261 nm) t_R = 6.8 (minor), 7.9 (major) minutes. **$[\alpha]_D^{25.0}$** = –6.6 (c 1.0, $CHCl_3$).

(*R*)-1-(3,5-dimethylpyridin-2-yl)-3-phenylpropan-1-ol (2m)

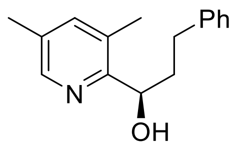

Following General Procedure A, 3,5-dimethylpyridine (21.4 mg, 0.2 mmol), 3-phenyl-1-propanol (545 mg, 4.0 mmol, 20 eq), dicumyl peroxide (64.9 mg, 0.24 mmol, 1.2 eq) and (*R*)-DIP (6.7 mg, 0.01 mmol, 5 mol%) were used. The crude material was purified *via* an initial flash column chromatography eluting with CH_2Cl_2/EtOAc 100:0 to 0:100, followed by subsequent flash column chromatography eluting with hexane/EtOAc 80:20 to afford the desired product **2m** as a colourless oil (22 mg, 33% yield, 94% ee)

Note: Despite multiple purifications via flash column chromatography, it was not possible to completely isolate compound **2m** completely free from a small amounts of a closely eluting impurity, possibly resulting from diaddition.

^{1}H NMR (500 MHz, $CDCl_3$) δ 8.21 (dd, J = 0.7 Hz, 1H), 7.28 – 7.23 (m, 3H), 7.22 – 7.19 (m, 2H), 7.18 – 7.14 (m, 1H), 4.87 – 4.69 (m, 2H), 2.84 – 2.79 (m, 2H), 2.29 (s, 3H), 2.15 (s, 3H), 2.03 – 1.94 (m, 1H), 1.84 – 1.75 (m, 1H); **^{13}C NMR** (126 MHz, $CDCl_3$) δ 156.9, 145.8, 142.3, 139.3, 131.8, 128.7 (2 × C), 128.6, 128.4 (2 × C), 125.8, 68.9, 39.6, 31.9, 18.0, 17.4. **HRMS m/z:** $[M+H]^+$ calculated for $[C_{16}H_{20}NO]^+$ 242.1539, found: 242.1548. **SFC Analysis:** Chiralpak IG (CO_2/MeOH = 85/15, 2.5 mL min^{-1}, 40 °C, 267 nm) t_R = 6.7 (major), 8.6 (minor) minutes. **$[\alpha]_D^{25.0}$** =+17.6 (c 1.0, $CHCl_3$).

(*R*)-1-(3,6-dimethylpyridin-2-yl)-3-phenylpropan-1-ol (2n)

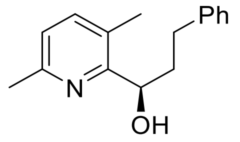

Following General Procedure A, 2,5-dimethylpyridine (21.4 mg, 0.2 mmol), 3-phenyl-1-propanol (545 mg, 4.0 mmol, 20 eq), dicumyl peroxide (64.9 mg, 0.24 mmol, 1.2 eq) and (*R*)-DIP (6.7 mg, 0.01 mmol, 5 mol%) were used. The crude material was purified *via* an initial flash column chromatography eluting with CH_2Cl_2/EtOAc 100:0 to 90:10, followed by subsequent flash column chromatography eluting with hexane/EtOAc 90:10 to 80:20 to afford the desired product **2n** as a colourless oil (18 mg, 37% yield, 91% ee).

^{1}H NMR (500 MHz, $CDCl_3$) δ 7.31 (d, J = 7.7 Hz, 1H), 7.28 – 7.24 (m, 2H), 7.23 – 7.20 (m, 2H), 7.18 – 7.14 (m, 1H), 6.95 (d, J = 7.7 Hz, 1H), 5.08 (br s, 1H), 4.78 (br d, J = 8.6 Hz, 1H), 2.89 – 2.78 (m, 2H), 2.51 (s, 3H), 2.13 (s, 3H), 1.99 (dddd, J = 13.9, 9.2, 7.6, 2.8 Hz, 1H), 1.77 (dtd, J = 13.8, 8.7, 6.1 Hz, 1H). **^{13}C NMR** (126 MHz, $CDCl_3$) δ 158.6, 154.2, 142.4, 138.9, 128.7 (2 × C), 128.4 (2 × C), 125.84, 125.77, 121.8, 68.9, 39.5, 32.1, 23.9, 17.0. HRMS m/z: $[M+H]^+$ calculated for $[C_{16}H_{20}NO]^+$ 242.1539, found: 242.1547. **SFC Analysis:** Chiralpak IG (CO_2/MeOH = 95/05, 2.5 mL min^{-1}, 40 °C, 267 nm) t_R = 10.4 (minor), 11.0 (major) minutes. **$[\alpha]_D^{25.0}$** = –1.2 (c 1.0, $CHCl_3$).

(*R*)-1-(3-methyl-5-phenylpyridin-2-yl)-3-phenylpropan-1-ol (2o)

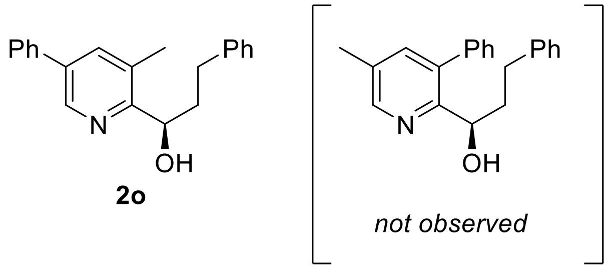

Following General Procedure A, 5-methyl-3-phenylpyridine (33.9 mg, 0.2 mmol), 3-phenyl-1-propanol (545 mg, 4.0 mmol, 20 eq), dicumyl peroxide (64.9 mg, 0.24 mmol, 1.2 eq) and (*R*)-DIP (6.7 mg, 0.01 mmol, 5 mol%) were used. Analysis of the ^{1}H NMR spectrum of the crude material determined the regioisomeric ratio to be >20:1, only resonances assigned to the major regioisomer **2o** were observed. The crude material was purified *via* an initial flash column chromatography eluting with CH_2Cl_2/EtOAc 100:0 to 90:10, followed by subsequent flash column chromatography eluting with hexane/EtOAc 100:0 to 70:30 to afford the desired product **2o** as a colourless oil (19 mg, 31% yield, 88% ee).

2o: ^{1}**H NMR** (500 MHz, $CDCl_3$) δ 8.61 (d, *J* = 2.2 Hz, 1H), 7.62 (dd, *J* = 2.2, 0.8 Hz, 1H), 7.60 – 7.53 (m, 2H), 7.51 – 7.43 (m, 2H), 7.42 – 7.37 (m, 1H), 7.30 – 7.26 (m, 2H), 7.24 – 7.21 (m, 2H), 7.20 – 7.15 (m, 1H), 4.88 (td, *J* = 7.8, 2.9 Hz, 1H), 4.76 (d, *J* = 7.5 Hz, 1H), 2.90 – 2.83 (m, 2H), 2.25 (br s, 3H), 2.09 – 2.01 (m, 1H), 1.91 – 1.81 (m, 1H); ^{13}**C NMR** (101 MHz, $CDCl_3$) δ 158.6, 144.0, 142.2, 137.7, 137.1, 135.6, 129.20 (2 × C), 129.16, 128.7 (2 × C), 128.5 (2 × C), 128.2, 127.2 (2 × C), 125.9, 69.1, 39.5, 32.0, 17.6. **HRMS m/z:** $[M+H]^+$ calculated for $[C_{21}H_{22}NO]^+$ 304.1696, found: 304.1703. **SFC Analysis:** Chiralpak IE (CO_2/MeOH = 75/25, 2.5 mL min^{-1}, 40 °C, 245) t_R = 7.2 (major), 9.02 (minor) minutes. $[\alpha]_D^{25.0}$ = –3.0 (*c* 1.0, $CHCl_3$).

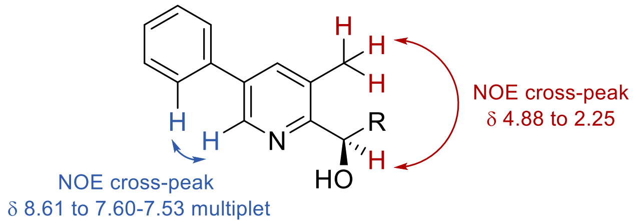

(*R*)-1-(3-methylpyridin-2-yl)-3-phenylpropan-1-ol (2pa) and (*R*)-1-(5-methylpyridin-2-yl)-3-phenylpropan-1-ol (2pb)

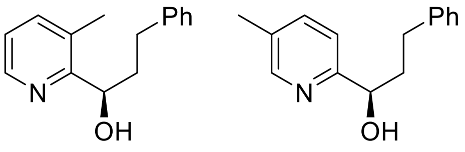

r.r. = 1.9:1

Following General Procedure A, 3-methylpyridine (37.3 mg, 0.4 mmol), 3-phenyl-1-propanol (1.09 g, 8.0 mmol, 20 eq), dicumyl peroxide (129 mg, 0.48 mmol, 1.2 eq) and (*R*)-DIP (13.4 mg, 0.02 mmol, 5 mol%) were used. The combined NMR yield of the two regioisomer products **2pa** and **2pb** was determined to be 46% with reference to 1,3,5-trimethoxybenzene as an internal standard. Analysis of the ^{1}H NMR spectrum of the crude material determined the regioisomeric ratio to be 1.9:1, determined by the relative integrations of diagnostic resonances for each regioisomer. The crude material was purified *via* an initial flash column chromatography eluting with CH_2Cl_2/EtOAc 100:0 to 50:50, followed by subsequent flash column chromatography eluting with hexane/EtOAc 85:15 which separated the two regioisomers, affording major regioisomer **2pa** as a colourless oil (28 mg, 31% yield, 90% ee).

Crude **2pb** was further purified *via* flash column chromatography eluting with $CHCl_3$/Et_2O 80:20 to afford the desired product **2pb** as a colourless oil (22 mg, 24% yield, 76% ee).

2pa (major regioisomer): ^{1}H NMR (500 MHz, $CDCl_3$) δ 8.32 (dd, J = 4.8, 0.9 Hz, 1H), 7.36 (ddd, J = 7.6, 1.6, 0.8 Hz, 1H), 7.23 – 7.18 (m, 2H), 7.17 – 7.11 (m, 2H), 7.13 – 7.06 (m, 1H), 7.05 (dd, J = 7.6, 4.8 Hz, 1H), 4.76 (dd, J = 8.4, 2.1 Hz, 1H), 2.80 – 2.73 (m, 2H), 2.11 (s, 3H), 1.99 – 1.89 (m, 1H), 1.78 – 1.69 (m, 1H); **^{13}C NMR** (126 MHz, $CDCl_3$) δ 159.8, 145.6, 142.3, 138.5, 129.3, 128.7 (2 × C), 128.5 (2 × C), 125.9, 122.4, 69.1, 39.5, 32.0, 17.5. **HRMS m/z:** $[M+H]^+$ calculated for $[C_{15}H_{18}NO]^+$ 228.1383, found: 228.1388. **SFC Analysis:** Chiralpak IG (CO_2/MeOH = 85/15, 2.5 mL min^{-1}, 40 °C, 262 nm) t_R = 5.3 (major), 6.9 (minor) minutes. **$[\alpha]_D^{25.0}$** = +16.6 (c 1.0, $CHCl_3$).

2pb (minor regioisomer): ^{1}H NMR (500 MHz, $CDCl_3$) δ 8.37 (br s, 1H), 7.48 (ddd, J = 7.9, 2.2, 0.8 Hz, 1H), 7.28 – 7.25(m, 2H), 7.22 – 7.19 (m, 2H), 7.19 – 7.15 (m, 1H), 7.13 (d, J = 8.0 Hz, 1H), 4.74 (dd, J = 8.1, 4.0 Hz, 1H), 4.25 (br s, 1H), 2.82 – 2.70 (m, 2H), 2.34 (s, 3H), 2.16 – 2.08 (m, 1H), 2.02 – 1.93 (m, 1H); **^{13}C NMR** (126 MHz, $CDCl_3$) δ 159.1, 148.6, 142.3, 137.5, 131.9, 128.6 (2 × C), 128.5 (2 × C), 125.9, 119.9, 71.9, 40.5, 31.7, 18.2. **HRMS m/z:** $[M+H]^+$ calculated for $[C_{15}H_{18}NO]^+$ 228.1383, found: 228.1384. **SFC Analysis:** Chiralpak IG (CO_2/MeOH = 85/15, 2.5 mL min^{-1}, 40 °C, 265 nm) t_R = 8.0 (major), 9.5 (minor) minutes. **$[\alpha]_D^{25.0}$** = +2.9 (c 0.5, $CHCl_3$).

tert-butyl (_R_)-((6-(1-hydroxy-3-phenylpropyl)-5-methylpyridin-2-yl)methyl)carbamate (2q)

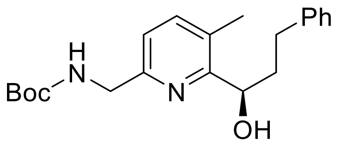

Following General Procedure A, *tert*-butyl ((5-methylpyridin-2-yl)methyl)carbamate (44.4 mg, 0.2 mmol), 3-phenyl-1-propanol (545 mg, 4.0 mmol, 20 eq), dicumyl peroxide (64.9 mg, 0.24 mmol, 1.2 eq) and (*R*)-DIP (6.7 mg, 0.01 mmol, 5 mol%) were used. The crude material was purified *via* flash column chromatography eluting with hexane/EtOAc 75:25 to afford the desired product **2q** as a colourless oil (30 mg, 42% yield, 90% ee).

^{1}H NMR (500 MHz, $CDCl_3$) δ 7.41 (d, *J* = 7.8 Hz, 1H), 7.29 – 7.24 (m, 2H), 7.23 – 7.19 (m, 2H), 7.19 – 7.15 (m, 1H), 7.10 (d, *J* = 7.7 Hz, 1H), 5.28 (br s, 1H), 4.81 (br d, *J* = 7.7 Hz, 1H), 4.76 (br s, 1H), 4.41 (d, *J* = 5.8 Hz, 2H), 2.90 – 2.79 (m, 2H), 2.16 (s, 3H), 2.03 – 1.95 (m, 1H), 1.85 – 1.75 (m, 1H), 1.46 (s, 9H); **^{13}C NMR** (126 MHz, $CDCl_3$) δ 159.1, 156.2, 153.8, 142.1, 139.5, 128.6 (2 × C), 128.5 (2 × C), 127.8, 125.9, 120.3, 79.8, 69.0, 45.5, 39.5, 32.0, 28.6 (3 × C), 17.2. **HRMS m/z:** $[M+H]^+$ calculated for $[C_{21}H_{29}N_2O_3]^+$ 357.2173, found: 357.2162. **SFC Analysis:** Chiralpak IE (CO_2/MeOH = 80/20, 2.5 mL min^{-1}, 40 °C, 266 nm) t_R = 5.5 (major), 6.0 (minor) minutes. $[\alpha]_D^{25.0}$ = –5.3 (*c* 1.0, $CHCl_3$).

(_R_)-1-(3-ethyl-6-methylpyridin-2-yl)-3-phenylpropan-1-ol (2r) isolated as (_R_)-1-(3-ethyl-6-methylpyridin-2-yl)-3-phenylpropyl acetate (2ra)

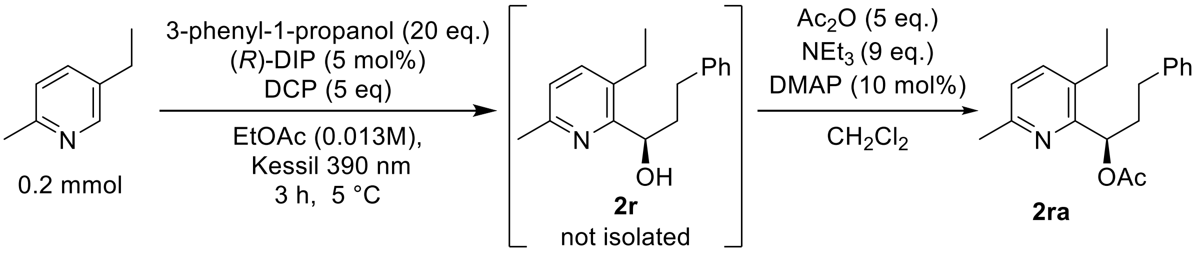

In a slight modification to the General Procedure A, 5-ethyl-2-methylpyridine (24.2 mg, 0.2 mmol), 3-phenyl-1-propanol (545 mg, 4.0 mmol, 20 eq), dicumyl peroxide (270 mg, 1.0 mmol, 1.2 eq) and (*R*)-DIP (6.7 mg, 0.01 mmol, 5 mol%) were used. The reaction mixture was stirred under irradiation for 3 h. The crude material was purified *via* an initial flash column chromatography eluting with CH_2Cl_2/EtOAc 100:0 to 50:50 to remove the excess alcohols. The mixture obtained was then dissolved in CH_2Cl_2 (5 mL). Sequentially, triethylamine (0.25 mL, 1.8 mmol, 9 eq.), DMAP (2.4 mg, 0.02 mmol, 10 mol%) and acetic anhydride (0.1 mL, 1.0 mmol, 5 eq.) were added. The mixture was stirred at room temperature for 16 h. The solvent was then concentrated *in* vacuo and the crude mixture was subjected to flash column chromatography eluting with hexane/EtOAc 95:05 to afford the desired acylated product **2ra** as a colourless oil (23 mg, 39% yield, 90% ee).

Note: Despite purification via flash column chromatography, it was not possible to completely isolate compound **2ra** 100% free from small amounts of closely eluting unidentified impurities.

^{1}**H NMR** (500 MHz, $CDCl_3$) δ 7.35 (d, *J* = 7.8 Hz, 1H), 7.29 – 7.25 (m, 2H), 7.21 – 7.16 (m, 3H), 6.99 (d, *J* = 7.9 Hz, 1H), 5.97 (dd, *J* = 9.1, 4.9 Hz, 1H), 2.81 (ddd, *J* = 13.6, 9.9, 5.3 Hz, 1H), 2.71 – 2.56 (m, 3H), 2.52 (s, 3H), 2.47 – 2.37 (m, 1H), 2.15 – 2.07 (m, 1H, overlapping with resonance δ 2.11 (s, 3H)), 2.11 (s, 3H, overlapping with resonance δ 2.15 – 2.07 (m, 1H)), 1.14 (t, *J* = 7.6 Hz, 3H); ^{13}**C NMR** (126 MHz, $CDCl_3$) δ 171.0, 155.8, 155.1, 141.5, 137.1, 133.7, 128.544 (2 × C), 128.536 (2 × C), 126.1, 122.8, 72.2, 36.0, 32.3, 24.3, 24.2, 21.4, 15.1. **HRMS m/z:** $[M+H]^+$ calculated for $[C_{19}H_{24}NO_2]^+$ 298.1802, found: 298.1806. **SFC Analysis:** Chiral ART SC (CO_2/MeOH = 97/03, 1.5 mL min^{-1}, 40 °C. 267 nm) t_R = 7.5 (minor), 8.0 (major) minutes. $[\alpha]_D^{25.0}$ = +18.0 (*c* 1.0, $CHCl_3$).

(*R*)-3-phenyl-1-(pyridin-2-yl)propan-1-ol (2s) and (1*R*,1'R)-1,1'-(pyridine-2,6-diyl)bis(3-phenylpropan-1-ol (2u) isolated as (*R*)-3-phenyl-1-(pyridin-2-yl)propyl acetate (2sa) and (1*R*,1'*R*)-pyridine-2,6-diylbis(3-phenylpropane-1,1-diyl) diacetate (2ua)

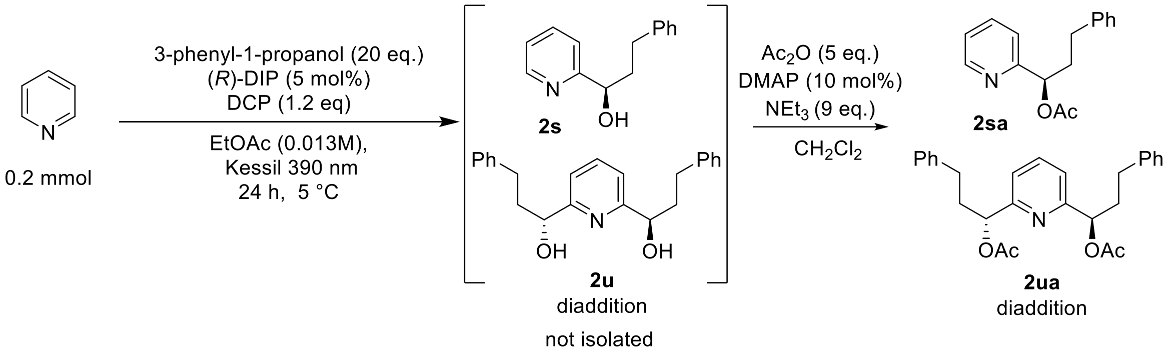

Following General Procedure A, pyridine (16 µL, 0.2 mmol), 3-phenyl-1-propanol (545 mg, 4.0 mmol, 20 eq), dicumyl peroxide (64.9 mg, 0.24 mmol, 1.2 eq) and (*R*)-DIP (6.7 mg, 0.01 mmol, 5 mol%) were used. The crude material was purified *via* an initial flash column chromatography eluting with CH_2Cl_2/EtOAc 100:0 to 50:50 to remove the excess alcohols, followed by a subsequent flash column chromatography eluting with hexane/EtOAc 85:15 to 80:20. The mixture obtained was then dissolved in CH_2Cl_2 (5 mL). Sequentially, triethylamine (0.25 mL, 1.8 mmol, 9 eq.), DMAP (2.4 mg, 0.02 mmol, 10 mol%) and acetic anhydride (0.1 mL, 1.0 mmol, 5 eq.) were added. The mixture was stirred at room temperature for 16 h. The solvent was then concentrated *in* vacuo and the crude mixture was subjected to flash column chromatography eluting with hexane/EtOAc 100:0 to 70:30 to afford the acylated product **2sa** as a colourless oil (12 mg, 24% yield, 73% ee) and diacetylated product **2ua** as a colourless oil (4 mg, 5% yield, 95% ee).

Note: Despite multiple purifications via flash column chromatography, it was not possible to completely isolate compound **2ua** 100% free from small amounts of closely eluting unidentified impurities.

2sa mono-addition product: ^{1}**H NMR** (500 MHz, $CDCl_3$) δ 8.62 (ddd, *J* = 4.8, 1.8, 0.9 Hz, 1H), 7.70 (td, *J* = 7.7, 1.8 Hz, 1H), 7.32 (dt, *J* = 7.9, 1.1 Hz, 1H), 7.31 – 7.26 (m, 2H), 7.23 (ddd, *J* = 7.7, 4.9, 1.2 Hz, 1H, overlapping with resonance δ 7.21 – 7.17 (m, 3H), 7.21 – 7.17 (m, 3H, overlapping with resonance δ 7.23 (ddd, 1H)), 5.84 (dd, *J* = 7.9, 5.5 Hz, 1H), 2.76 – 2.70 (m, 1H), 2.70 – 2.65 (m, 1H), 2.38 – 2.31 (m, 1H), 2.31 – 2.24 (m, 1H), 2.15 (s, 3H); ^{13}**C NMR** (126 MHz, $CDCl_3$) δ 170.6, 159.4, 149.6, 141.3, 136.9,

128.6 (2 × C), 128.5 (2 × C), 126.1, 122.9, 121.3, 76.3, 36.5, 31.9, 21.3. **HRMS m/z:** $[M+H]^+$ calculated for $[C_{16}H_{18}NO_2]^+$ 256.1332, found: 256.1339. **SFC Analysis:** Chiralpak IE (CO_2/MeOH = 95/05, 2.5 mL min^{-1}, 40 °C, 258 nm) t_R = 7.9 (major), 9.8 (minor) minutes. $[\alpha]_D^{25.0}$ = +20.3 (*c* 1.0, $CHCl_3$).

2ua di-addition product: ^{1}**H NMR** (500 MHz, $CDCl_3$) δ 7.66 (t, *J* = 7.7 Hz, 1H), 7.28 – 7.23 (m, 4H), 7.21 (d, *J* = 7.8 Hz, 2H), 7.19 – 7.14 (m, 6H), 5.87 (dd, *J* = 7.6, 5.5 Hz, 2H), 2.73 – 2.61 (m, 4H), 2.36 – 2.23 (m, 4H), 2.11 (s, 6H); ^{13}**C NMR** (126 MHz, $CDCl_3$) δ 170.5 (2 × C), 159.1 (2 × C), 141.5 (2 × C), 137.5, 128.6 (4 × C), 128.5 (4 × C), 126.1 (2 × C), 120.0 (2 × C), 76.1 (2 × C), 36.4 (2 × C), 31.8 (2 × C), 21.3 (2 × C). **HRMS m/z:** $[M+H]^+$ calculated for $[C_{27}H_{30}NO_4]^+$ 432.2169, found: 432.2172. **SFC Analysis:** Chiralpak IE (CO_2/MeOH = 90/10, 2.5 mL min^{-1}, 40 °C, 262 nm) t_R = 5.4 major), 5.8 (minor) minutes.

(*R*)-1-(4-(*tert*-butyl)pyridin-2-yl)-3-phenylpropan-1-ol (2t)

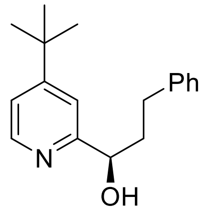

Following General Procedure A, 4-*tert*butylpyridine (27 mg, 0.2 mmol), 3-phenyl-1-propanol (545 mg, 4.0 mmol, 20 eq), dicumyl peroxide (64.9 mg, 0.24 mmol, 1.2 eq) and (*R*)-DIP (6.7 mg, 0.01 mmol, 5 mol%) were used. The crude material was purified *via* an initial flash column chromatography eluting with CH_2Cl_2/EtOAc 100:0 to 50:50, followed by subsequent flash column chromatography eluting with $CHCl_3$/Et_2O 80:20 to afford the desired product **2t** as a colourless oil (22 mg, 41% yield).

^{1}**H NMR** (500 MHz, $CDCl_3$) δ 8.46 – 8.41 (m, 1H), 7.30 – 7.26 (m, 2H), 7.23 – 7.20 (m, 2H), 7.20 – 7.15 (m, 3H), 4.75 (dd, *J* = 8.3, 4.0 Hz, 1H), 4.28 (br s, 1H), 2.84 – 2.75 (m, 2H), 2.18 – 2.10 (m, 1H), 2.03 – 1.94 (m, 1H), 1.31 (s, 9H); ^{13}**C NMR** (126 MHz, $CDCl_3$) δ 161.8, 161.1, 148.1, 142.3, 128.7 (2 × C), 128.5 (2 × C), 125.9, 119.8, 117.2, 72.4, 40.7, 35.0, 31.9, 30.7 (3 × C). **HRMS m/z:** $[M+H]^+$ calculated for $[C_{18}H_{24}NO]^+$ 270.1852, found: 270.1855. $[\alpha]_D^{25.0}$ = +13.0 (*c* 1.0, $CHCl_3$).

(*R*)-1-(4-(tert-butyl)pyridin-2-yl)-3-phenylpropyl acetate for determination of ee of 2ta

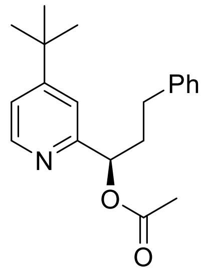

2t (4.5 mg, 16.7 µmol) was dissolved in CH_2Cl_2 (4 mL). Sequentially, triethylamine (15 µL, 110 µmol, 6.6 eq.), DMAP (1 mg, 8.2 µmol, 49 mol%) and acetic anhydride (6 µL, 64 mmol, 3.8 eq.) were added. The mixture was stirred at room temperature for 16 h. The solvent was then concentrated *in* vacuo and the crude mixture was subjected to flash column chromatography eluting with hexane/EtOAc 75:25 to afford desired product **2ta** as a colourless oil (5 mg, 96% yield, 84% ee).

^{1}**H NMR** (400 MHz, $CDCl_3$) δ 8.48 (d, *J* = 5.2 Hz, 1H), 7.28 – 7.23 (m, 3H), 7.19 – 7.13 (m, 4H), 5.79 (dd, *J* = 8.1, 5.5 Hz, 1H), 2.74 – 2.59 (m, 2H), 2.36 – 2.27 (m, 1H), 2.27 – 2.19 (m, 1H), 2.11 (s, 3H), 1.30 (s, 9H); ^{13}**C NMR** (101 MHz, $CDCl_3$) δ 170.7, 160.9, 159.0, 149.5, 141.4, 128.5 (4 × C), 126.1, 120.1, 118.4, 76.5, 36.6, 34.9, 32.0, 30.7 (3 × C), 21.3. **HRMS m/z:** $[M+H]^+$ calculated for $[C_{20}H_{26}NO_2]^+$ 312.1958, found: 312.1963. **SFC Analysis:** Chiralpak SC (CO_2/MeOH = 95/05, 2.5 mL min^{-1}, 40 °C, 257 nm) t_R = 4.2 (major), 4.7 (minor) minutes. $[\alpha]_D^{25.0}$ = +6.3 (*c* 0.5, $CHCl_3$).

Reactions of Lepidine

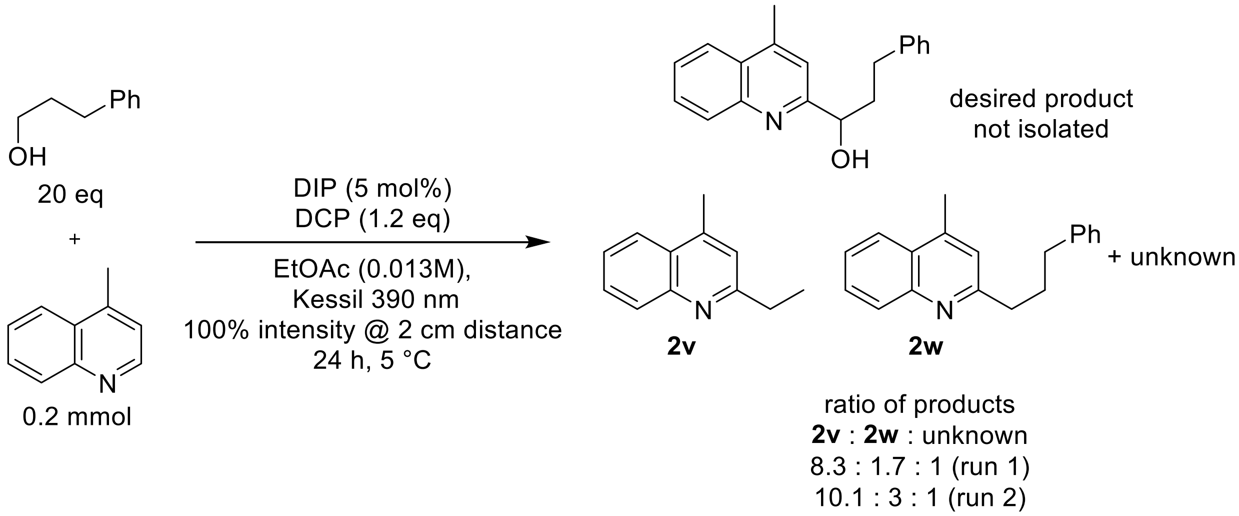

Following General Procedure A, lepidine (28.6 mg, 0.20 mmol), 3-phenyl-1-propanol (545 mg, 4.0 mmol, 20 eq), dicumyl peroxide (64.9 mg, 0.24 mmol, 1.2 eq) and (*R*)-DIP (6.7 mg, 0.01 mmol, 5 mol%) were used. After 24 h, the reaction was quenched with a few drops of triethylamine and concentrated *in vacuo*. Analysis of the ^{1}H NMR spectrum of the crude reaction mixture did not show diagnostic resonances indicating formation of the desired hydroxylalkylated product. The crude residue was dissolved in diethyl ether (5 mL) and shaken with 3 M HCl (5 mL). The two phases were separated, and the aqueous phase was then washed with diethyl ether (1 × 5 mL), neutralised to pH 7 using sat. $NaHCO_3$ solution and extracted with CH_2Cl_2 (3 ×5 mL). The organic phase was dried using Na_2SO_4, filtered and concentrated *in vacuo*. The resultant material was purified *via* flash column chromatography eluting with hexane/EtOAc 100:0 to 0:100. A mixture of products were isolated as a mixture of **2v** and **2w** but also containing a small amount of another unidentified product (4 mg, ca. 12% yield w.r.t mmol lepidine used). In the isolated material the two major products present, **2v** and **2w** were identified by comparison of the resonances in the ^{1}H NMR spectra of the crude residue post acid wash and the isolated material post flash column chromatography to those which are reported in the literature for the respective compounds.[15-16]

Resonances assigned to 2v: ^{1}**H NMR** (500 MHz, $CDCl_3$) δ 8.04 (d, *J* = 8.4 Hz, 1H), 7.95 (d, *J* = 8.3 Hz, 1H), 7.67 (ddt, *J* = 8.3, 6.8, 1.5 Hz, 1H), 7.50 (tt, *J* = 6.9, 1.5 Hz, 1H), 7.16 (s, 1H), 7.16 (d, *J* = 1.1 Hz, 1H), 2.96 (q, *J* = 7.6 Hz, 2H), 2.68 (d, *J* = 1.0 Hz, 3H), 1.39 (t, *J* = 7.7 Hz, 3H). ^{1}H NMR data was consistent with the literature data.[15]

Resonances which could be assigned to 2w: ^{1}**H NMR** (500 MHz, $CDCl_3$) δ 8.08 – 8.05 (m, 1H), 7.13 (d, *J* = 1.1 Hz, 2H), 2.76 – 2.72 (m, 2H), 2.67 (d, *J* = 0.9 Hz, 3H), 2.18 – 2.11 (m, 3H). The remaining

resonances were overlapping with the resonances assigned to **2v** and the unknown compound present in the mixture. ^{1}H NMR data was consistent with the literature data.[16]

***tert*-Butyl (*R*)-(6-(1-hydroxy-3-phenylpropyl)pyridin-3-yl)carbamate (2x) and *tert*-butyl (2-(1-hydroxy-3-phenylpropyl)pyridin-3-yl)carbamate (2xa)**

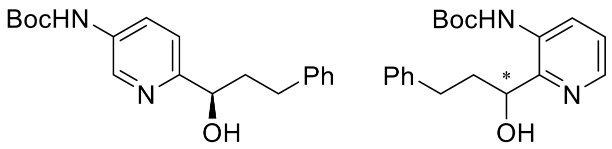

2x **2xa**

r.r. = 1.6 : 1

Following General Procedure A, 3-(Boc-amino)pyridine (77.7 mg, 0.4 mmol), 3-phenyl-1-propanol (1.09g, 8.0 mmol, 20 eq), dicumyl peroxide (129.8 mg, 0.48 mmol, 1.2 eq) and (*R*)-DIP (13.4 mg, 0.02 mmol, 5 mol%) were used. Regioisomeric ratio was determined from the crude ^{1}H NMR (1.6 (**2x**) : 1 (**2xa**)). The crude material was purified *via* an initial flash column chromatography eluting with 1% triethylamine in hexane/EtOAc 100:0 to 50:50, followed by subsequent flash column chromatography eluting with 1% triethylamine in CH_2Cl_2/Et_2O 100:0 to 99:1. A final purification by preparatory TLC to separate the regioisomers eluting with hexane/ethyl acetate 20:80 afforded the desired products **2xa** (9 mg, 7% yield, 19% ee) and **2x** (14mg, 11% yield, 66% ee).

***tert*-Butyl (*R*)-(6-(1-hydroxy-3-phenylpropyl)pyridin-3-yl)carbamate (2xa):**

^{1}H NMR (500 MHz, $CDCl_3$) δ 8.29 (br. d, J = 8.6 Hz, 1H), 8.22 (dd, J = 4.7, 1.4 Hz, 7.31 – 7.25 (m, 2H), 7.24 – 7.17 (m, 4H), 4.83 (ddd, J = 8.6, 5.5, 4.4 Hz, 1H), 3.66 (d, J = 8.6 Hz, 1H), 2.86 – 2.74 (m, 2H), 2.18 – 2.10 (m, 1H), 2.10 – 2.01 (m, 1H), 1.51 (s, 9H); **^{13}C NMR** (126 MHz, $CDCl_3$) δ 153.2, 150.2, 143.0, 141.8, 133.1, 128.9, 128.8, 125.4, 123.3, 81.4, 72.5, 38.0, 32.0, 28.7; **HRMS m/z:** $[M+H]^+$ calculated for $[C_{19}H_{24}N_2O_3]^+$ 329.1861, found: 329.1861. **SFC Analysis:** CHIRAL ART IG (CO_2/MeOH = 85/15, 2.5 mL min^{-1}, 40 °C) t_R = 2.7 (minor), 3.0 (major) minutes. **$[\alpha]_D^{25.0}$** = –5.3 (c 0.31, $CHCl_3$).

hydroxy-3-phenylpropyl)pyridin-3-yl)carbamate (2x):

^{1}H NMR (500 MHz, $CDCl_3$) δ 8.38 (d, J = 2.3 Hz, 1H), 7.94 (br. s, 1H), 7.29 – 7.24 (m, 2H), 7.22 – 7.15 (m, 4H), 6.51 (br. s, 1H), 4.73 (ddd, J = 8.3, 4.9, 4.5 Hz, 1H), 3.97 (d, J = 5.5 Hz, 1H), 2.83 – 2.68 (m, 2H), 2.11 (dddd, J = 13.7, 9.8, 6.8, 4.0 Hz, 1H), 1.97 (dddd, J = 13.7, 9.7, 8.0, 5.7 Hz, 1H), 1.52 (s, 9H); **^{13}C NMR** (126 MHz, $CDCl_3$) δ 156.6, 153.0, 142.4, 138.9, 134.4, 128.8, 128.7, 127.1, 126.1, 120.6, 81.7, 72.1 (2 x C), 40.6, 31.9, 28.6 (2 x C); **HRMS m/z:** $[M+H]^+$ calculated for $[C_{19}H_{24}N_2O_3]^+$ 329.1861, found: 329.1862. ***SFC Analysis:*** **SFC Analysis:** CHIRAL ART IE (CO_2/MeOH = 70/30, 2.5 mL min^{-1}, 40 °C) t_R = 3.5 (major), 4.6 (minor) minutes. **$[\alpha]_D^{25.0}$** = 0.83 (c 0.73, $CHCl_3$).

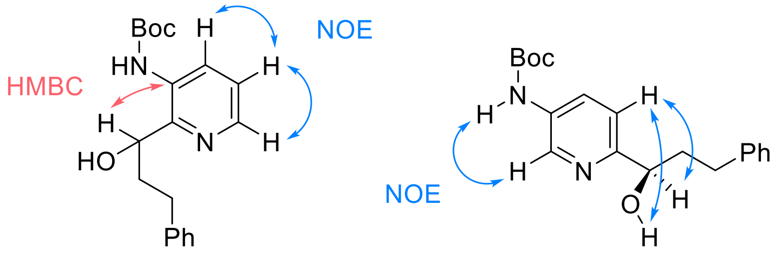

Alcohol Scope – Synthesis of Products

(*R*)-3-(2-Bromophenyl)-1-(6-pentylpyridin-2-yl)propan-1-ol (6a)

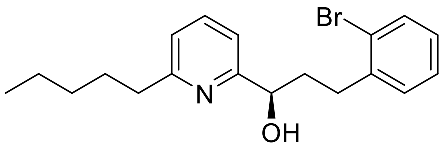

Following general procedure A, 2-pentylpyridine (29.8 mg, 0.2 mmol), 3-(2-bromophenyl)propan-1-ol (860 mg, 4.0 mmol, 20 eq), dicumyl peroxide (64.9 mg, 0.24 mmol, 1.2 eq) and (*R*)-DIP (6.7 mg, (0.01 mmol, 5 mol %) were used. The crude material was purified *via* initial flash column chromatography eluting with Petroleum Ether 40-60/EtOAc (9:1) followed by subsequent flash column chromatography eluting with CH_2Cl_2/MeOH (1:0 to 99:1) to afford the title product as a white solid (32.4 mg, 0.09 mmol, 45 % yield, 84 % *ee*).

^{1}H NMR (400 MHz; $CDCl_3$) δ 7.59 (t, *J* = 7.7 Hz, 1H), 7.53 (dd, *J* = 8.0, 1.1 Hz, 1H), 7.28 (dd, *J* = 7.6, 1.8 Hz, 1H), 7.23 (td, *J* = 7.4, 1.2 Hz, 1H), 7.08 – 7.03 (m, 3H), 5.06 – 4.72 (br s, 1H), 4.79 (dd, *J* = 7.8, 3.4 Hz, 1H), 2.96 – 2.84 (m, 2H), 2.81 (t, *J* = 7.7 Hz, 1H), 2.17 – 2.14 (m, 1H), 1.99 – 1.91 (m, 1H), 1.77 (q, *J* = 7.6 Hz, 2H), 1.40 – 1.34 (m, 4H), 0.92 (t, *J* = 7.0 Hz, 3H). **^{13}C NMR (101 MHz; $CDCl_3$) δ** 160.9, 160.4, 141.5, 136.9, 132.8, 130.6, 127.5, 127.4, 124.4, 121.2, 117.3, 71.4, 38.5, 37.9, 31.9, 31.5, 29.2, 22.5, 14.0. **HRMS:** m/z: $[M+H]^+$ calc'd for $[C_{19}H_{25}NO^{79}Br]^+$ expected 361.1041, observed 361.1046. **SFC Analysis:** CHIRAL ART SC (CO_2/MeOH = 97/3, 2.5 ml min^{-1}, 40 °C, 263 nm) t_R = 12.1 (major), 14.3 (minor) minutes. **$[\alpha]_D^{25.0}$** = -22.5 (c 1.0 $CHCl_3$).

(*R*)-3-(2-methoxyphenyl)-1-(6-pentylpyridin-2-yl)propan-1-ol (6b)

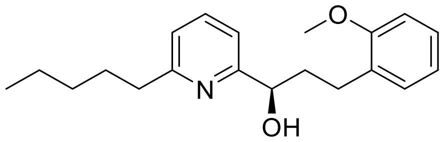

Following general procedure A, 2-pentylpyridine (29.8 mg, 0.2 mmol), 3-(2-methoxyphenyl)propan-1-ol (664 mg, 4.0 mmol, 20 eq), dicumyl peroxide (64.9 mg, 0.24 mmol, 1.2 eq) and (*R*)-DIP (6.7 mg, (0.01 mmol, 5 mol %) were used. The crude material was purified *via* initial flash column chromatography eluting with Petroleum Ether 40-60/EtOAc (9:1) followed by subsequent flash column chromatography eluting with CH_2Cl_2/MeOH (1:0 to 99:1) to afford the title product as a white solid (24.0 mg, 0.076 mmol, 38 % yield, 81 % *ee*).

^{1}H NMR (400 MHz; $CDCl_3$) δ 7.58 (t, *J* = 7.7 Hz, 1H), 7.21 – 7.17 (m, 2H), 7.08 (d, *J* = 7.8 Hz, 1H), 7.04 (d, *J* = 7.6 Hz, 1H), 6.89 (td, *J* = 7.4, 0.8 Hz, 1H), 6.86 (d, *J* = 8.5 Hz, 1H), 4.80 (s, 1H), 4.76 (dd, *J* = 8.1, 3.8 Hz, 1H), 3.84 (s, 3H), 2.83 – 2.79 (m, 4H), 2.18 – 2.11 (m, 1H), 1.97 – 1.91 (m, 1H), 1.76 (quin, *J* = 7.6 Hz, 2H), 1.40 – 1.32 (m, 4H), 0.92 (t, *J* = 6.9 Hz, 3H). **^{13}C NMR (101 MHz; $CDCl_3$) δ** 161.0, 160.9, 157.5, 136.8, 130.6, 130.1, 127.0, 121.0, 120.5, 117.3, 110.2, 71.9, 55.3, 38.6, 38.0, 31.5, 29.3, 26.2, 22.5, 14.0. **HRMS:** m/z: $[M+H]^+$ calc'd for $[C_{20}H_{28}NO_2]^+$ expected 314.2115, observed 314.2119. **SFC Analysis:** CHIRAL ART SC (CO_2/MeOH = 93/7, 2.5 ml min^{-1}, 40 °C, 263 nm) t_R = 7.1 (major), 7.9 (minor) minutes. **$[\alpha]_D^{25.0}$** = -12.6 (c 1.0 $CHCl_3$).

(*R*)-3-(4-Bromophenyl)-1-(6-pentylpyridin-2-yl)propan-1-ol (6c)

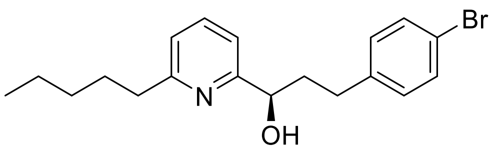

Following general procedure A, 2-pentylpyridine (29.8 mg, 0.2 mmol), 3-(4-bromophenyl)propan-1-ol (860 mg, 4.0 mmol, 20 eq), dicumyl peroxide (64.9 mg, 0.24 mmol, 1.2 eq) and (*R*)-DIP (6.7 mg, (0.01 mmol, 5 mol %) were used. The crude material was purified *via* initial flash column chromatography eluting with Petroleum Ether 40-60/EtOAc (9:1) followed by subsequent flash column chromatography eluting with CH_2Cl_2/MeOH (1:0 to 99:1) to afford the title product as a white solid (36.3 mg, 0.10 mmol, 50 % yield, 81 % *ee*).

^{1}H NMR (400 MHz; $CDCl_3$) δ 7.59 (t, *J* = 7.7 Hz, 1H), 7.40 (d, *J* = 8.4 Hz, 2H), 7.10 (d, *J* = 8.4 Hz, 2H), 7.06 (d, *J* = 7.5 Hz, 1H), 7.02 (d, *J* = 7.7 Hz, 1H), 4.84 (br s, 1H), 4.74 (dd, *J* = 8.1, 3.8 Hz), 2.86 – 2.82 (m, 4H), 2.16 – 2.08 (m, 1H), 1.97 – 1.89 (m, 1H), 1.76 (qn, *J* = 7.4 Hz, 2H), 1.41 – 1.32 (m, 4H), 0.92 (t, *J* = 7.1 Hz). **^{13}C NMR (101 MHz; $CDCl_3$) δ** 161.0, 160.5, 141.2, 137.0, 131.4, 130.3, 121.2, 119.5, 117.2, 71.2, 40.2, 37.9, 31.5, 30.9, 29.2, 22.5, 14.0. **HRMS:** m/z: $[M+H]^+$ calc'd for $[C_{19}H_{25}NO^{79}Br]^+$ expected 361.1041, observed 361.1048. **SFC Analysis:** CHIRAL ART SC (CO_2/MeOH = 97/3, 2.5 ml min^{-1}, 40 °C, 263 nm) t_R = 11.3 (major), 12.4 (minor) minutes. **$[\alpha]_D^{25.0}$ =** -24.0 (c 1.0 $CHCl_3$).

(*R*)-3-(2-Fluorophenyl)-1-(6-pentylpyridin-2-yl)propan-1-ol (6d)

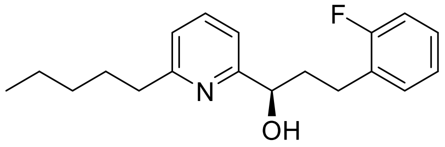

Following general procedure A, 2-pentylpyridine (29.8 mg, 0.2 mmol), 3-(2-fluorophenyl)propan-1-ol (616 mg, 4.0 mmol, 20 eq), dicumyl peroxide (64.9 mg, 0.24 mmol, 1.2 eq) and (*R*)-DIP (6.7 mg, (0.01 mmol, 5 mol %) were used. The crude material was purified *via* initial flash column chromatography eluting with Petroleum Ether 40-60/EtOAc (9:1) followed by subsequent flash column chromatography eluting with CH_2Cl_2/MeOH (1:0 to 99:1) to afford the title product as a white solid (33.7 mg, 0.11 mmol, 56 %, 86 % *ee*).

^{1}H NMR (400 MHz; $CDCl_3$) δ 7.58 (t, *J* = 7.7 Hz, 1H), 7.24 (td, *J* = 7.6, 1.5 Hz, 1H), 7.15 – 7.19 (m, 1H), 7.04 – 7.07 (m, 3H), 7.01 (t, *J* = 9.6 Hz, XH?), 4.90 (s, 1H), 4.77 (dd, *J* = 8.0, 3.7 Hz, 1H), 2.79 – 2.86 (m, 4H), 2.13 – 2.20 (m, 1H), 1.92 – 1.99 (m, 1H), 1.76 (quin, *J* = 7.5 Hz, 2H), 1.33 – 1.42 (m, 4H), 0.92 (t, *J* = 6.9 Hz, 3H). **^{13}C NMR (101 MHz; $CDCl_3$) δ** 161.2 (d, *J* = 244.7 Hz), 161.0, 160.5, 136.9, 130.8 (d, *J* = 5.2 Hz), 129.0 (d, *J* = 15.9 Hz), 127.5 (d, *J* = 8.1 Hz), 123.9 (d, *J* = 3.5 Hz), 121.2, 117.3, 115.2 (d, *J* = 22.1 Hz), 71.4, 38.9, 38.0, 31.5, 29.2, 25.0 (d, *J* = 2.4 Hz), 22.5, 14.0. **^{19}F NMR (376 MHz; $CDCl_3$) δ** -118.8. **HRMS:** m/z: $[M+H]^+$ calc'd for $[C_{19}H_{25}NOF]^+$ expected 302.1915, observed 302.1922. **SFC Analysis:** CHIRAL ART SC (CO_2/MeOH = 97/3, 2.5 ml min^{-1}, 40 °C, 200 nm) t_R = 5.7 (minor), 6.2 (major) minutes. **$[\alpha]_D^{25.0}$ =** -21.0 (c 1.0 $CHCl_3$).

(*R*)-2-(2-fluorophenyl)-1-(6-pentylpyridin-2-yl)ethan-1-ol (6e)

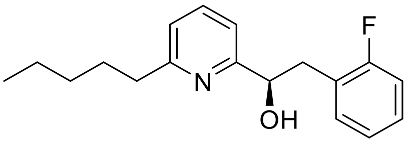

Following general procedure A, 2-pentylpyridine (29.8 mg, 0.2 mmol), 2-(2-fluorophenyl)ethanol (560 mg, 4.0 mmol, 20 eq), dicumyl peroxide (64.9 mg, 0.24 mmol, 1.2 eq) and (*R*)-DIP (6.7 mg, (0.01 mmol, 5 mol %) were used. The crude material was purified *via* initial flash column chromatography eluting with Petroleum Ether 40-60/EtOAc (9:1) followed by subsequent flash column chromatography eluting with CH_2Cl_2/MeOH (1:0 to 99:1) to afford the title product as a white solid (22.8 mg, 0.08 mmol, 40 %, 88 % *ee*).

^{1}H NMR (400 MHz; $CDCl_3$) δ 7.54 (t, *J* = 7.7 Hz, 1H), 7.23 – 7.18 (m, 2H), 7.07 – 6.98 (m, 3H), 6.93 (d, *J* = 7.7 Hz, 1H), 4.99 (t, *J* = 6.1 Hz, 1H), 4.82 – 4.65 (br s, 1H), 3.15 (dd, *J* = 13.7, 5.0 Hz, 1H), 3.02 (dd, *J* = 13.7, 7.5 Hz, 1H), 2.78 (t, *J* = 7.7 Hz, 2H), 1.72 (quin, *J* = 7.3 Hz, 2H), 1.39 – 1.28 (m, 4H), 0.92 (t, *J* = 6.8 Hz, 3H). **^{13}C NMR (101 MHz; $CDCl_3$) δ** 161.3 (d, *J* = 244.9 Hz), 161.0, 159.8, 136.7, 132.1 (d, *J* = 4.9 Hz), 128.1 (d, *J* = 8.1 Hz), 125.1 (d, *J* = 15.6 Hz), 123.8 (d, *J* = 3.6 Hz), 121.4, 117.7, 115.1 (d, *J* = 22.3 Hz), 72.1, 38.5, 37.9, 31.5, 29.2, 22.5, 14.0. **^{19}F NMR (376 MHz; $CDCl_3$) δ** -118.2. **HRMS:** m/z: $[M+H]^+$ calc'd for $[C_{18}H_{23}NOF]^+$ expected 288.1758, observed 288.1761. **SFC Analysis:** CHIRALCEL IH (CO_2/MeOH = 98.5/1.5, 2.5 ml min^{-1}, 40 °C, 263 nm) t_R = 5.6 (major), 6.3 (minor) minutes. **$[\alpha]_D^{25.0}$ =** -8.5 (c 1.0 $CHCl_3$).

(*R*)-2-(4-Bromophenyl)-1-(6-pentylpyridin-2-yl)ethan-1-ol (6f)

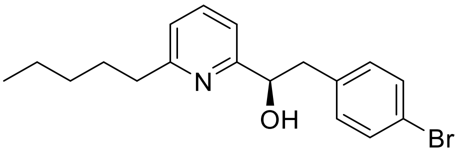

Following general procedure A, 2-pentylpyridine (29.8 mg, 0.2 mmol), 2-(4-bromophenyl)ethanol (804 mg, 4.0 mmol, 20 eq), dicumyl peroxide (64.9 mg, 0.24 mmol, 1.2 eq) and (*R*)-DIP (6.7 mg, (0.01 mmol, 5 mol %) were used. The crude material was purified *via* initial flash column chromatography eluting with Petroleum Ether 40-60/EtOAc (9:1) followed by subsequent flash column chromatography eluting with CH_2Cl_2/MeOH (1:0 to 99:1) to afford the title product as a white solid (30.0 mg, 0.086 mmol, 43 % yield, 83% *ee*).

^{1}H NMR (400 MHz; $CDCl_3$) δ 7.56 (t, *J* = 7.7 Hz, 1H), 7.37 (d, *J* = 8.4 Hz, 2H), 7.05 (d, *J* = 7.4 Hz, 1H), 7.01 (d, *J* = 8.4 Hz, 2H), 6.92 (d, *J* = 7.7 Hz, 1H), 4.94 (t, *J* = 6.0 Hz, 1H), 4.64 (s, 1H), 3.07 (dd, *J* = 13.7, 5.2 Hz, 1H), 2.97 (dd, *J* = 13.7, 7.0 Hz, 1H), 2.76 (t, *J* = 7.7 Hz, 2H), 1.69 (quin, *J* = 7.5 Hz, 2H), 1.40 – 1.27 (m, 4H), 0.92 (t, *J* = 7.1 Hz, 3H). **^{13}C NMR (101 MHz; $CDCl_3$) δ** 161.2, 159.6, 136.8, 136.8, 131.4, 131.2, 121.4, 120.2, 117.7, 72.9, 44.5, 37.9, 31.5, 29.2, 25.1, 25.0, 22.5, 14.1. **HRMS:** m/z: $[M+H]^+$ calc'd for $[C_{18}H_{23}NO^{79}Br]^+$ expected 347.0885, observed 347.0892. **SFC Analysis:** CHIRAL ART SC (CO_2/MeOH = 95/5, 2.5 ml min^{-1}, 40 °C, 263 nm); t_R = 5.7 (minor), 6.0 (major) minutes.**$[\alpha]_D^{25.0}$ =** -12.1 (c 1.0 $CHCl_3$).

(*R*)-2-(2-tolyl)-1-(6-pentylpyridin-2-yl)ethan-1-ol (6g)

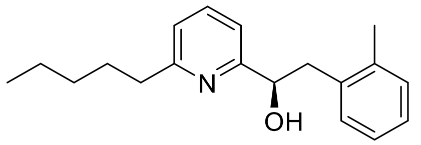

Following general procedure A, 2-pentylpyridine (29.8 mg, 0.2 mmol), 2-tolylethanol (544 mg, 4.0 mmol, 20 eq), dicumyl peroxide (64.9 mg, 0.24 mmol, 1.2 eq) and (*R*)-DIP (6.7 mg, (0.01 mmol, 5 mol %) were used. The crude material was purified *via* initial flash column chromatography eluting with Petroleum Ether 40-60/EtOAc (9:1) followed by subsequent flash column chromatography eluting with CH_2Cl_2/MeOH (1:0 to 98:2) to afford the title product as a white solid (22.9 mg, 0.08 mmol, 40 % yield, 85 % *ee*).

^{1}H NMR (400 MHz; $CDCl_3$) δ 7.49 (t, *J* = 7.6 Hz, 1H), 7.15 – 7.12 (m, 4H), 7.05 (d, *J* = 7.6 Hz, 1H), 6.73 (d, *J* = 7.6 Hz, 1H), 4.92 (t, *J* = 6.8 Hz, 1H), 4.65 – 4.55 (br s, 1H), 3.09 (dd, *J* = 13.8, 7.3 Hz, 1H), 3.04 (dd, *J* = 13.8, 6.3 Hz, 1H), 2.80 (t, *J* = 7.7 Hz, 2H), 2.21 (s, 3H), 1.75 (quin, *J* = 7.5 Hz, 2H), 1.40 – 1.33 (m, 4H), 0.93 (t, *J* = 7.1 Hz, 3H). **^{13}C NMR (101 MHz; $CDCl_3$) δ** 161.3, 160.2, 136.8, 136.5, 136.3, 130.4, 130.2, 126.5, 125.8, 121.4, 117.9, 73.0, 42.7, 38.0, 31.5, 29.3, 22.5, 19.5, 14.0. **HRMS:** m/z: $[M+H]^+$ calc'd for $[C_{19}H_{26}NO]^+$ expected 283.1936, observed 283.1943. **SFC Analysis:** CHIRAL ART SJ (CO_2/MeOH = 99/1, 2.5 ml min^{-1}, 40 °C, 263 nm) t_R = 6.1 (major), 6.8 (minor) minutes. **$[\alpha]_D^{25.0}$ =** -17.2 (c 1.0 $CHCl_3$).

(*R*)-2-(4-Methoxyphenyl)-1-(6-pentylpyridin-2-yl)ethan-1-ol (6h)

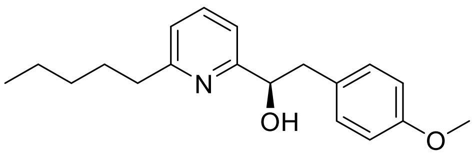

Following general procedure A, 2-pentylpyridine (29.8 mg, 0.2 mmol), 2-(4-methoxyphenyl)ethanol (608 mg, 4.0 mmol, 20 eq), dicumyl peroxide (64.9 mg, 0.24 mmol, 1.2 eq) and (*R*)-DIP (6.7 mg, (0.01 mmol, 5 mol %) were used. The crude material was purified *via* initial flash column chromatography eluting with Petroleum Ether 40-60/EtOAc (9:1) followed by subsequent flash column chromatography eluting with CH_2Cl_2/MeOH (1:0 to 99:1) to afford the title product as a white solid (32.4 mg, 0.11 mmol, 54 %, 84 % *ee*).

^{1}H NMR (400 MHz; $CDCl_3$) δ 7.54 (t, *J* = 7.7 Hz, 1H), 7.07 (d, *J* = 8.6 Hz, 2H), 7.05 (d, *J* = 7.8 Hz, 1H), 6.90 (d, *J* = 7.7 Hz, 1H), 6.82 (d, *J* = 8.6 Hz, 2H), 4.93 (t, *J* = 6.1 Hz, 1H), 4.54 (s, 1H), 3.80 (s, 3H), 3.05 (dd, *J* = 13.8, 5.6 Hz, 1H), 3.00 (dd, *J* = 13.7, 7.1 Hz), 2.79 (t, *J* = 7.7 Hz, 2H), 1.72 (qn, *J* = 7.5 Hz, 2H), 1.40 – 1.30 (m, 4H), 0.92 (t, *J* = 7.0 Hz, 3H). **^{13}C NMR (101 MHz; $CDCl_3$) δ** 161.1, 160.1, 158.2, 136.7, 130.5, 129.9, 121.3, 117.9, 113.7, 73.5, 55.2, 44.3, 37.9, 31.5, 29.3, 22.5, 14.0. **HRMS:** m/z: $[M+H]^+$ calc'd for $[C_{19}H_{26}NO_2]^+$ expected 300.1958, observed 300.1956. **SFC Analysis:** CHIRAL ART SJ (CO_2/MeOH = 97/3, 2.5 ml min^{-1}, 40 °C, 263 nm) t_R = 5.51 (minor), 5.9 (major) minutes. **$[\alpha]_D^{25.0}$ =** -2.4 (c 1.0 $CHCl_3$).

(*R*)-1-(6-pentylpyridin-2-yl)-4-phenylbutan-1-ol (6i)

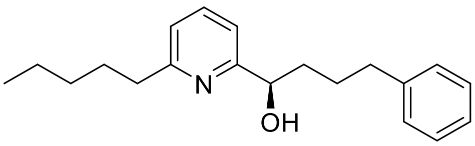

Following general procedure A, 2-pentylpyridine (29.8 mg, 0.2 mmol), 4-phenylpropan-1-ol (600 mg, 4.0 mmol, 20 eq), dicumyl peroxide (64.9 mg, 0.24 mmol, 1.2 eq) and (*R*)-DIP (6.7 mg, (0.01 mmol, 5 mol %) were used. The crude material was purified *via* initial flash column chromatography eluting with Petroleum Ether 40-60/EtOAc (9:1) followed by subsequent flash column chromatography eluting with CH_2Cl_2/MeOH (1:0 to 99:1) to afford the title product as a white solid (27.1 mg, 0.09 mmol, 46 %).

^{1}H NMR (400 MHz; $CDCl_3$) δ 7.59 (t, *J* = 7.5 Hz, 1H), 7.30 – 7.27 (m, 2H), 7.21 – 7.18 (m, 3H), 7.05 (d, *J* = 7.5 Hz), 7.02 (d, *J* = 7.6 Hz, 1H), 4.75 (br s, 2H), 2.80 (t, *J* = 7.6 Hz, 2H), 2.69 – 2.65 (m, 2H), 1.90 – 1.71 (m, 6H), 1.42 – 1.34 (m, 4H), 0.92 – 0.88 (m, 3H). **^{13}C NMR (101 MHz; $CDCl_3$) δ** 160.9, 160.8, 142.4, 136.9, 128.4, 128.3, 125.7, 121.1, 117.3, 71.9, 38.1, 37.9, 35.8, 31.5, 29.2, 26.9, 22.5, 14.0. **HRMS:** m/z: $[M+H]^+$ calc'd for $[C_{20}H_{28}NO]^+$ expected 297.2093, observed 297.2099. **$[\alpha]_D^{25.0}$ =** -8.5 (c 1.0 $CHCl_3$).

(*R*)-1-(6-pentylpyridin-2-yl)-4-phenylbutan-1-yl acetate (6ia)

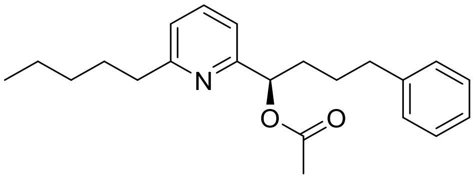

(*R*)-1-(6-pentylpyridin-2-yl)-4-phenylbutan-1-ol (0.3 mg, 0.001 mmol) and triethylamine (0.015 ml ,0.1 mmol) were dissolved in dichloromethane (2 ml) before trifluoroacetic anhydride (0.01 ml, 0.1 mmol) was added and the mixture stirred at room temperature for one hour. The reaction was quenched by addition of water (2 ml) and the layers separated. The aqueous layer was washed with dichloromethane (3 x 5 ml) and the combined organic layers dried over magnesium sulphate and concentrated. Purification by flash column chromatography eluting with Petroleum Ether 40-60/EtOAc (9:1) gave the title compound as a colourless oil (0.3 mg, 0.001 mmol, 95 %, 88 % *ee*)

^{1}H NMR (400 MHz; $CDCl_3$) δ 7.56 (t, *J* = 7.7 Hz, 1H), 7.30 – 7.26 (m, 2H), 7.21 – 7.16 (m, 3H), 7.09 (d, *J* = 7.6 Hz, 1H), 7.05 (d, *J* = 7.7 Hz, 1H), 5.83 (t, *J* = 6.7 Hz, 1H), 2.79 (t, *J* = 7.8 Hz, 2H), 2.65 (t, *J* = 7.7 Hz, 2H), 2.14 (s, 3H), 2.03 – 1.98 (m, 2H), 1.81 – 1.62 (m, 4H), 1.37 – 1.31 (m, 4H), 0.91 (t, *J* = 6.9 Hz, 3H). **^{13}C NMR (101 MHz; $CDCl_3$) δ** 170.4, 162.2, 158.9, 142.1, 136.6, 128.4, 128.3, 125.8, 121.5, 117.7, 38.3, 35.6, 34.6, 31.6, 29.6, 27.2, 22.6, 21.2, 14.0. **HRMS:** m/z: $[M+H]^+$ calc'd for $[C_{22}H_{30}NO_2]^+$ expected 340.2271, observed 340.2298. **SFC Analysis:** CHIRALCEL IH (CO_2/MeOH = 98/2, 2.5 ml min, 40 °C, 200 nm) t_R = 3.8 (major), 4.3 (minor) minutes.

(R)-1-(6-pentylpyridin-2-yl)butan-1-ol (6j)

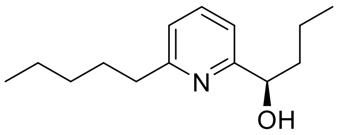

Following general procedure A, 2-pentylpyridine (29.8 mg, 0.2 mmol), butan-1-ol (296 mg, 4.0 mmol, 20 eq), dicumyl peroxide (64.9 mg, 0.24 mmol, 1.2 eq) and (*R*)-DIP (6.7 mg, (0.01 mmol, 5 mol %) were used. The crude material was purified *via* initial flash column chromatography eluting with Petroleum Ether 40-60/EtOAc (9:1) followed by subsequent flash column chromatography eluting with CH_2Cl_2/MeOH (1:0 to 99:1) to afford the title product as a white solid (20.8 mg, 0.094 mmol, 47 %, 84 % *ee*).

^{1}H NMR (400 MHz; $CDCl_3$) δ 7.57 (t, *J* = 7.7 Hz, 1H), 7.02 (d, *J* = 7.7 Hz, 2H), 4.71 (dd, *J* = 7.7, 4.2 Hz, 1H), 4.81 – 4.55 (br s, 1H), 2.78 (t, *J* = 7.7 Hz, 2H), 1.81 – 1.59 (m, 4H), 1.49 – 1.40 (m, 2H), 1.37 – 1.29 (m, 4H), 0.93 (t, *J* = 7.4 Hz, 3H), 0.89 (t, *J* = 7.0 Hz, 3H). **^{13}C NMR (101 MHz; $CDCl_3$) δ** 161.1, 160.8, 136.9, 121.0, 117.3, 71.9, 40.9, 37.9, 31.5, 29.2, 22.5, 18.2, 14.1, 14.0. **HRMS:** m/z: $[M+H]^+$ calc'd for $[C_{14}H_{24}NO]^+$ expected 222.1852, observed 222.1852. **SFC Analysis:** CHIRALCEL IG (CO_2/MeOH = 95/5, 2.5 ml min^{-1}, 40 °C, 263 nm) t_R = 4.3 (major), 4.9 (minor) minutes. **$[\alpha]_D^{25.0}$ =** -1.5 (c 1.0 $CHCl_3$).

(*R*)-3-cyclohexyl-1-(6-pentylpyridin-2-yl)propan-1-ol (6k)

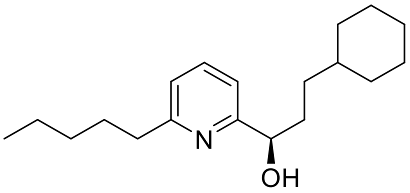

Following general procedure A, 2-pentylpyridine (29.8 mg, 0.2 mmol), 3-cyclohexylpropan-1-ol (568 mg, 4.0 mmol, 20 eq), dicumyl peroxide (64.9 mg, 0.24 mmol, 1.2 eq) and (*R*)-DIP (6.7 mg, (0.01 mmol, 5 mol %) were used. The crude material was purified *via* initial flash column chromatography eluting with Petroleum Ether 40-60/EtOAc (9:1) followed by subsequent flash column chromatography eluting with CH_2Cl_2/MeOH (1:0 to 99:1) to afford the title product as a white solid (23.5 mg, 0.08 mmol, 41 %, 85 % *ee*).

^{1}H NMR (400 MHz; $CDCl_3$) δ 7.60 (t, *J* = 7.7 Hz, 1H), 7.05 (d, *J* = 7.7 Hz, 2H), 4.71 (dd, *J* = 7.6, 4.3 Hz, 1H), 4.70 (br s, 1H), 2.81 (t, *J* = 7.7 Hz, 2H), 1.87 – 1.62 (m, 9H), 1.42 – 1.07 (m, 10H), 0.92 (t, *J* = 6.9 Hz, 3H), 0.83 – 0.91 (m, 2H). **^{13}C NMR (101 MHz; $CDCl_3$) δ** 160.2, 160.8, 137.0, 121.1, 117.4, 72.4, 37.7, 36.0, 33.5, 33.2, 32.8, 31.5, 29.2, 26.8, 26.4, 26.4, 22.5, 14.0. **HRMS:** m/z: $[M+H]^+$ calc'd for $[C_{19}H_{32}NO]^+$ expected 290.2478, observed 290.2489. **SFC Analysis:** CHIRAL ART SC (CO_2/MeOH = 98/2, 2.5 ml min^{-1}, 40 °C, 263 nm) t_R = 9.1 (major), 11.5 (minor) minutes. **$[\alpha]_D^{25.0}$ =** -9.1 (c 1.0 $CHCl_3$).

(*R*)-cyclobutyl(6-pentylpyridin-2-yl)methanol (6l)

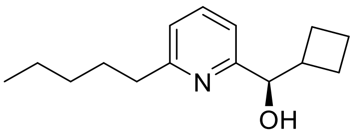

Following general procedure A, 2-pentylpyridine (29.8 mg, 0.2 mmol), cyclobutylmethanol (344 mg, 4.0 mmol, 20 eq), dicumyl peroxide (64.9 mg, 0.24 mmol, 1.2 eq) and (*R*)-DIP (6.7 mg, (0.01 mmol, 5 mol %) were used. The crude material was purified *via* initial flash column chromatography eluting with Petroleum Ether 40-60/EtOAc (9:1) followed by subsequent flash column chromatography eluting with CH_2Cl_2/MeOH (1:0 to 99:1) to afford the title product as a white solid (20.4 mg, 0.09 mmol, 44 %, 88 % *ee*).

^{1}H NMR (400 MHz; $CDCl_3$) δ 7.55 (t, *J* = 7.7 Hz, 1H,), 7.03 (d, *J* = 7.6 Hz, 1H), 6.99 (d, *J* = 7.7 Hz, 1H), 4.75 (s, 1H), 4.60 (d, *J* = 6.3 Hz, 1H), 2.78 (t, *J* = 7.7 Hz, 2H), 2.61 (sex, *J* = 7.9 Hz, 1H), 2.16 – 2.08 (m, 1H), 2.05 – 1.99 (m, 1H), 1.97 – 1.78 (m, 4H), 1.74 (quin, *J* = 7.2 Hz, 2H), 1.39 – 1.31 (m, 4H), 0.91 (t, *J* = 7.0 Hz, 3H). **^{13}C NMR (101 MHz; $CDCl_3$) δ** 160.9, 159.7, 136.6, 121.1, 117.5, 74.8, 42.3, 37.9, 31.5, 29.2, 24.3, 23.1, 22.5, 18.1, 14.0. **HRMS:** m/z: $[M+H]^+$ calc'd for $[C_{15}H_{24}NO]^+$ expected 234.1852, observed 234.1856. **SFC Analysis:** CHIRAL ART SC (CO_2/MeOH = 98/2, 2.5 ml min^{-1}, 40 °C, 263 nm) t_R = 6.4 (major), 7.2 (minor) minutes. **$[\alpha]_D^{25.0}$ =** -8.0 (c 1.0 $CHCl_3$).

(*R*)-1-(6-pentylpyridin-2-yl)but-3-yn-1-ol (6m)

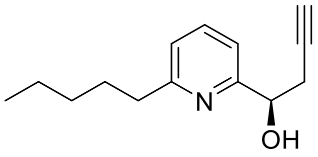

Following general procedure A, 2-pentylpyridine (29.8 mg, 0.2 mmol), but-3-yn-1-ol (280 mg, 4.0 mmol, 20 eq), dicumyl peroxide (64.9 mg, 0.24 mmol, 1.2 eq) and (*R*)-DIP (6.7 mg, (0.01 mmol, 5 mol %) were used. The crude material was purified *via* initial flash column chromatography eluting with Petroleum Ether 40-60/EtOAc (9:1) followed by subsequent flash column chromatography eluting with CH_2Cl_2/MeOH (1:0 to 99:1) to afford the title product as a white solid (14.2 mg, 0.066 mmol, 33 %, 86 % *ee*).

^{1}H NMR (400 MHz; $CDCl_3$) δ 7.63 (t, *J* = 7.7 Hz, 1H), 7.21 (d, *J* = 7.7 Hz, 1H), 7.10 (d, *J* = 7.6 Hz, 1H), 4.88 (t, *J* = 6.0 Hz, 1H), 2.80 (t, *J* = 7.7 Hz, 2H), 2.70 (quin, *J* = 6.2 Hz, 2H), 2.03 (t, *J* = 2.5 Hz, 1H), 1.76 (quin, *J* = 7.5 Hz, 2H), 1.40 – 1.33 (m, 4H), 0.92 (t, *J* = 6.8 Hz, 3H). **^{13}C NMR (101 MHz; $CDCl_3$) δ** 161.2, 158.7, 136.9, 121.8, 117.7, 103.4, 80.7, 70.6, 70.6, 37.9, 31.5, 29.2, 28.6, 22.5, 14.0. **HRMS:** m/z: $[M+H]^+$ calc'd for $[C_{14}H_{20}NO]^+$ expected 218.1539, observed 218.1546. **SFC Analysis:** CHIRAL ART SC (CO_2/MeOH = 98/2, 2.5 ml min^{-1}, 40 °C, 263 nm) t_R = 4.6 (major), 5.1 (minor) minutes. **$[\alpha]_D^{25.0}$ =** -11.2 (c 1.0 $CHCl_3$).

(*R*)-1-(6-pentylpyridin-2-yl)-4-(trimethylsilyl)but-3-yn-1-ol (6n)

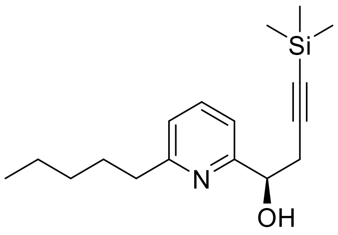

Following general procedure A, 2-pentylpyridine (29.8 mg, 0.2 mmol), 4-(trimethylsilyl)but-3-yn-1-ol (568 mg, 4.0 mmol, 20 eq), dicumyl peroxide (64.9 mg, 0.24 mmol, 1.2 eq) and (*R*)-DIP (6.7 mg, (0.01 mmol, 5 mol %) were used. The crude material was purified *via* initial flash column chromatography eluting with Petroleum Ether 40-60/EtOAc (9:1) followed by subsequent flash column chromatography eluting with CH_2Cl_2/MeOH (1:0 to 99:1) to afford the title product as a white solid (16.4 mg, 0.06 mmol, 28 %, 90 % *ee*).

^{1}H NMR (400 MHz; $CDCl_3$) δ 7.62 (t, *J* = 7.7 Hz, 1H), 7.2 (d, *J* = 7.7 Hz, 1H), 7.11 (d, *J* = 7.6 Hz, 1H), 4.86 (t, *J* = 6.2 Hz, 1H), 5.05 – 4.65 (br s, 1H), 2.81 (t, *J* = 7.8 Hz, 2H), 2.79 (dd, *J* = 16.8, 5.7 Hz, 1H), 2.67 (dd, *J* = 16.8, 6.7 Hz, 1H), 1.76 (quin, *J* = 7.5 Hz, 2H), 1.40 – 1.34 (m, 4H), 0.93 (t, *J* = 6.9 Hz, 3H). **^{13}C NMR (101 MHz; $CDCl_3$) δ** 161.0, 158.8, 136.8, 121.7, 118.1, 103.2, 87.4, 70.7, 37.9, 31.5, 30.1, 29.2, 22.5, 14.0, 0.0. **HRMS:** m/z: $[M+H]^+$ calc'd for $[C_{17}H_{28}NOSi]^+$ expected 290.1935, observed 290.1939. **SFC Analysis:** CHIRAL ART SC (CO_2/MeOH = 99/1, 2.5 ml min^{-1}, 40 °C, 263 nm) t_R = 3.7 (major), 4.3 (minor) minutes. **$[\alpha]_D^{25.0}$ =** -26.4 (c 1.0 $CHCl_3$).

Methyl (R)-4-hydroxy-4-(6-pentylpyridin-2-yl)butanoate (6o)

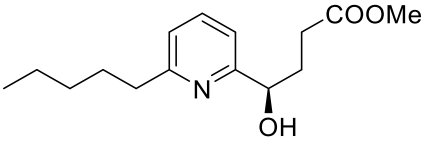

Following general procedure A, 2-pentylpyridine (29.8 mg, 0.2 mmol), methyl 4-hydroxybutanoate (472 mg, 4.0 mmol, 20 eq), dicumyl peroxide (64.9 mg, 0.24 mmol, 1.2 eq) and (*R*)-DIP (6.7 mg, (0.01 mmol, 5 mol %) were used. The crude material was purified *via* initial flash column chromatography eluting with CH_2Cl_2/EtOAc (1:0 to 1:1) followed by subsequent flash column chromatography eluting with Petroleum Ether 40-60/EtOAc (3:1) to afford the title product as a white solid (30.0 mg, 0.11 mmol, 57 % yield, 80 % *ee*).

^{1}H NMR (400 MHz; $CDCl_3$) δ 7.60 (t, *J* = 7.7 Hz, 1H), 7.20 (d, *J* = 7.7 Hz, 1H), 7.04 (d, *J* = 7.60 Hz, 1H), 4.93 (dd, *J* = 8.7, 3.1 Hz, 1H), 4.77 (s, 1H), 3.89 (sept, *J* = 6.0 Hz, 4H), 2.78 (t, *J* = 7.8 Hz, 2H), 2.13 – 2.07 (m, 1H), 1.97 – 1.83 (m, 1H), 1.74 (quin, *J* = 7.5 Hz, 2H), 1.37 – 1.33 (m, 4H), 0.94 (s, 9H), 0.91 (t, *J* = 7.1 Hz, 3H), 0.10 (s, 6H). **^{13}C NMR (101 MHz; $CDCl_3$) δ** 174.3, 161.0, 160.0, 137.0, 121.3, 117.3, 70.9, 51.6, 37.9, 33.5, 31.5, 29.7, 29.2, 22.5, 14.0. **HRMS:** m/z: $[M+H]^+$ calc'd for $[C_{15}H_{24}NO_3]^+$ expected 266.1751, observed 266.1755. **SFC Analysis:** CHIRALPAK SC (CO_2/MeOH = 95/5, 2.5 ml min^{-1}, 40 °C, 263 nm) t_R = 5.2 (minor), 5.9 (major) minutes. **$[\alpha]_D^{25.0}$ =** – 1.5(c 1.0 $CHCl_3$).

(*R*)-3-((tert-butyldimethylsilyl)oxy)-1-(6-pentylpyridin-2-yl)propan-1-ol (6p)

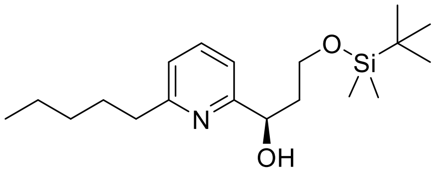

Following general procedure A, 2-pentylpyridine (29.8 mg, 0.2 mmol), 3-(tert-butyldimethylsilyl)oxypropan-1-ol (820 mg, 4.0 mmol, 20 eq), dicumyl peroxide (64.9 mg, 0.24 mmol, 1.2 eq) and (*R*)-DIP (6.7 mg, (0.01 mmol, 5 mol %) were used. The crude material was purified *via* initial flash column chromatography eluting with Petroleum Ether 40-60/EtOAc (9:1) followed by subsequent flash column chromatography eluting with CH_2Cl_2/MeOH (1:0 to 99:1) to afford the title product as a white solid (42.4 mg, 0.12 mmol, 60 % yield, 74 % *ee*).

^{1}H NMR (400 MHz; $CDCl_3$) δ 7.60 (t, *J* = 7.7 Hz, 1H, H_1), 7.20 (d, *J* = 7.7 Hz, 1H, H_2), 7.04 (d, *J* = 7.60 Hz, 1H, H_3), 4.93 (dd, *J* = 8.7, 3.1 Hz, 1H, H_4), 4.77 (s, 1H, H_5), 3.89 (m, 2H, H_6), 2.78 (t, *J* = 7.8 Hz, 2H, H_7), 2.07 – 2.13 (m, 1H, H_8), 1.83 – 1.97 (m, 1H, H_9), 1.74 (quin, *J* = 7.5 Hz, 2H, H_{10}), 1.33 – 1.37 (m, 4H, H_{11}), 0.94 (s, 9H, H_{12}), 0.91 (t, *J* = 7.1 Hz, 3H, H_{13}), 0.10 (s, 6H, H_{14}). **^{13}C NMR (101 MHz; $CDCl_3$) δ** 161.6, 161.1, 136.8, 120.9, 117.1, 71.7, 61.2, 40.6, 38.1, 31.5, 29.4, 25.9, 22.5, 18.2, 14.0, -5.4, -5.5. **HRMS:** m/z: [M + H]$^+$ calc'd for $[C_{19}H_{36}NO_2Si]^+$ expected 338.2510, observed 338.2511. **SFC Analysis:** CHIRALPAK SC (CO_2/MeOH = 98/2, 2.5 ml min^{-1}, 40 °C) t_R = 3.1 (minor), 3.8 (major) minutes. **$[\alpha]_D^{25.0}$ =** +9.3 (c 1.0 $CHCl_3$).

***tert*-butyl (*R*)-(3-hydroxy-3-(6-pentylpyridin-2-yl)propyl)carbamate (6q)**

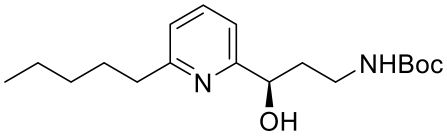

Following general procedure A, 2-pentylpyridine (29.9 mg, 0.2 mmol), 3-(Boc-Amino)-1-propanol (701 mg, 4.0 mmol, 20 eq), dicumyl peroxide (64.9 mg, 0.24 mmol, 1.2 eq) and (*R*)-DIP (6.7 mg, (0.01 mmol, 5 mol %) were used. The crude material was purified *via* initial flash column chromatography eluting with 1% triethylamine in Petroleum Ether 40-60/EtOAc 100:0 to 50:50 followed by subsequent flash column chromatography eluting with 1% triethylamine in hexane/EtOAc (90:10 to 80:20) to afford the title product as a white solid (17 mg, 0.053 mmol, 27% yield, 74% *ee*).

^{1}H NMR (500 MHz, $CDCl_3$) δ 7.58 (t, J = 7.6 Hz, 1H), 7.08 (br. d, J = 7.6 Hz, 1H), 7.03 (d, J = 7.6 Hz, 1H), 5.10 (br. s, 1H), 4.96 (br. s, 1H), 4.78 (dd, J = 8.7, 3.3 Hz, 1H), 3.45 – 3.35 (m, 1H), 3.31 – 3.21 (m, 1H), 2.76 (ap. t, J = 7.8 Hz, 2H), 2.10 – 2.02 (m, 1H), 1.77 – 1.67 (m, 3H), 1.43 (s, 9H), 1.36 – 1.31 (m, 4H), 0.89 (ap. t, J = 7.1 Hz, 3H); **C NMR** (126 MHz, $CDCl_3$) δ 161.3, 160.8, 156.6, 137.4, 121.5, 117.4, 79.4, 71.3, 38.4, 38.3, 38.1, 31.9, 29.6, 28.8, 22.9, 14.4; **HRMS m/z:** [M+H]$^+$ calculated for $[C_{18}H_{30}N_2O_3]^+$ 323.2325 found: 323.2325. **SFC Analysis:** CHIRAL ART IG (CO_2/MeOH = 75/25, 2.5 mL min^{-1}, 40 °C) t_R = 7.8 (minor), 8.9 (major) minutes. **$[\alpha]_D^{25.0}$** = –4.6 (c 0.54, $CHCl_3$).

Mosher Ester Analysis for Determination of Absolute Stereochemistry

To determine the configuration of the newly formed secondary alcohol stereocentre the NMR-based method of Mosher Ester Analysis was used.[17-18] To ensure the configuration deduced from the analysis was correct, Mosher Ester Analysis was first carried out on a commercially available carbinol of known stereochemistry and the same method was then applied to two carbinol products obtained from our HAT-driven Minisci reaction of alcohols and pyridines to determine the unknown stereochemistry.

General Procedure B – Synthesis of Mosher Esters

Mosher Esters were synthesised according to the protocol previously reported by Hoye and co-workers.[18] Sequentially, a stock solution of known concentration of the carbinol (1 eq) in anhydrous CH_2Cl_2, and anhydrous pyridine (3.1 or 6.3 eq, see below for specific example) were transferred to a 4 mL, 15 × 45mm crimp-top vial, containing a stirrer bar, previously sealed with a crimp seal, evacuated and purged with Nitrogen. The corresponding *R*-(-)- or *S*-(+)-α-methoxy-α-(trifluoromethyl)phenylacetyl chloride (MTPA-Cl) was added *via* microsyringe (1.9 or 3.8 eq, see below for specific example). The reaction mixture was stirred at room temperature until determined to be complete by TLC analysis (hexane/EtOAc; 3:2). The reaction mixture was then partitioned between diethyl ether (3 mL) and water (1 mL). The organic and aqueous layers were separated, and the aqueous layer further extracted with diethyl ether (2 × 3 mL). The combined organic layers were dried with Na_2SO_4, filtered, and concentrated *in vacuo*. The resulting residue was purified by flash column chromatography eluting with hexane/EtOAc to afford the desired Mosher (or MTPA) Ester product.

General Procedure C – Mosher Ester Analysis

Mosher Ester analysis was carried out in accordance with the protocol described by Hoye and co-workers.[18] The ^1H NMR spectra for the two diastereomeric MTPA esters were recorded, as well as 2D COSY and TOCSY NMR spectra. Where possible, with the aid of the 2D NMR spectra, the proton resonances - deemed to be relevant to the analysis (see reference 7) - in the ^1H NMR spectra of each MTPA ester diastereomer were unambiguously assigned.

The difference in chemical shift ($\Delta\delta^{SR}$) of analogous pairs of protons in the *S*- and *R*-MTPA esters were determined according to the following convention:

$$\Delta\delta^{SR}(=\delta_S - \delta_R)$$

where *S* = *S*-MTPA ester and *R* = *R*-MTPA ester

Positive and negative $\Delta\delta^{SR}$ values were then determined and tabulated as per Table S5, Table S6 and

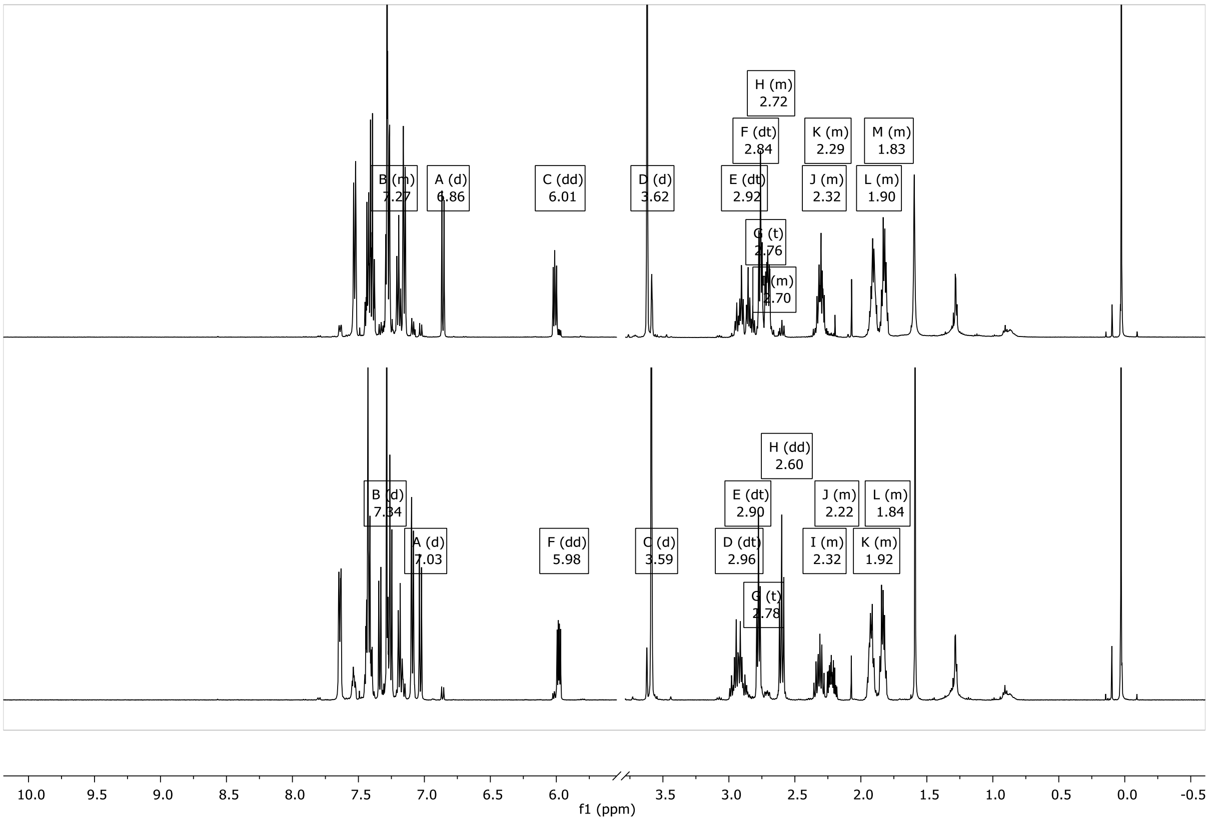

Figure **7**: Stacked ^{1}H NMR spectra (*500 MHz,* $CDCl_3$) 3-phenyl-1-(5,6,7,8-tetrahydroquinolin-2-yl)propyl (2*S*)-trifluoro-2-methoxy-2-phenylpropanoate (top spectrum) and 3-phenyl-1-(5,6,7,8-tetrahydroquinolin-2-yl)propyl (2*R*)-trifluoro-2-methoxy-2-phenylpropanoate (bottom spectru*m*).

Table S7. Relation of the values obtained to the conformations known to be spectroscopically important for Mosher Ester analysis, enabled the groups residing on the front plane (i.e. those in R_1, where $\Delta\delta^{SR}$ is positive) and the back plane (i.e. those in R_2, where $\Delta\delta^{SR}$ is negative) to be determined Figure 4. Using the Cahn-Inghold-Prelog convention the configuration of each carbinol centre was assigned as *R* or *S*.[19]

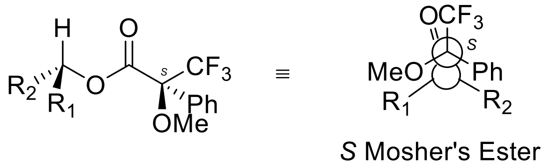

S Mosher's Ester

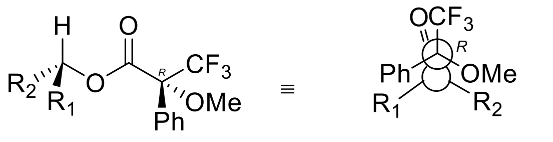

R Mosher's Ester

Figure 4: Conformers of R- and S-MTPA esters which exert greatest influence on spectroscopic features enabling the MTPA esters to be distinguished

Application of the Moscher's ester analysis method to carbinol (*R*)-1-(2'-pyridyl)ethanol of *known* stereochemistry (purchased from Acros organics, 382862500)

(*R*)-1-(pyridin-2-yl)ethyl-(*S*)-trifluoro-2-methoxy-2-phenylpropanoate

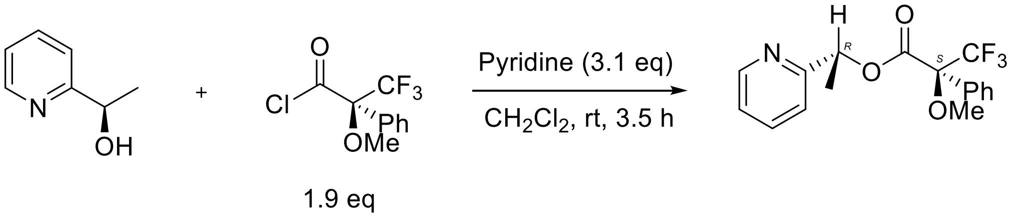

In a slight modification to the General Procedure B, (*R*)-1-(2'-pyridyl)ethanol (7.9 mg, 0.064 mmol) was weighed into a 4 mL, 15 × 45mm crimp-top vial. The vial was then sealed with a crimp seal, evacuated and purged with nitrogen. Sequentially, anhydrous pyridine (16 µL, 0.2 mmol, 3.1 eq), anhydrous CH_2Cl_2 (1 mL) and *R*-(-)-α-methoxy-α-(trifluoromethyl)phenylacetyl chloride (22 µL, 0.12 mmol, 1.9 eq) were added to the sealed vial *via* syringe. The reaction was determined complete by TLC analysis after 3.5 h. Following the work-up procedure outlined in General Procedure C, purification *via* flash column chromatography eluting with hexane/EtOAc 75:25, afforded the desired *S*-MPTA ester as a colourless oil (15 mg, 69% yield).

^{1}H NMR (500 MHz, $CDCl_3$) δ 8.58 (ddd, *J* = 4.9, 1.8, 1.0 Hz, 1H), 7.63 (td, *J* = 7.7, 1.8 Hz, 1H), 7.56 – 7.52 (m, 2H), 7.46 – 7.38 (m, 3H), 7.22 (ddd, *J* = 7.6, 4.8, 1.2 Hz, 1H), 7.16 (d, *J* = 7.9 Hz, 1H), 6.19 (q, *J* = 6.7 Hz, 1H), 3.63 (q, *J* = 1.3 Hz, 3H), 1.73 (d, *J* = 6.7 Hz, 3H); **^{13}C NMR** (126 MHz, $CDCl_3$) δ 165.8, 159.4, 149.3, 137.0, 132.3, 129.8, 128.5 (2 × C), 127.5 (q, *J* = 1.3 Hz, 2 × C), 123.5 (q, *J* = 288.5 Hz), 123.0, 120.1, 84.7 (q, *J* = 27.7 Hz), 75.6, 55. 7 (q, *J* = 1.5 Hz), 20.8;**^{19}F NMR** (471 MHz, $CDCl_3$) δ –72.5 **HRMS m/z:** $[M+H]^+$ calculated for $[C_{17}H_{17}F_3NO_3]^+$ 340.1155, found: 340.1160.

(*R*)-1-(pyridin-2-yl)ethyl-(*R*)-trifluoro-2-methoxy-2-phenylpropanoate

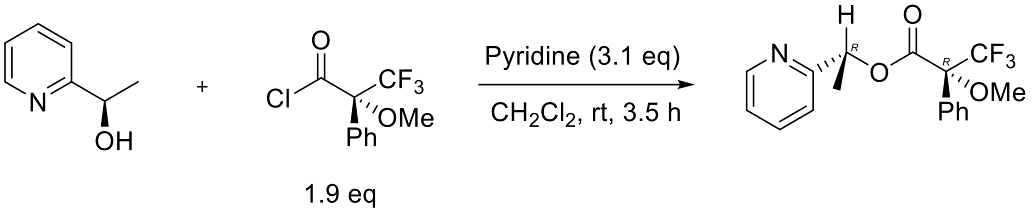

In a slight modification to the General Procedure B, (*R*)-1-(2'-pyridyl)ethanol (7.9 mg, 0.064 mmol) was weighed into a 4 mL, 15 × 45mm crimp-top vial. The vial was then sealed with a crimp seal, evacuated and purged with nitrogen. Sequentially, anhydrous pyridine (16 µL, 0.2 mmol, 3.1 eq), anhydrous CH_2Cl_2 (1 mL) and *S*-(+)-α-methoxy-α-(trifluoromethyl)phenylacetyl chloride (22 µL, 0.12 mmol, 1.9 eq) were added to the sealed vial *via* syringe. The reaction was determined complete by TLC analysis after 3.5 h. Following the work-up procedure outlined in General Procedure C, purification *via* flash column chromatography eluting with hexane/EtOAc 75:25, afforded the desired *R*-MPTA ester as a colourless oil (20 mg, 92% yield).

^{1}H NMR (500 MHz, $CDCl_3$) δ 8.62 (ddd, *J* = 4.8, 1.8, 0.9 Hz, 1H), 7.71 (td, *J* = 7.7, 1.8 Hz, 1H), 7.58 – 7.54 (m, 2H), 7.45 – 7.38 (m, 3H), 7.36 (dd, *J* = 7.9, 1.2 Hz, 1H), 7.25 (ddd, *J* = 7.6, 4.8, 1.2 Hz, 1H), 6.18 (q, *J* = 6.6 Hz, 1H), 3.56 (q, *J* = 1.2 Hz, 3H), 1.67 (d, *J* = 6.7 Hz, 3H); **^{13}C NMR** (126 MHz, $CDCl_3$) δ 165.9, 159.2,

149.4, 137.1, 132.3, 129.7, 128.5 (2 × C), 127.65 (q, *J* = 1.4 Hz, 2 × C), 123.5 (q, *J* = 288.4 Hz), 123.2, 120.6, 84.8 (q, *J* = 27.7 Hz), 75.7, 55.6 (q, *J* = 1.5 Hz), 20.5; **^{19}F NMR** (471 MHz, $CDCl_3$) δ –72.5 **HRMS m/z:** $[M+H]^+$ calculated for $[C_{17}H_{17}F_3NO_3]^+$ 340.1155, found: 340.1156.

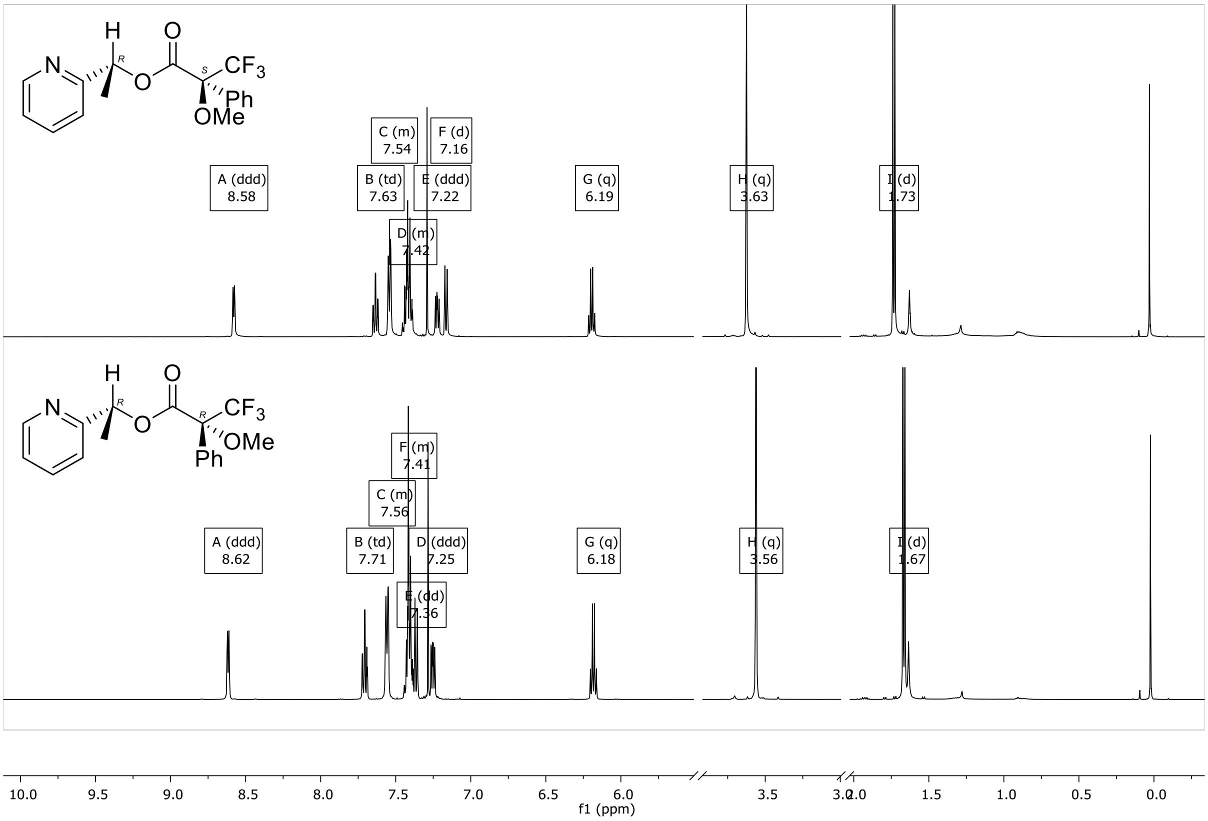

Figure 5: Stacked ^{1}H NMR spectra (500 MHz, $CDCl_3$) (R)-1-(pyridin-2-yl)ethyl-(S)-trifluoro-2-methoxy-2-phenylpropanoate (top spectrum) and (R)-1-(pyridin-2-yl)ethyl-(R)-trifluoro-2-methoxy-2-phenylpropanoate (bottom spectrum).

Table S5: Application of the Mosher's ester analysis method to carbinol of <u>known</u> stereochemistry:

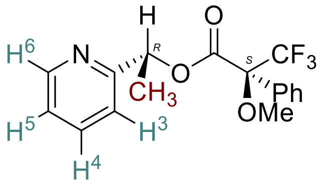

R-carbinol with S-Mosher's ester

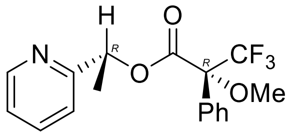

R-carbinol with R-Mosher's ester

In $CDCl_3$ (500 MHz):

			$\Delta\delta^{SR}(=\delta_S - \delta_R)$	
	δ S-ester (ppm)	δ R-ester (ppm)	ppm	Hz (500 MHz)
H^6	8.58	8.62	-0.04	-20
H^5	7.22	7.25	-0.03	-15
H^4	7.63	7.71	-0.08	-40
H^3	7.16	7.36	-0.2	-100
CH_3	1.73	1.67	+0.06	+30

Those with a positive $\Delta\delta^{SR}$ reside within R1 and those protons with a negative $\Delta\delta^{SR}$ reside within R2 in the conformations below

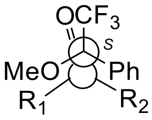

S Mosher's Ester

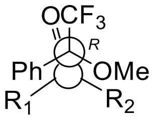

R Mosher's Ester

Based on the information outlined in the above table for $\Delta\delta^{SR}$ $R_1 = CH_3$ and R_2 = py

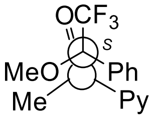

S Mosher's Ester

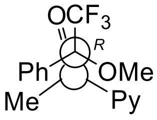

R Mosher's Ester

Following Cahn-Ingold convention the carbinol centre can therefore be assigned as *R* in accordance with the known absolute stereochemistry of this chiral carbinol centre.

Application of the Moscher's ester analysis method to carbinols (*R*)-1-(6-methylpyridin-2-yl)-3-phenylpropan-1-ol (Xb) and (*R*)-3-phenyl-1-(5,6,7,8-tetrahydroquinolin-2-yl)propan-1-ol (Xi) of *<u>unknown</u>* stereochemistry

1-(6-methylpyridin-2-yl)-3-phenylpropyl (2*S*)-trifluoro-2-methoxy-2-phenylpropanoate

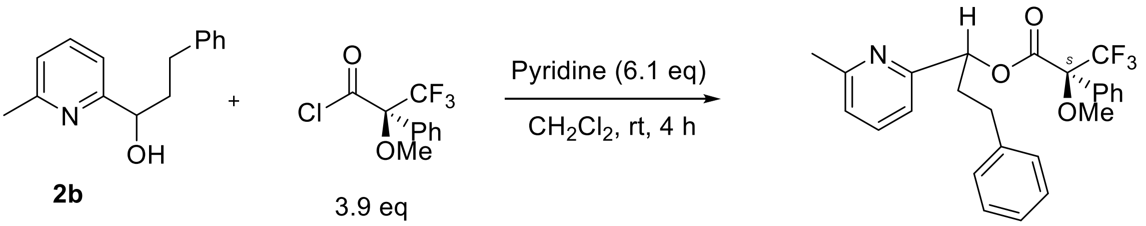

Following the General Procedure B, a solution of **2b** in anhydrous CH_2Cl_2 (7.5 mg, 0.033 mmol, 0.06 M), anhydrous pyridine (16 µL, 0.2 mmol, 6.1 eq), and *R*-(-)-α-methoxy-α-(trifluoromethyl)phenylacetyl chloride (24 µL, 0.13 mmol, 3.9 eq) were used. The reaction was determined complete by TLC analysis after 4 h. Following the work-up procedure outlined in General Procedure C, purification *via* flash column chromatography eluting with hexane/EtOAc 80:20, afforded the desired *S*-MPTA ester as a colourless oil (12 mg, 82% yield).

^{1}H NMR (400 MHz, $CDCl_3$) δ 7.55 – 7.51 (m, 2H overlapping with resonance δ 7.50 (app t, 1H)), 7.50 (app t, *J* = 7.5 Hz, 1H, overlapping with resonance δ 7.55 – 7.51 (m, 2H)), 7.46 – 7.39 (m, 3H), 7.32 – 7.26 (m, 2H), 7.23 – 7.14 (m, 3H), 7.07 (d, *J* = 7.8 Hz, 1H), 6.93 (d, *J* = 7.7 Hz, 1H), 6.05 (t, *J* = 6.5 Hz, 1H), 3.63 (s, 3H), 2.71 (dd, *J* = 9.8, 6.7 Hz, 2H), 2.54 (s, 3H), 2.37 – 2.28 (m, 2H); **^{13}C NMR** (126 MHz, $CDCl_3$) δ 166.0, 158.2, 157.8, 141.1, 137.2, 132.2, 129.7, 128.6 (2 × C), 128.50 (2 × C), 128.46 (2 × C), 127.7 (q, *J* = 1.4 Hz, 2 × C), 126.2, 123.5 (q, *J* = 288.5 Hz), 122.7, 117.5, 84.8 (q, *J* = 27.7 Hz), 78.9, 55.6 (q, *J* = 1.5 Hz), 37.0, 31.7, 24.4; **^{19}F NMR** (471 MHz, $CDCl_3$) –72.2 **HRMS m/z:** $[M+H]^+$ calculated for $[C_{25}H_{25}F_3NO_3]^+$ 444.1781, found: 444.1783.

1-(6-methylpyridin-2-yl)-3-phenylpropyl (2*R*)- trifluoro-2-methoxy-2-phenylpropanoate

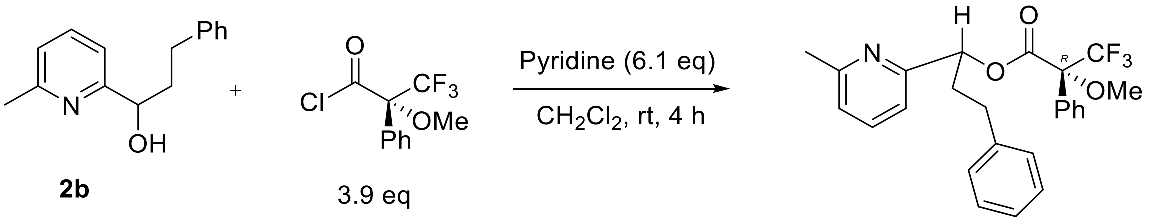

Following the General Procedure B, a solution of **2b** in anhydrous CH_2Cl_2 (7.5 mg, 0.033 mmol, 0.06 M), anhydrous pyridine (16 µL, 0.2 mmol, 6.1 eq), and *S*-(+)-α-methoxy-α-(trifluoromethyl)phenylacetyl chloride (24 µL, 0.13 mmol, 3.9 eq) were used. The reaction was determined complete by TLC analysis after 4 h. Following the work-up procedure outlined in General Procedure C, purification *via* flash column chromatography eluting with hexane/EtOAc 80:20, afforded the desired *R*-MPTA ester as a colourless oil (10 mg, 68% yield).

^{1}H NMR (400 MHz, $CDCl_3$) δ 7.64 (dd, *J* = 7.3, 2.1 Hz, 2H), 7.57 (t, *J* = 7.7 Hz, 1H), 7.45 – 7.39 (m, 3H), 7.28 – 7.24 (m, 2H), 7.19 (t, *J* = 7.5 Hz, 1H), 7.11 – 7.07 (m, 3H), 6.01 (dd, *J* = 8.5, 4.5 Hz, 1H), 3.59 (s, 3H), 2.61 (app t, *J* = 8.1 Hz, 2H. overlapping with resonance δ 2.58 (s, 3H)), 2.58 (s, 3H, overlapping with resonance δ 2.61 (t, 2H)), 2.40 – 2.30 (m, 1H), 2.30 – 2.18 (m, 1H).**^{13}C NMR** (101 MHz, $CDCl_3$) δ 166.1, 158.4, 157.7, 141.1, 137.0, 132.4, 129.8, 128.6 (2 × C), 128.52 (2 × C), 128.48 (2 × C), 127.7 (2 × C), 126.2, 123.6 (q, *J* = 288.4 Hz), 122.8, 118.1, 84.7 (q, *J* = 27.8 Hz), 78.8, 55.7, 36.9, 31.4, 24.5. **^{19}F NMR** (471 MHz, $CDCl_3$) −72.2. **HRMS m/z:** $[M+H]^+$ calculated for $[C_{25}H_{25}F_3NO_3]^+$ 444.1781, found: 444.1792.

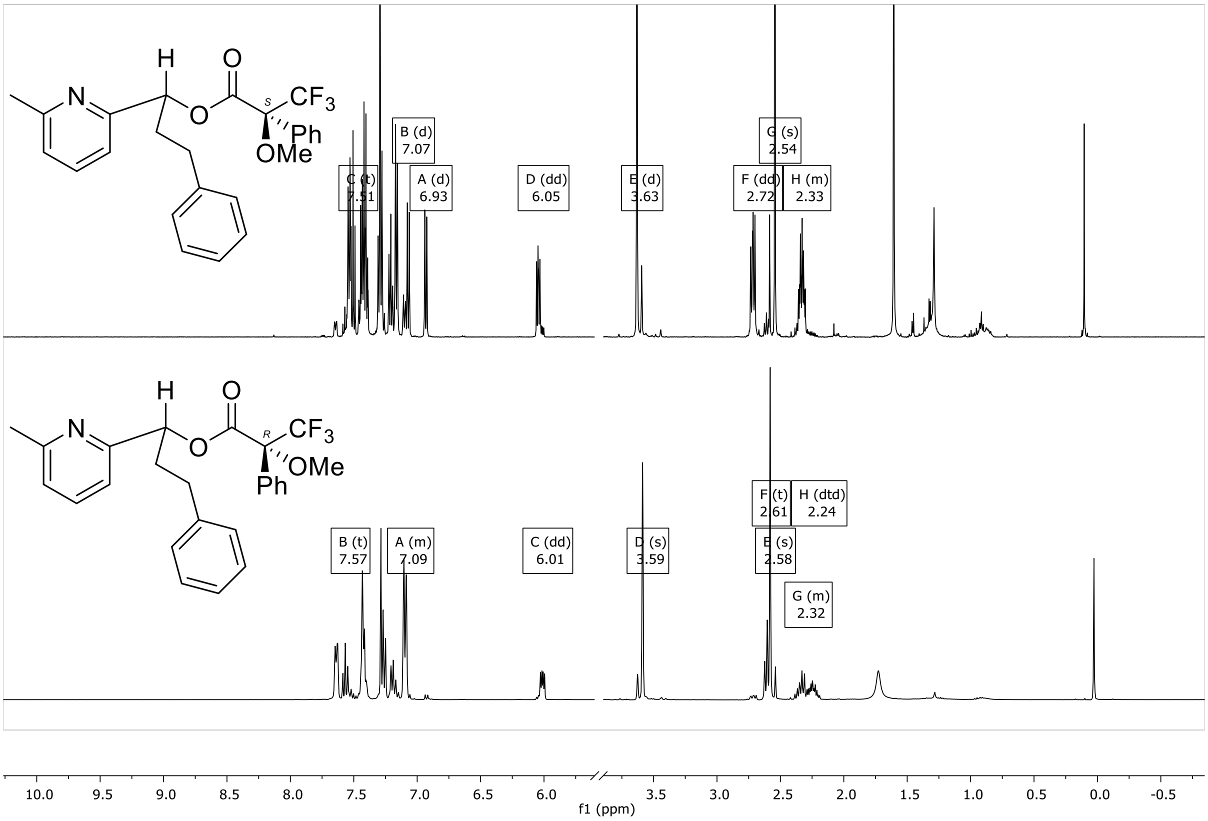

Figure 6: Stacked ^{1}H NMR spectra ($CDCl_3$) 1-(6-methylpyridin-2-yl)-3-phenylpropyl (2S)-trifluoro-2-methoxy-2-phenylpropanoate (top spectrum; 500 MHz) and 1-(6-methylpyridin-2-yl)-3-phenylpropyl (2R)- trifluoro-2-methoxy-2-phenylpropanoate (bottom spectrum, 400 MHz).

Table S6: Application of the Mosher's ester analysis method to carbinol of <u>unknown</u> stereochemistry:

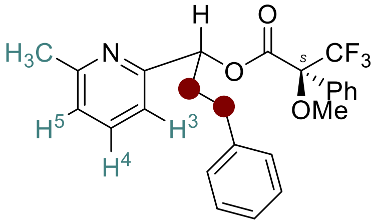

with S-Mosher's ester

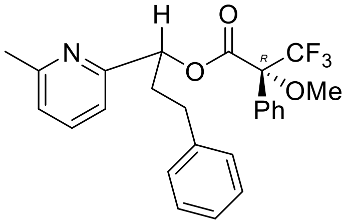

with R-Mosher's ester

In $CDCl_3$ (400 MHz):

			$\Delta\delta^{SR}(=\delta_S - \delta_R)$		
	δ S-ester (ppm)	δ R-ester (ppm)	ppm	Hz (500 MHz)	
CH_3	2.54	2.58	-0.04	-20	
H^3	6.93	7.09	-0.16	-80	
H^4	7.50	7.57	-0.07	-35	
H^5	7.07	7.09	-0.02	-10	Discount small values
OCH***CH₂***CH₂Ph	2.32	2.34	-0.02	-10	
	2.32	2.24	+0.08	+40	
OCHCH₂***CH₂***Ph	2.71	2.61	+0.1	+50	

Based on the information outlined in the above table for $\Delta\delta^{SR}$ $R_1 = CH_2CH_2Ph$ and R_2 = py

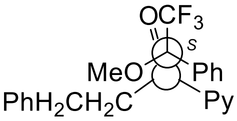

S Mosher's Ester

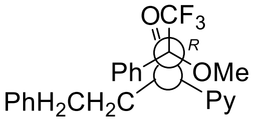

R Mosher's Ester

Following Cahn-Ingold convention the carbinol centre can therefore be assigned as *R*.

3-phenyl-1-(5,6,7,8-tetrahydroquinolin-2-yl)propyl (2*S*)- trifluoro-2-methoxy-2-phenylpropanoate

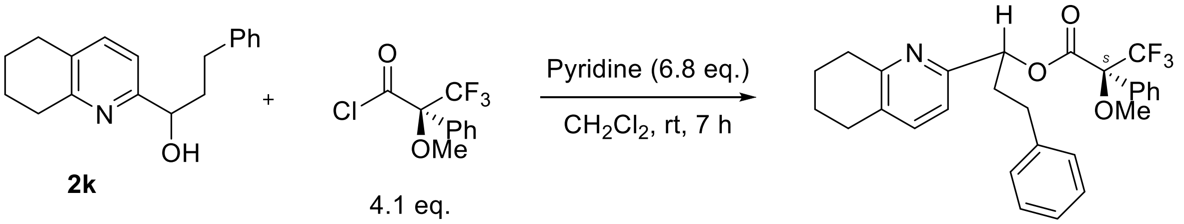

Following the General Procedure B, a solution of **2k** in anhydrous CH_2Cl_2 (6.0 mg, 0.022 mmol, 0.06 M), anhydrous pyridine (12 µL, 0.15 mmol, 6.8 eq), and *R*-(-)-α-methoxy-α-(trifluoromethyl)phenylacetyl chloride (16 µL, 0.09 mmol, 4.1 eq) were used. The reaction was determined complete by TLC analysis after 7 h. Following the work-up procedure outlined in General

Procedure C, purification *via* flash column chromatography eluting with hexane/EtOAc 80:20, afforded the desired *S*-MPTA ester as a colourless oil (7 mg, 65% yield).

^{1}H NMR (500 MHz, $CDCl_3$) δ 7.55 – 7.51 (m, 2H), 7.44 – 7.37 (m, 3H), 7.30 – 7.25 (m, 3H), 7.22 – 7.17 (m, 1H), 7.17 – 7.14 (m, 2H), 6.86 (d, *J* = 7.9 Hz, 1H), 6.01 (dd, *J* = 7.5, 5.5 Hz, 1H), 3.62 (d, *J* = 1.3 Hz, 3H), 2.92 (dt, *J* = 17.7, 6.5 Hz, 1H), 2.84 (dt, *J* = 17.7, 6.3 Hz, 1H), 2.76 (t, *J* = 6.3 Hz, 2H), 2.73 – 2.71 (m, 1H), 2.70 (dd, *J* = 6.6, 2.2 Hz, 1H), 2.34 – 2.30 (m, 1H), 2.30 – 2.27 (m, 1H), 1.95 – 1.87 (m, 2H), 1.86 – 1.78 (m, 2H); **^{13}C NMR** (126 MHz, $CDCl_3$) δ 166.0, 157.1, 155.3, 141.2, 137.4, 132.3, 131.7, 129.7, 128.6 (2 × C), 128.5 (4 × C), 127.7 (2 × C), 126.2, 123.5 (q, *J* = 288.4 Hz), 117.6, 84.8 (q, *J* = 27.7 Hz), 79.2, 55.8, 37.0, 32.6, 31.7, 28.7, 23.2, 22.7. **^{19}F NMR** (471 MHz, $CDCl_3$) δ –72.3 **HRMS m/z:** $[M+H]^+$ calculated for $[C_{28}H_{29}F_3NO_3]^+$ 484.2094, found: 484.2102.

3-phenyl-1-(5,6,7,8-tetrahydroquinolin-2-yl)propyl (2*R*)-trifluoro-2-methoxy-2-phenylpropanoate

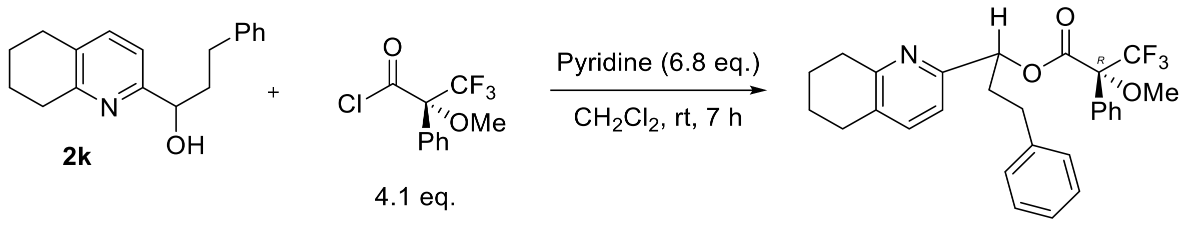

Following the General Procedure B, a solution of **2k** in anhydrous CH_2Cl_2 (6.0 mg, 0.022 mmol, 0.06 M), anhydrous pyridine (12 µL, 0.15 mmol, 6.8 eq), and *S*-(+)-α-methoxy-α-(trifluoromethyl)phenylacetyl chloride (16 µL, 0.09 mmol, 4.1 eq) were used. The reaction was determined complete by TLC analysis after 7 h. Following the work-up procedure outlined in General Procedure C, purification *via* flash column chromatography eluting with hexane/EtOAc 80:20, afforded the desired *R*-MPTA ester as a colourless oil (10 mg, 92% yield).

^{1}H NMR (500 MHz, $CDCl_3$) δ 7.68 – 7.60 (m, 2H), 7.45 – 7.40 (m, 3H), 7.34 (d, *J* = 7.9 Hz, 1H), 7.28 – 7.24 (m, 2H), 7.21 – 7.15 (m, 1H), 7.12 – 7.07 (m, 2H), 7.03 (d, *J* = 7.9 Hz, 1H), 5.98 (dd, *J* = 8.5, 4.5 Hz, 1H), 3.59 (d, *J* = 1.2 Hz, 3H), 2.96 (dt, *J* = 17.7, 6.4 Hz, 1H), 2.90 (dt, *J* = 17.6, 6.3 Hz, 1H), 2.78 (t, *J* = 6.3 Hz, 2H), 2.60 (dd, *J* = 9.0, 7.2 Hz, 2H), 2.38 – 2.27 (m, 1H), 2.22 (ddt, *J* = 12.4, 9.6, 4.3 Hz, 1H), 1.95 – 1.89 (m, 2H), 1.87 – 1.79 (m, 2H); **^{13}C NMR** (126 MHz, $CDCl_3$) δ 166.3, 157.2, 155.2, 141.2, 137.5, 132.5, 131.8, 129.7, 128.53 (2 × C), 128.50 (2 × C), 128.49 (2 × C), 127.8 (2 × C), 126.1, 123.61 (q, *J* = 288.5 Hz), 118.2, 84.7 (q, *J* = 27.7 Hz), 79.2, 55.7, 36.9, 32.7, 31.4, 28.7, 23.2, 22.7; **^{19}F NMR** (471 MHz, $CDCl_3$) δ –72.2 **HRMS m/z:** $[M+H]^+$ calculated for $[C_{28}H_{29}F_3NO_3]^+$ 484.2094, found: 484.2102.

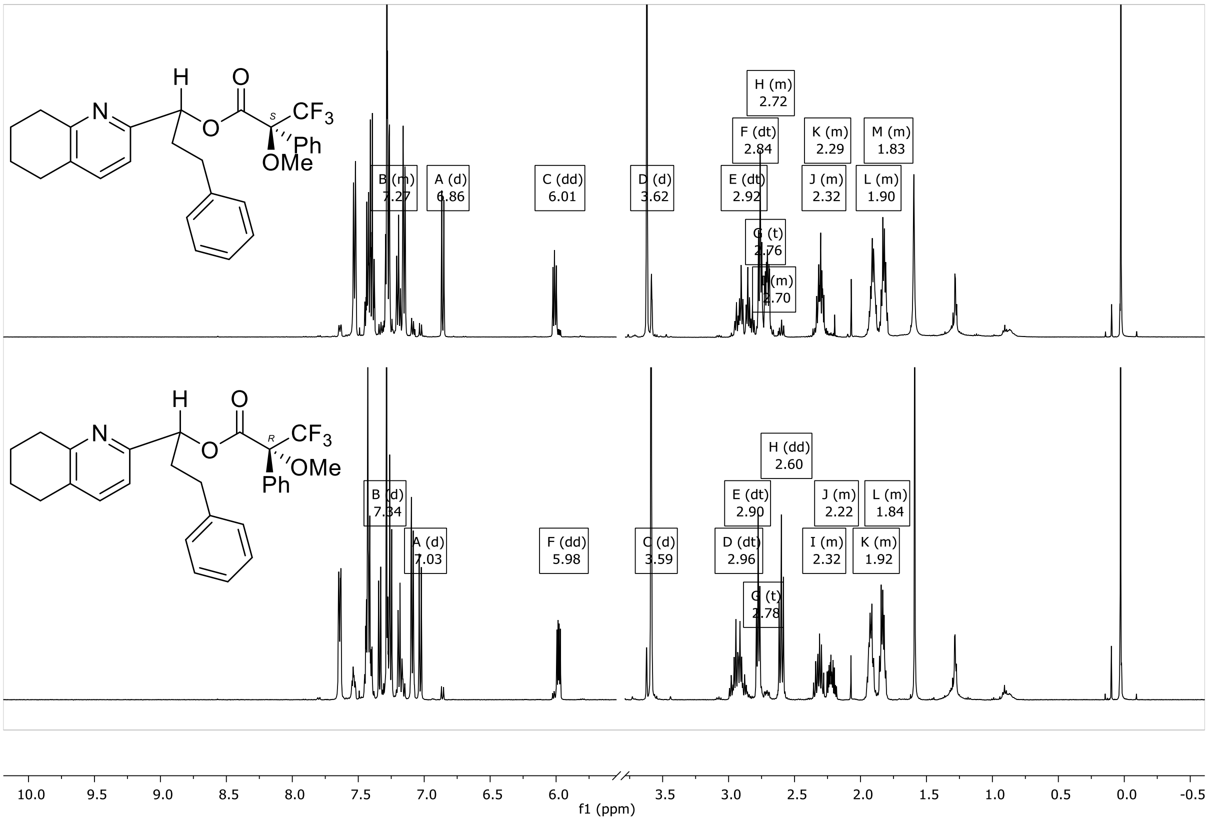

Figure 7: Stacked ^{1}H NMR spectra (*500 MHz,* $CDCl_3$) 3-phenyl-1-(5,6,7,8-tetrahydroquinolin-2-yl)propyl (2*S*)-trifluoro-2-methoxy-2-phenylpropanoate (top spectrum) and 3-phenyl-1-(5,6,7,8-tetrahydroquinolin-2-yl)propyl (2*R*)-trifluoro-2-methoxy-2-phenylpropanoate (bottom spectru*m*).

Table S7: Application of the Mosher's ester analysis method to carbinol of <u>unknown</u> stereochemistry:

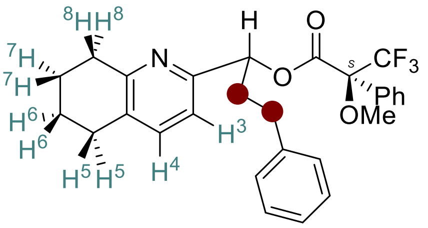

with S-Mosher's ester

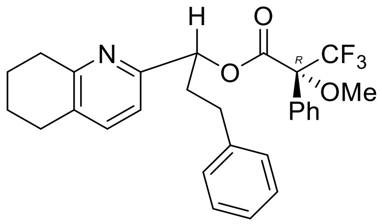

with R-Mosher's ester

In $CDCl_3$ (500 MHz):

			$\Delta\delta^{SR}(=\delta_S - \delta_R)$		
	δ S-ester (ppm)	δ R-ester (ppm)	ppm	Hz (500 MHz)	
H^3	6.86	7.03	-0.17	-85	
H^4	7.27	7.34	-0.07	-35	
H^8	2.92	2.96	-0.04	-20	
	2.84	2.90	-0.06	-30	
H^7	1.90	1.92	-0.02	-10	
H^6	1.83	1.83	0	0	Small values

H[5]	2.76	2.78	-0.02	-10	discounted
OCH***CH$_2$***CH$_2$Ph	2.32	2.31	-0.01	-5	
	2.29	2.22	+0.07	+35	
OCHCH$_2$***CH$_2$***Ph	2.72	2.60	+0.12	+60	
	2.70	2.60	+0.1	+50	

Based on the information outlined in the above table for $\Delta\delta^{SR}$ R_1 = CH_2CH_2Ph and R_2 = py

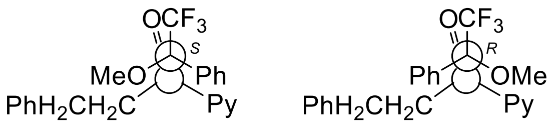

S Mosher's Ester *R* Mosher's Ester

Following Cahn-Ingold convention the carbinol centre can therefore be assigned as *R*.

Control Experiments and Experiments to Probe Mechanism

Control Reaction with no H-Bond Donor

2-(1-methoxy-3-phenylpropyl)-6-pentylpyridine (7a)

Preparation of Racemic Sample of Control Product **7a**

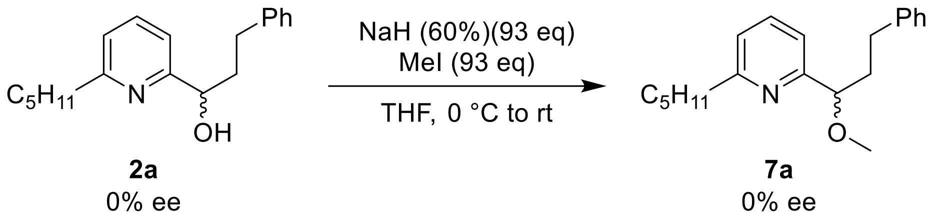

Under Nitrogen atmosphere, racemic **2a** (4.0 mg, 14 µmol) was dissolved in anhydrous THF (2 mL). The flask was cooled to 0 °C (using ice/water) and NaH (60% mineral oil) (50 mg, 1.3 mmol, 93 eq) was added in one portion. The ice bath was removed and the reaction stirred at room temperature for 1 h. The flask was again cooled to 0 °C. Methyl iodide (78 µL, 1.3 mmol, 93 eq) was added *via* microsyringe. The flask was left to warm slowly to rt and stirred overnight. The reaction was quenched with MeOH (0.5 mL) and diluted with H_2O (3 mL). The mixture was transferred to a separating funnel and the aqueous layer was extracted with EtOAc (2 × 5 mL). The organic layer was dried over Na_2SO_4, filtered and concentrated *in vacuo*. The crude material was purified *via* flash column chromatography eluting with hexane/EtOAc 100:0 to 85:15, to afford the desired product **7a** as a colourless oil (4 mg, 95% yield).

^{1}H NMR (400 MHz, $CDCl_3$) δ 7.59 (t, *J* = 7.7 Hz, 1H), 7.28 – 7.22 (m, 2H), 7.22 – 7.12 (m, 4H), 7.03 (d, *J* = 7.7 Hz, 1H), 4.28 (t, *J* = 6.5 Hz, 1H), 3.32 (s, 3H), 2.83 – 2.73 (m, 3H), 2.67 (dt, *J* = 13.7, 8.1 Hz, 1H), 2.10 – 1.98 (m, 2H), 1.76 – 1.65 (m, 2H), 1.37 – 1.29 (m, 4H), 0.94 – 0.82 (m, 3H); **^{13}C NMR** (101 MHz, $CDCl_3$) δ 162.1, 161.6, 142.3, 136.9, 128.6 (2 × C), 128.4 (2 × C), 125.8, 121.3, 117.5, 84.6, 57.3, 38.9, 38.6, 32.1, 31.8, 30.0, 22.7, 14.2. **HRMS m/z:** $[M+H]^+$ calculated for $[C_{20}H_{28}NO]^+$ 298.2165, found: 298.2167.

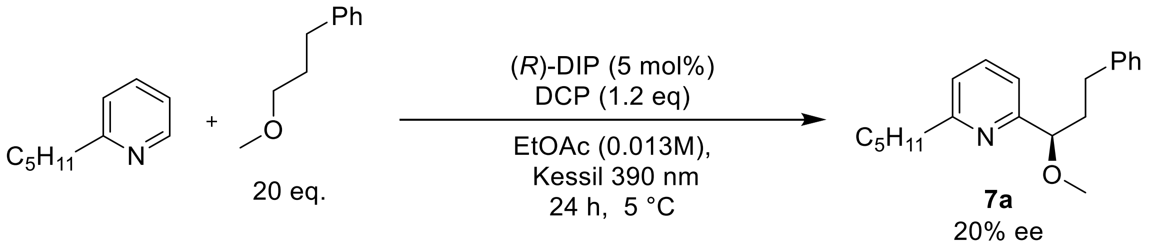

In a slight modification to General Procedure A, 2-pentylpyridine (29.9 mg, 0.2 mmol), 3-phenyl-1-methoxypropane (601 mg, 4.0 mmol, 20 eq), dicumyl peroxide (64.9 mg, 0.24 mmol, 1.2 eq) and (*R*)-DIP (6.7 mg, 0.01 mmol, 5 mol%) were used. Analysis of the ^{1}H NMR spectrum of the crude material with reference to 1,3,5-trimethoxybenzene as an internal standard determined the NMR yield of **7a** to be 14% with 68% of the 2-pentylpyridine starting material unreacted. The crude material was purified *via* an initial flash column chromatography eluting with CH_2Cl_2/EtOAc 100:0 to 70:30, followed by subsequent flash column chromatography eluting with hexane:EtOAc 100:0 to 95:05 to afford the desired product **7a** as a colourless oil but containing small amounts of an unidentified impurity (8 mg, 13% yield, 20% ee).

SFC Analysis: Chiralpak IE (CO_2/MeOH = 95/05, 2.5 mL min^{-1}, 40 °C, 263 nm) t_R = 4.3 (major), 4.6 (minor) minutes.

Intermolecular Competition KIE Experiment

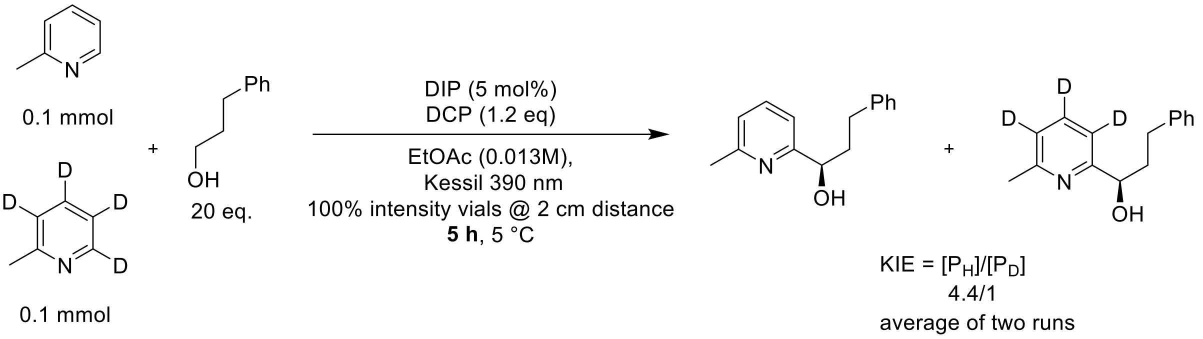

KIE = $[P_H]/[P_D]$
4.4/1
average of two runs

In a slight modification to General Procedure A, 2-methylpyridine (10 µL, 0.1 mmol) and 2-methylpyridine-*d_4* (9.7 mg, 0.1 mmol), 3-phenyl-1-propanol (545 mg, 4.0 mmol, 20 eq w.r.t. 0.2 mmol), dicumyl peroxide (64.9 mg, 0.24 mmol, 1.2 eq w.r.t. 0.2 mmol) and (*R*)-DIP (6.7 mg, 0.01 mmol, 5 mol% w.r.t. 0.2 mmol) were used. The reaction was stopped after irradiation with 390 nm light for **5h**. The crude material was purified *via* an initial flash column chromatography eluting with CH_2Cl_2/EtOAc 100:0 to 90:10, followed by subsequent flash column chromatography eluting with hexane/EtOAc 90:10 to afford the desired products as a mixture of isotopes. Analysis of the ^{1}H NMR spectrum of the isolated products determined the product ratio to be 4.4:1 for the product resulting from addition to 2-methylpyridine compared to 2-methylpyridine-*d_4* (average of two runs).

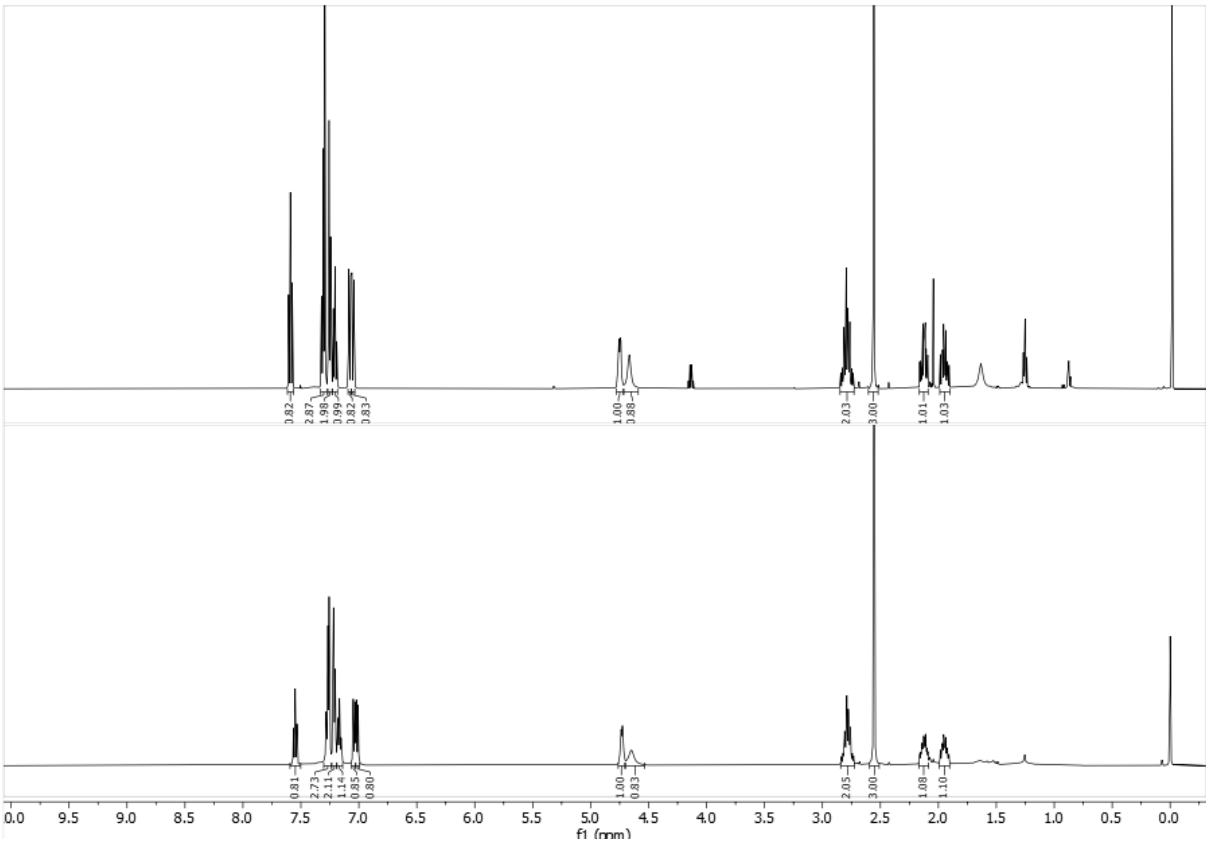

Figure 8: Stacked ^{1}H NMR spectra (500 MHz, $CDCl_3$) of isolated mixture of isotopes from intermolecular competition experiment; run 1 (top spectrum) run 2 (bottom spectrum).

Computational Investigations

Computational methods

All conformational searches were performed using MacroModel (Version 9.9) in the gas phase utilizing the OPLS3 force field[11] and a mixture of Low Mode following and Monte Carlo search algorithms.[12] Quantum mechanical calculations were carried out using Gaussian16.[13] DFT structure optimisations were done either in gas phase or using the implicit SMD solvent model.[14] All single point calculations were done using the SMD solvent model. The molecular geometries were optimized at the DFT level of theory using the B3LYP functional[15] with the 6-31G** basis set.[16] Single-point energies were separately calculated using M06-2X functional[17] and def2-TZVP[18]. On a model system, single point energies were also calculated using double-hybrid B2PLYPD3[19] in combination with def2-TZVP basis set. Frequency calculations were performed on all structures and confirmed to contain no imaginary frequencies or just one imaginary frequency for ground states and transition states, respectively. The free energies were corrected using quasi-harmonic approximation, corrections were done using GoodVibes script.[20] Full set of DFT output files with optimized structures, frequencies and high-level single-point energies are provided (see below for a description) and can be found at DOI: http://doi.org/10.17639/nott.7152

Model study

In previous studies we had encountered a known systematic error in standard DFT methods (B3LYP and M06-2X) which overestimated the stability of delocalized radicals in the reaction mechanism. To check for this issue in the modelling of the current Minisci reaction with alcohol coupling partners, a model study of the overall reaction mechanism was conducted, and results from M06-2X and B2PLYD3 single-point energy calculations compared (Figure 9).

As can be seen, a similar systematic error can be seen for the standard M06-2X functional as previously (Figure 9B). The energies of more localized radical species at the beginning of the mechanism are increased and the energies of more delocalized radical species are decreased, when compared to the double-hybrid B2PLYPD3 results (Figure 9C). In the M06-2X energy diagram it is not clear if addition or deprotonation is more likely to be selectivity determining, while double-hybrid B2PLYPD3 results unambiguously show that deprotonation is the selectivity-determining step.

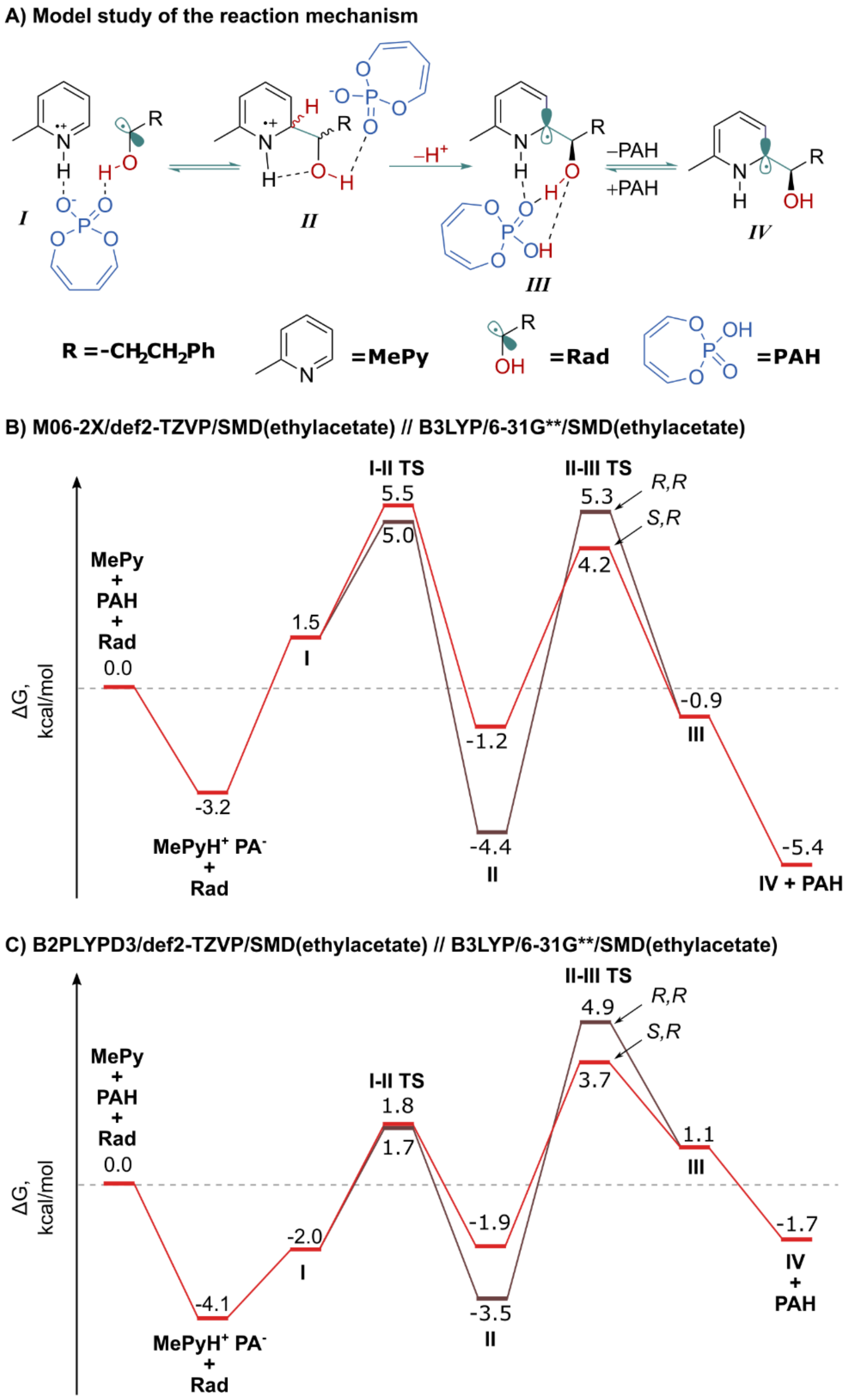

Figure 9: Comparison of model reaction mechanism (**A**) energy diagrams calculated at M06-2X/def2-TZVP/SMD(ethylacetate) (**B**) and B2PLYPD3/def2-TZVP/SMD(ethylacetate) (**C**).

Non-covalent interaction plots for lowest energy full system deprotonation transition states optimized at B3LYP/6-31G*/SMD(ethylacetate)

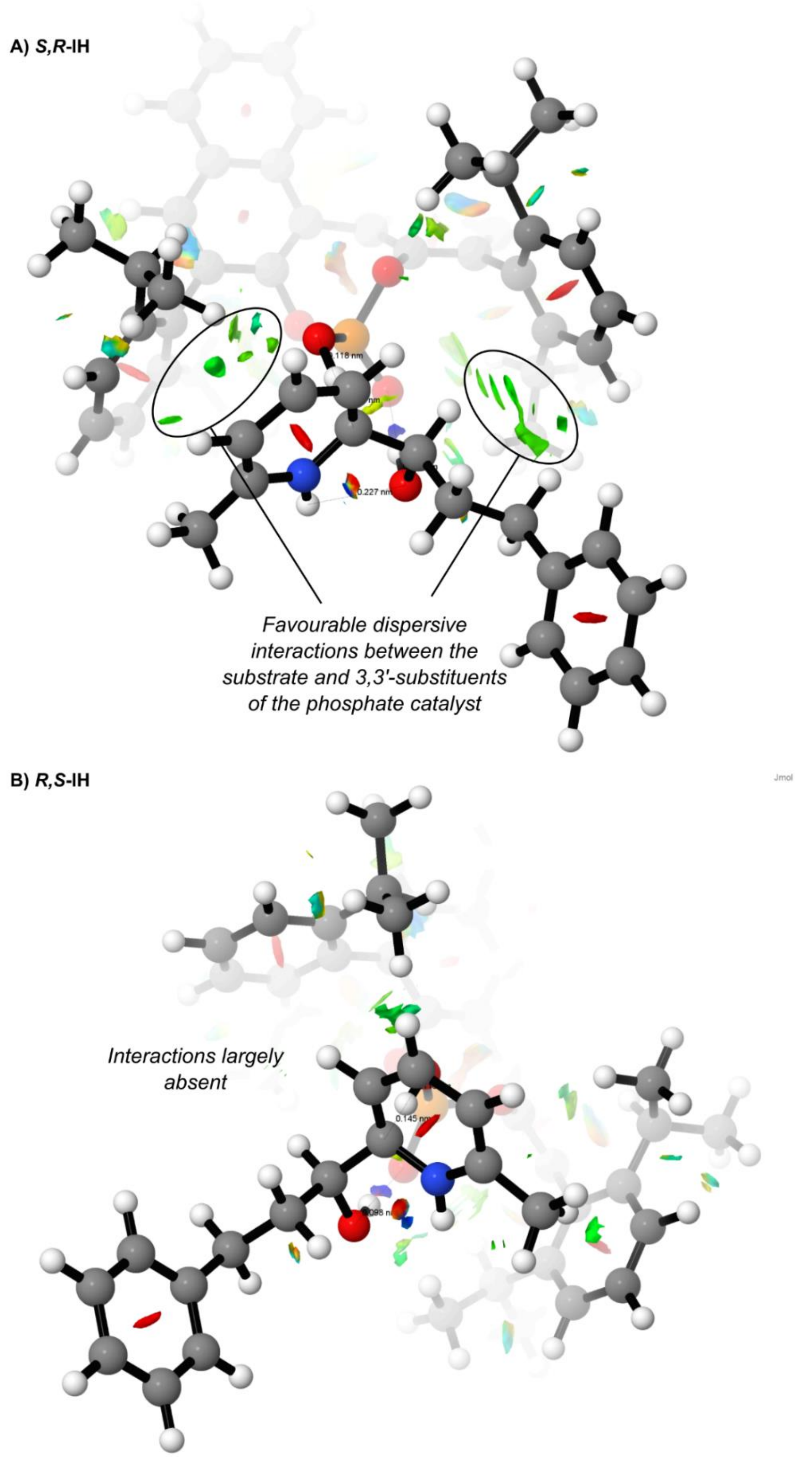

Figure 10: Non-covalent interaction plots for the B3LYP/6-31G**/SMD(ethylacetate) optimized deprotonation transition state geometries - experimentally major ***S,R*-IH** (**A**) and experimentally minor ***R,S*-IH** (**B**).

Distortion-interaction analysis of the lowest energy transition states for the major and minor enantiomeric products

Table S8: Distortion-interaction analysis of the lowest energy transition states for the major (***S*,*R*-IH**) and the minor (***R*,S-IH**) enantiomeric product. All energies are electronic energies calculated at M06-2X / 6-31G** / SMD(ethylacetate) and quoted in a.u. All geometries have been optimized at M06-2X / 6-31G** / SMD(ethylacetate). All energy differences quoted in kcal/mol.

**Component**	**Minimum geometry**	**As in S,R-IH**	**As in R,S-IH**	**Difference between minor and major TS** (kcal/mol)
**DIP phosphate** (a.u.)	-2345.70302274	-2345.69650752	-2345.6969747	
**DIP phosphate distortion** (kcal/mol)		4.09	3.80	-0.29
**Substrate** (a.u.)	-712.7869049	-712.7602353	-712.757296821	
**Substrate distortion** (kcal/mol)		16.74	18.58	1.84
**Full system** (a.u.)		-3058.524503	-3058.522967	
**DIP phosphate-substrate interaction** (kcal/mol)		-42.52	-43.11	-0.59
**Interaction-distortion difference** (kcal/mol)		-21.70	-20.73	0.96

Summary of the associated computational dataset contents

This dataset contains Gaussian DFT output files of the key ground-states and transition state DFT optimized structures. It is available at DOI: http://doi.org/10.17639/nott.7152

There are two top level folders. 'ModelSystem_Full_Pathway' contains computational files from the whole phosphoric acid-catalyzed pathway exploration using a model catalyst (Figure 2 in the paper and Figure 6 in ESI). The subfolders are named according to the notation used in the figures.

'FullSystem_Deprotonation' contains computational files from the investigation of the deprotonation transition state using the full DIP catalyst structure. It has 2 subfolders - B3LYP_SMD_opt and M06-2X_SMD_opt, containing the geometries optimized at the respective levels of theory.

Each of the lower-level folders contain the output of a frequency calculation at B3LYP/6-31G** level with SMD(1,4-dioxane) solvent model (*_freq.out files), as well as at least one single point calculation at a higher level, either at M06-2X/def2-TZVP/SMD(1,4-dioxane) or B2PLYPD3/def2-TZVP/SMD(1,4-dioxane) level (*_sp.out files). All optimized geometries are also provided as *.sdf files for even better usability.

All of the files can be opened in any text editor. Gaussian output structures can be viewed and the frequency modes visualised in GausView, Avogadro, jmol and in most other molecular viewers/editors. *.sdf files can be viewed in essentially all 3D molecular editors and viewers.

```
├── FullSystem_Deprotonation
│   ├── B3LYP_SMD_opt
│   │   ├── RR1F_B3LYP_freq.out
│   │   ├── RR1F_M062X_def2-TZVP_sp.out
│   │   ├── RR1F_M062X_def2-TZVP_sp.sdf
│   │   ├── RR2F_B3LYP_freq.out
│   │   ├── RR2F_M062X_def2-TZVP_sp.out
│   │   ├── RR2F_M062X_def2-TZVP_sp.sdf
│   │   ├── RR8F_B3LYP_freq.out
│   │   ├── RR8F_M062X_def2-TZVP_sp.out
│   │   ├── RR8F_M062X_def2-TZVP_sp.sdf
│   │   ├── RS22F_B3LYP_freq.out
│   │   ├── RS22F_M062X_def2-TZVP_sp.out
│   │   ├── RS22F_M062X_def2-TZVP_sp.sdf
│   │   ├── RS23F_B3LYP_freq.out
│   │   ├── RS23F_M062X_def2-TZVP_sp.out
│   │   ├── RS23F_M062X_def2-TZVP_sp.sdf
│   │   ├── SR50F_B3LYP_freq.out
│   │   ├── SR50F_M062X_def2-TZVP_sp.out
│   │   ├── SR50F_M062X_def2-TZVP_sp.sdf
│   │   ├── SR51F_B3LYP_freq.out
│   │   ├── SR51F_M062X_def2-TZVP_sp.out
│   │   ├── SR51F_M062X_def2-TZVP_sp.sdf
│   │   ├── SS12F_B3LYP_freq.out
│   │   ├── SS12F_M062X_def2-TZVP_sp.out
│   │   ├── SS12F_M062X_def2-TZVP_sp.sdf
│   │   ├── SS1F_B3LYP_freq.out
│   │   ├── SS1F_M062X_def2-TZVP_sp.out
│   │   └── SS1F_M062X_def2-TZVP_sp.sdf
│   └── M06-2X_SMD_opt
│       ├── RS23C_M062X_def2-TZVP_sp.out
│       ├── RS23C_M062X_def2-TZVP_sp.sdf
│       ├── RS23C_M062X_freq.out
│       ├── SR51C_M062X_def2-TZVP_sp.out
│       ├── SR51C_M062X_def2-TZVP_sp.sdf
│       └── SR51C_M062X_freq.out

├── ModelSystem_Full_Pathway
│   ├── I
│   │   ├── SMC_B2PLYPD3_def2-TZVP_sp.out
│   │   ├── SMC_B3LYP_freq.out
│   │   ├── SMC_M062X_def2-TZVP_sp.out
│   │   └── SMC_M062X_def2-TZVP_sp.sdf
│   ├── II
```

```
│   │   ├── RSimC_B2PLYPD3_def2-TZVP_sp.out
│   │   ├── RSimC_B3LYP_freq.out
│   │   ├── RSimC_M062X_def2-TZVP_sp.out
│   │   ├── RSimC_M062X_def2-TZVP_sp.sdf
│   │   ├── SSimC_B2PLYPD3_def2-TZVP_sp.out
│   │   ├── SSimC_B3LYP_freq.out
│   │   ├── SSimC_M062X_def2-TZVP_sp.out
│   │   └── SSimC_M062X_def2-TZVP_sp.sdf
│   ├── III
│   │   ├── ProdC_B2PLYPD3_def2-TZVP_sp.out
│   │   ├── ProdC_B3LYP_freq.out
│   │   ├── ProdC_M062X_def2-TZVP_sp.out
│   │   └── ProdC_M062X_def2-TZVP_sp.sdf
│   ├── I-II
│   │   ├── RSaddF_B2PLYPD3_def2-TZVP_sp.out
│   │   ├── RSaddF_B3LYP_freq.out
│   │   ├── RSaddF_M062X_def2-TZVP_sp.out
│   │   ├── RSaddF_M062X_def2-TZVP_sp.sdf
│   │   ├── SSaddF_B2PLYPD3_def2-TZVP_sp.out
│   │   ├── SSaddF_B3LYP_freq.out
│   │   ├── SSaddF_M062X_def2-TZVP_sp.out
│   │   └── SSaddF_M062X_def2-TZVP_sp.sdf
│   ├── II-III
│   │   ├── RSdepr1F_B2PLYPD3_def2-TZVP_sp.out
│   │   ├── RSdepr1F_B3LYP_freq.out
│   │   ├── RSdepr1F_M062X_def2-TZVP_sp.out
│   │   ├── RSdepr1F_M062X_def2-TZVP_sp.sdf
│   │   ├── RSdepr32F_B2PLYPD3_def2-TZVP_sp.out
│   │   ├── RSdepr32F_B3LYP_freq.out
│   │   ├── RSdepr32F_M062X_def2-TZVP_sp.out
│   │   ├── RSdepr32F_M062X_def2-TZVP_sp.sdf
│   │   ├── RSdepr3F_B2PLYPD3_def2-TZVP_sp.out
│   │   ├── RSdepr3F_B3LYP_freq.out
│   │   ├── RSdepr3F_M062X_def2-TZVP_sp.out
│   │   ├── RSdepr3F_M062X_def2-TZVP_sp.sdf
│   │   ├── RSdepr59F_B2PLYPD3_def2-TZVP_sp.out
│   │   ├── RSdepr59F_B3LYP_freq.out
│   │   ├── RSdepr59F_M062X_def2-TZVP_sp.out
│   │   ├── RSdepr59F_M062X_def2-TZVP_sp.sdf
│   │   ├── RSdepr9F_B2PLYPD3_def2-TZVP_sp.out
│   │   ├── RSdepr9F_B3LYP_freq.out
│   │   ├── RSdepr9F_M062X_def2-TZVP_sp.out
│   │   ├── RSdepr9F_M062X_def2-TZVP_sp.sdf
│   │   ├── SSdepr12F_B2PLYPD3_def2-TZVP_sp.out
│   │   ├── SSdepr12F_B3LYP_freq.out
│   │   ├── SSdepr12F_M062X_def2-TZVP_sp.out
│   │   ├── SSdepr12F_M062X_def2-TZVP_sp.sdf
│   │   ├── SSdepr13F_B2PLYPD3_def2-TZVP_sp.out
```

```
│   │   ├── SSdepr13F_B3LYP_freq.out
│   │   ├── SSdepr13F_M062X_def2-TZVP_sp.out
│   │   ├── SSdepr13F_M062X_def2-TZVP_sp.sdf
│   │   ├── SSdepr1F_B2PLYPD3_def2-TZVP_sp.out
│   │   ├── SSdepr1F_B3LYP_freq.out
│   │   ├── SSdepr1F_M062X_def2-TZVP_sp.out
│   │   ├── SSdepr1F_M062X_def2-TZVP_sp.sdf
│   │   ├── SSdepr4F_B2PLYPD3_def2-TZVP_sp.out
│   │   ├── SSdepr4F_B3LYP_freq.out
│   │   ├── SSdepr4F_M062X_def2-TZVP_sp.out
│   │   ├── SSdepr4F_M062X_def2-TZVP_sp.sdf
│   │   ├── SSdepr5F_B2PLYPD3_def2-TZVP_sp.out
│   │   ├── SSdepr5F_B3LYP_freq.out
│   │   ├── SSdepr5F_M062X_def2-TZVP_sp.out
│   │   └── SSdepr5F_M062X_def2-TZVP_sp.sdf
│   ├── IV
│   │   ├── ProdDisocA_B2PLYPD3_def2-TZVP_sp.out
│   │   ├── ProdDisocA_B3LYP_freq.out
│   │   ├── ProdDisocA_M062X_def2-TZVP_sp.out
│   │   └── ProdDisocA_M062X_def2-TZVP_sp.sdf
│   ├── MePy
│   │   ├── MePyA_B2PLYPD3_def2-TZVP_sp.out
│   │   ├── MePyA_B3LYP_freq.out
│   │   ├── MePyA_M062X_def2-TZVP_sp.out
│   │   └── MePyA_M062X_def2-TZVP_sp.sdf
│   ├── MePyH_PA
│   │   ├── PAMePyA_B2PLYPD3_def2-TZVP_sp.out
│   │   ├── PAMePyA_B3LYP_freq.out
│   │   ├── PAMePyA_M062X_def2-TZVP_sp.out
│   │   └── PAMePyA_M062X_def2-TZVP_sp.sdf
│   ├── PAH
│   │   ├── PAA_B2PLYPD3_def2-TZVP_sp.out
│   │   ├── PAA_B3LYP_freq.out
│   │   ├── PAA_M062X_def2-TZVP_sp.out
│   │   └── PAA_M062X_def2-TZVP_sp.sdf
│   └── Rad
│       ├── RadA_B2PLYPD3_def2-TZVP_sp.out
│       ├── RadA_B3LYP_freq.out
│       ├── RadA_M062X_def2-TZVP_sp.out
│       └── RadA_M062X_def2-TZVP_sp.sdf
└── readme.txt
```

14 directories, 118 files

References

1. List, B.; Klussmann, M.; Ratjen, L.; Hoffmann, S.; Wakchaure, V.; Goddard, R., Synthesis of TRIP and Analysis of Phosphate Salt Impurities. *Synlett* **2010,** *2010* (14), 2189-2192.
2. Neel, A. J.; Milo, A.; Sigman, M. S.; Toste, F. D., Enantiodivergent Fluorination of Allylic Alcohols: Data Set Design Reveals Structural Interplay between Achiral Directing Group and Chiral Anion. *J Am Chem Soc* **2016,** *138* (11), 3863-75.
3. Mandai, H.; Murota, K.; Mitsudo, K.; Suga, S., Kinetic Resolution of Secondary Alcohols by the Combination of a Chiral Brønsted Acid, DABCO, and Acetyl Chloride. *Organic Letters* **2012,** *14*, 3486 - 3489.
4. Gribkov, D. V.; Hultzsch, K. C.; Hampel, F., Synthesis and characterization of new biphenolate and binaphtholate rare-Earth-metal amido complexes: catalysts for asymmetric olefin hydroamination/cyclization. *Chemistry* **2003,** *9* (19), 4796-810.
5. Rauniyar, V.; Wang, Z. J.; Burks, H. E.; Toste, F. D., Enantioselective synthesis of highly substituted furans by a copper(II)-catalyzed cycloisomerization-indole addition reaction. *J Am Chem Soc* **2011,** *133* (22), 8486-9.
6. Czaplik, W. M.; Mayer, M.; Jacobi von Wangelin, A., Domino iron catalysis: direct aryl-alkyl cross-coupling. *Angew Chem Int Ed Engl* **2009,** *48* (3), 607-10.
7. Zhang, P.; Huang, D.; Newhouse, T. R., Aryl-Nickel-Catalyzed Benzylic Dehydrogenation of Electron-Deficient Heteroarenes. *J Am Chem Soc* **2020,** *142* (4), 1757-1762.
8. Kempf, D. J.; Codacovi, L.; Wang, X. C.; Kohlbrenner, W. E.; Wideburg, N. E.; Saldivar, A.; Vasavanonda, S.; Marsh, K. C.; Bryant, P.; Sham, H. L.; Green, B. E.; Betebenner, D. A. E., J. and Norbeck, D. W., Symmetry-Based Inhibitors of HIV Protease. Structure-Activity Studies of Acylated 2,4-Diamino-l,5-diphenyl-3-hydroxypentane and 2,5-Diamino-l,6-diphenylhexane-3,4-diol. *J Med Chem* **1993,** *36*, 320-330.
9. Min, X. L.; Sun, C.; He, Y., Synthesis of 1-Amino-2 H-quinolizin-2-one Scaffolds by Tandem Silver Catalysis. *Org Lett* **2019,** *21* (3), 724-728.
10. Schneider, M. J.; Ungemach, F. S.; Broquist, H. P.; Harris, T. M., Biosynthesis of swainsonine in Rhizoctonia leguminicola. Epimerization at the ring fusion. *J Am Chem Soc* **1982,** *104*, 6863-6864.
11. Marshall, L. J. R., M. D. Slawin, A. M. Z. and Walton. J. C. , Effect of Chain Length on Radical to Carbanion Cyclo-Coupling of Bromoaryl Alkyl-Linked Oxazolines: 1,3- reneotropic Migration of Oxazolines. *The Journal of Organic Chemistry* **2007,** *72*, 898-911.
12. Liang, H.; Ciufolini, M. A., Oxidative spirocyclization of phenolic sulfonamides: scope and applications. *Chemistry* **2010,** *16* (44), 13262-70.
13. Falk, E.; Gasser, V. C. M.; Morandi, B., Synthesis of N-Alkyl Anilines from Arenes via Iron-Promoted Aromatic C-H Amination. *Org Lett* **2021,** *23* (4), 1422-1426.
14. Andersen, C.; Ferey, V.; Daumas, M.; Bernardelli, P.; Guerinot, A.; Cossy, J., Introduction of Cyclopropyl and Cyclobutyl Ring on Alkyl Iodides through Cobalt-Catalyzed Cross-Coupling. *Org Lett* **2019,** *21* (7), 2285-2289.
15. Zhang, L.; Liu, Z. Q., Molecular Oxygen-Mediated Minisci-Type Radical Alkylation of Heteroarenes with Boronic Acids. *Org Lett* **2017,** *19* (24), 6594-6597.
16. de Pedro Beato, E.; Spinnato, D.; Zhou, W.; Melchiorre, P., A General Organocatalytic System for Electron Donor-Acceptor Complex Photoactivation and Its Use in Radical Processes. *J Am Chem Soc* **2021,** *143* (31), 12304-12314.
17. Dale, J. A.; Mosher, H. S., Nuclear magnetic resonance enantiomer regents. Configurational correlations via nuclear magnetic resonance chemical shifts of diastereomeric mandelate, O-methylmandelate, and .alpha.-methoxy-.alpha.-trifluoromethylphenylacetate (MTPA) esters. *J Am Chem Soc* **1973,** *2*, 512-519.
18. Hoye, T. R.; Jeffrey, C. S.; Shao, F., Mosher ester analysis for the determination of absolute configuration of stereogenic (chiral) carbinol carbons. *Nat Protoc* **2007,** *2* (10), 2451-8.
19. Cahn, R. S.; Ingold, C. K.; Prelog, V., The Specification of Asymmetric Configuation in Organic Chemistry *Experientia* **1956,** *12*, 81-94.

NMR Spectra

^{1}H NMR (500 MHz, $CDCl_3$) 2-(2-((triethylsilyl)oxy)ethyl)pyridine

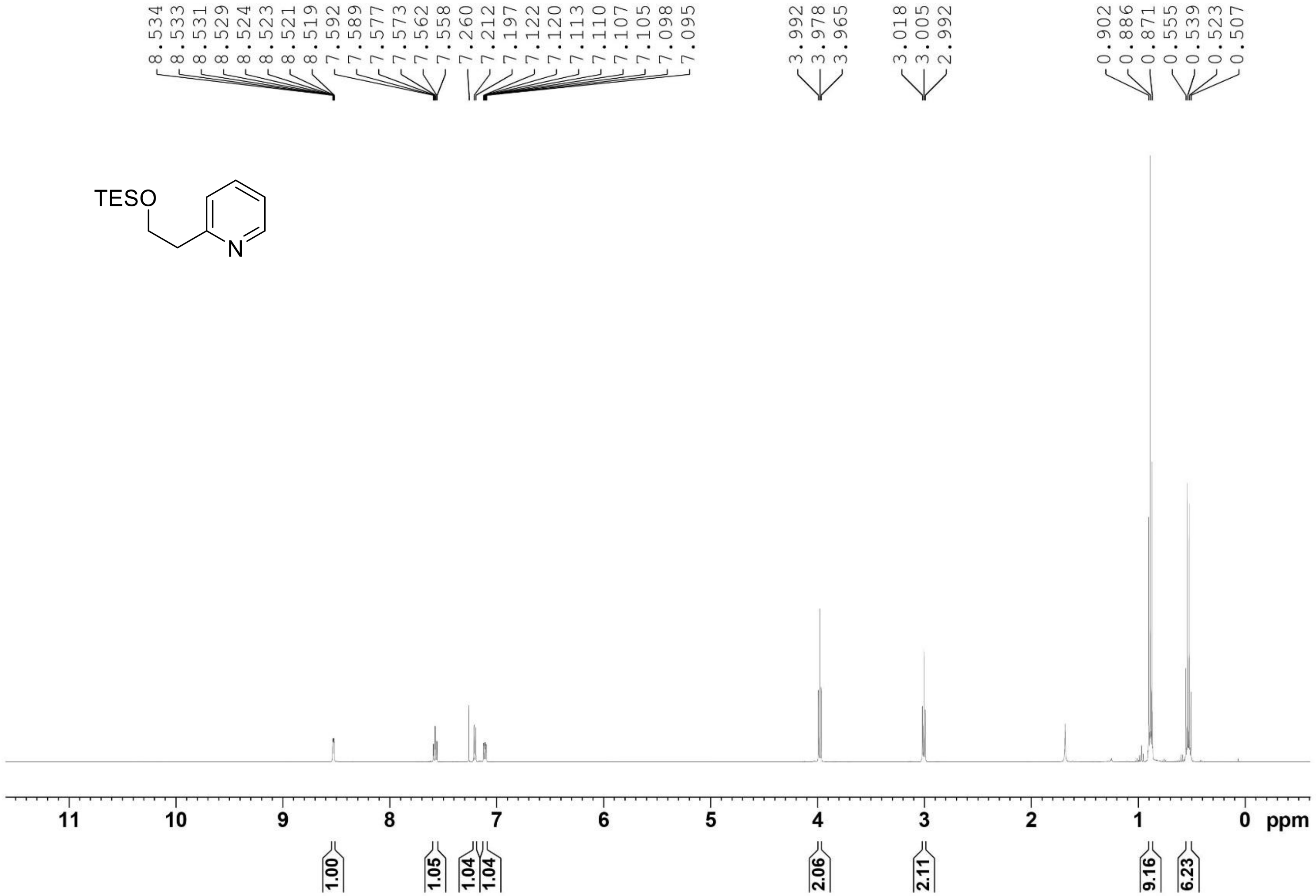

^{13}C NMR (126 MHz, $CDCl_3$) 2-(2-((triethylsilyl)oxy)ethyl)pyridine

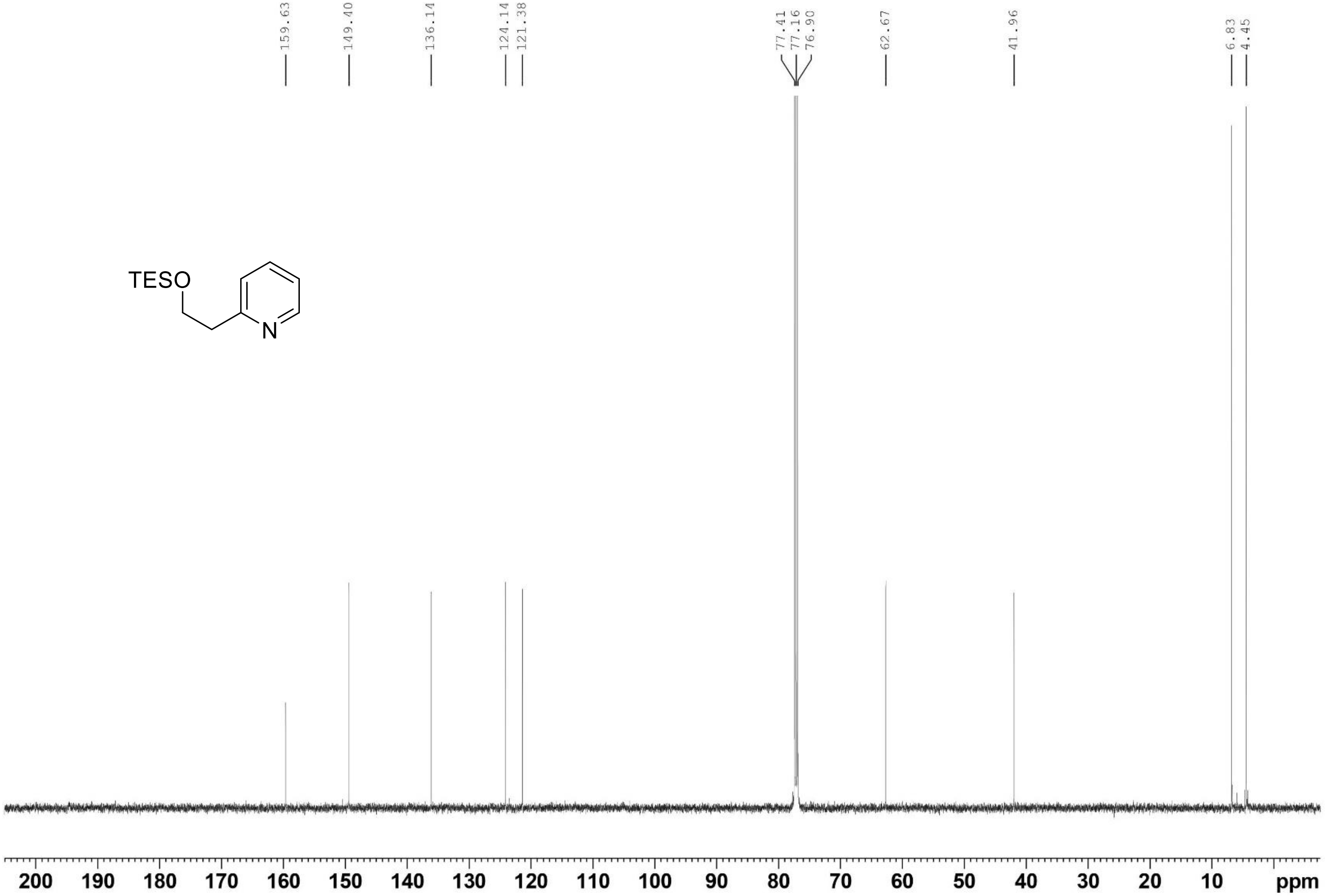

^{1}H NMR (500 MHz, $CDCl_3$) *tert*-butyl ((5-methylpyridin-2-yl)methyl)carbamate

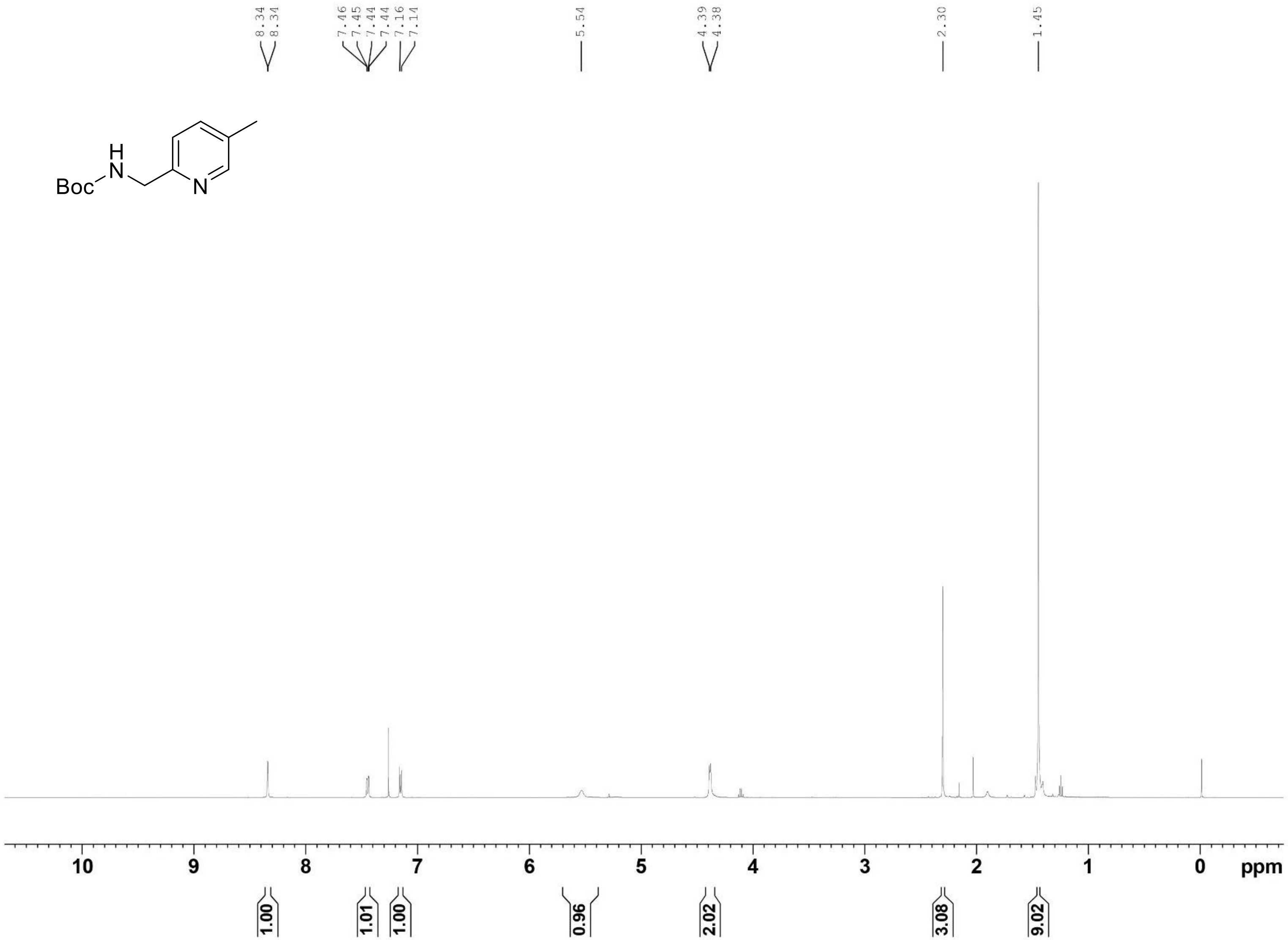

^{13}C NMR (126 MHz, $CDCl_3$) *tert*-butyl ((5-methylpyridin-2-yl)methyl)carbamate

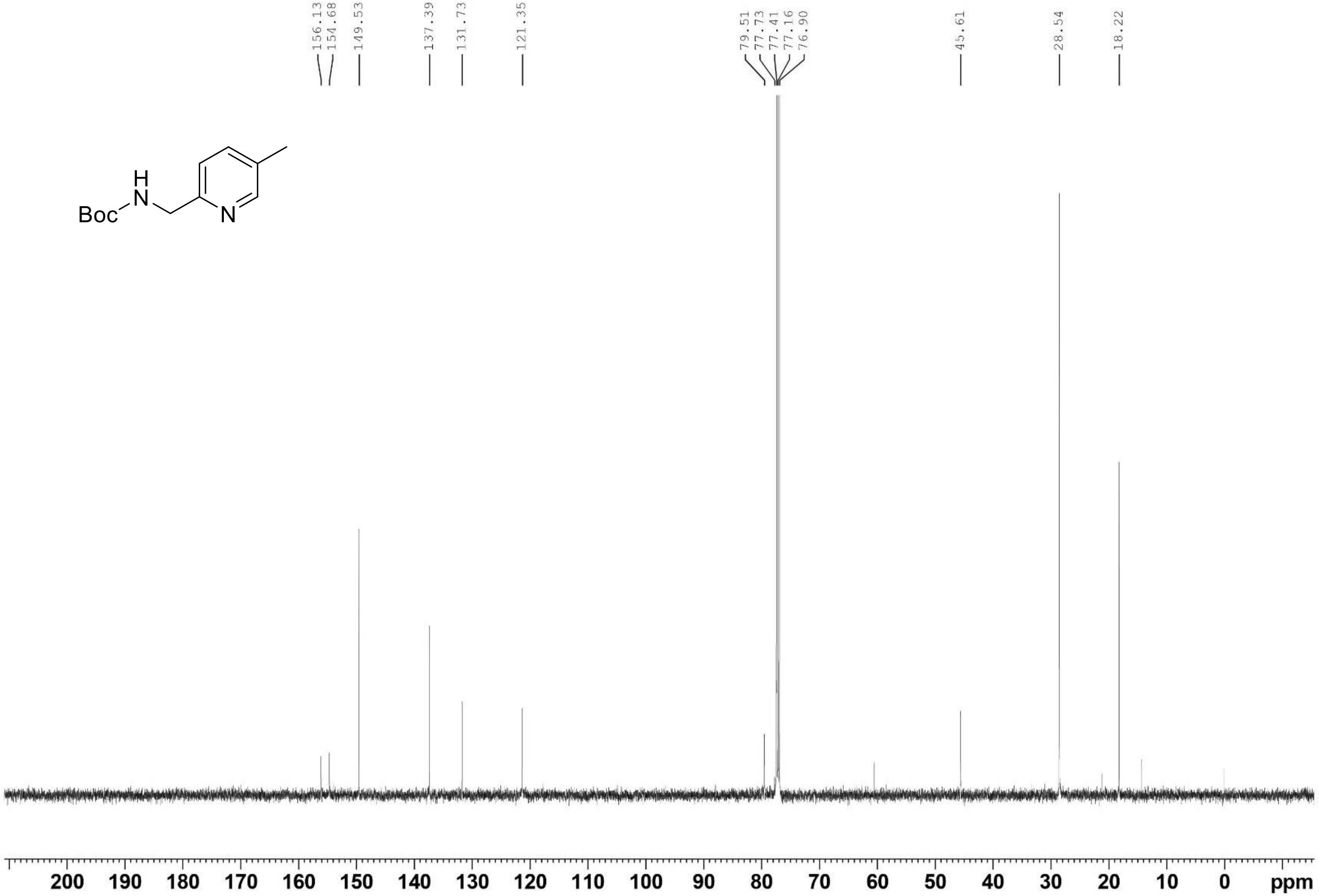

^{1}H NMR (500 MHz, CDCl$_3$) (*R*)-1-(6-pentylpyridin-2-yl)-3-phenylpropan-1-ol (2a)

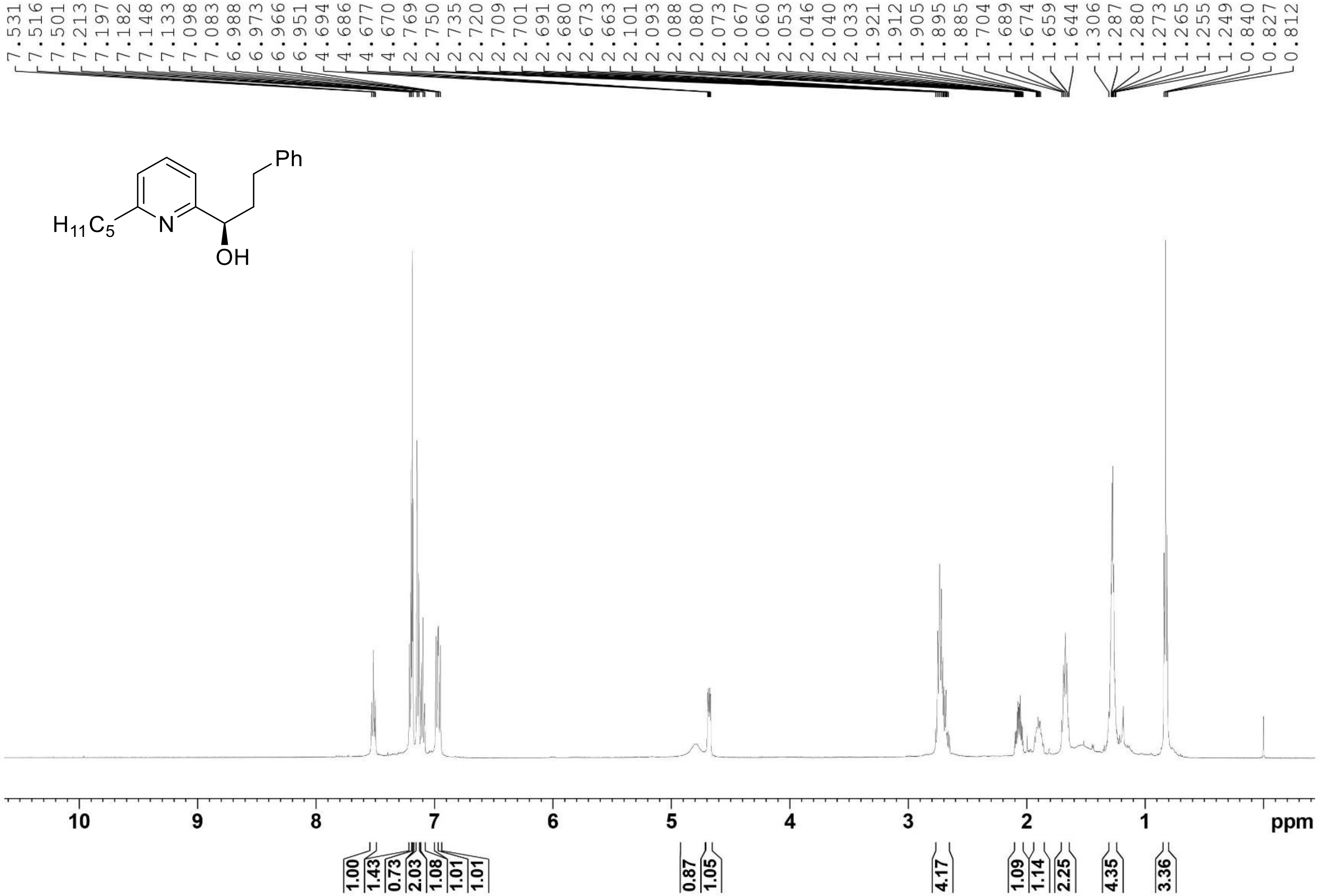

^{13}C NMR (101 MHz, $CDCl_3$) (*R*)-1-(6-pentylpyridin-2-yl)-3-phenylpropan-1-ol (2a)

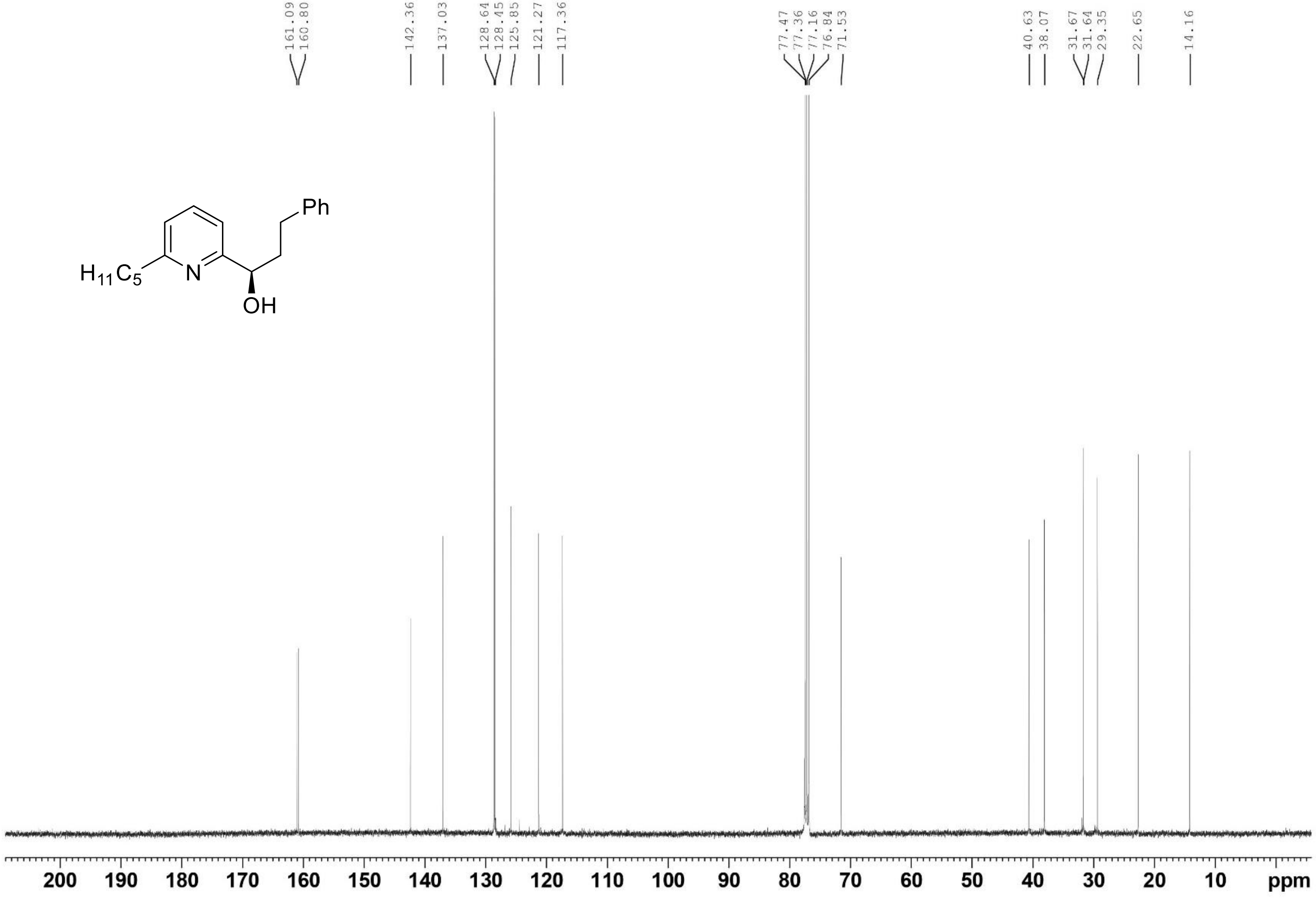

^{1}H NMR (500 MHz, $CDCl_3$) (*R*)-1-(6-methylpyridin-2-yl)-3-phenylpropan-1-ol (2b)

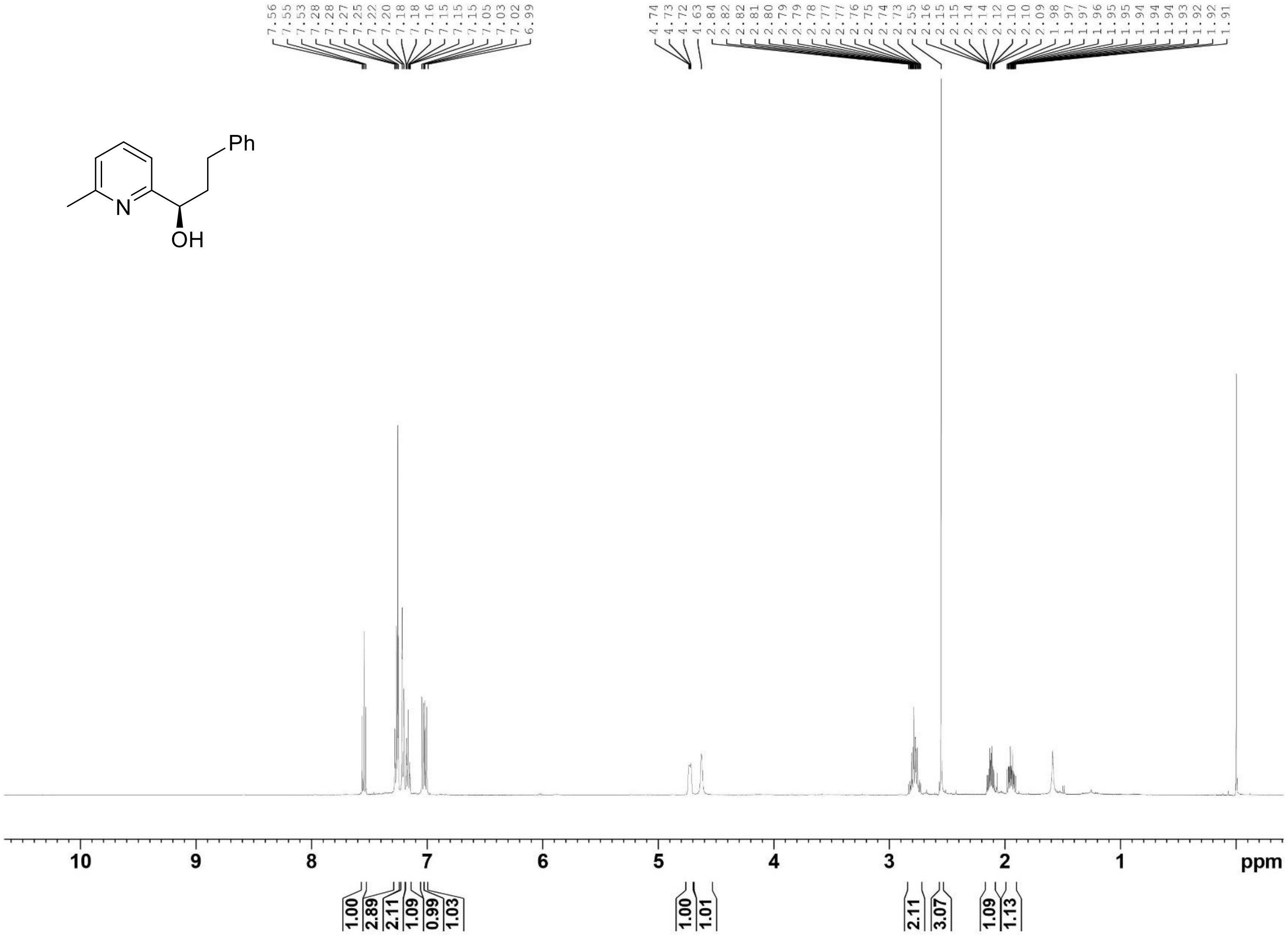

^{13}C NMR (126 MHz, CDCl$_3$) (*R*)-1-(6-methylpyridin-2-yl)-3-phenylpropan-1-ol (2b)

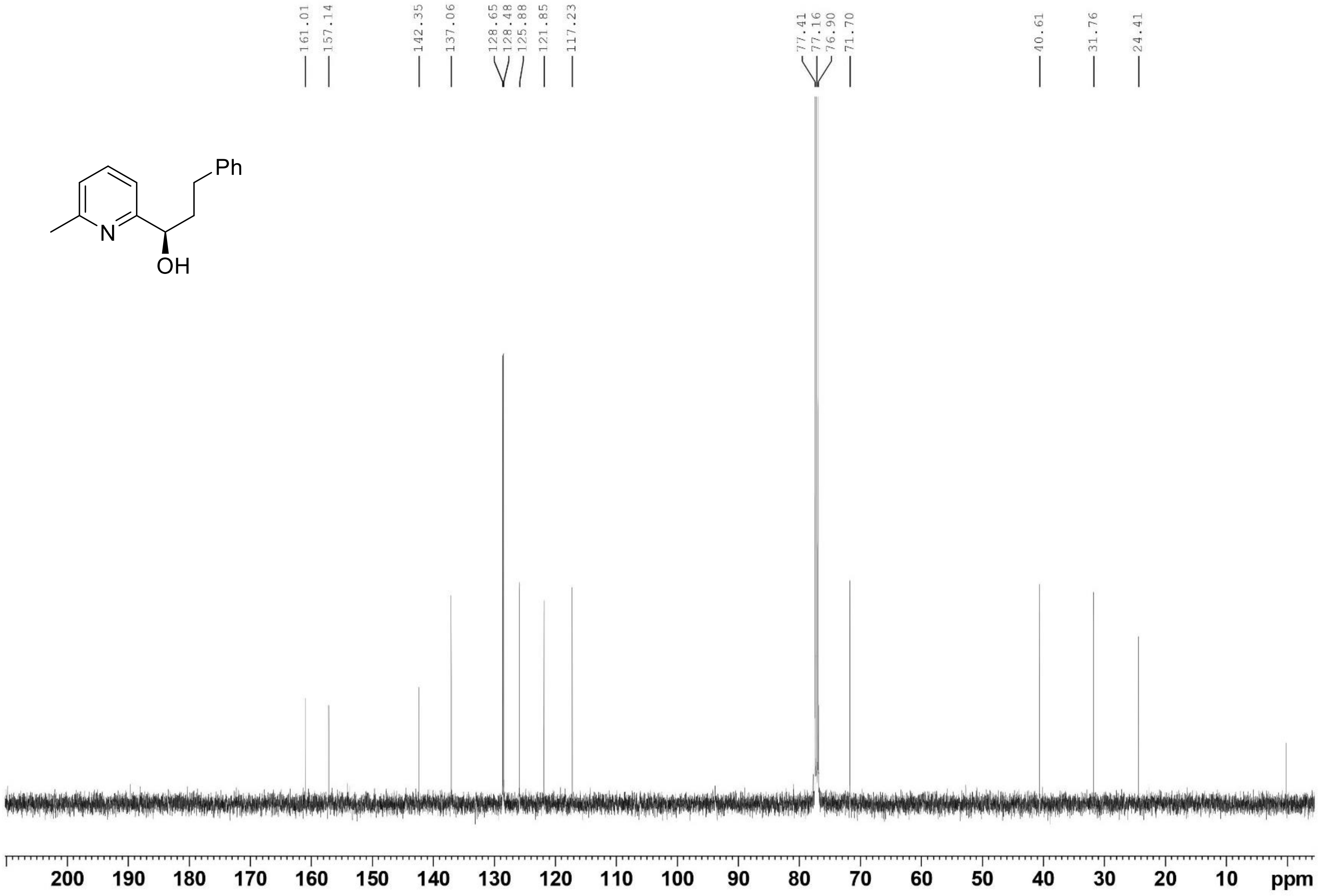

^{1}H NMR (400 MHz, CDCl$_3$) (*R*)-1-(6-isopropylpyridin-2-yl)-3-phenylpropan-1-ol (2c)

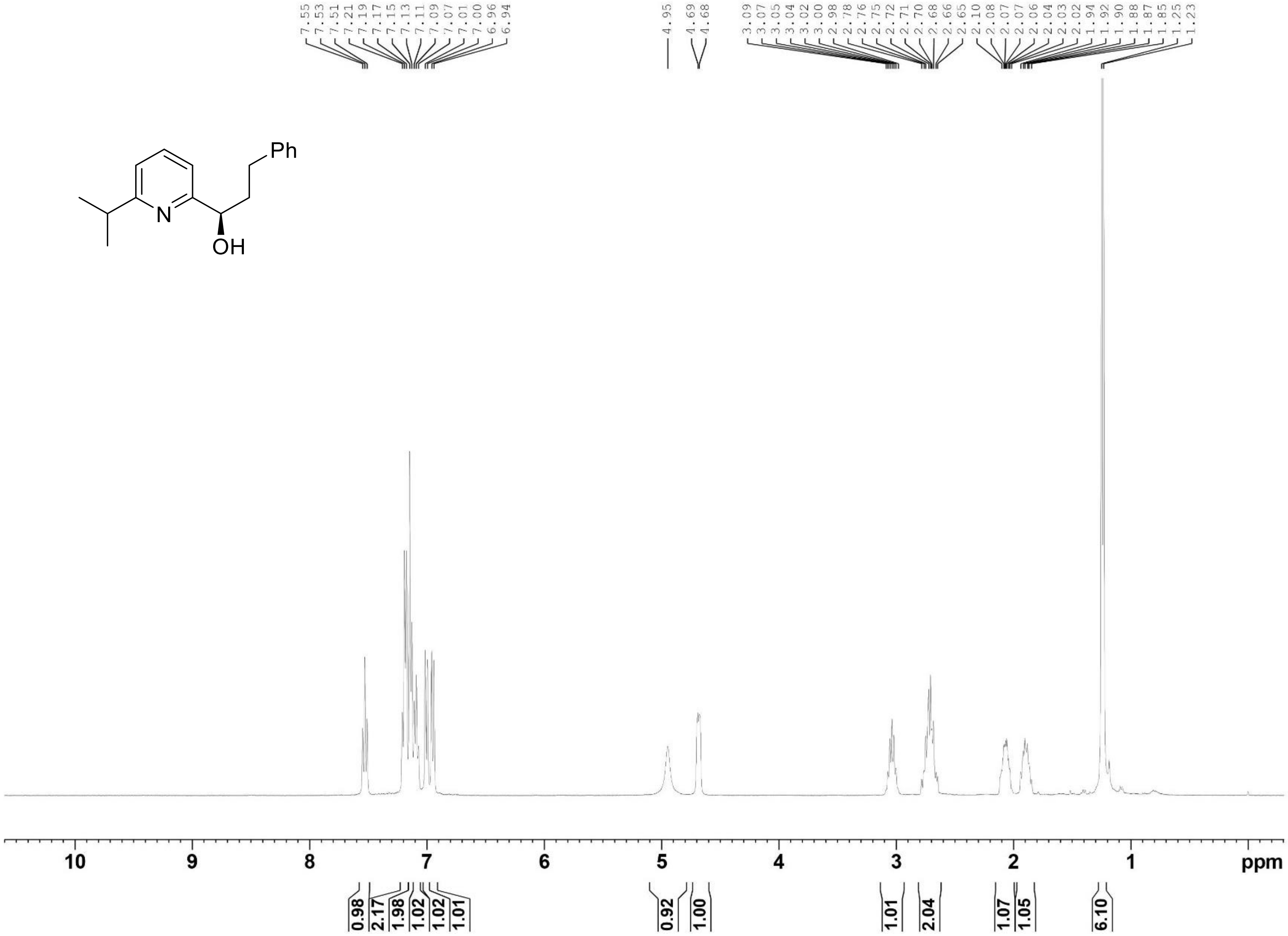

^{13}C NMR (101 MHz, $CDCl_3$) (*R*)-1-(6-isopropylpyridin-2-yl)-3-phenylpropan-1-ol (2c)

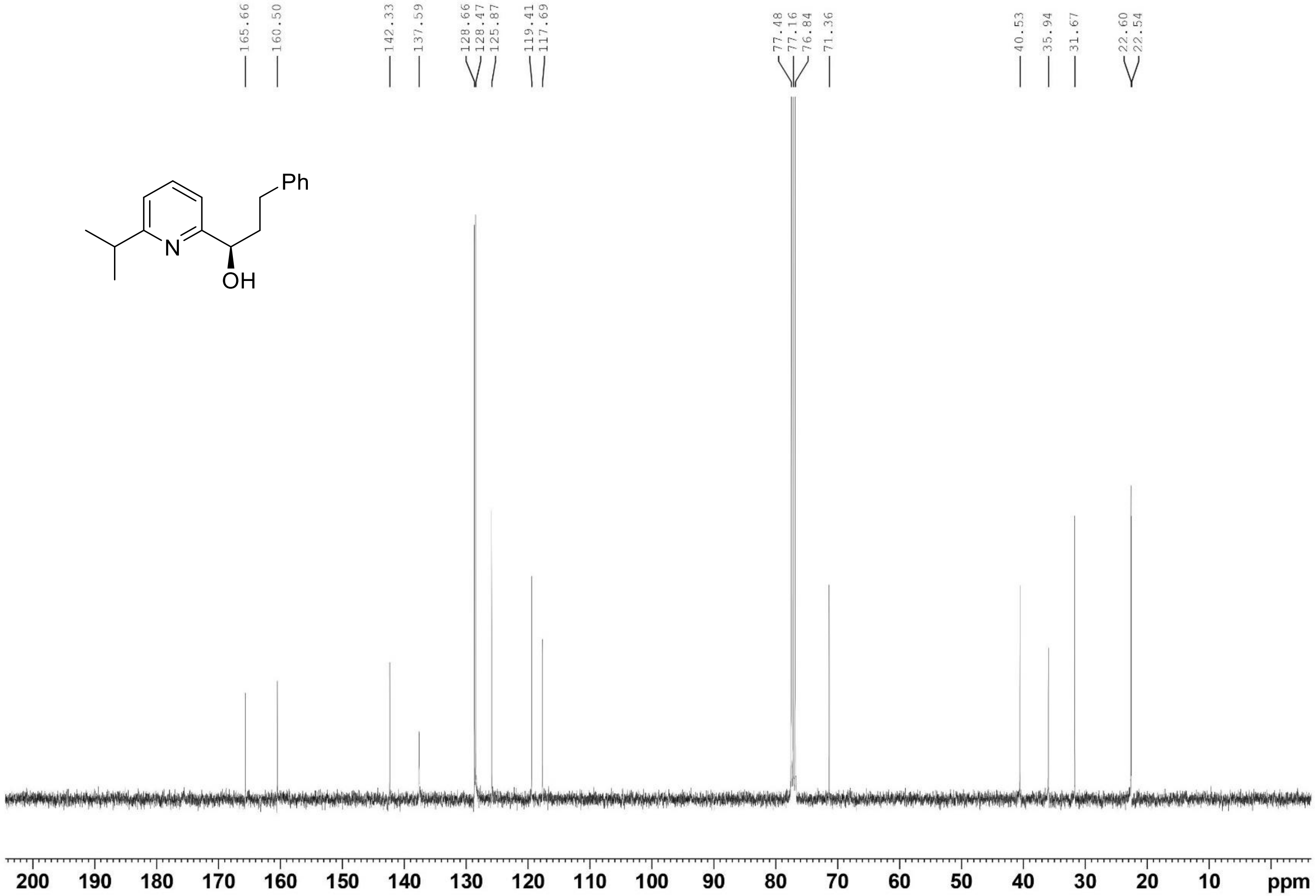

^{1}H NMR (400 MHz, $CDCl_3$) (*R*)-1-(6-cyclohexylpyridin-2-yl)-3-phenylpropan-1-ol (2d)

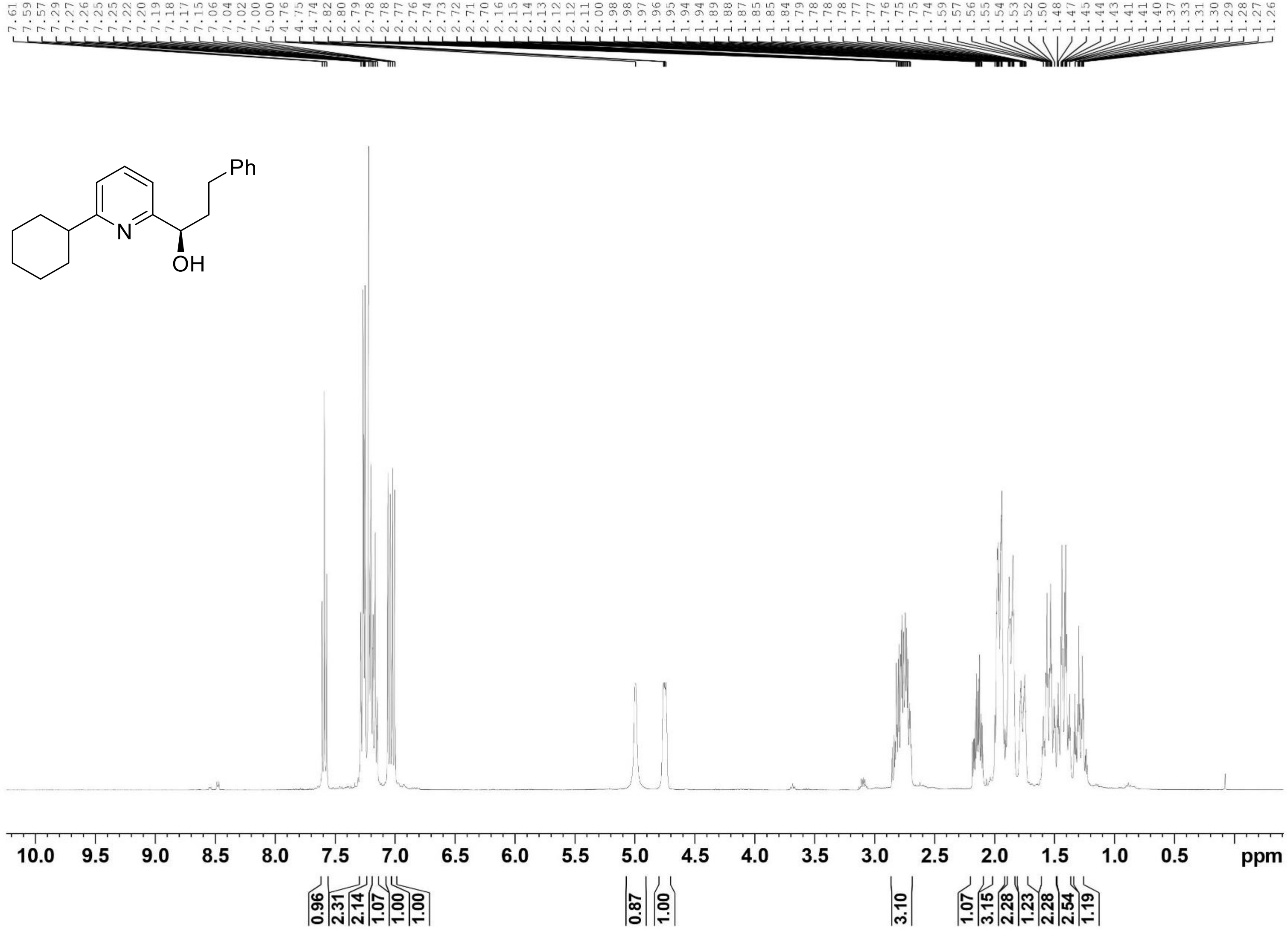

^{13}C NMR (101 MHz, CDCl$_3$) (*R*)-1-(6-cyclohexylpyridin-2-yl)-3-phenylpropan-1-ol (2d)

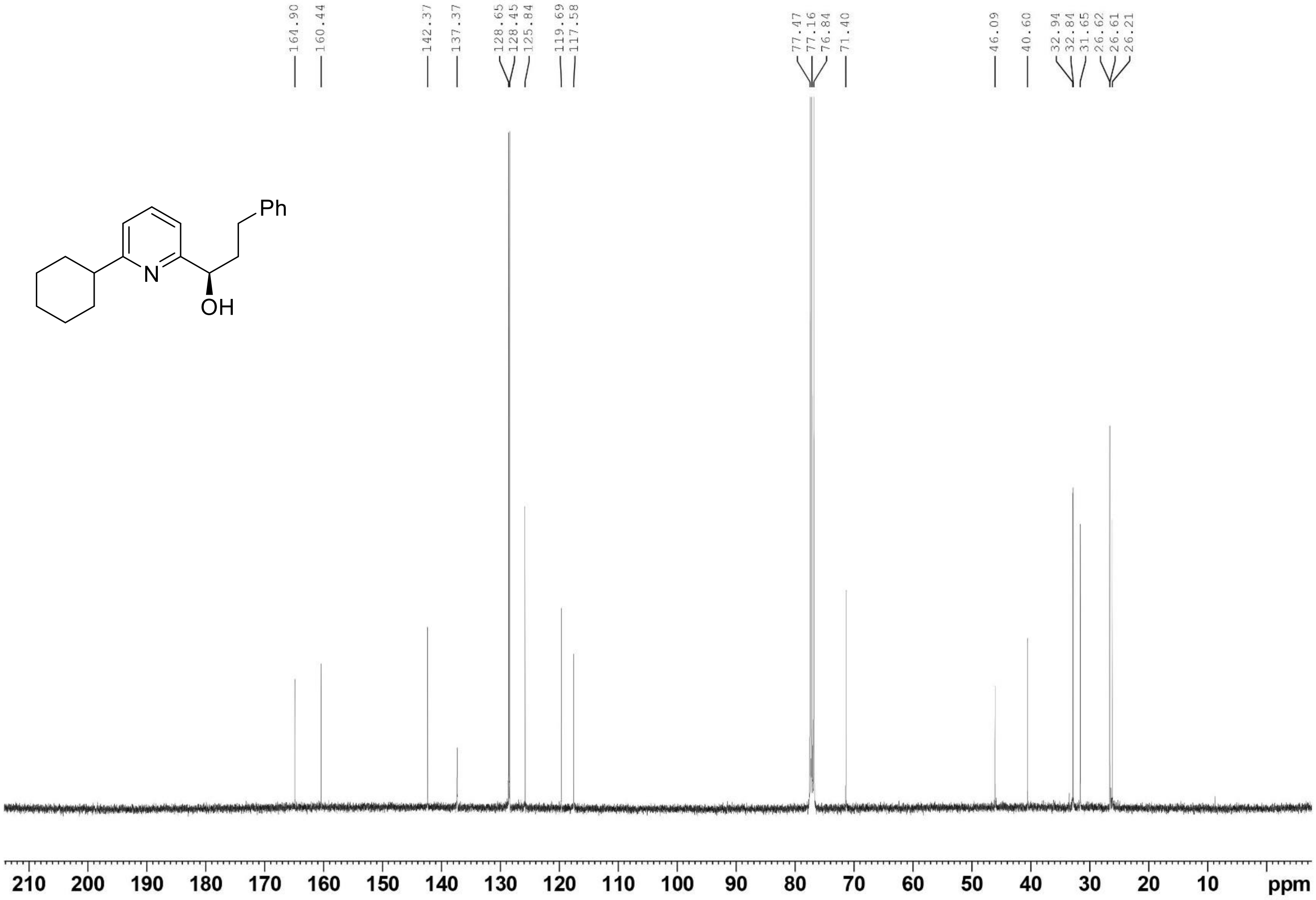

^{1}H NMR (400 MHz, $CDCl_3$) (*R*)-1-(6-(cyclohexylmethyl)pyridin-2-yl)-3-phenylpropan-1-ol (Xe)

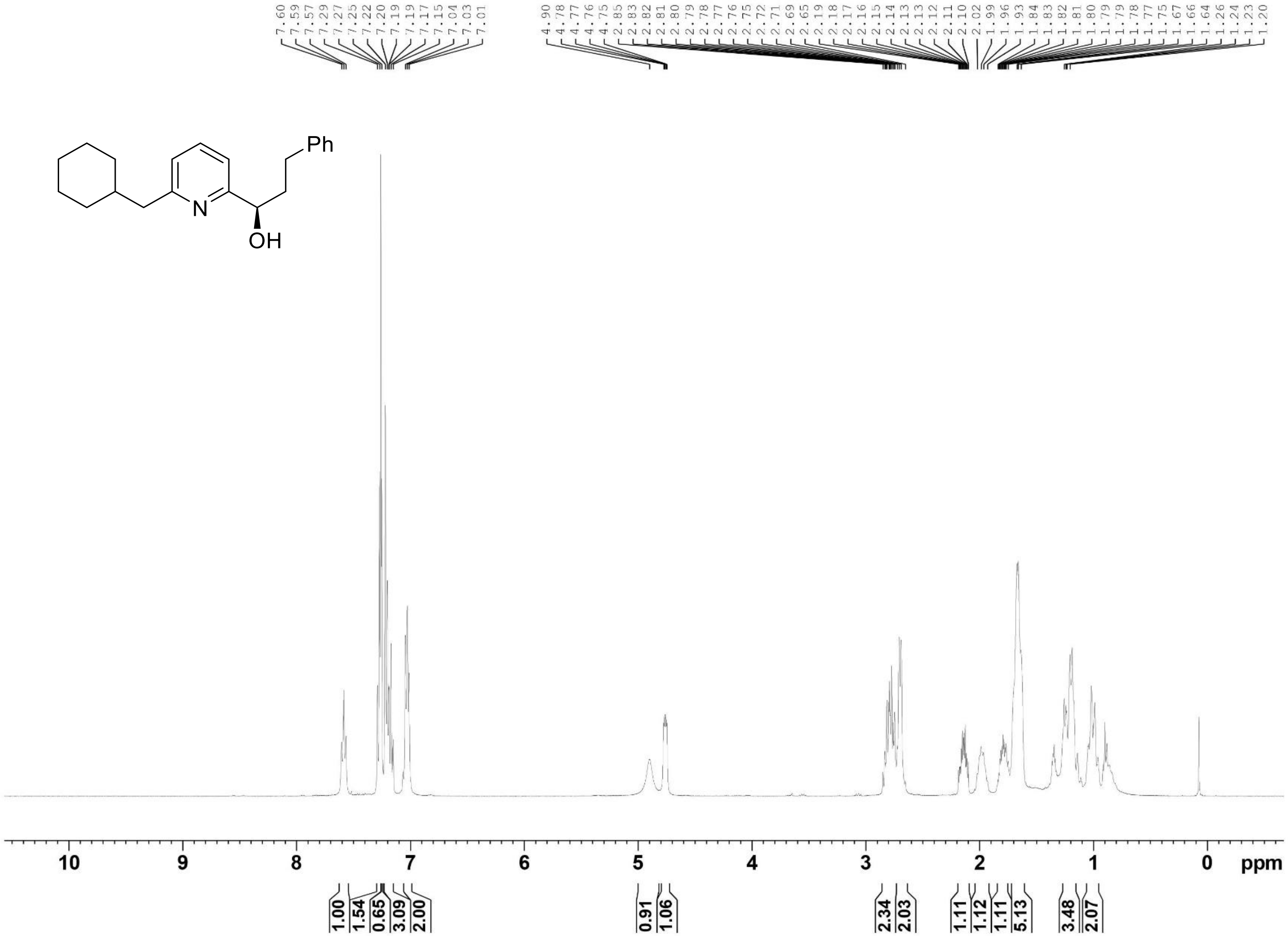

^{13}C NMR (126 MHz, CDCl$_3$) (*R*)-1-(6-(cyclohexylmethyl)pyridin-2-yl)-3-phenylpropan-1-ol (Xe)

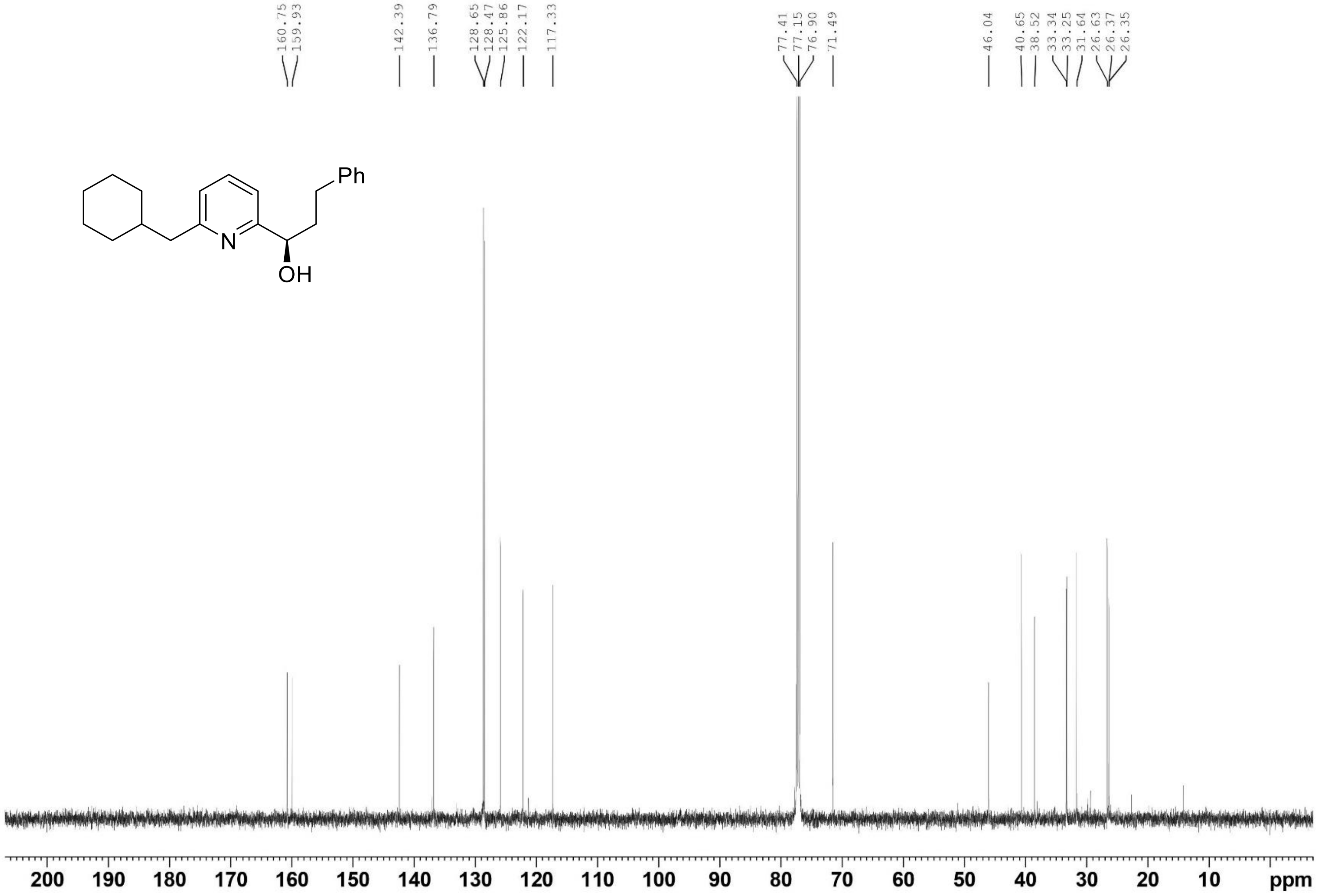

^{1}H NMR (400 MHz, $CDCl_3$) *tert*-butyl (*R*)-(2-(6-(1-hydroxy-3-phenylpropyl)pyridin-2-yl)ethyl)carbamate (2f)

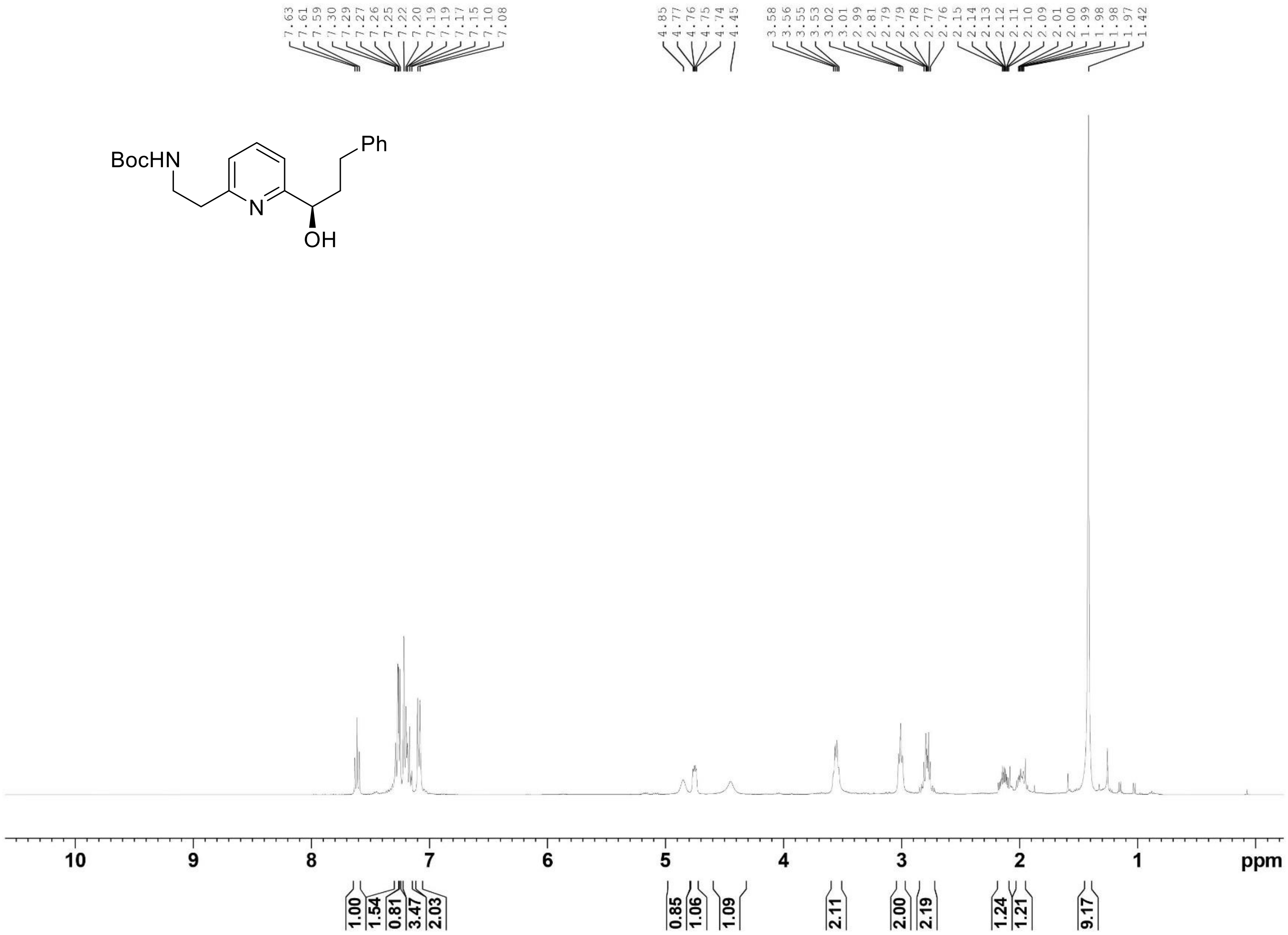

^{13}C NMR (101 MHz, $CDCl_3$) *tert*-butyl (*R*)-(2-(6-(1-hydroxy-3-phenylpropyl)pyridin-2-yl)ethyl)carbamate (2f)

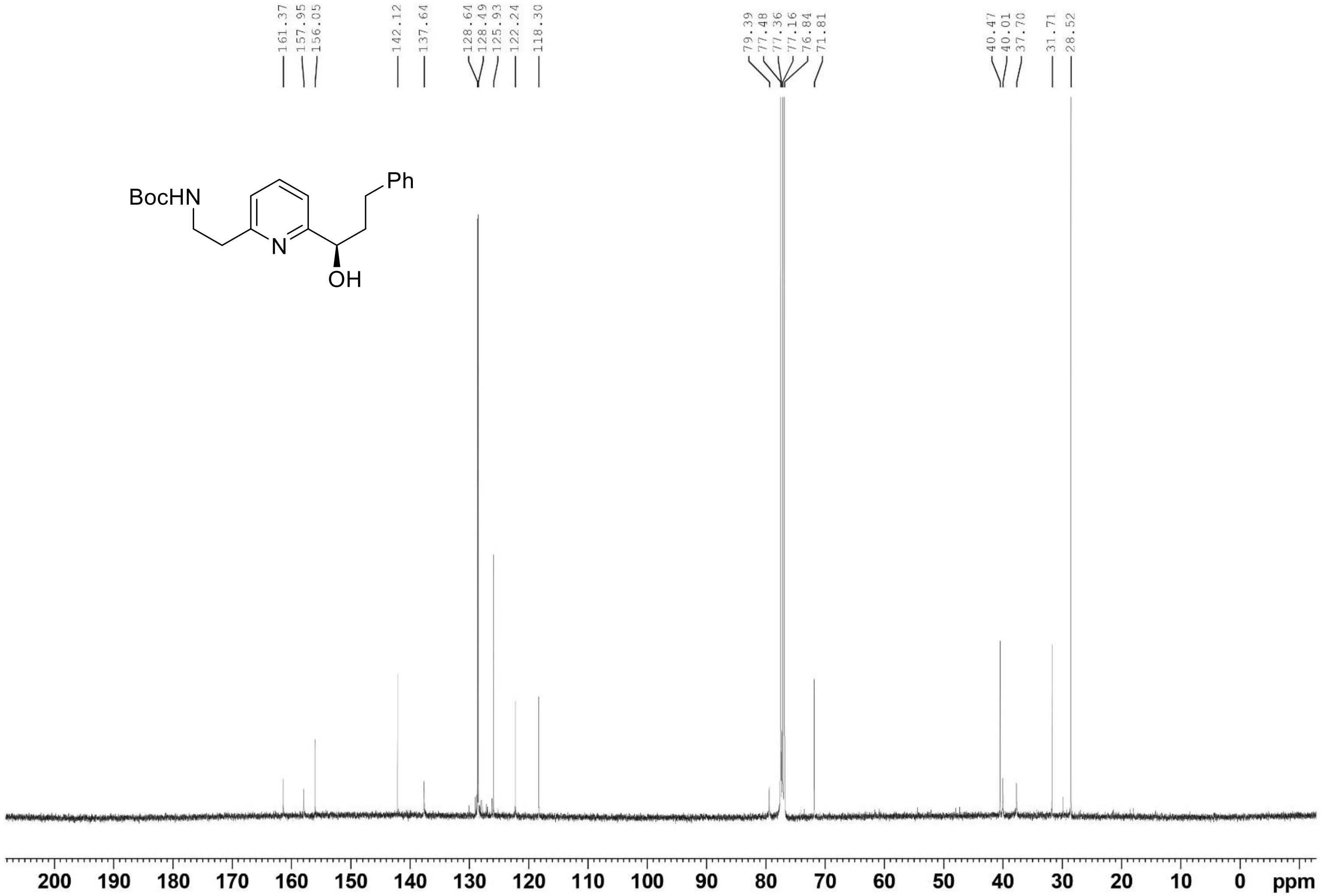

^{1}H NMR (400 MHz, $CDCl_3$) (*R*)-1-(6-(2-hydroxyethyl)pyridin-2-yl)-3-phenylpropan-1-ol (2ga)

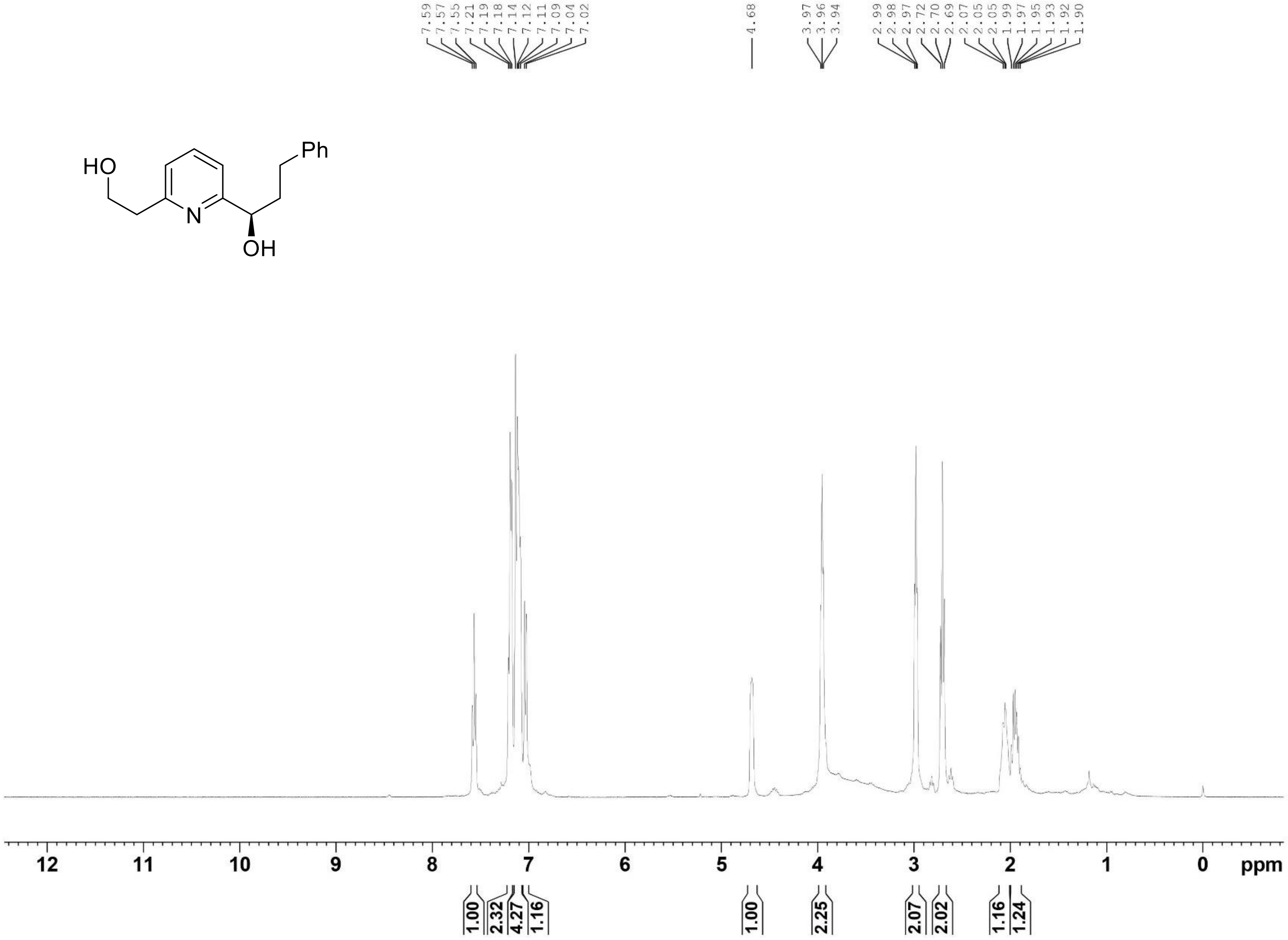

^{13}C NMR (101 MHz, $CDCl_3$) (*R*)-1-(6-(2-hydroxyethyl)pyridin-2-yl)-3-phenylpropan-1-ol (2ga)

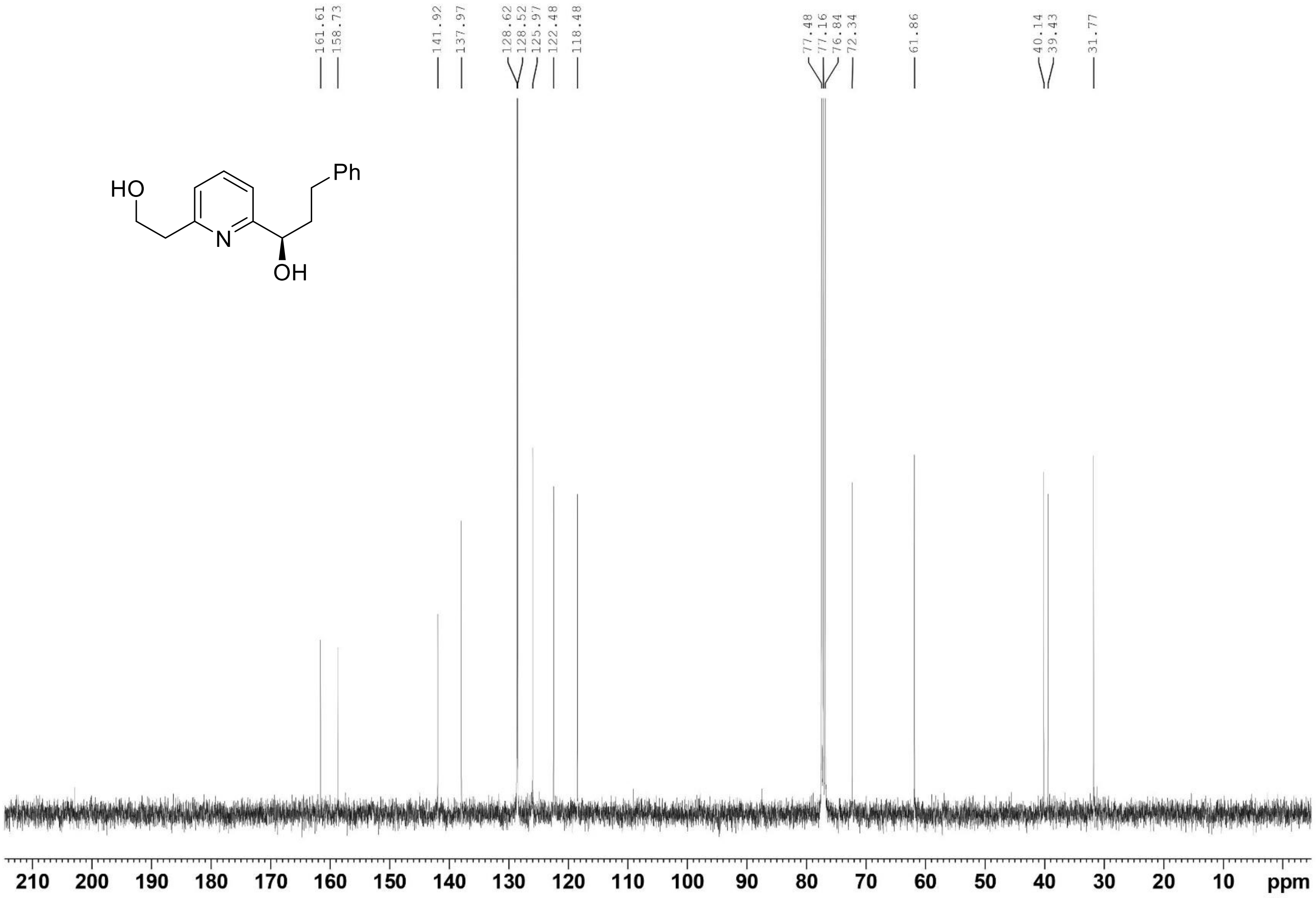

^{1}H NMR (500 MHz, $CDCl_3$) (*R*)-3-phenyl-1-(6-(3-phenylpropyl)pyridin-2-yl)propan-1-ol (2h)

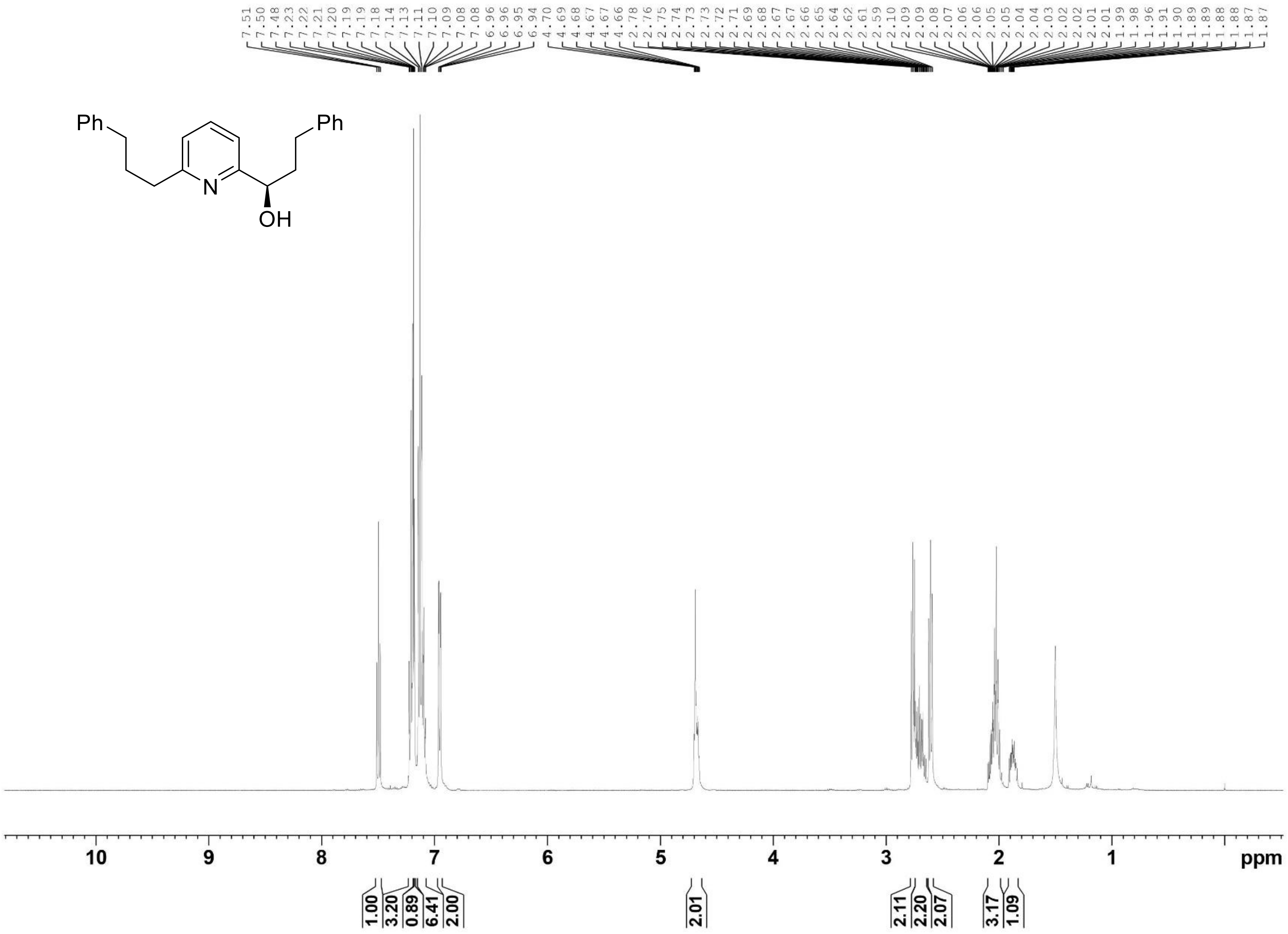

^{13}C NMR (126 MHz, $CDCl_3$) (*R*)-3-phenyl-1-(6-(3-phenylpropyl)pyridin-2-yl)propan-1-ol (2h)

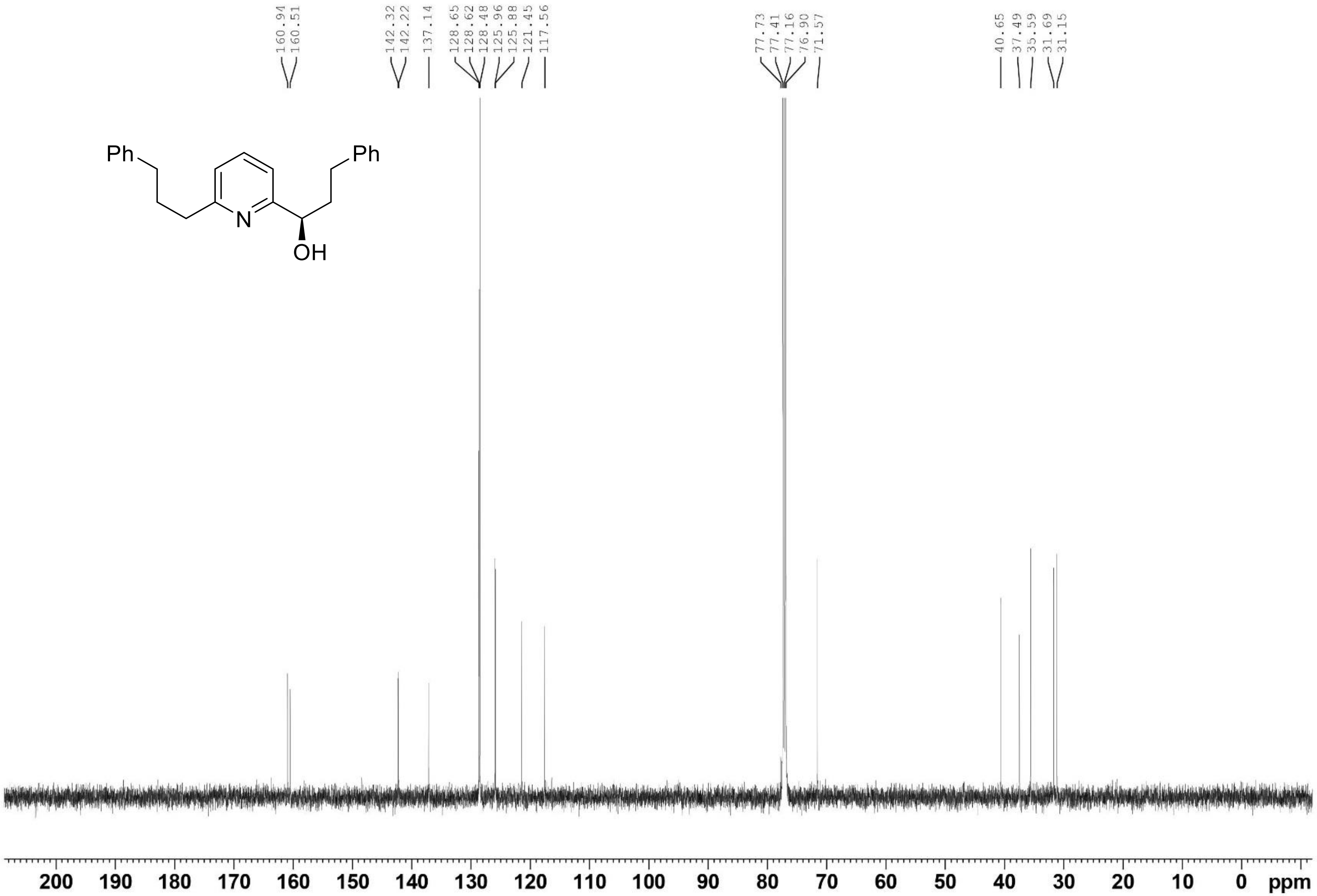

^{1}H NMR (400 MHz, CDCl$_3$) (*R*)-1-(5,6-dimethylpyridin-2-yl)-3-phenylpropan-1-ol (2i)

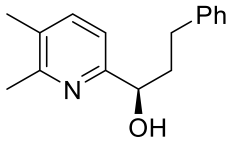

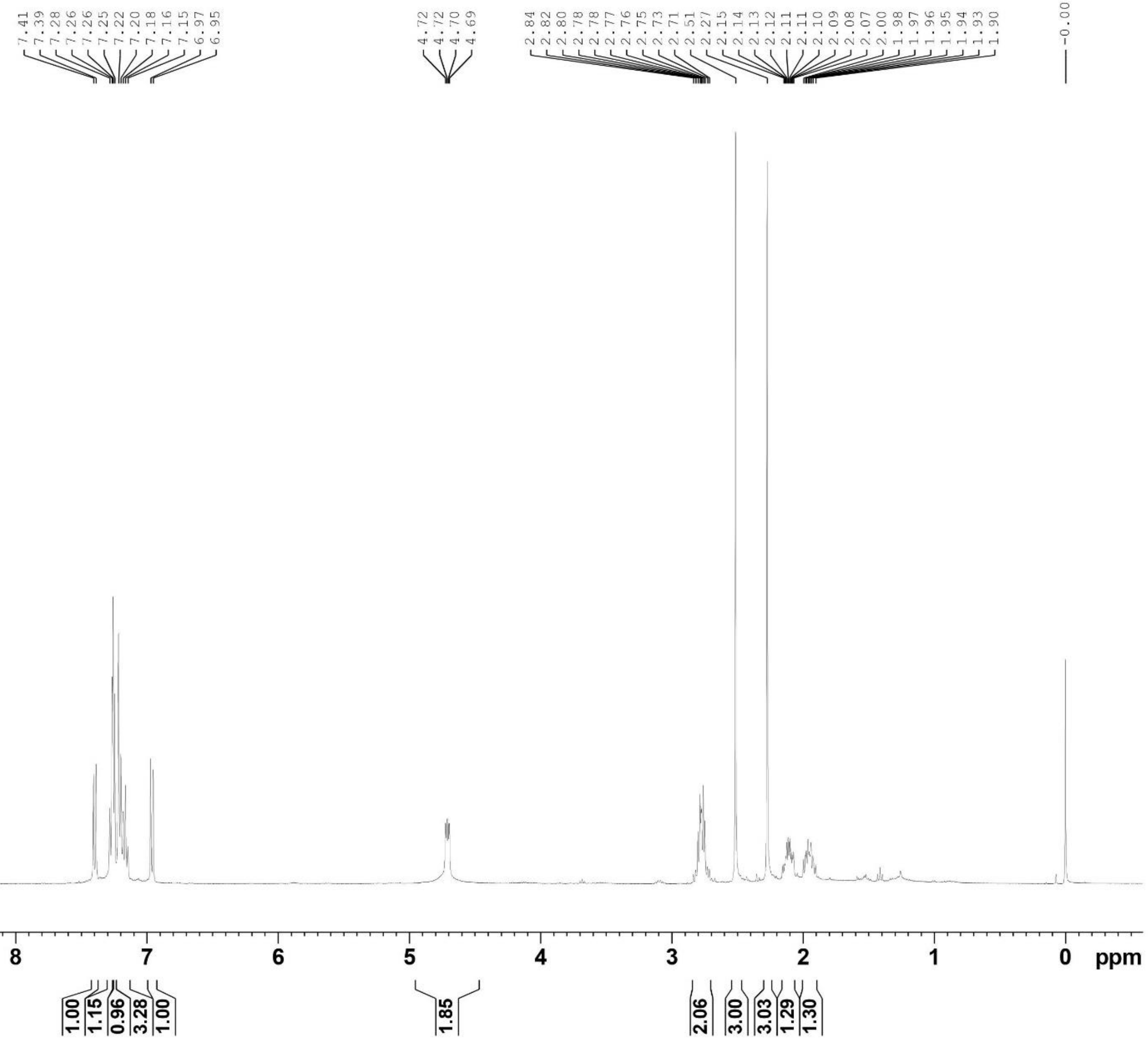

^{13}C NMR (101 MHz, $CDCl_3$) (*R*)-1-(5,6-dimethylpyridin-2-yl)-3-phenylpropan-1-ol (2i)

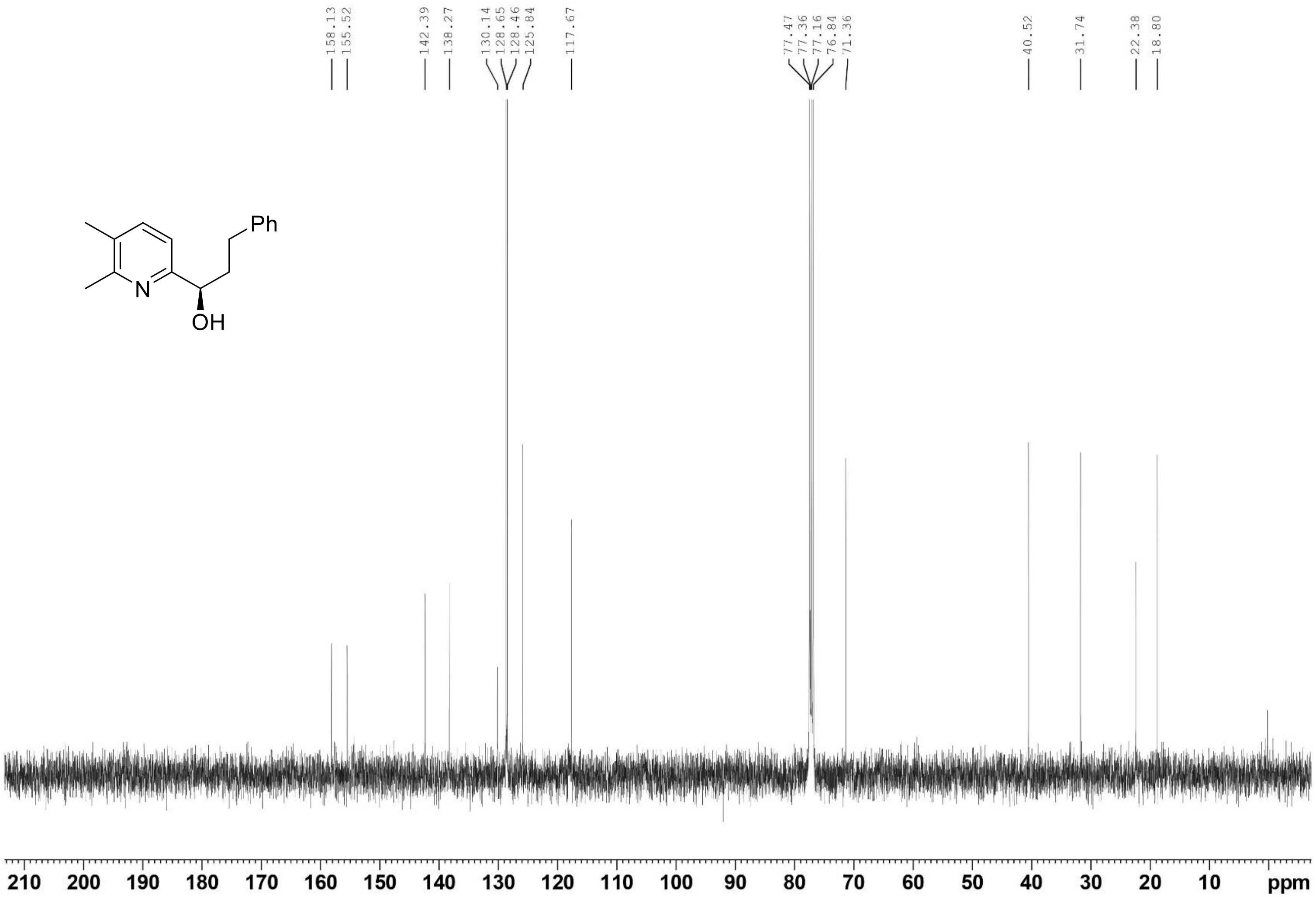

^{1}H NMR (400 MHz, $CDCl_3$) (*R*)-1-(6,7-dihydro-5H-cyclopenta-pyridin-2-yl)-3-phenylpropan-1-ol (2j)

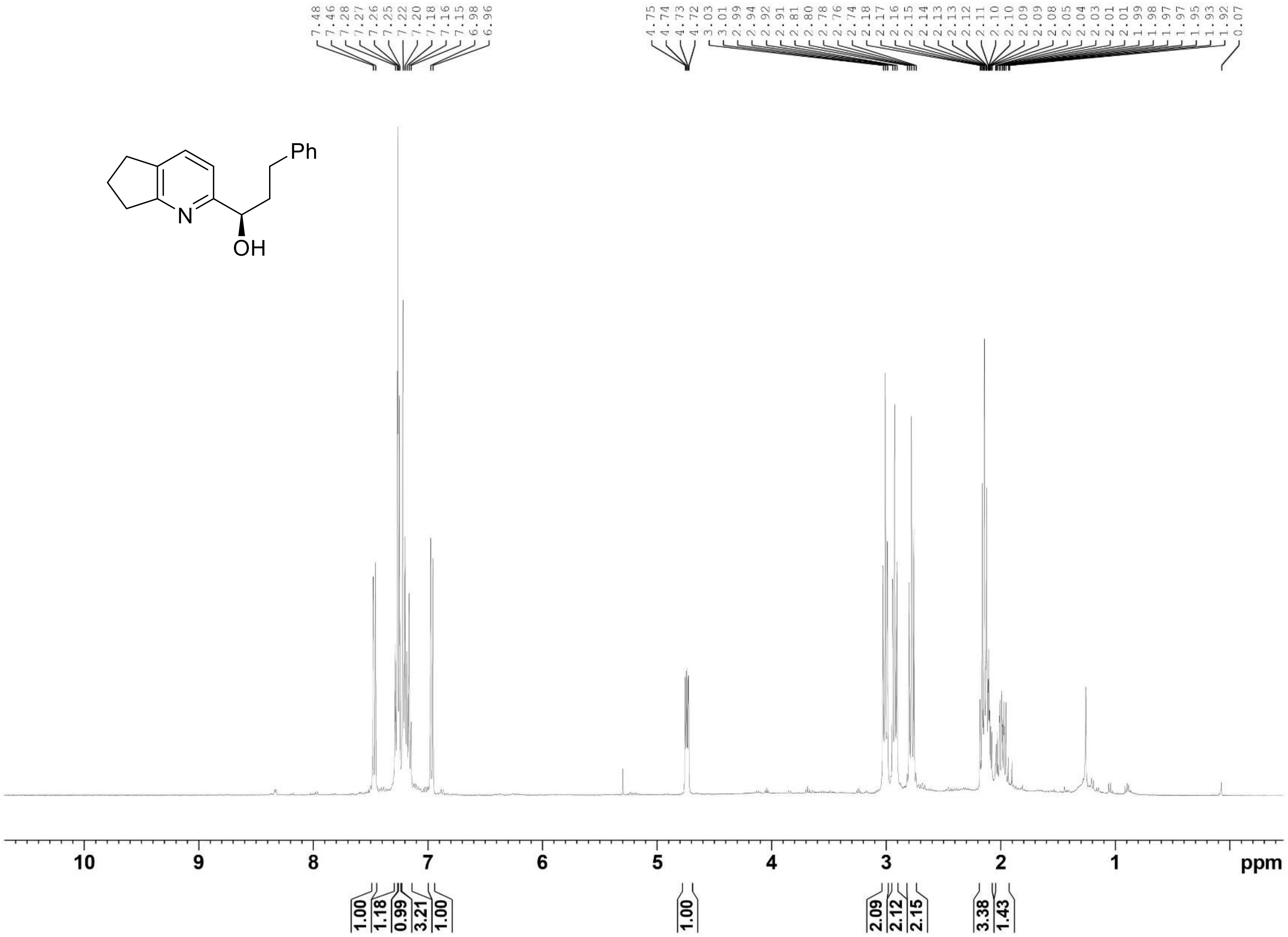

^{13}C NMR (101 MHz, CDCl$_3$) (*R*)-1-(6,7-dihydro-5H-cyclopenta-pyridin-2-yl)-3-phenylpropan-1-ol (2j)

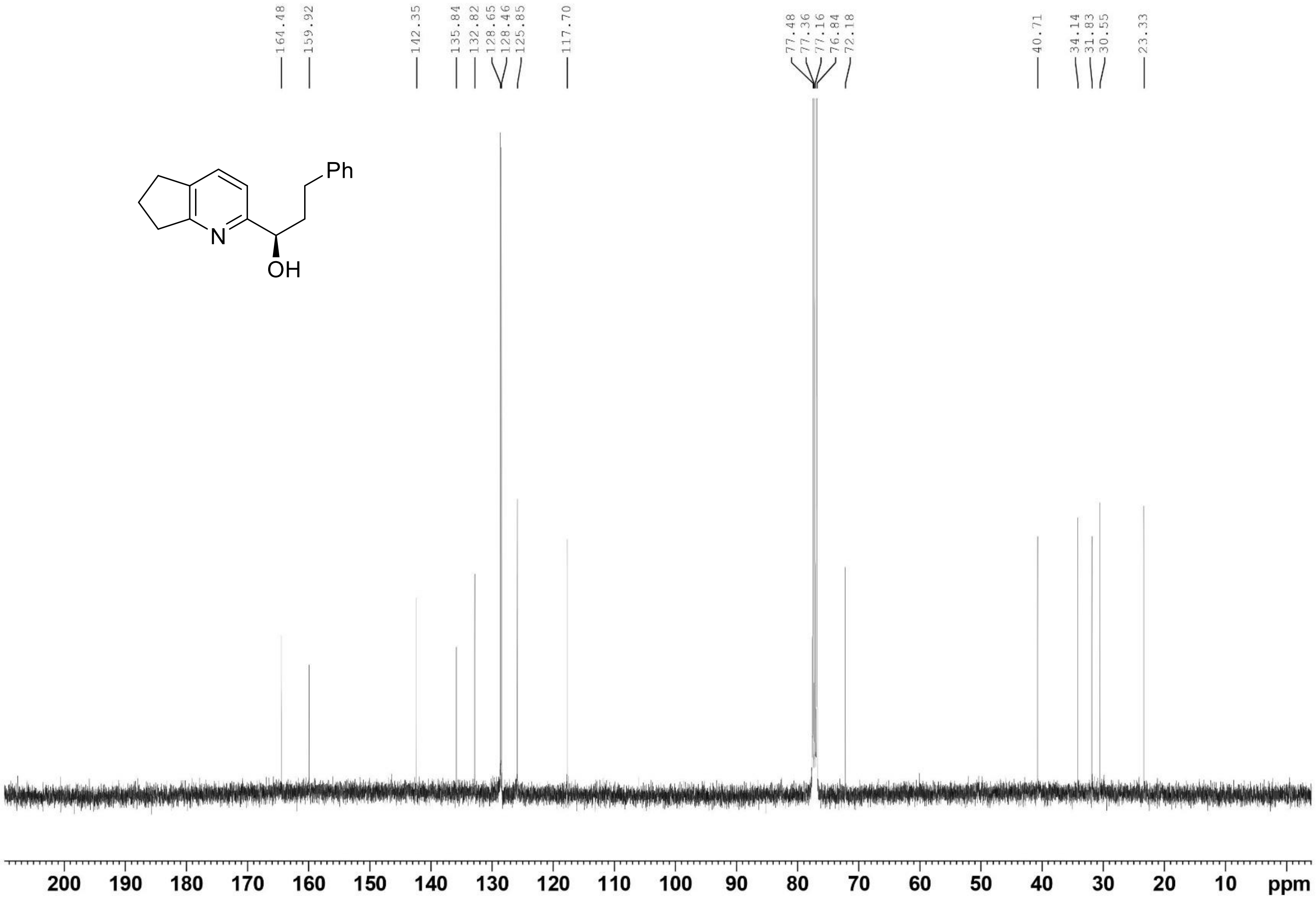

^{1}H NMR (400 MHz, $CDCl_3$) (*R*)-3-phenyl-1-(5,6,7,8-tetrahydroquinolin-2-yl)propan-1-ol (2k)

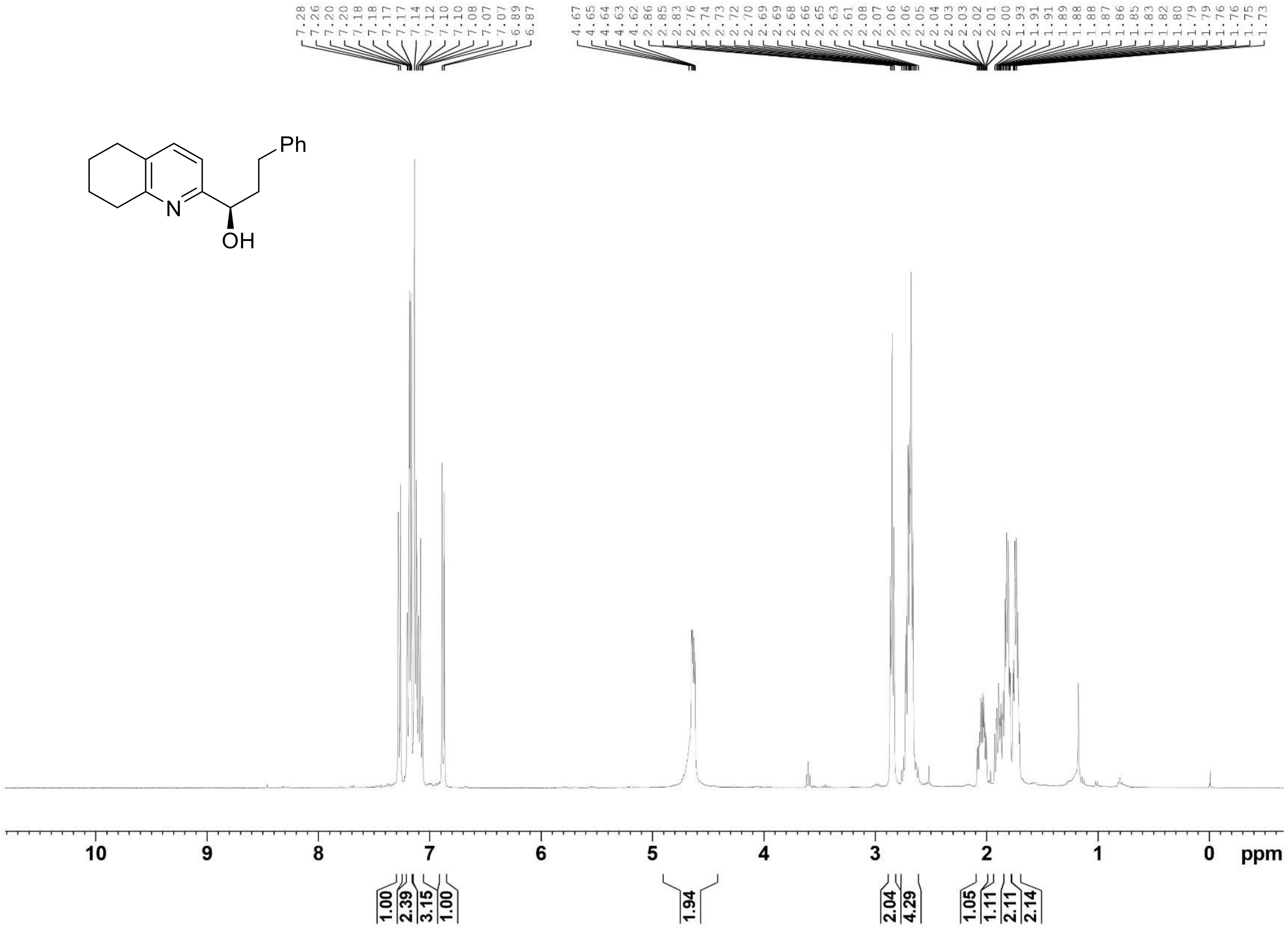

^{13}C NMR (101 MHz, $CDCl_3$) (*R*)-3-phenyl-1-(5,6,7,8-tetrahydroquinolin-2-yl)propan-1-ol (2k)

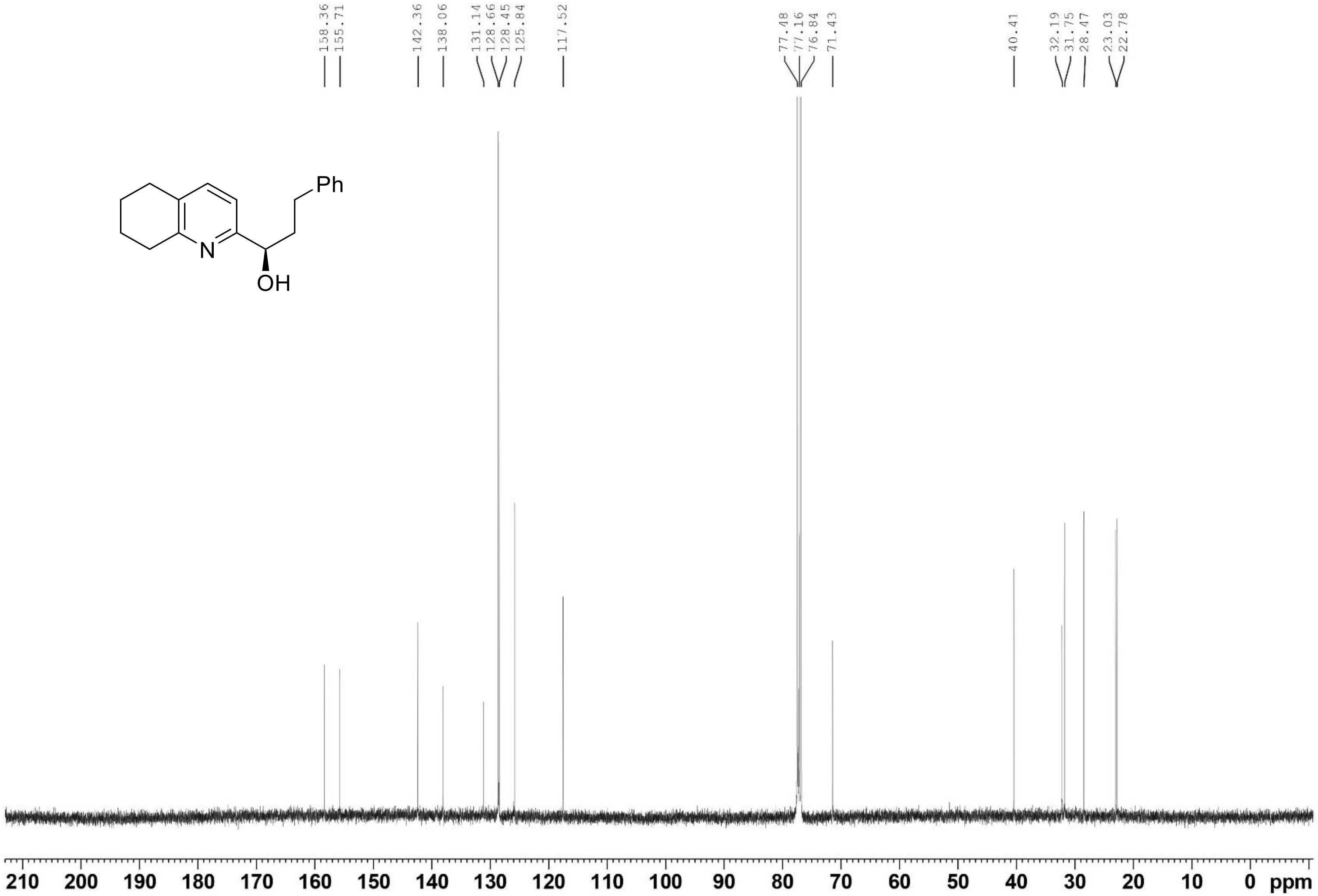

^{1}H NMR (700 MHz, $CDCl_3$) (*R*)-1-(5,6-dimethylpyridin-2-yl)-3-phenylpropan-1-ol (Xl)

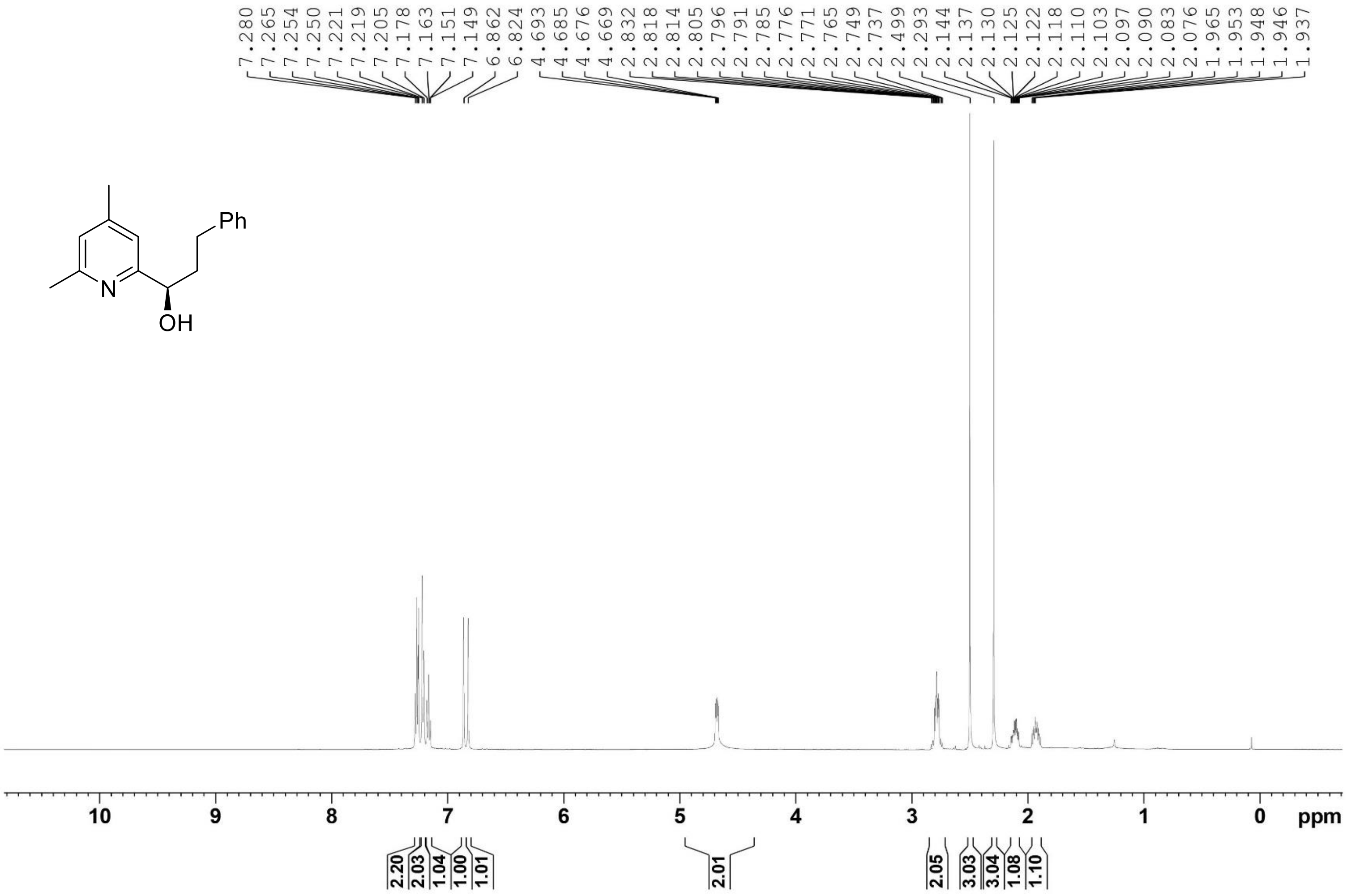

^{13}C NMR (126 MHz, $CDCl_3$) (*R*)-1-(5,6-dimethylpyridin-2-yl)-3-phenylpropan-1-ol (XI)

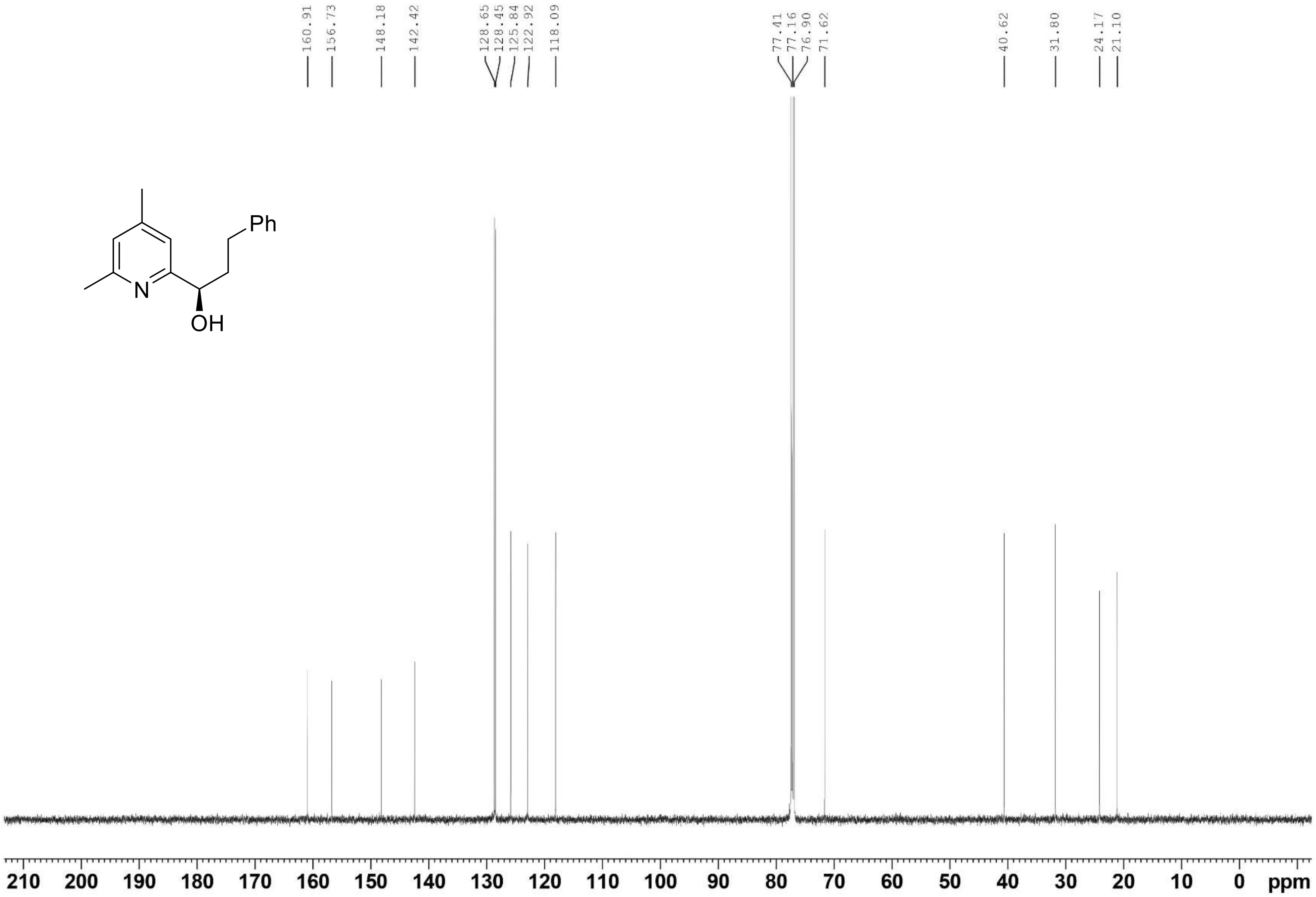

^{1}H NMR (500 MHz, $CDCl_3$) (*R*)-1-(3,5-dimethylpyridin-2-yl)-3-phenylpropan-1-ol (2m)

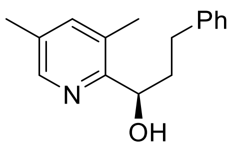

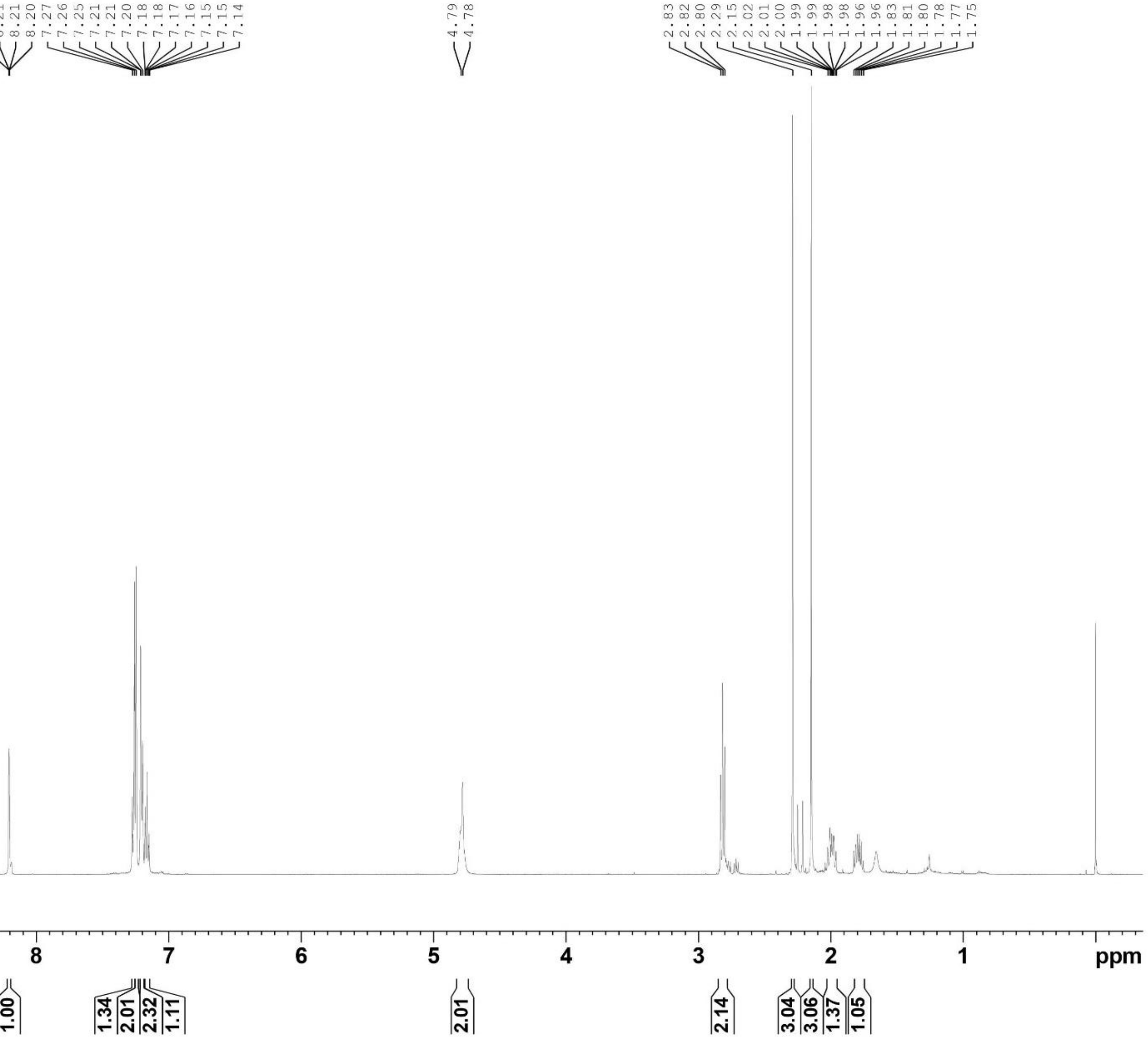

^{13}C NMR (126 MHz, $CDCl_3$) (*R*)-1-(3,5-dimethylpyridin-2-yl)-3-phenylpropan-1-ol (2m)

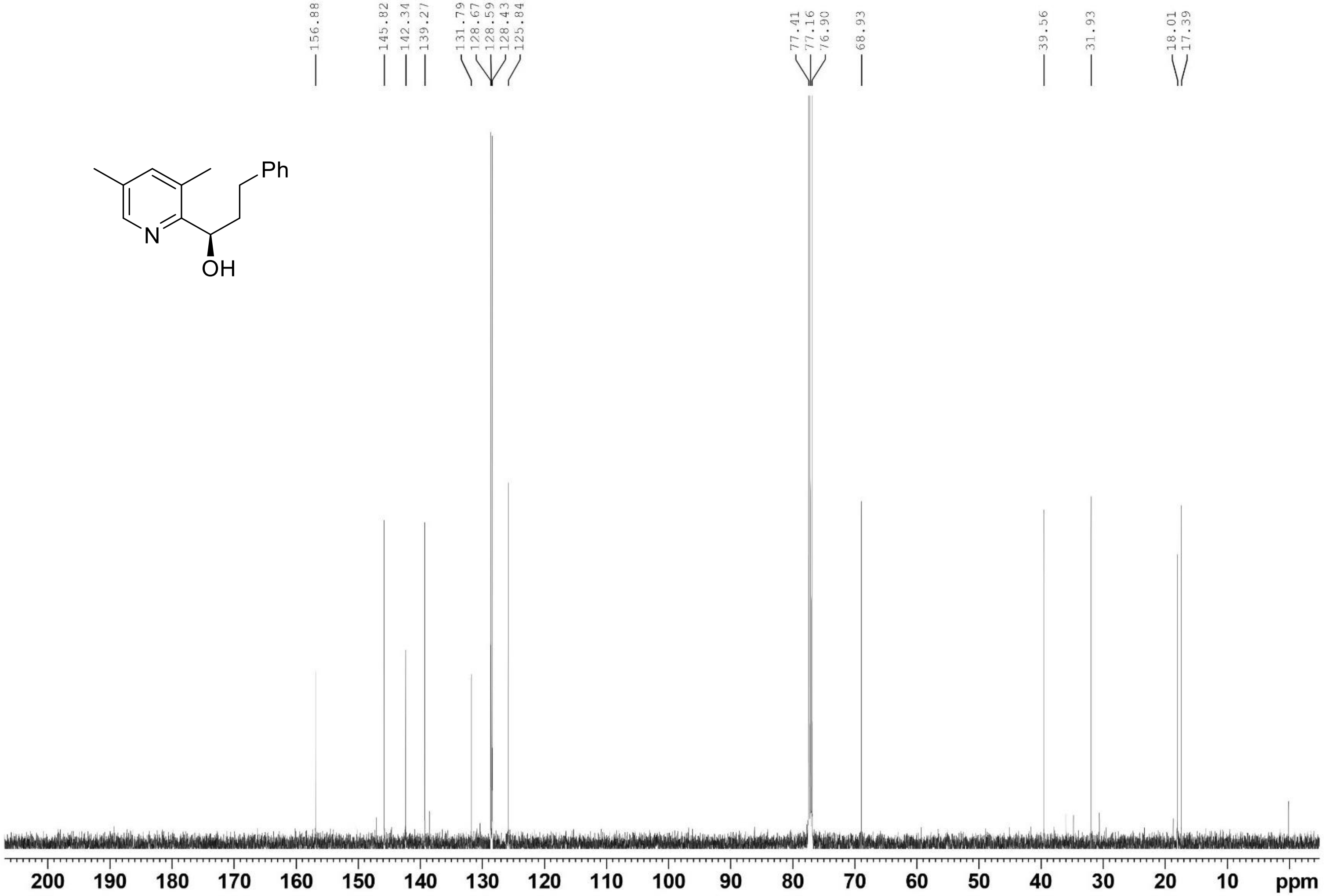

^{1}H NMR (500 MHz, CDCl$_3$) (*R*)-1-(3,6-dimethylpyridin-2-yl)-3-phenylpropan-1-ol (2n)

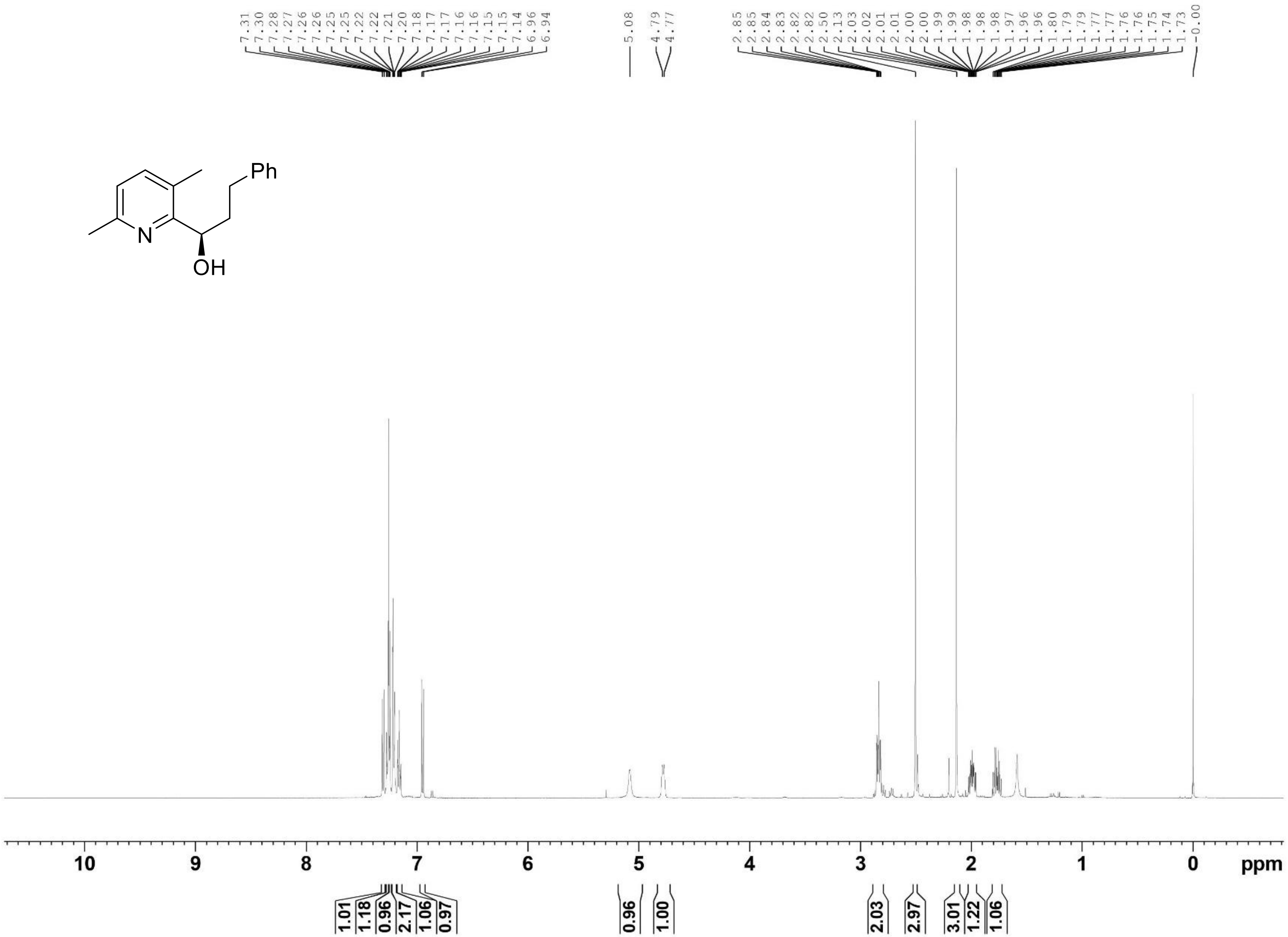

^{13}C NMR (126 MHz, $CDCl_3$) (*R*)-1-(3,6-dimethylpyridin-2-yl)-3-phenylpropan-1-ol (2n)

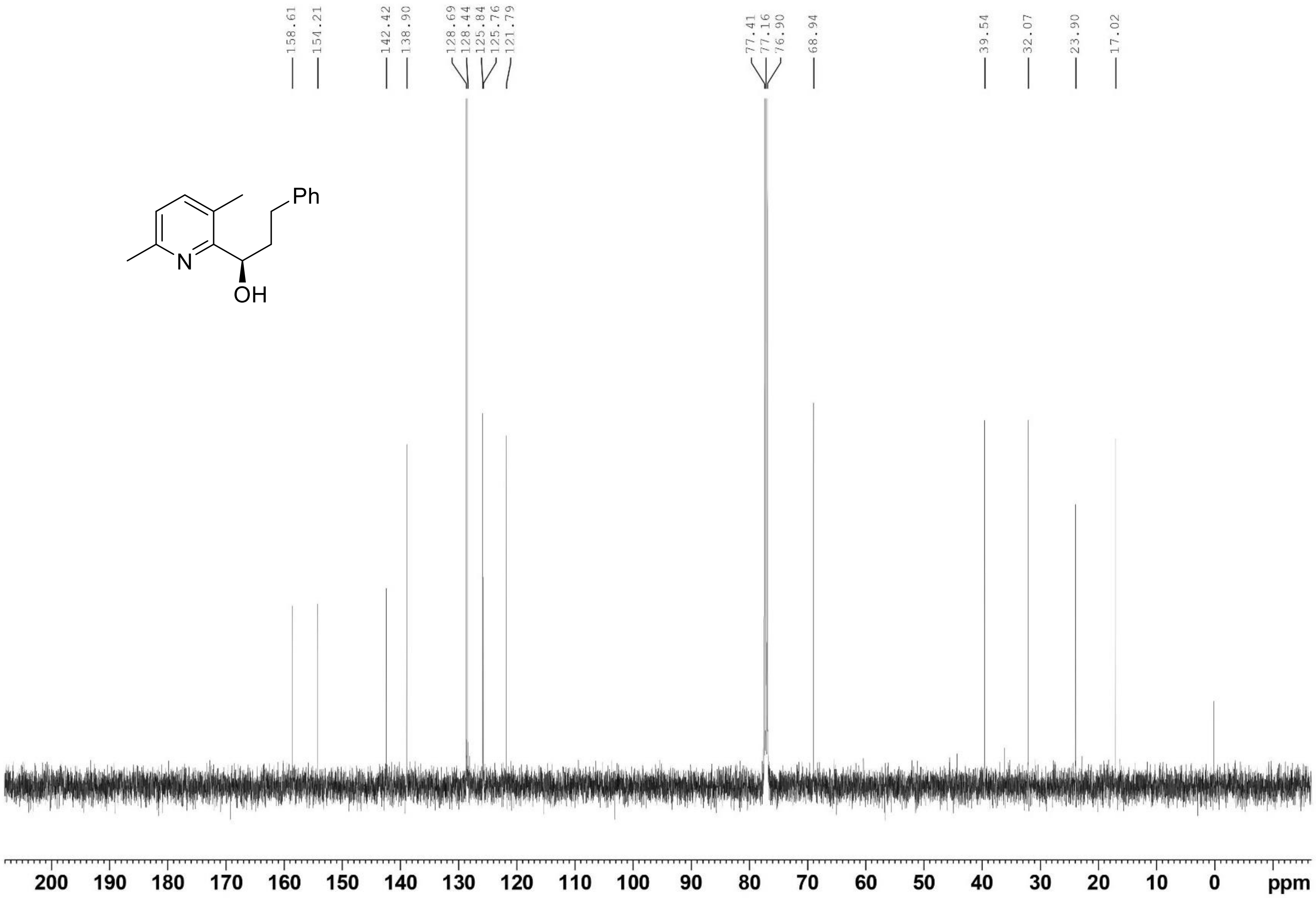

^{1}H NMR (500 MHz, $CDCl_3$) (*R*)-1-(3-methyl-5-phenylpyridin-2-yl)-3-phenylpropan-1-ol (2o)

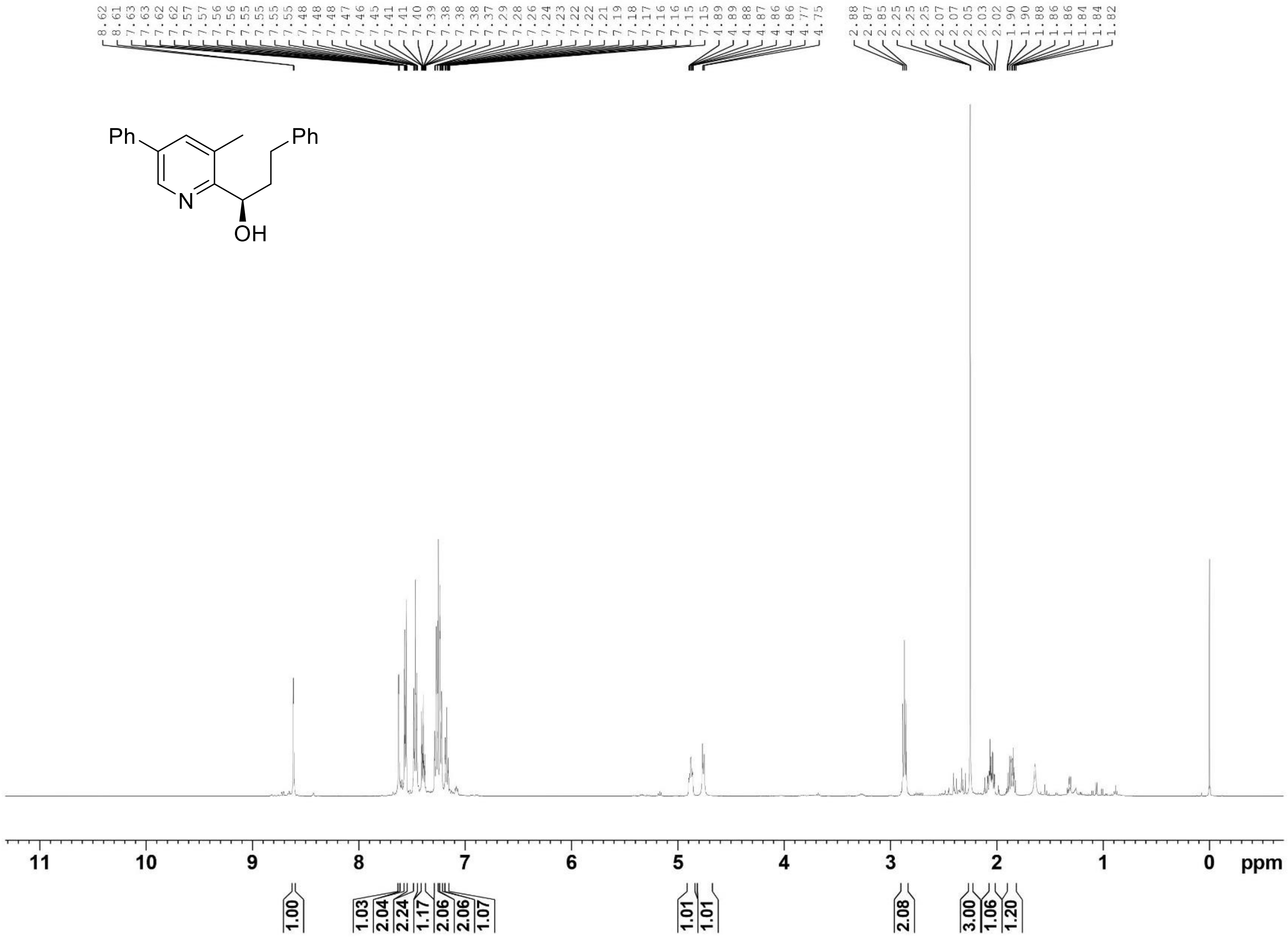

^{13}C NMR (126 MHz, $CDCl_3$) (*R*)-1-(3-methyl-5-phenylpyridin-2-yl)-3-phenylpropan-1-ol (2o)

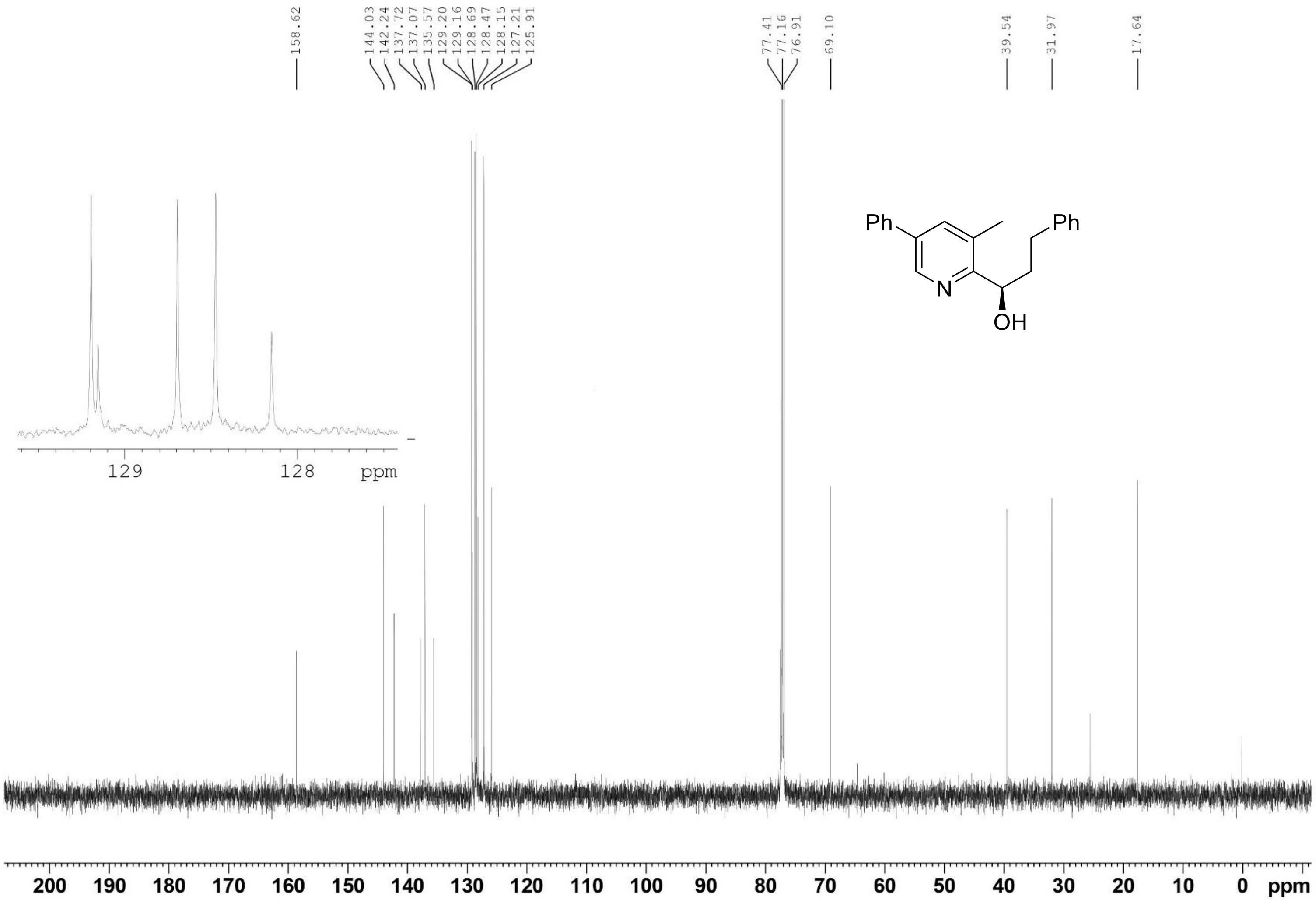

^{1}H NMR (500 MHz, $CDCl_3$) (*R*)-1-(3-methylpyridin-2-yl)-3-phenylpropan-1-ol (2pa)

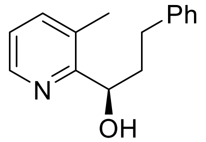

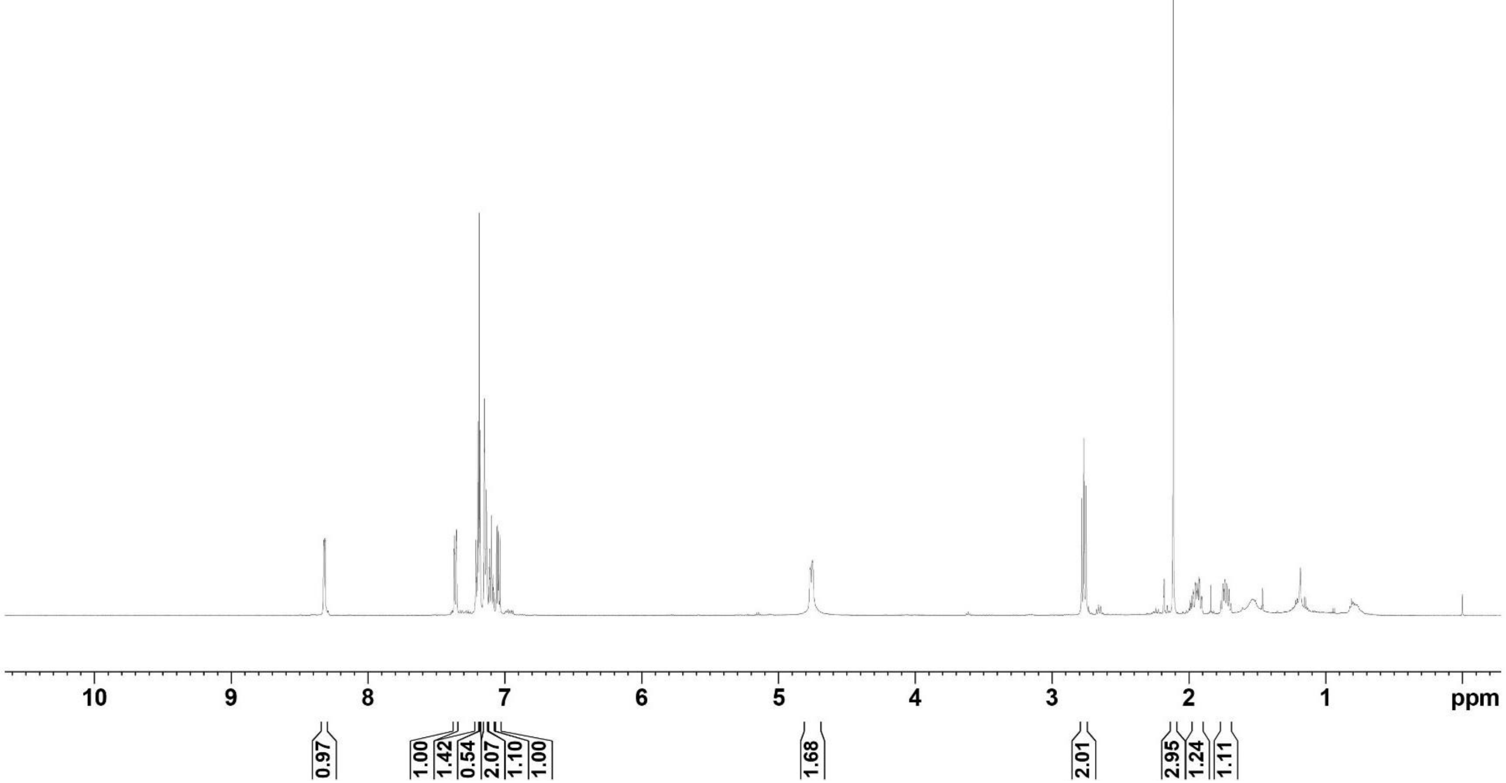

^{13}C NMR (126 MHz, $CDCl_3$) (*R*)-1-(3-methylpyridin-2-yl)-3-phenylpropan-1-ol (2pa)

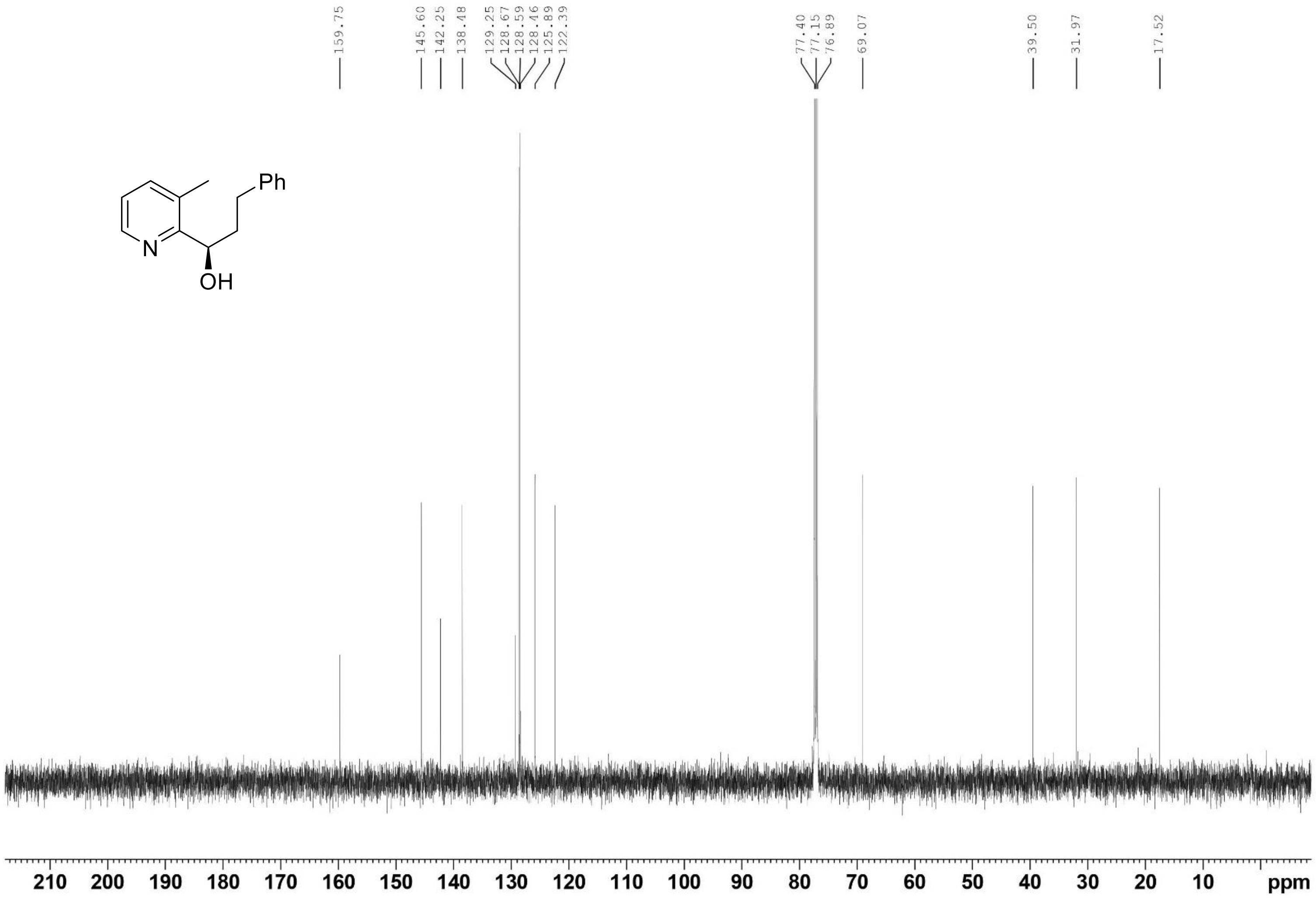

^{1}H NMR (500 MHz, $CDCl_3$) (*R*)-1-(5-methylpyridin-2-yl)-3-phenylpropan-1-ol (2pb)

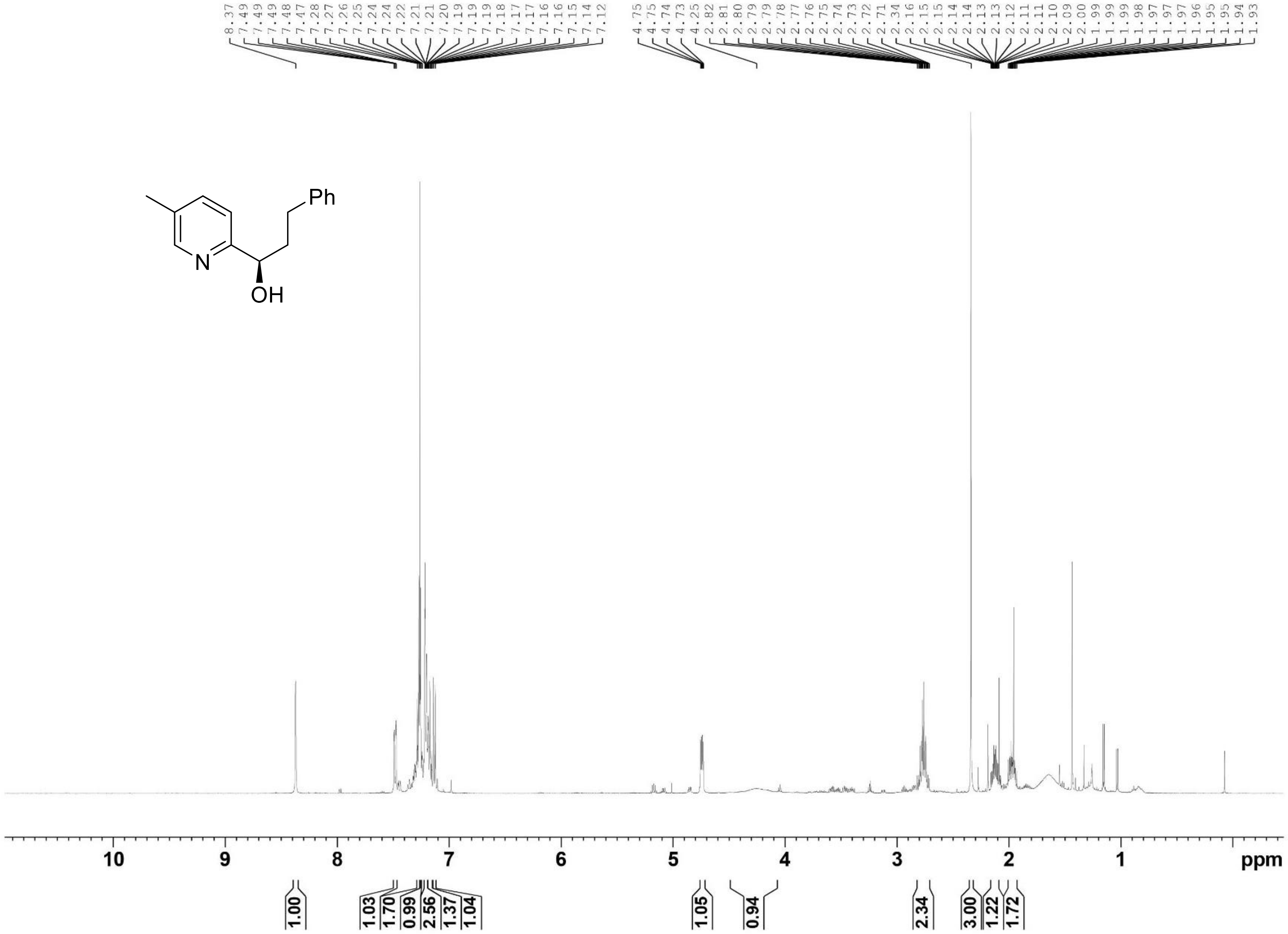

^{13}C NMR (126 MHz, $CDCl_3$) (*R*)-1-(5-methylpyridin-2-yl)-3-phenylpropan-1-ol (2pb)

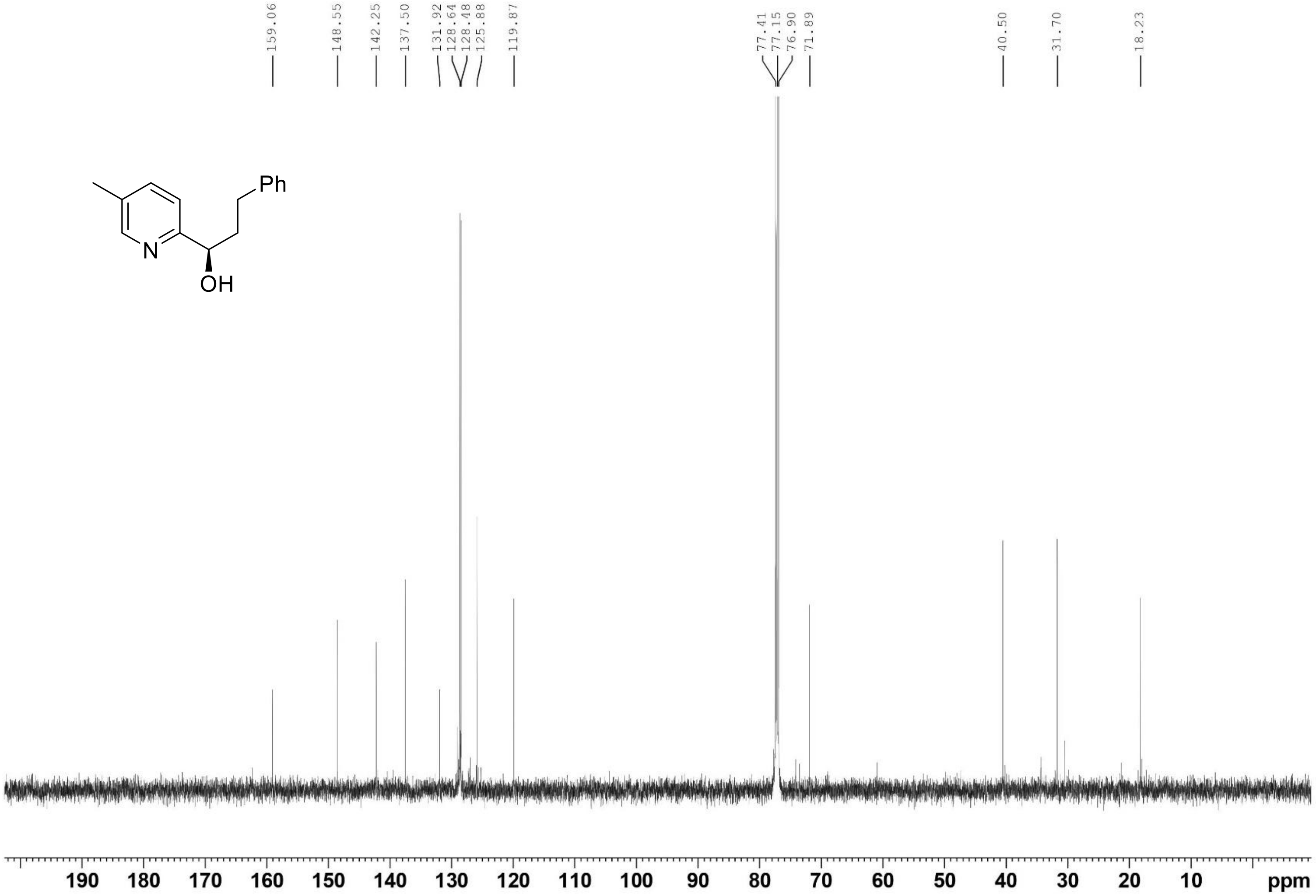

^{1}H NMR (500 MHz, $CDCl_3$) *tert*-butyl (*R*)-((6-(1-hydroxy-3-phenylpropyl)-5-methylpyridin-2-yl)methyl)carbamate (2q)

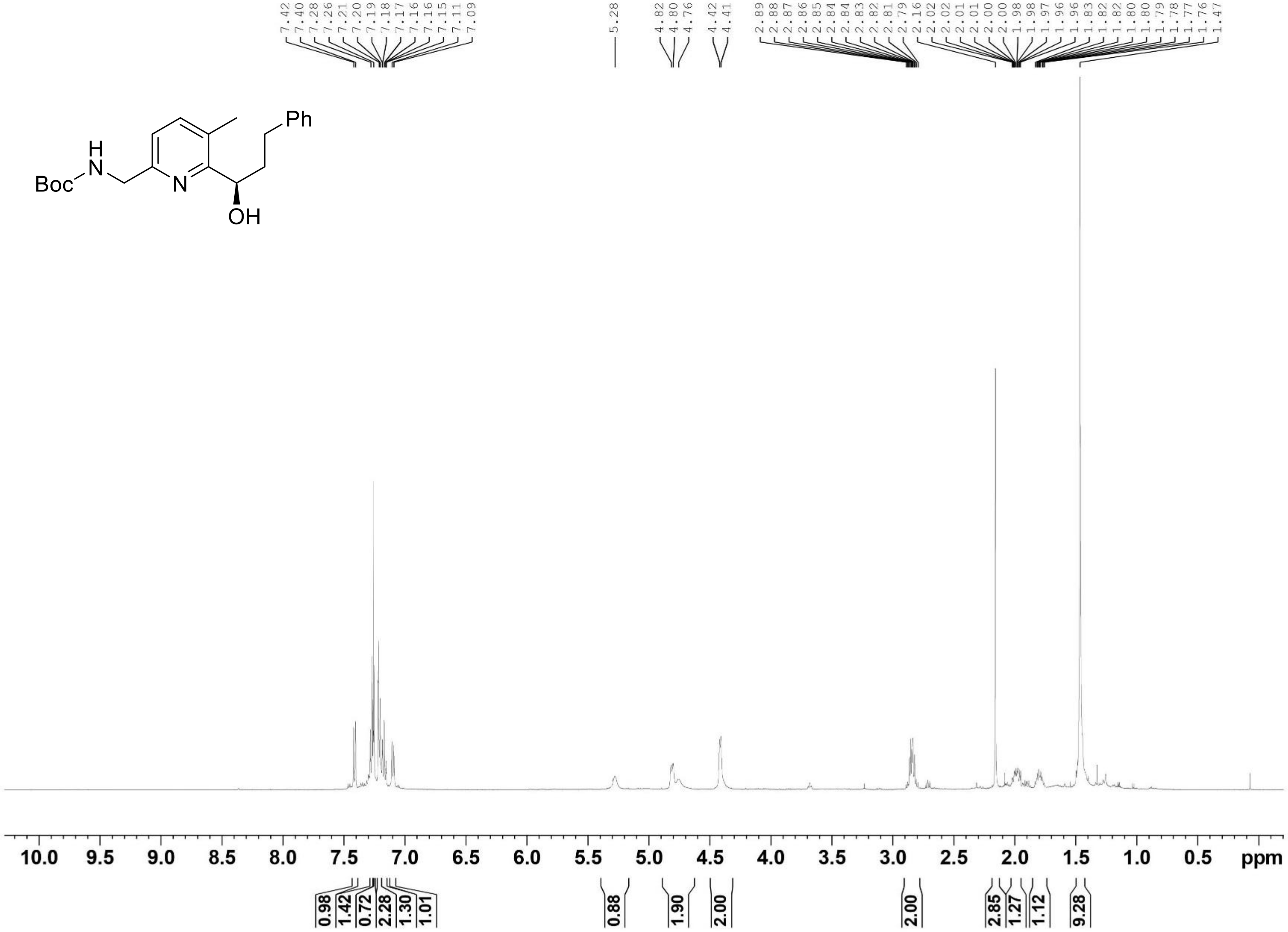

^{13}C NMR (126 MHz, $CDCl_3$) *tert*-butyl (*R*)-((6-(1-hydroxy-3-phenylpropyl)-5-methylpyridin-2-yl)methyl)carbamate (2q)

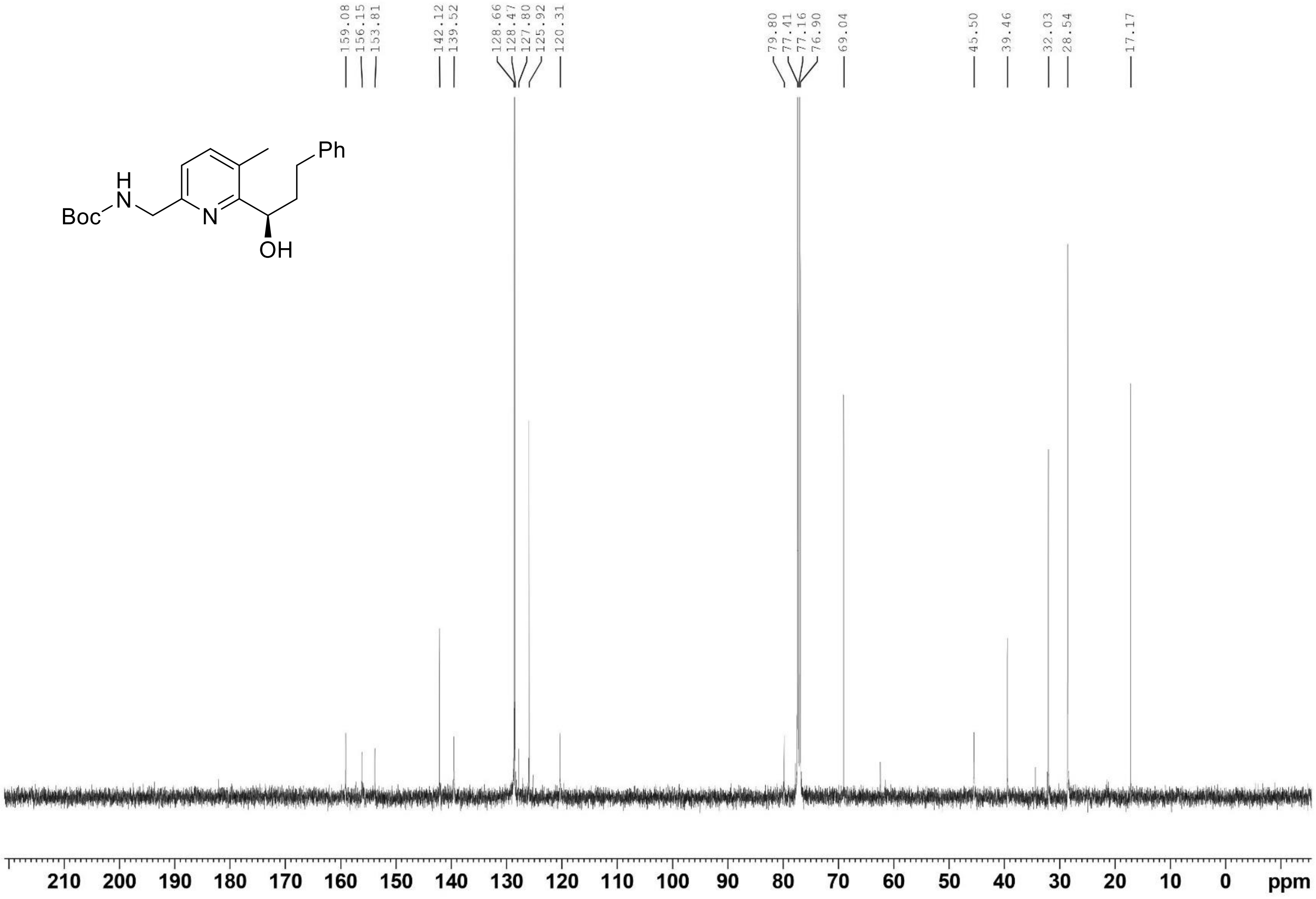

^{1}H NMR (500 MHz, $CDCl_3$) (*R*)-1-(3-ethyl-6-methylpyridin-2-yl)-3-phenylpropyl acetate (2ra)

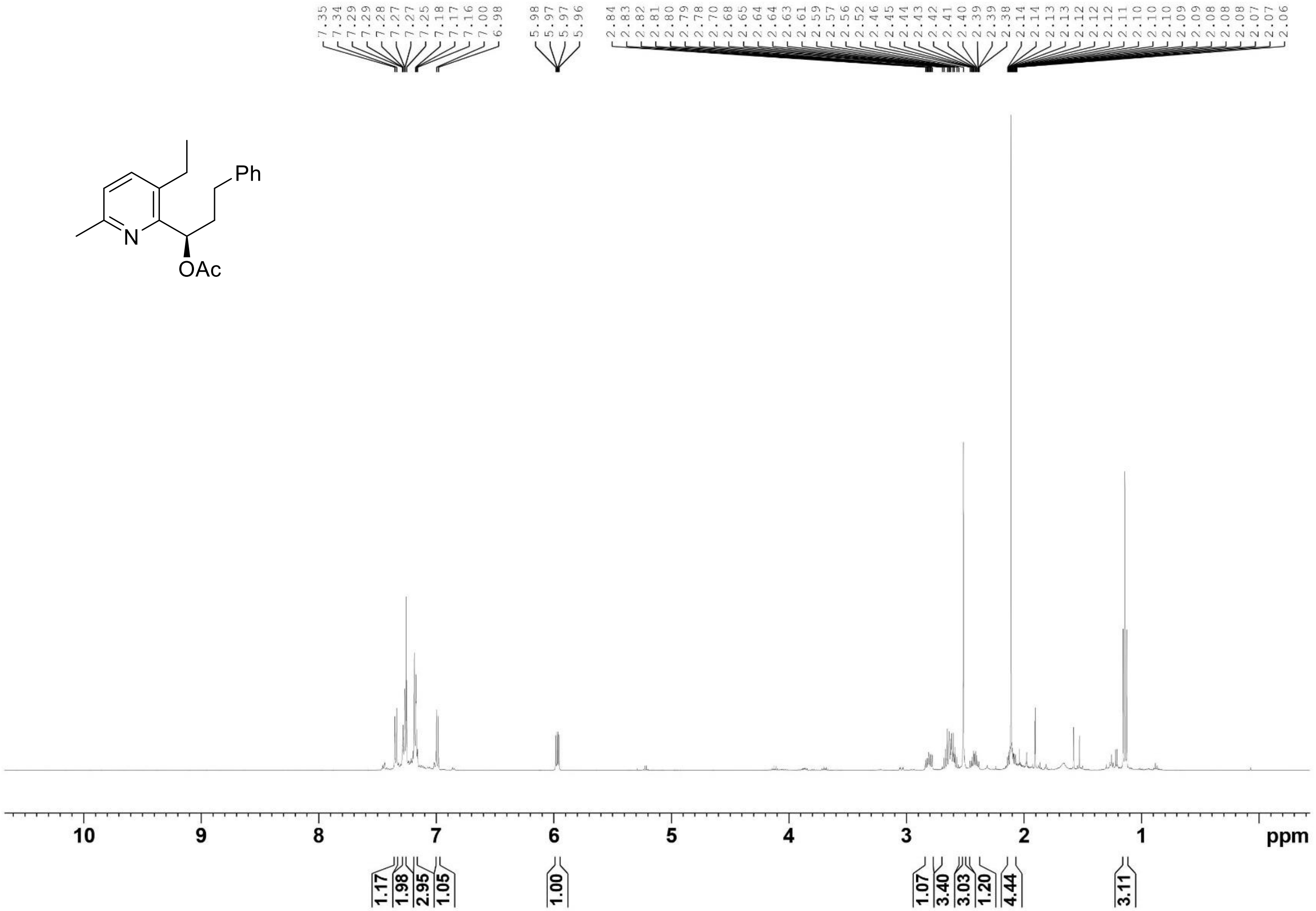

^{13}C NMR (126 MHz, $CDCl_3$) (*R*)-1-(3-ethyl-6-methylpyridin-2-yl)-3-phenylpropyl acetate (2ra)

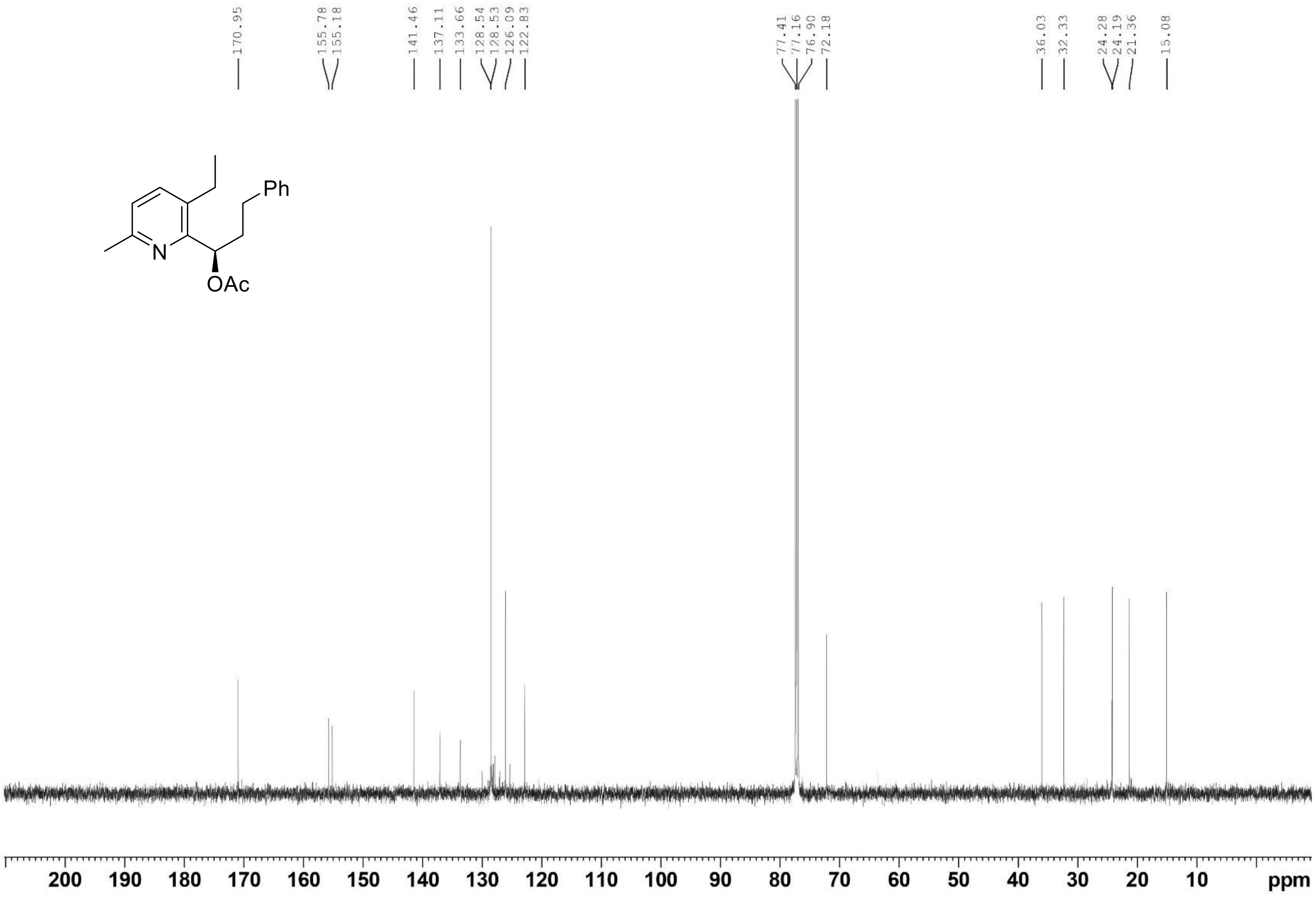

^{1}H NMR (500 MHz, $CDCl_3$) (*R*)-3-phenyl-1-(pyridin-2-yl)propyl acetate (2sa)

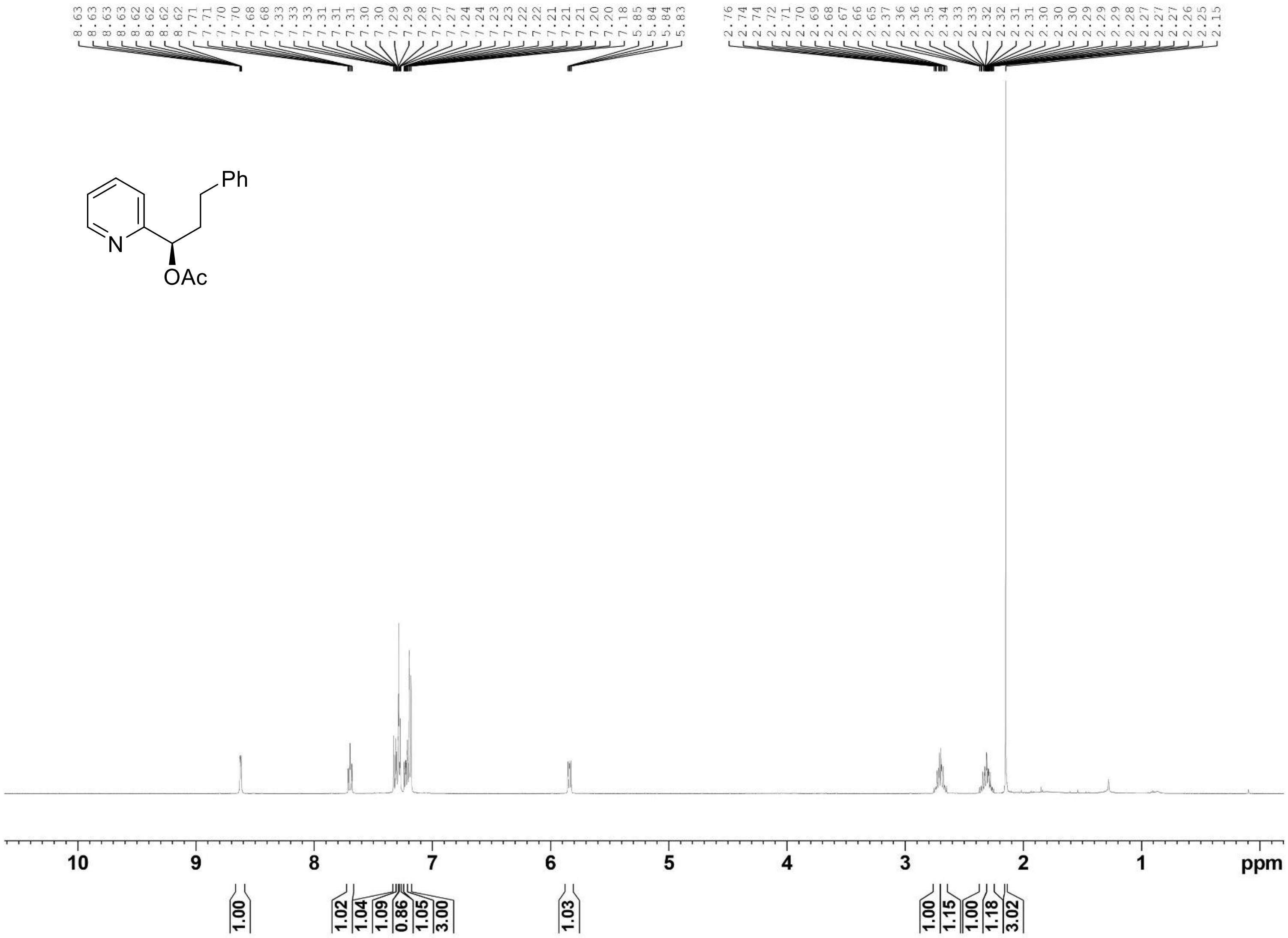

^{13}C NMR (126 MHz, $CDCl_3$) (*R*)-3-phenyl-1-(pyridin-2-yl)propyl acetate (2sa)

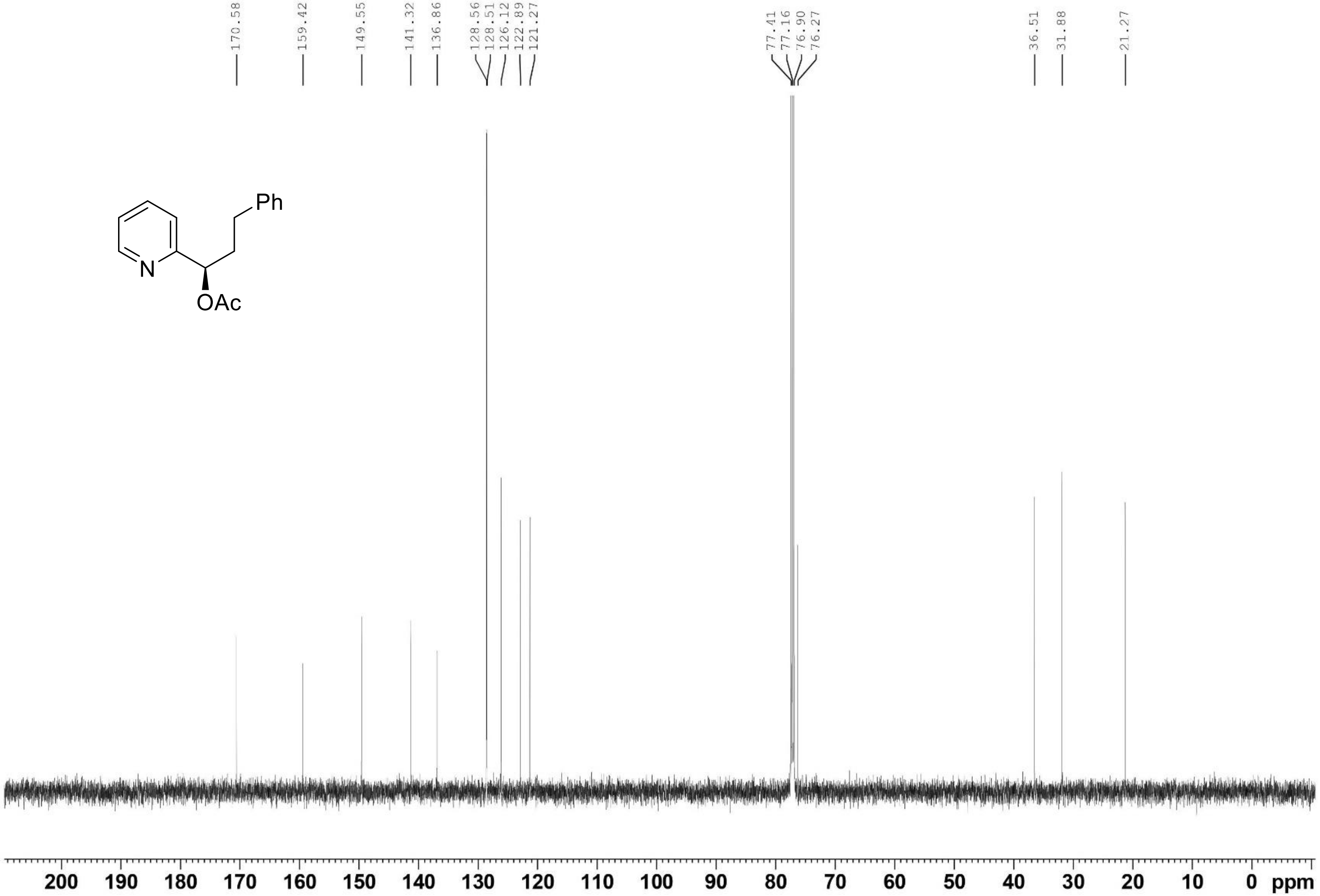

^{1}H NMR (500 MHz, CDCl$_3$) (1*R*,1'*R*)-pyridine-2,6-diylbis(3-phenylpropane-1,1-diyl) diacetate (2ua)

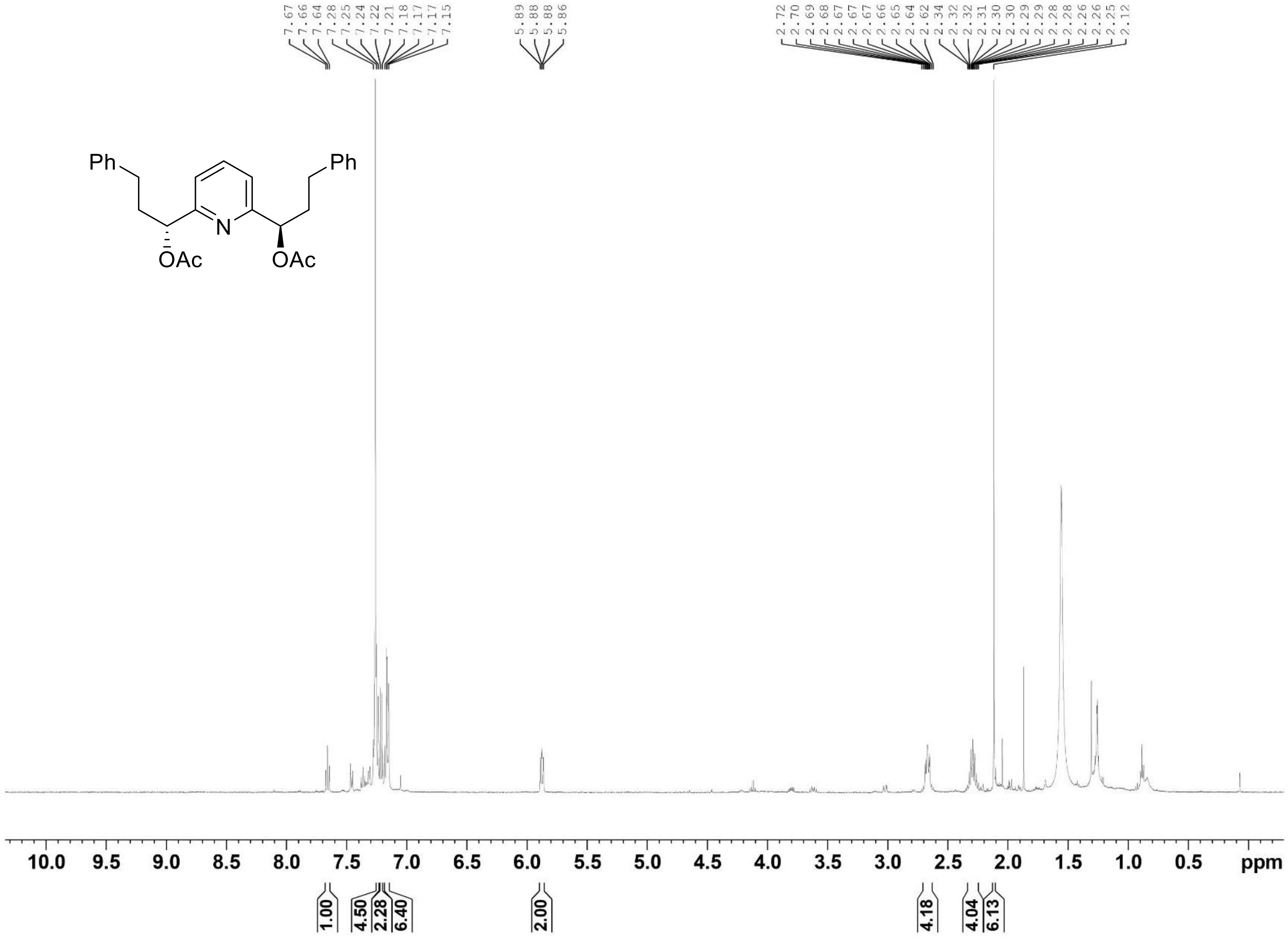

^{13}C NMR (126 MHz, CDCl$_3$) (1*R*,1'*R*)-pyridine-2,6-diylbis(3-phenylpropane-1,1-diyl) diacetate (2ua)

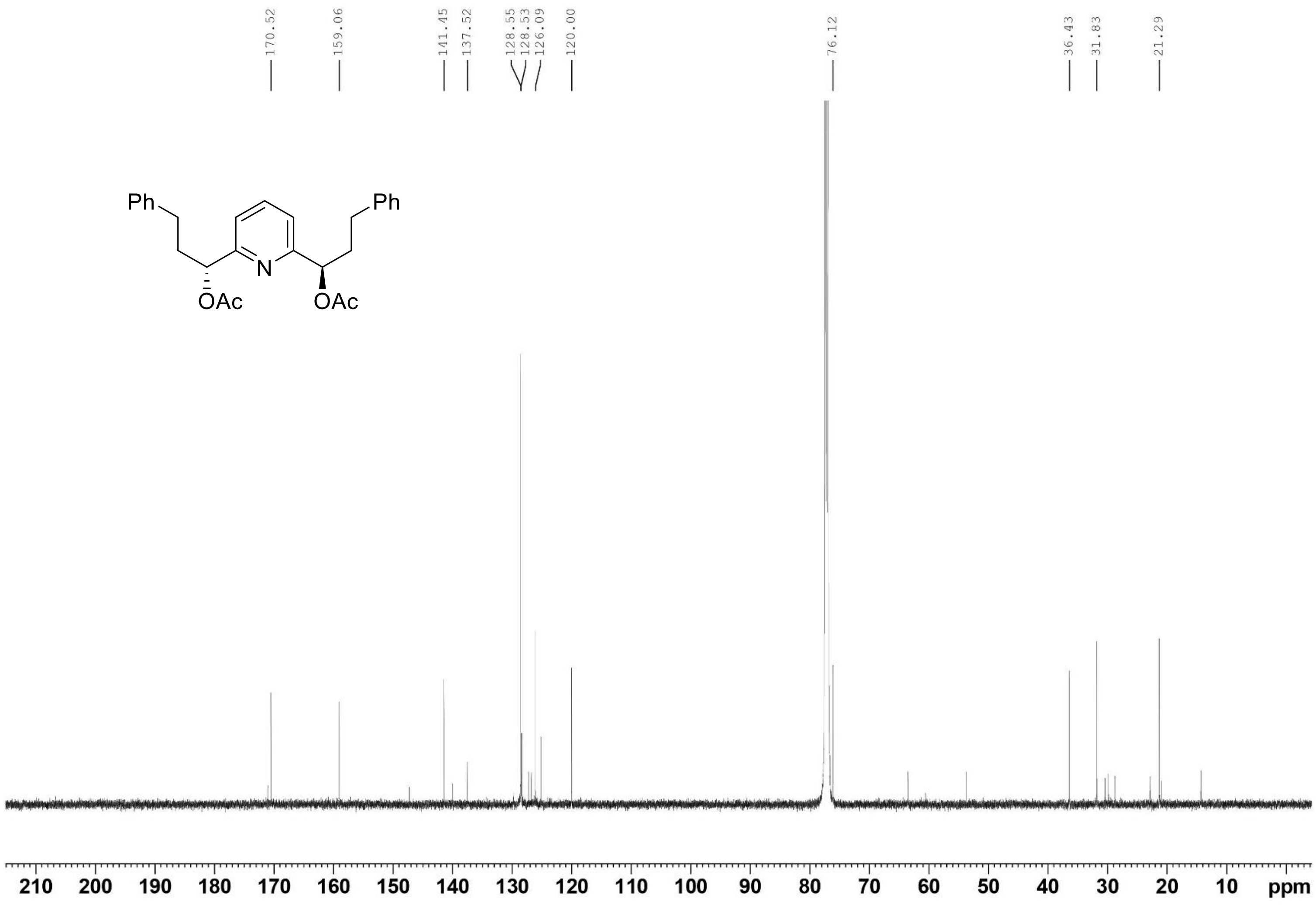

^{1}H NMR (500 MHz, $CDCl_3$) (*R*)-1-(4-(*tert*-butyl)pyridin-2-yl)-3-phenylpropan-1-ol (2t)

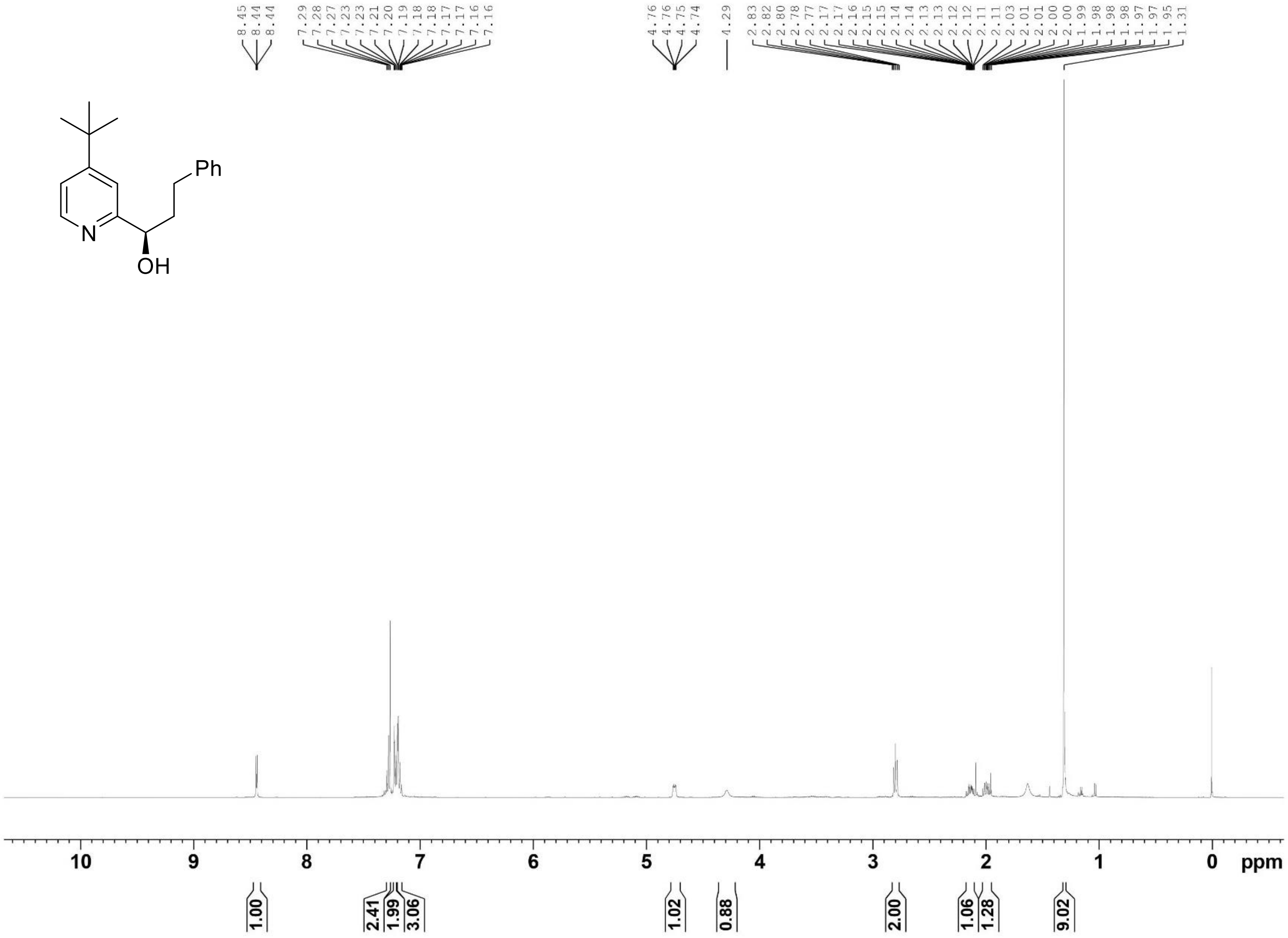

^{13}C NMR (126 MHz, CDCl$_3$) (*R*)-1-(4-(*tert*-butyl)pyridin-2-yl)-3-phenylpropan-1-ol (2t)

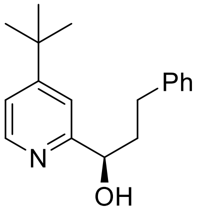

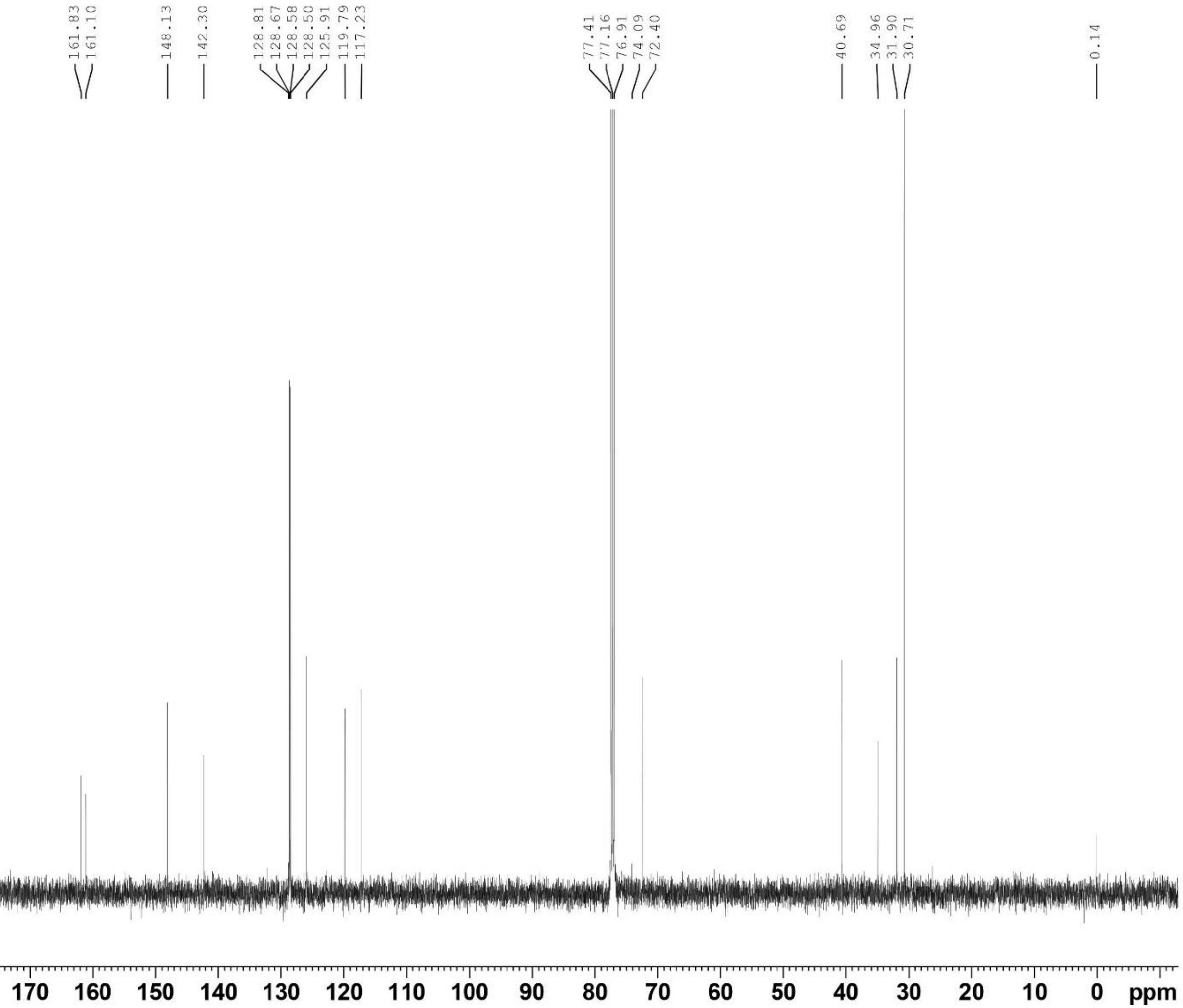

^{1}H NMR (400 MHz, $CDCl_3$) (*R*)-1-(4-(tert-butyl)pyridin-2-yl)-3-phenylpropyl acetate (2ta)

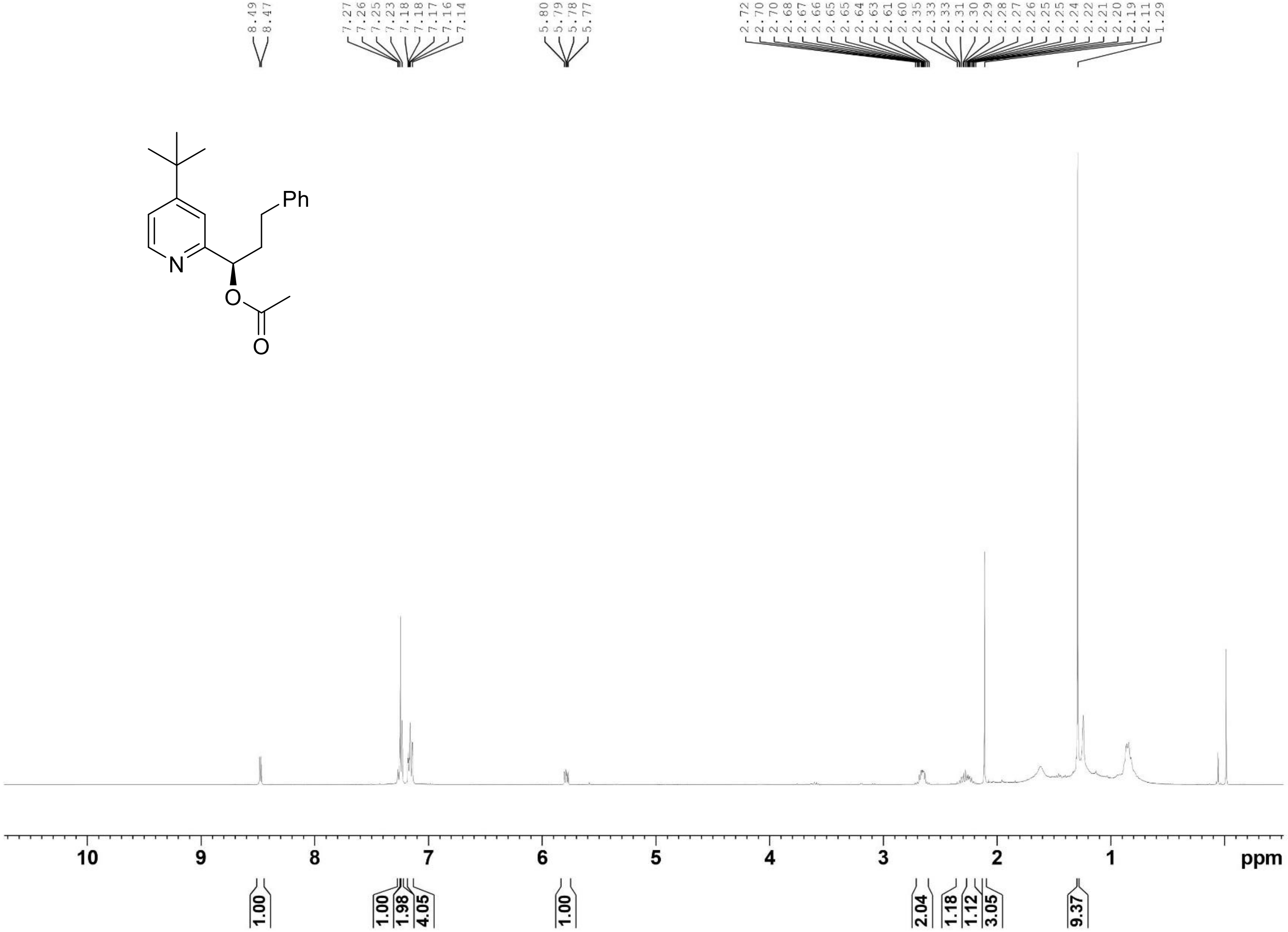

^{13}C NMR (101 MHz, $CDCl_3$) (*R*)-1-(4-(tert-butyl)pyridin-2-yl)-3-phenylpropyl acetate (2ta)

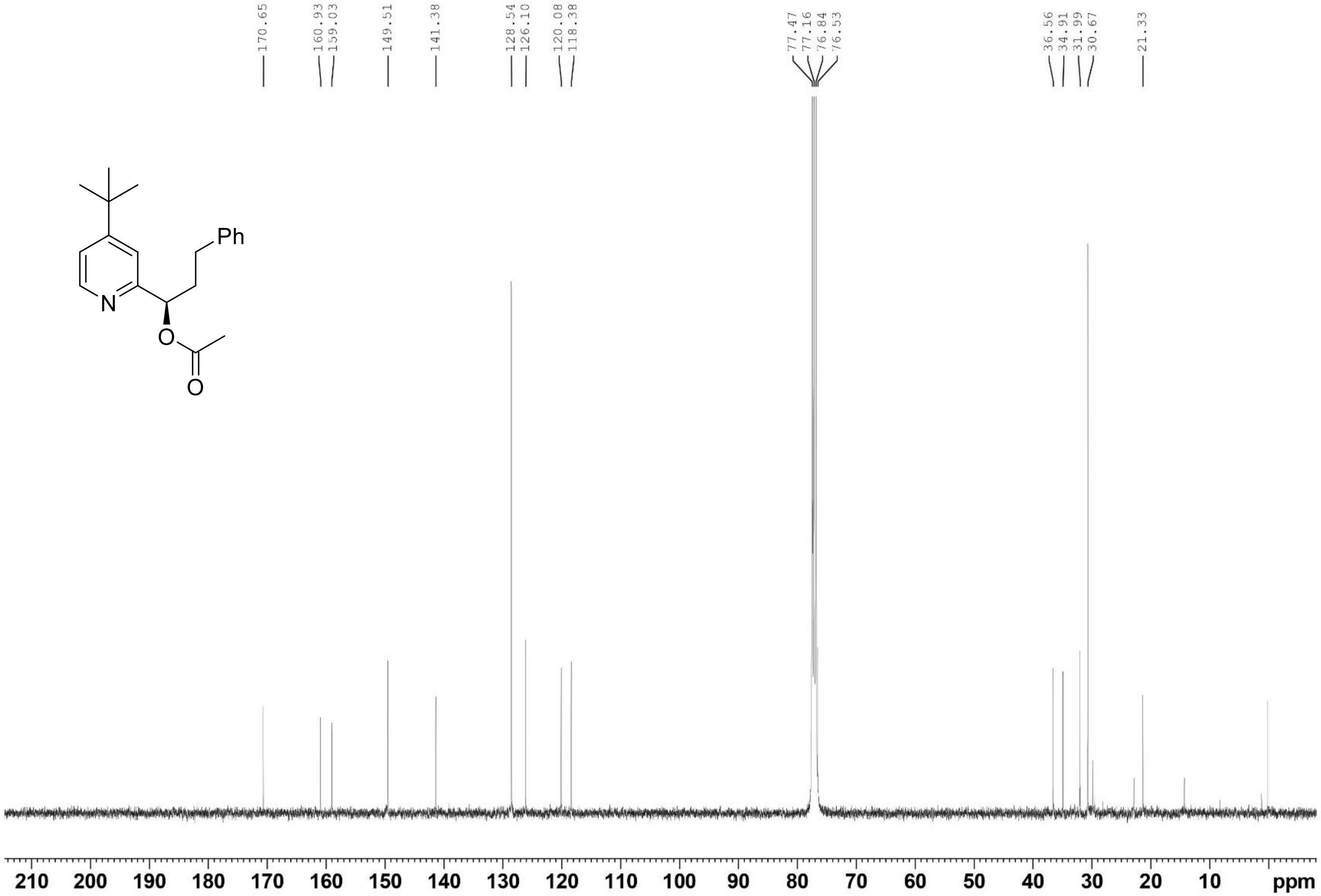

^{1}H NMR (500 MHz, CDCl$_3$) *tert*-butyl (2-(1-hydroxy-3-phenylpropyl)pyridin-3-yl)carbamate 2xa

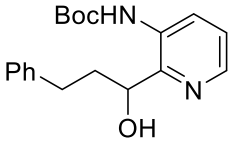

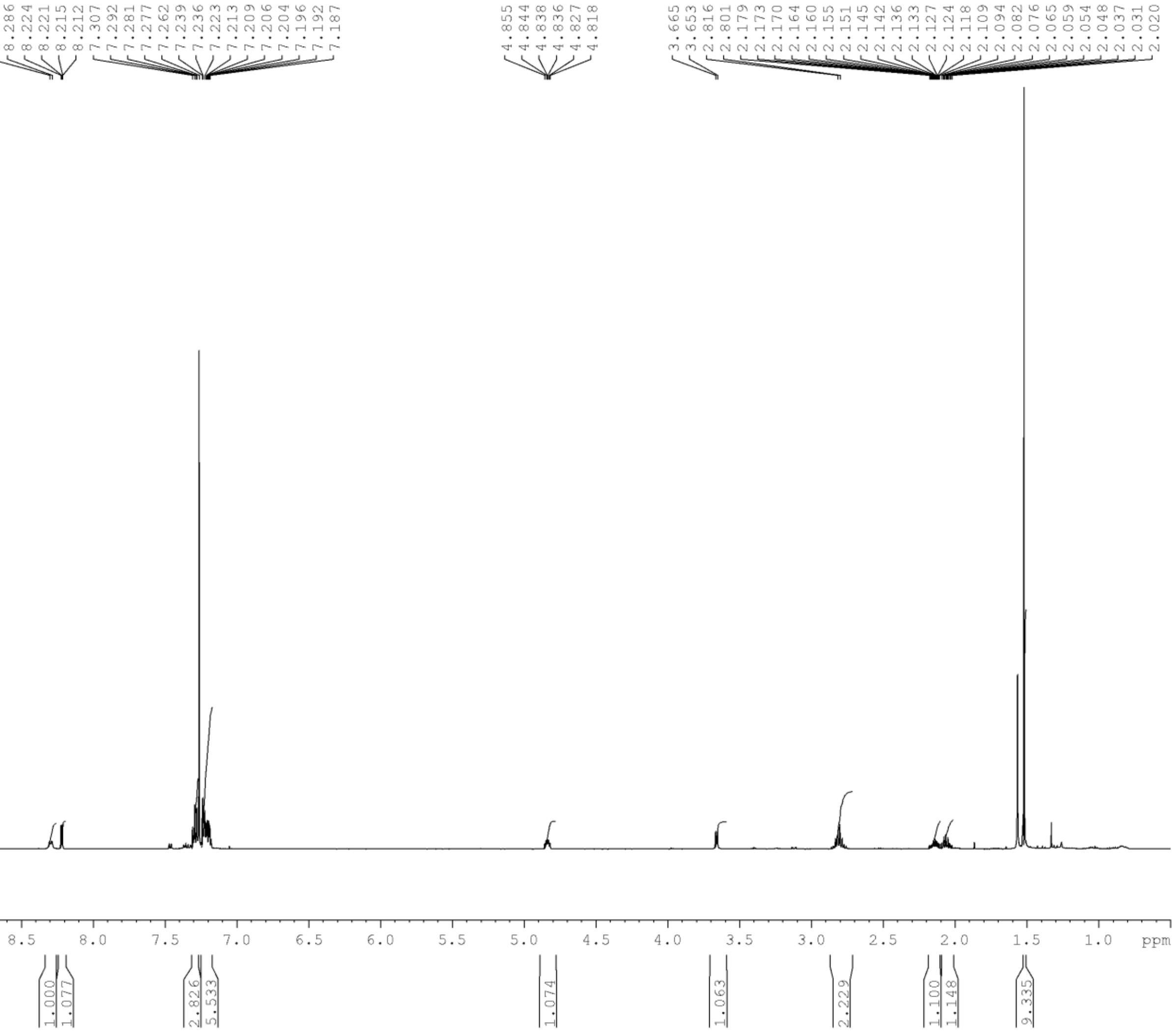

^{13}C NMR (126 MHz, CDCl$_3$) *tert*-butyl (2-(1-hydroxy-3-phenylpropyl)pyridin-3-yl)carbamate 2xa

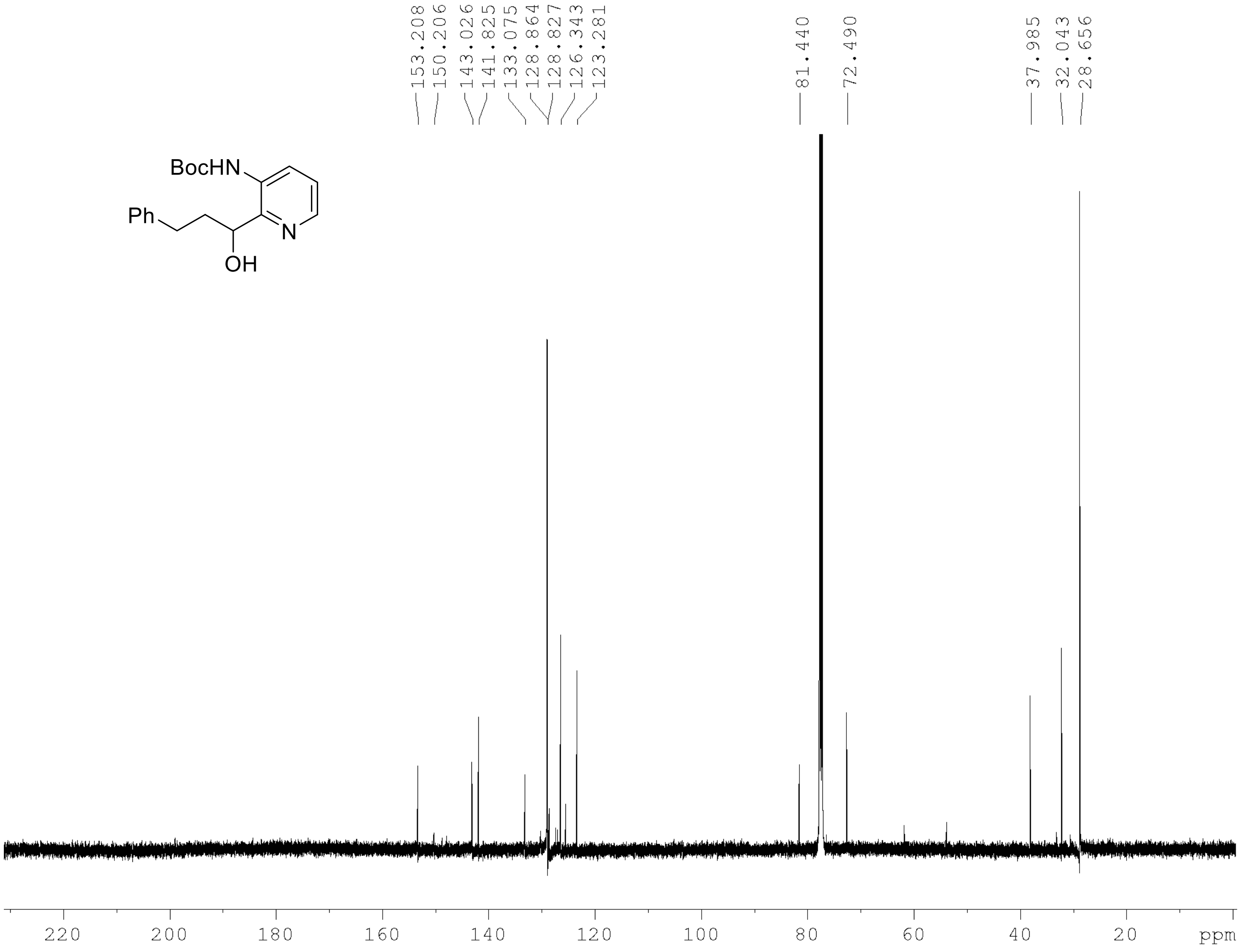

^{1}H NMR (500 MHz, $CDCl_3$) *tert*-Butyl (*R*)-(6-(1-hydroxy-3-phenylpropyl)pyridin-3-yl)carbamate 2x

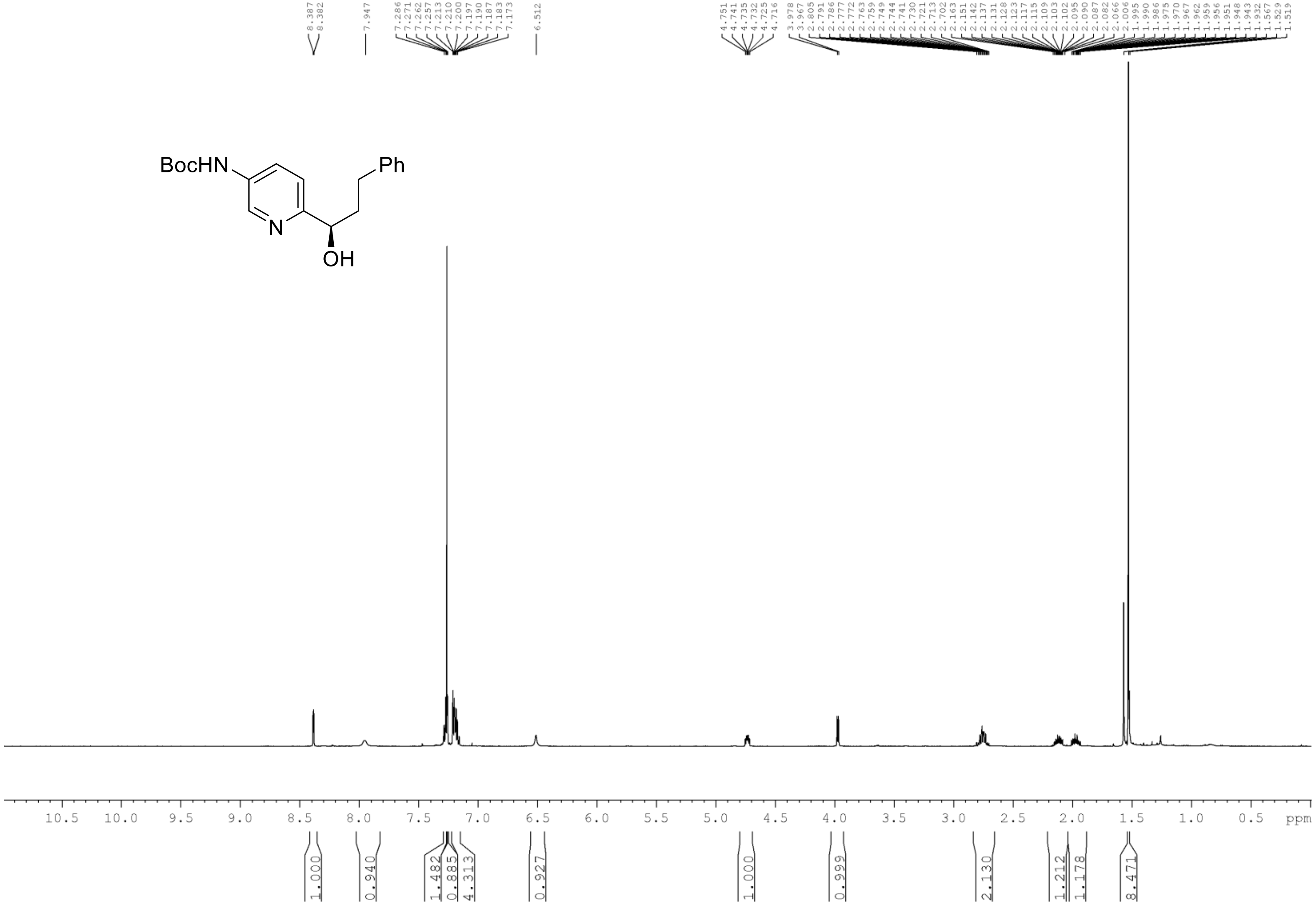

^{13}C NMR (126 MHz, $CDCl_3$)*tert*-Butyl (*R*)-(6-(1-hydroxy-3-phenylpropyl)pyridin-3-yl)carbamate 2x

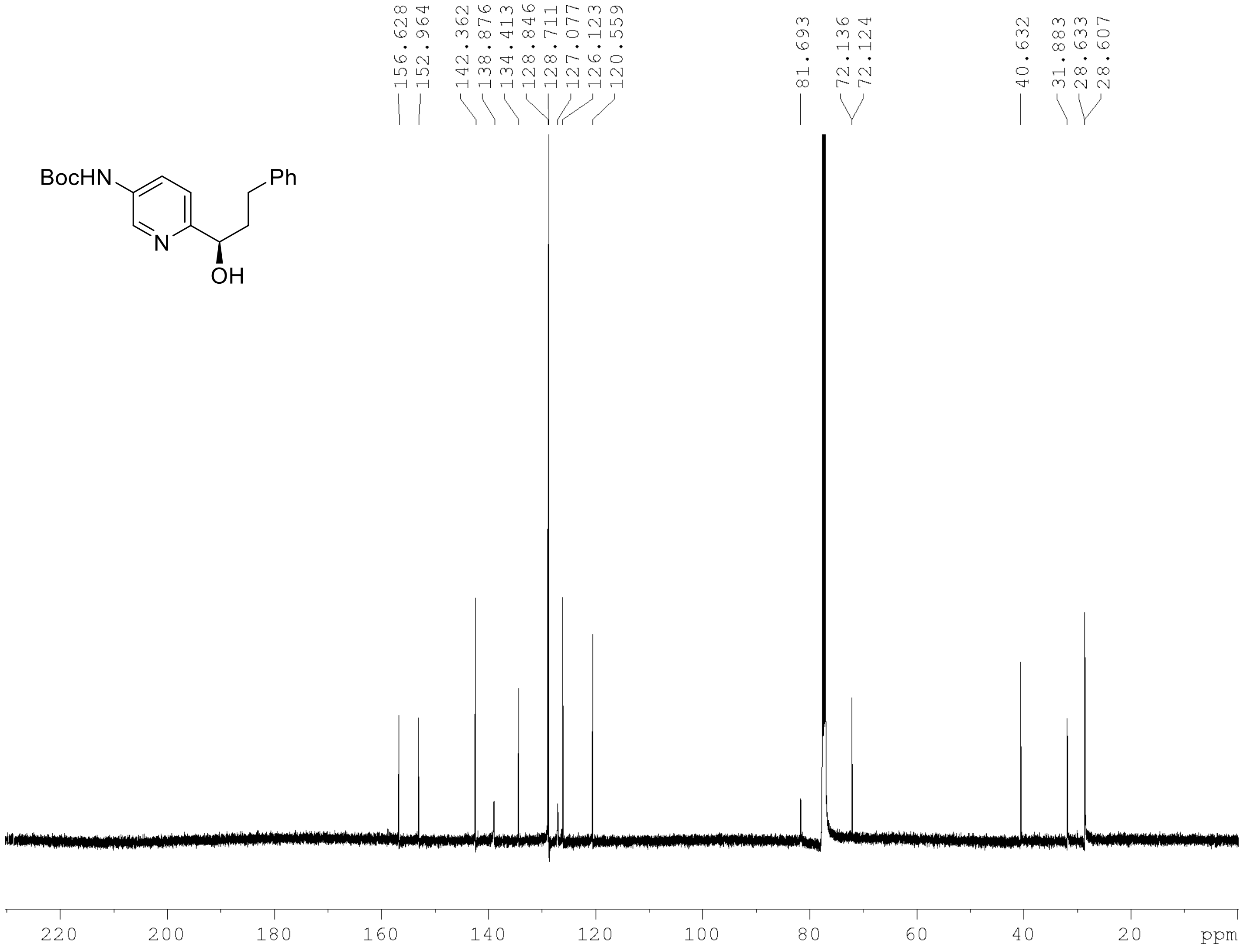

^{1}H NMR (400 MHz, $CDCl_3$) (*R*)-3-(2-Bromophenyl)-1-(6-pentylpyridin-2-yl)propan-1-ol (6a)

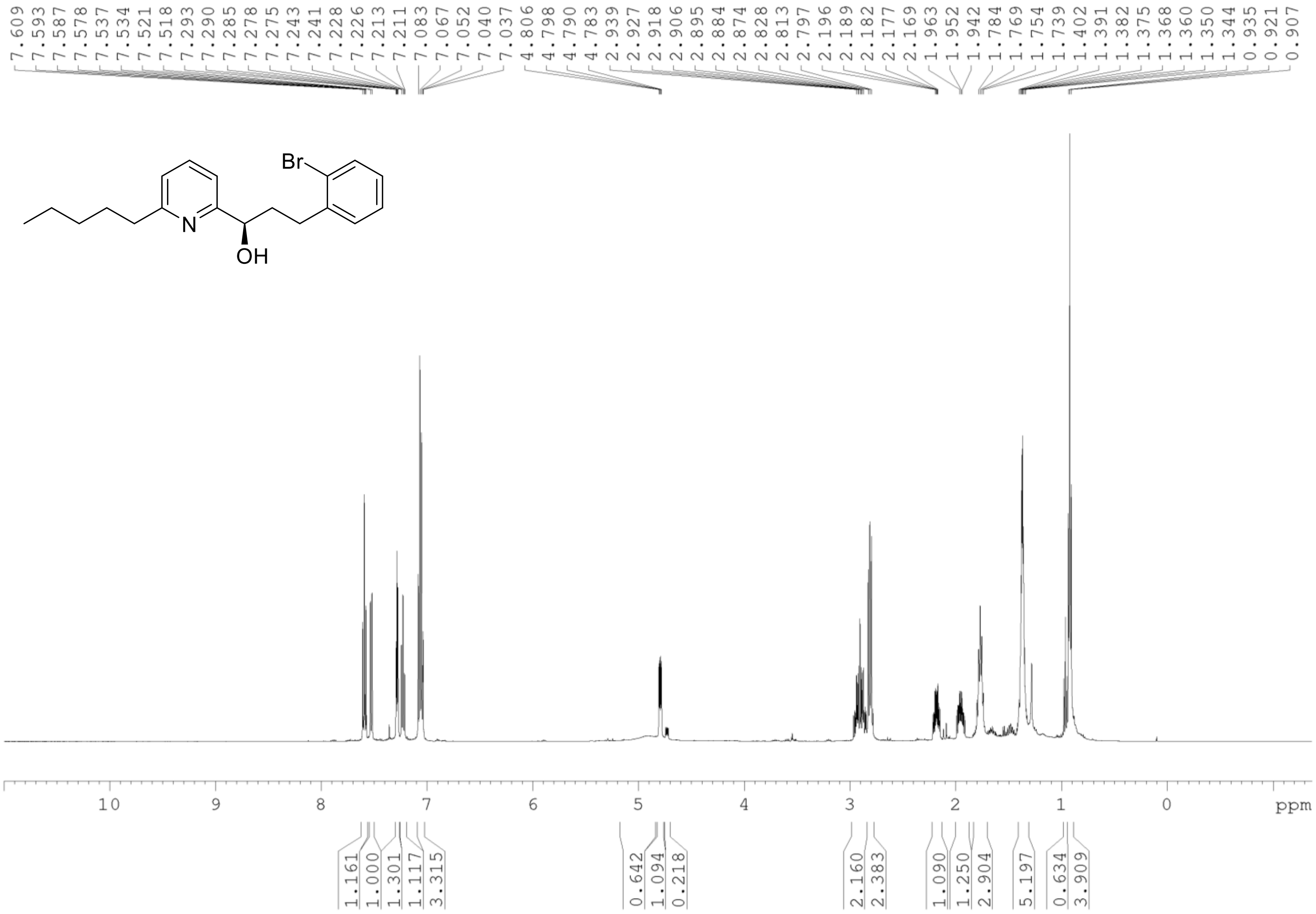

^{13}C NMR (101 MHz, $CDCl_3$) (*R*)-3-(2-Bromophenyl)-1-(6-pentylpyridin-2-yl)propan-1-ol (6a)

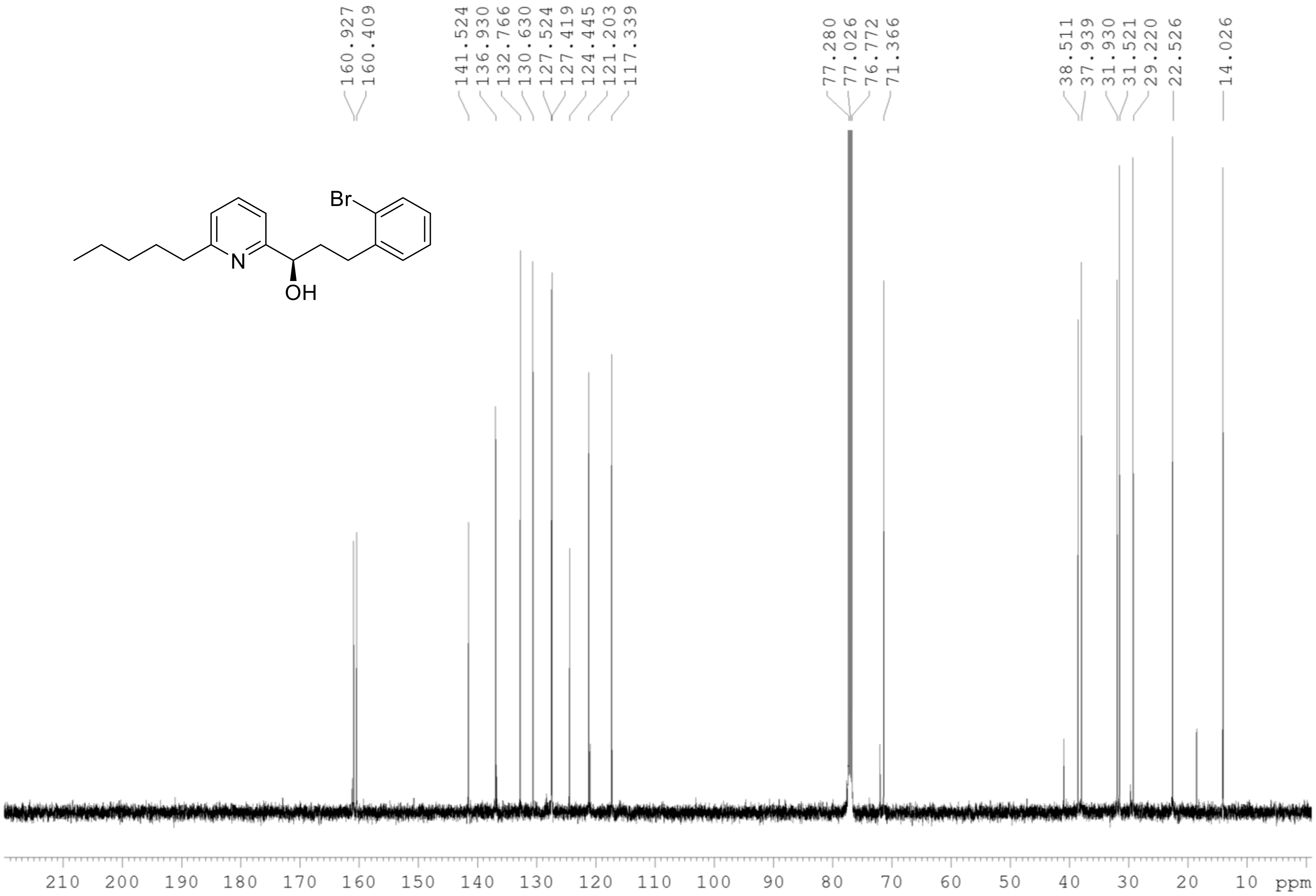

^{1}H NMR (400 MHz, $CDCl_3$) (*R*)-3-(2-methoxyphenyl)-1-(6-pentylpyridin-2-yl)propan-1-ol (6b)

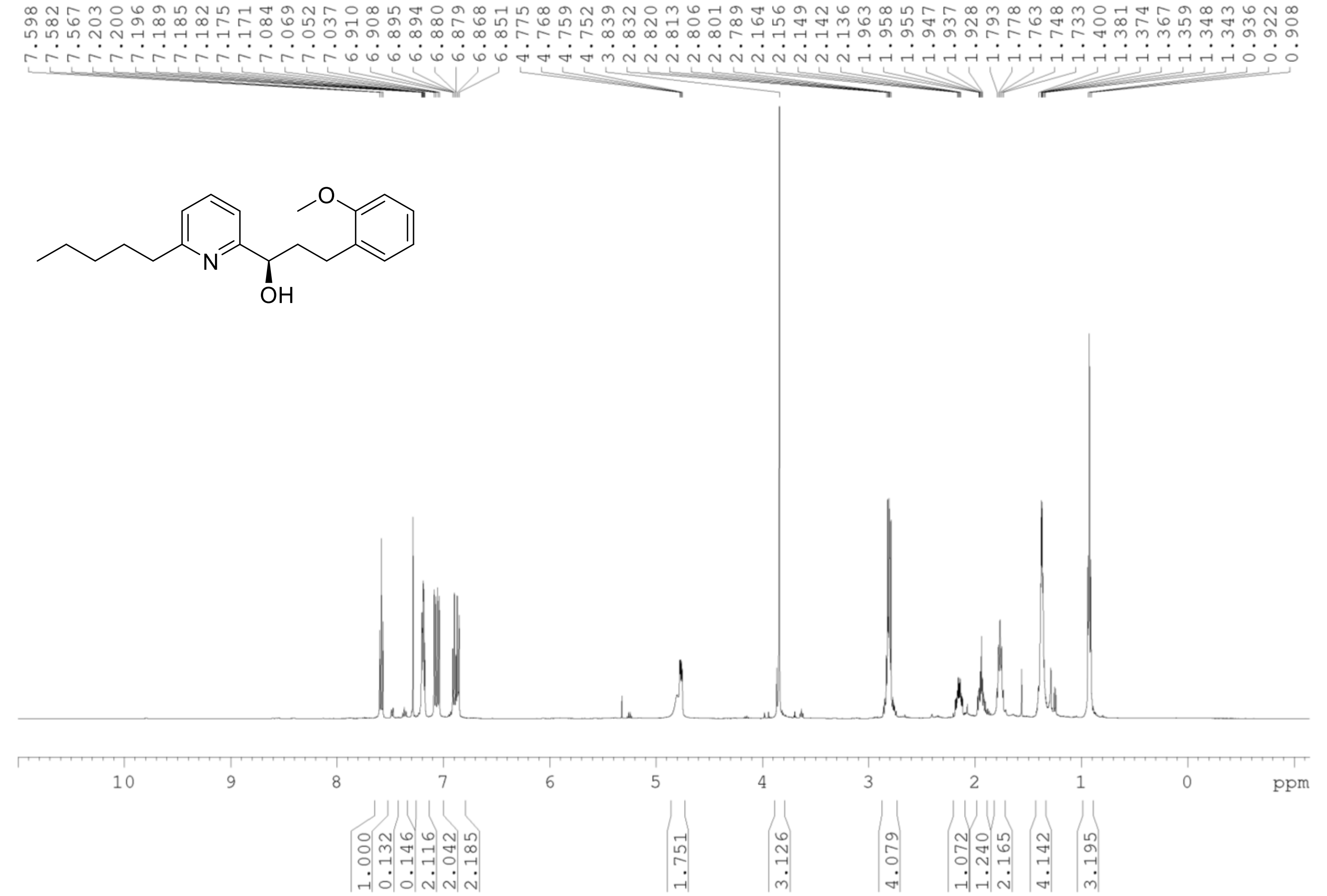

^{13}C NMR (101 MHz, CDCl$_3$) (*R*)-3-(2-methoxyphenyl)-1-(6-pentylpyridin-2-yl)propan-1-ol (6b)

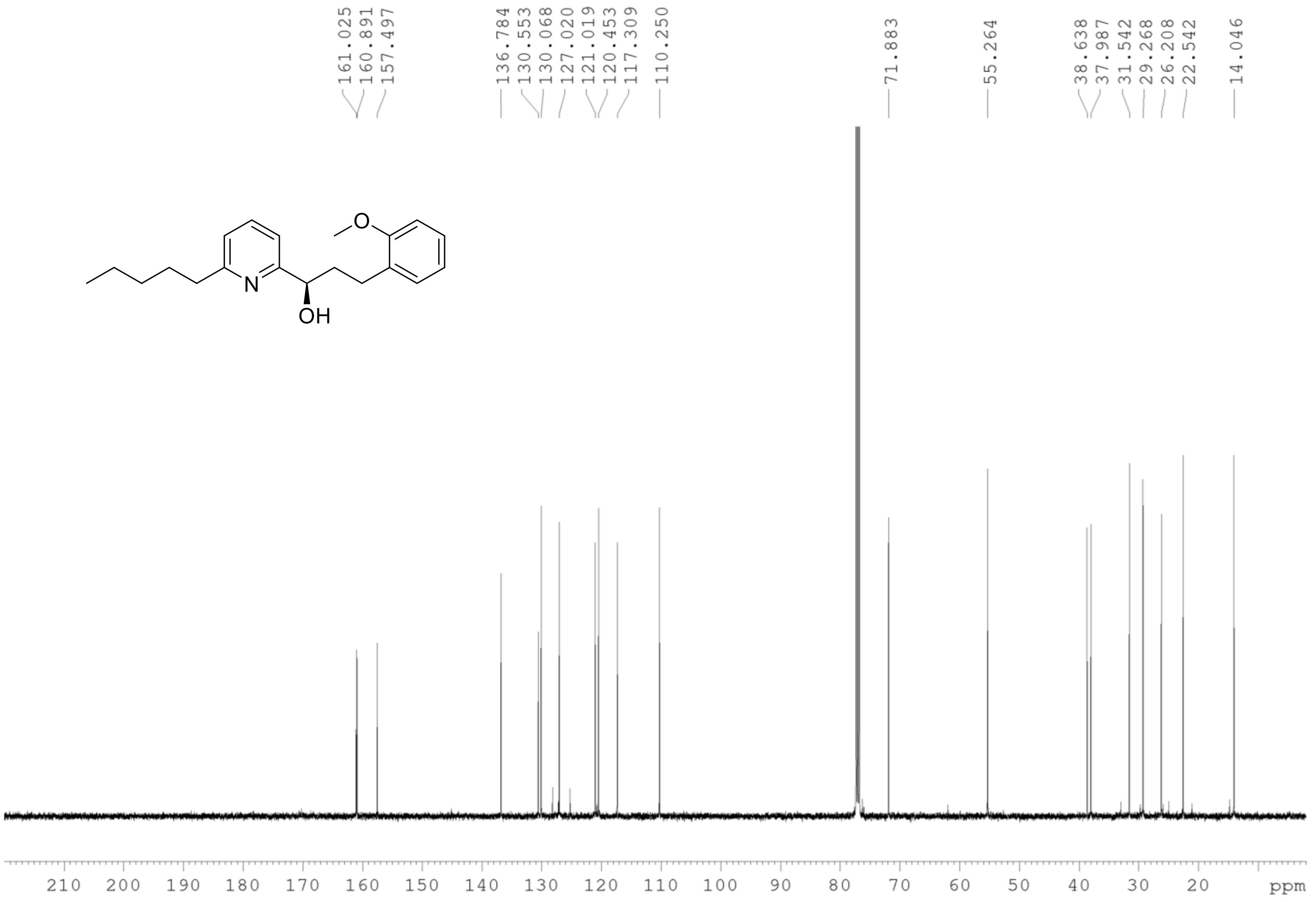

^{1}H NMR (400 MHz, $CDCl_3$) (*R*)-3-(4-bromophenyl)-1-(6-pentylpyridin-2-yl)propan-1-ol (6c)

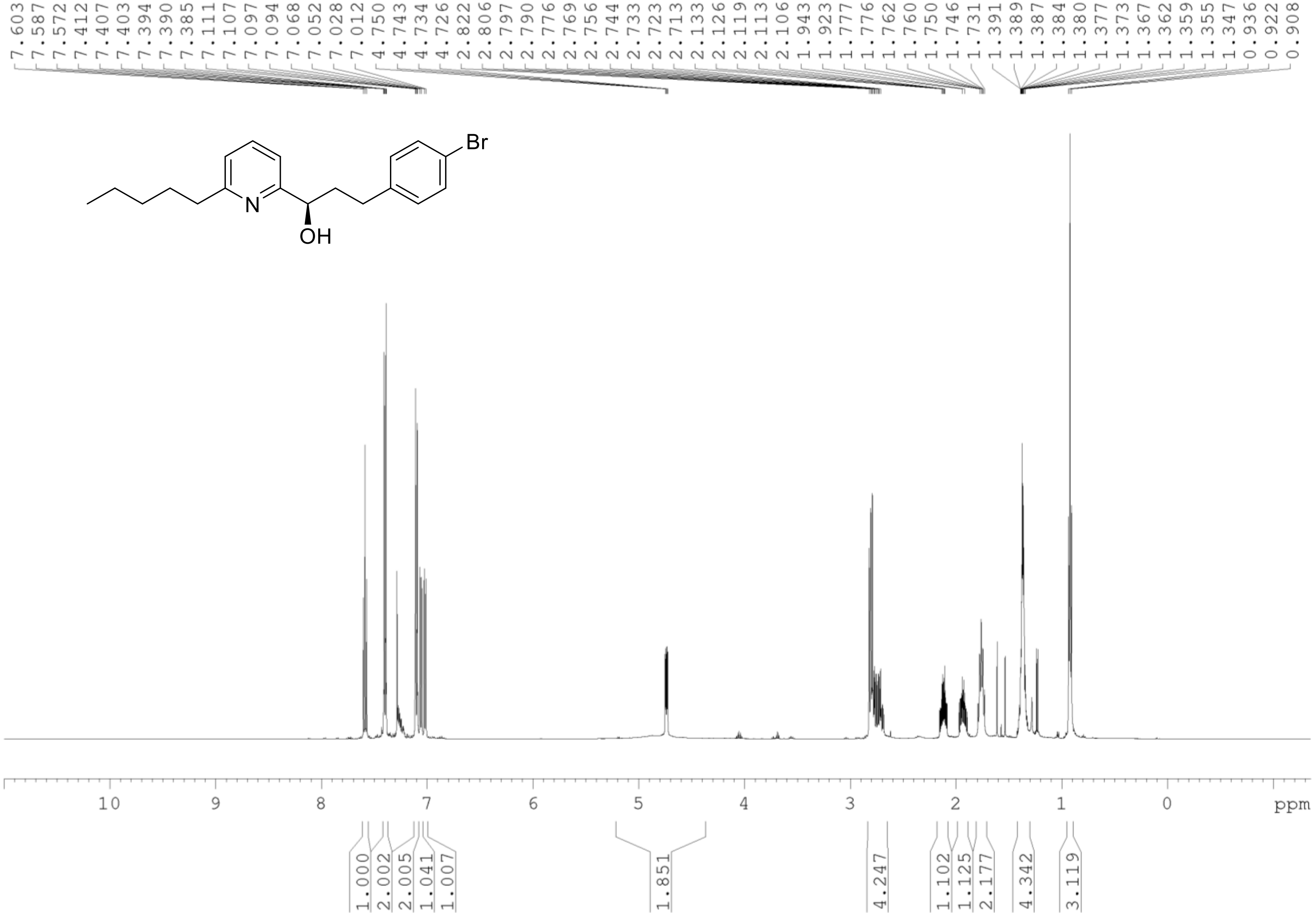

^{13}C NMR (101 MHz, $CDCl_3$) (*R*)-3-(4-bromophenyl)-1-(6-pentylpyridin-2-yl)propan-1-ol (6c)

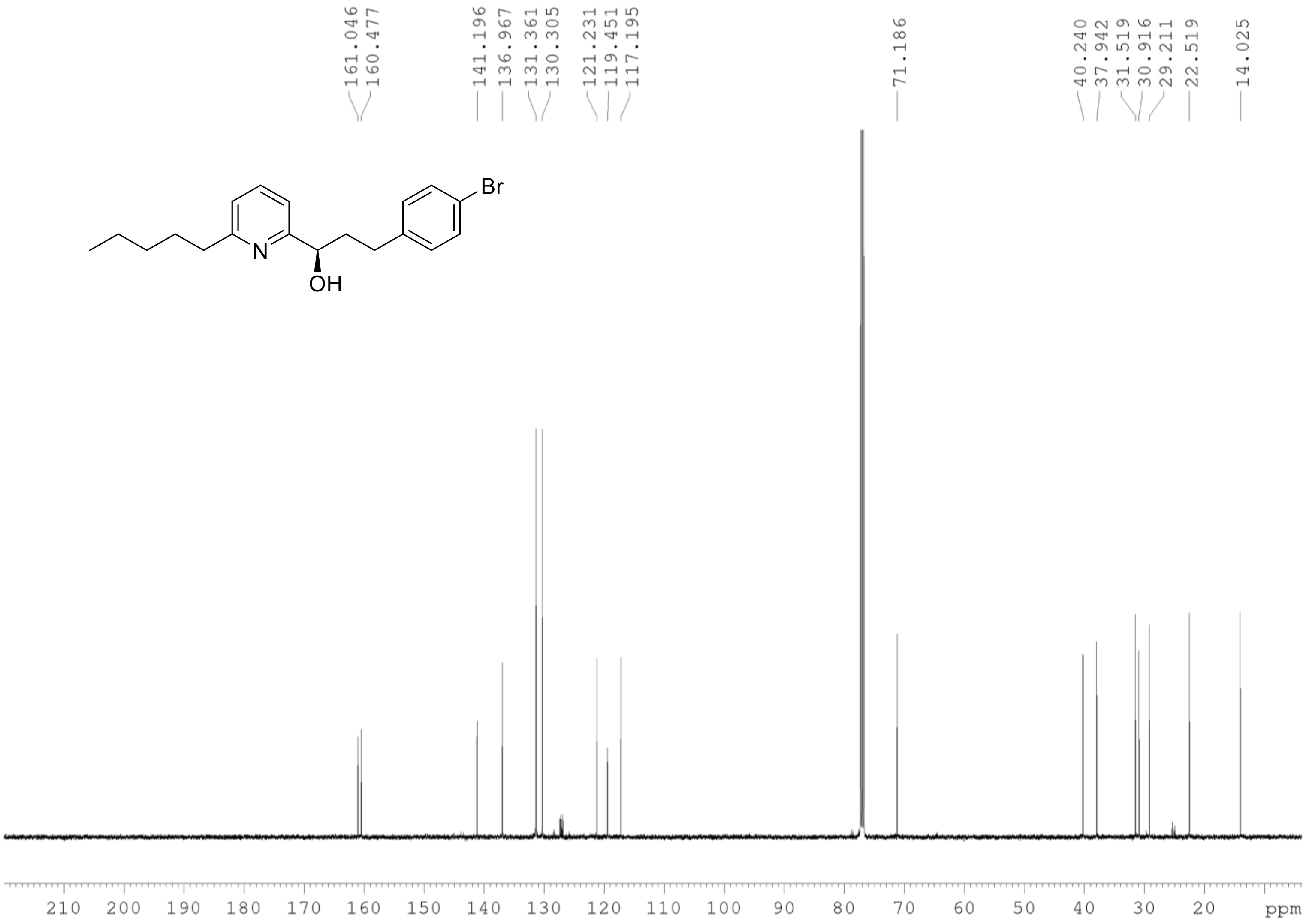

^{1}H NMR (400 MHz, $CDCl_3$) (*R*)-3-(2-fluorophenyl)-1-(6-pentylpyridin-2-yl)propan-1-ol (6d)

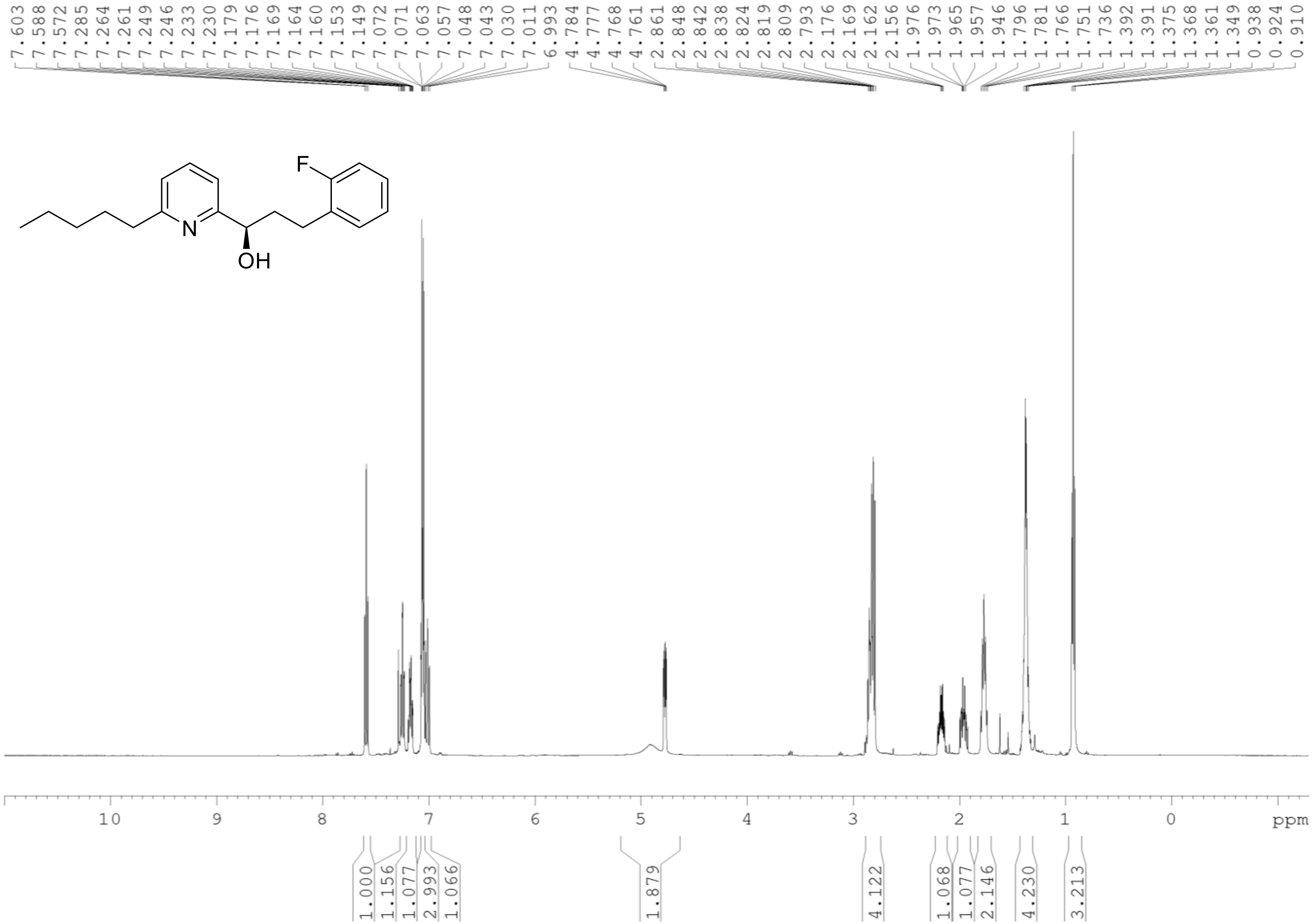

^{13}C NMR (101 MHz, CDCl$_3$) (*R*)-3-(2-fluorophenyl)-1-(6-pentylpyridin-2-yl)propan-1-ol (6d)

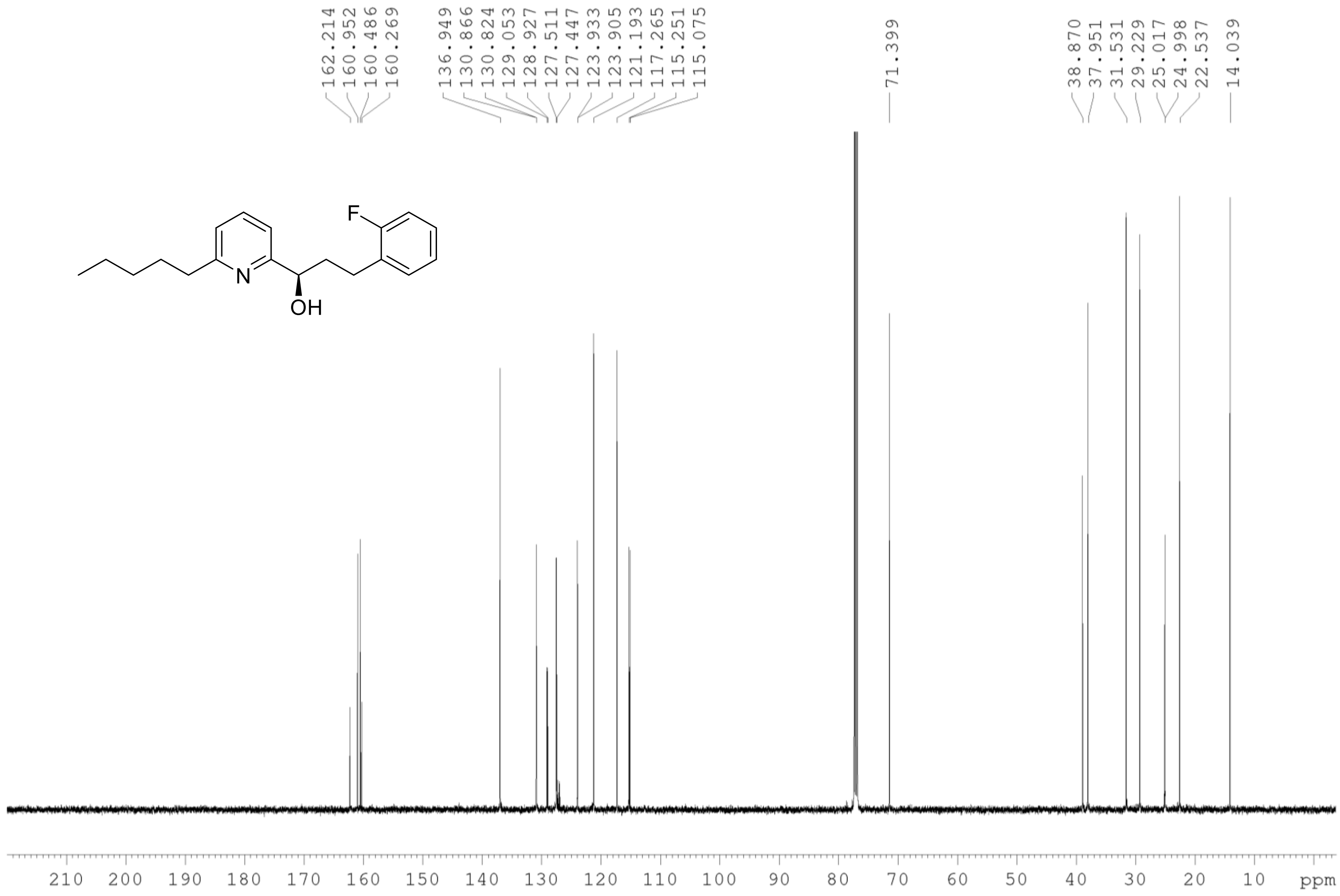

^{19}F NMR (176 MHz, $CDCl_3$) (*R*)-3-(2-fluorophenyl)-1-(6-pentylpyridin-2-yl)propan-1-ol (6d)

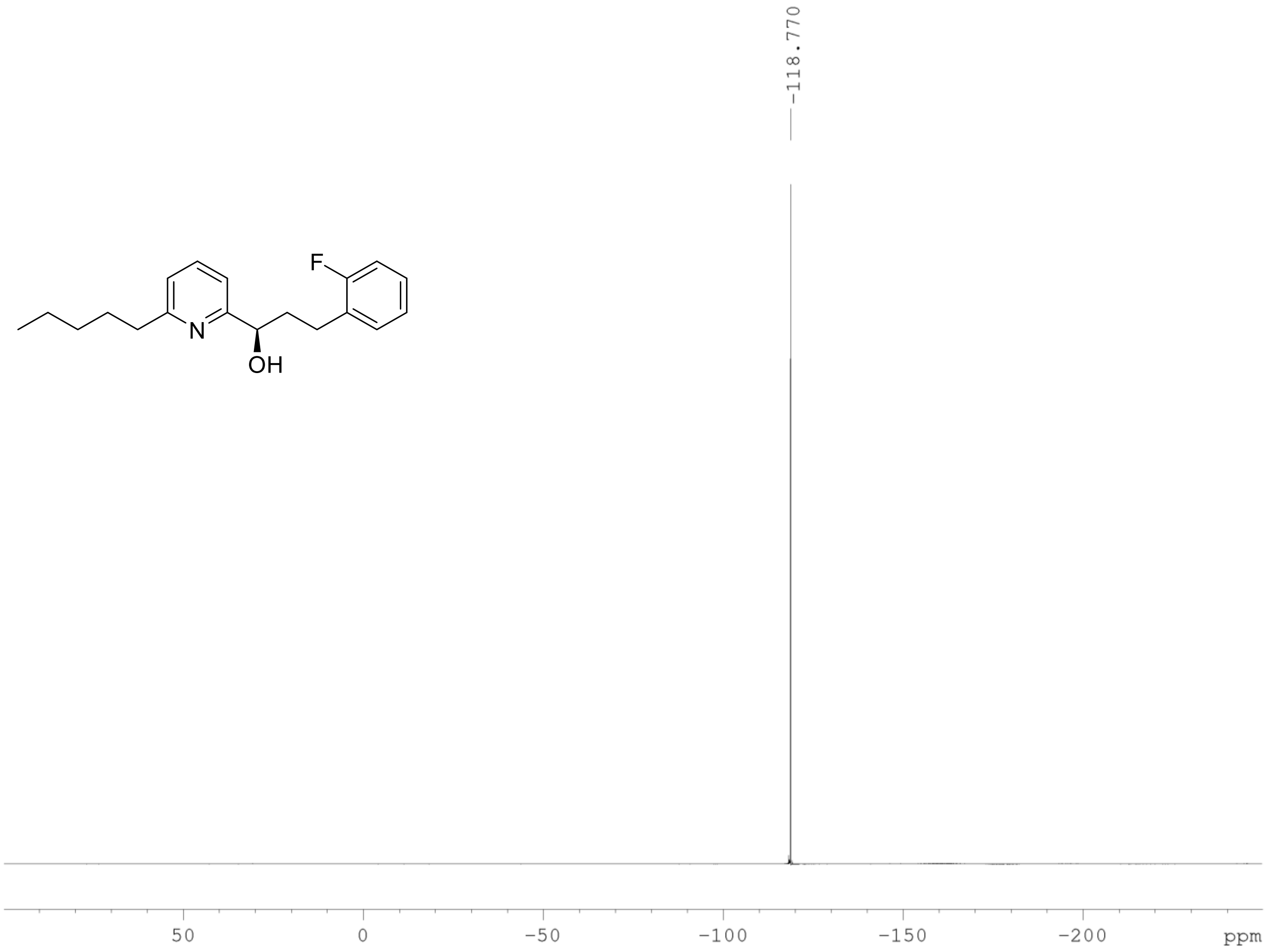

^{1}H NMR (400 MHz, $CDCl_3$) (*R*)-2-(2-fluorophenyl)-1-(6-pentylpyridin-2-yl)ethan-1-ol (6e)

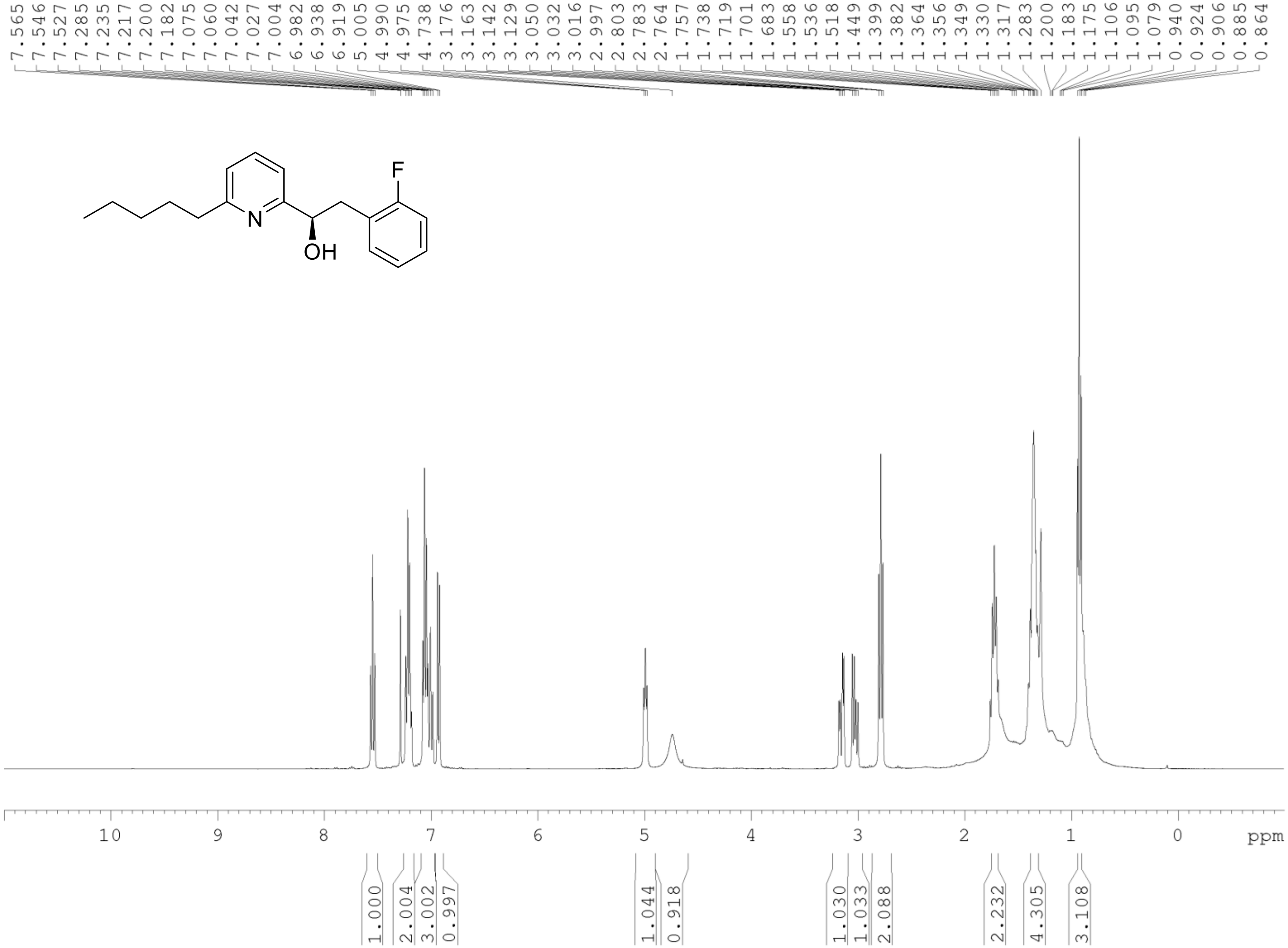

^{13}C NMR (101 MHz, $CDCl_3$) (*R*)-2-(2-fluorophenyl)-1-(6-pentylpyridin-2-yl)ethan-1-ol (6e)

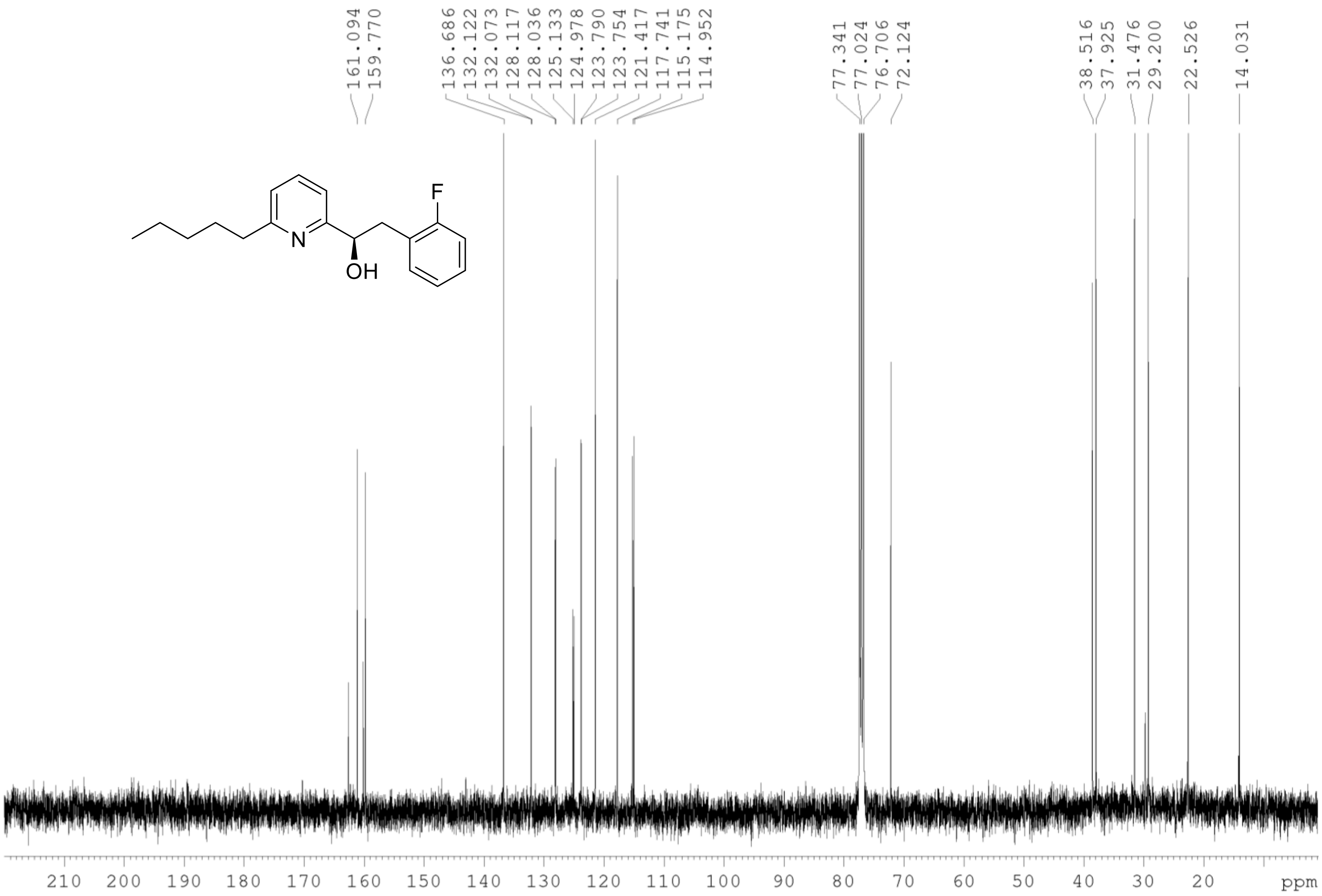

^{19}F NMR (176 MHz, $CDCl_3$) (*R*)-2-(2-fluorophenyl)-1-(6-pentylpyridin-2-yl)ethan-1-ol (6e)

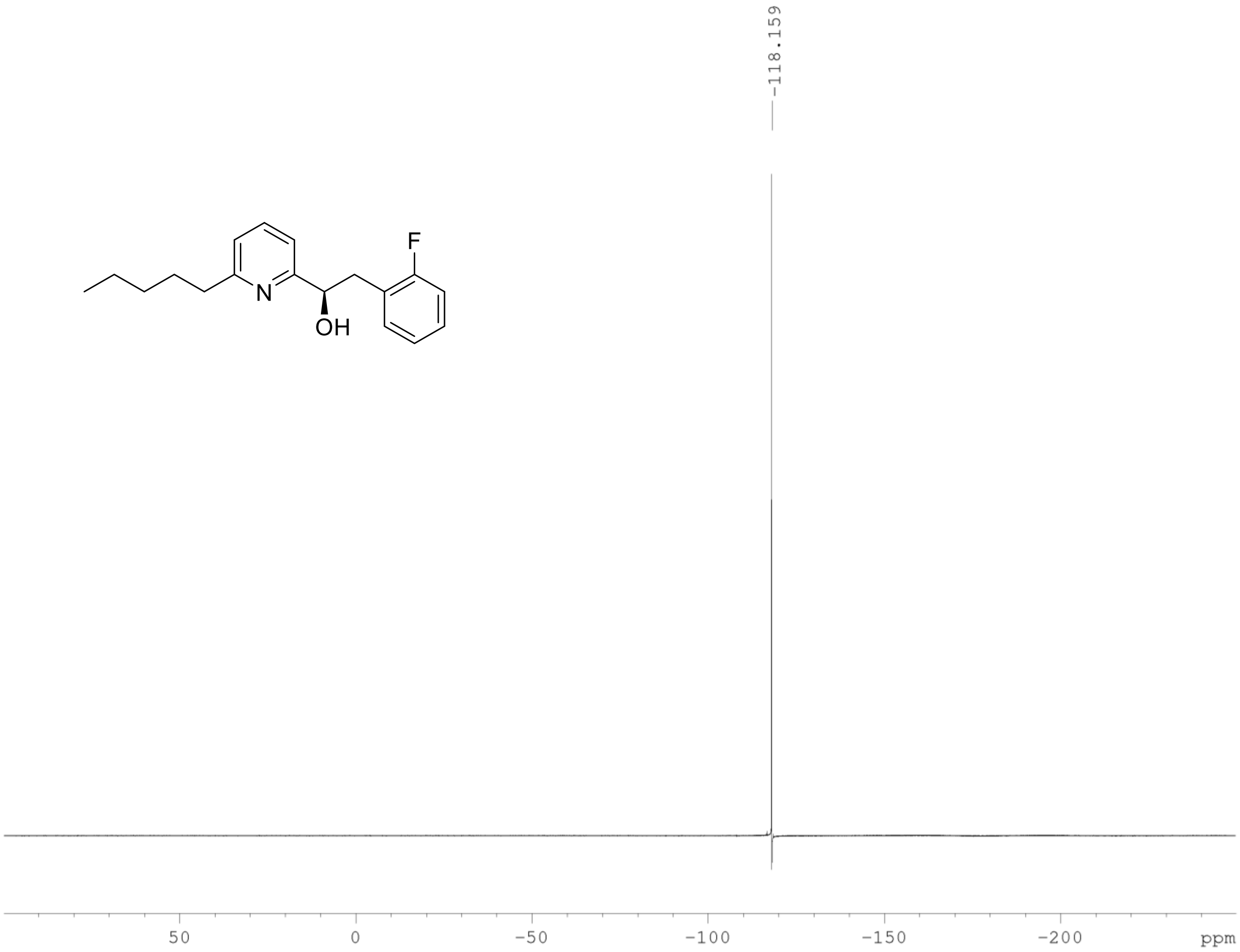

^{1}H NMR (400 MHz, $CDCl_3$) (*R*)-2-(4-Bromophenyl)-1-(6-pentylpyridin-2-yl)ethan-1-ol (6f)

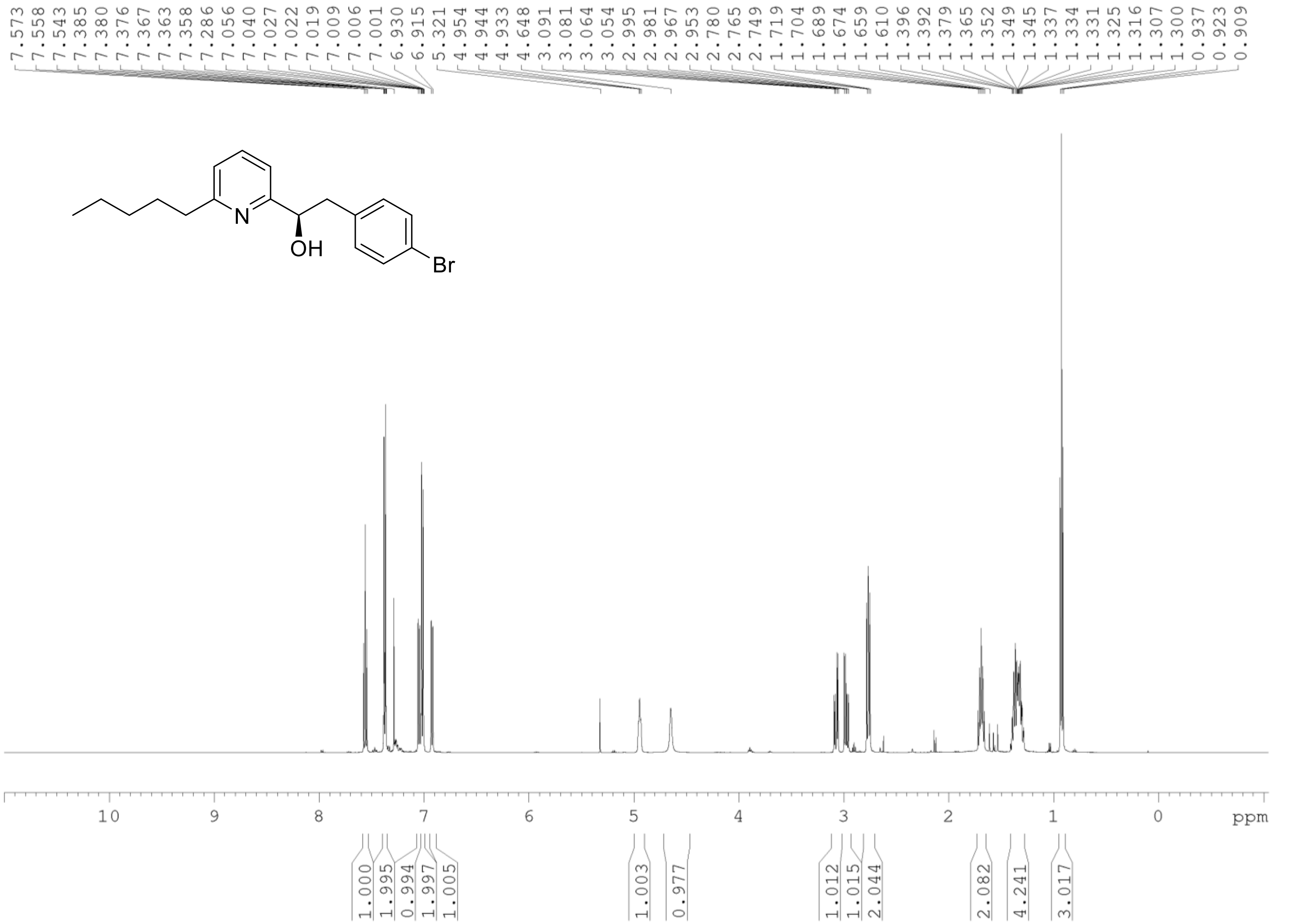

^{13}C NMR (101 MHz, CDCl$_3$) (*R*)-2-(4-Bromophenyl)-1-(6-pentylpyridin-2-yl)ethan-1-ol (6f)

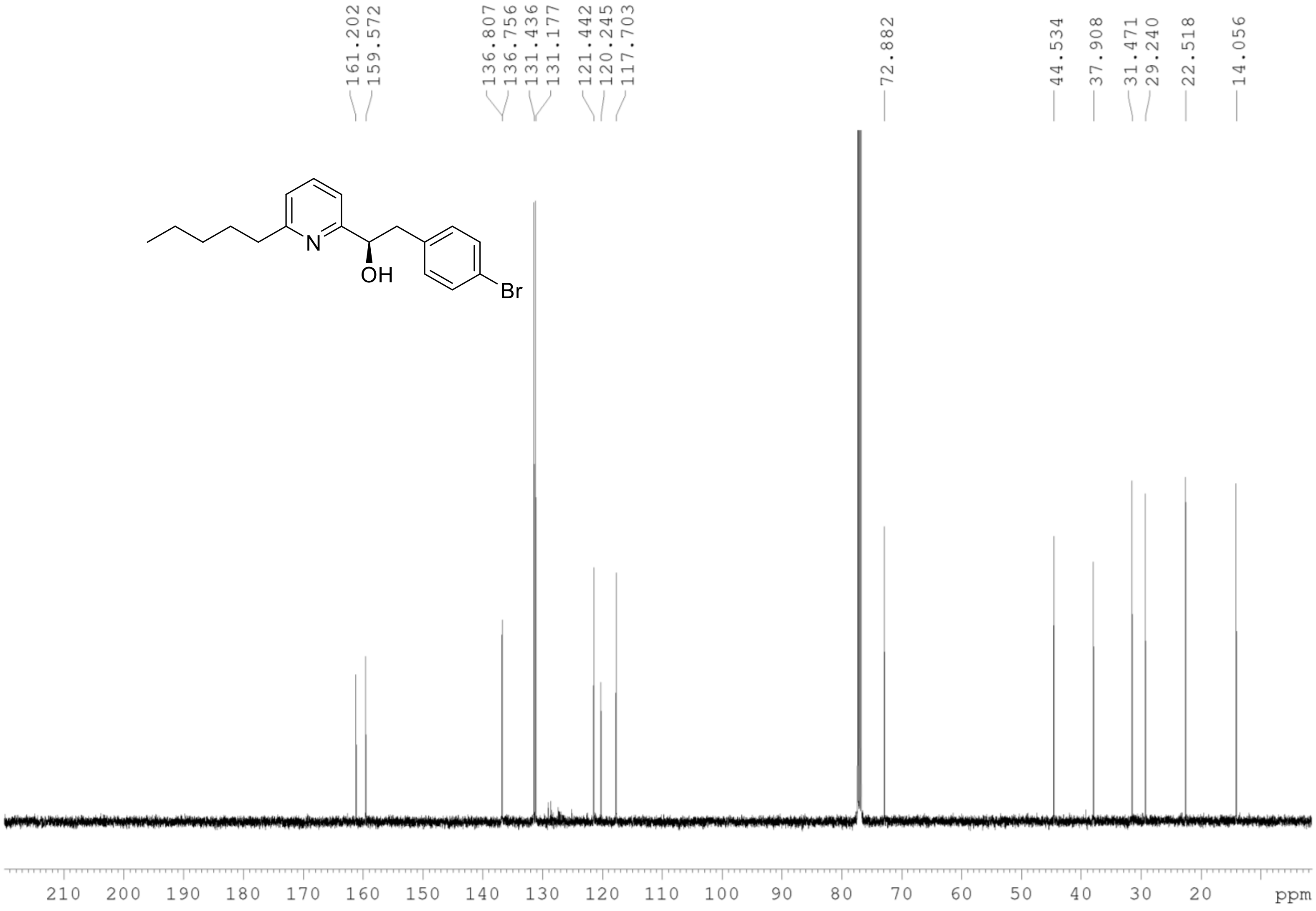

^{1}H NMR (400 MHz, $CDCl_3$) (*R*)-2-(2-tolyl)-1-(6-pentylpyridin-2-yl)ethan-1-ol (6g)

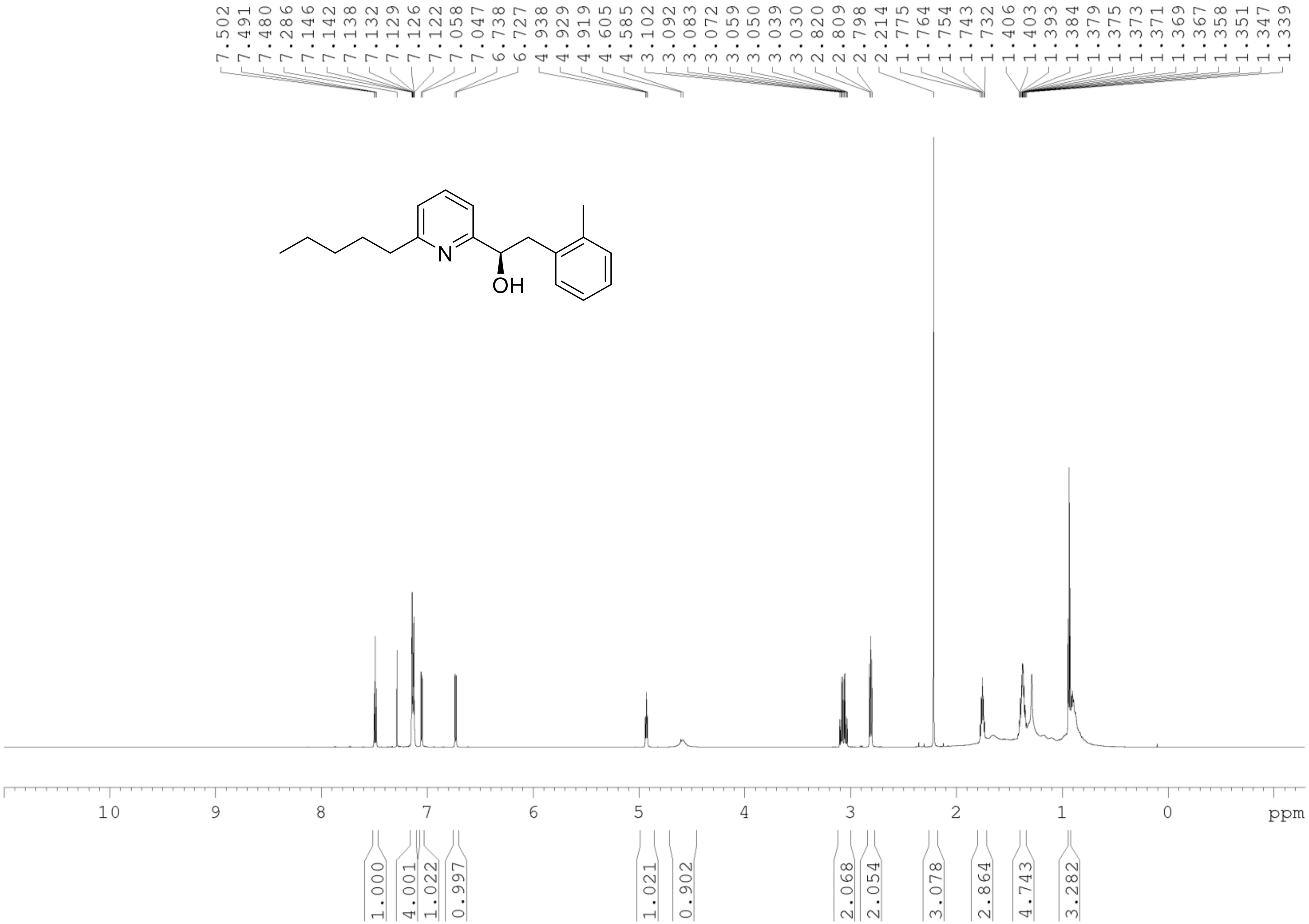

^{13}C NMR (101 MHz, $CDCl_3$) (*R*)-2-(2-tolyl)-1-(6-pentylpyridin-2-yl)ethan-1-ol (6g)

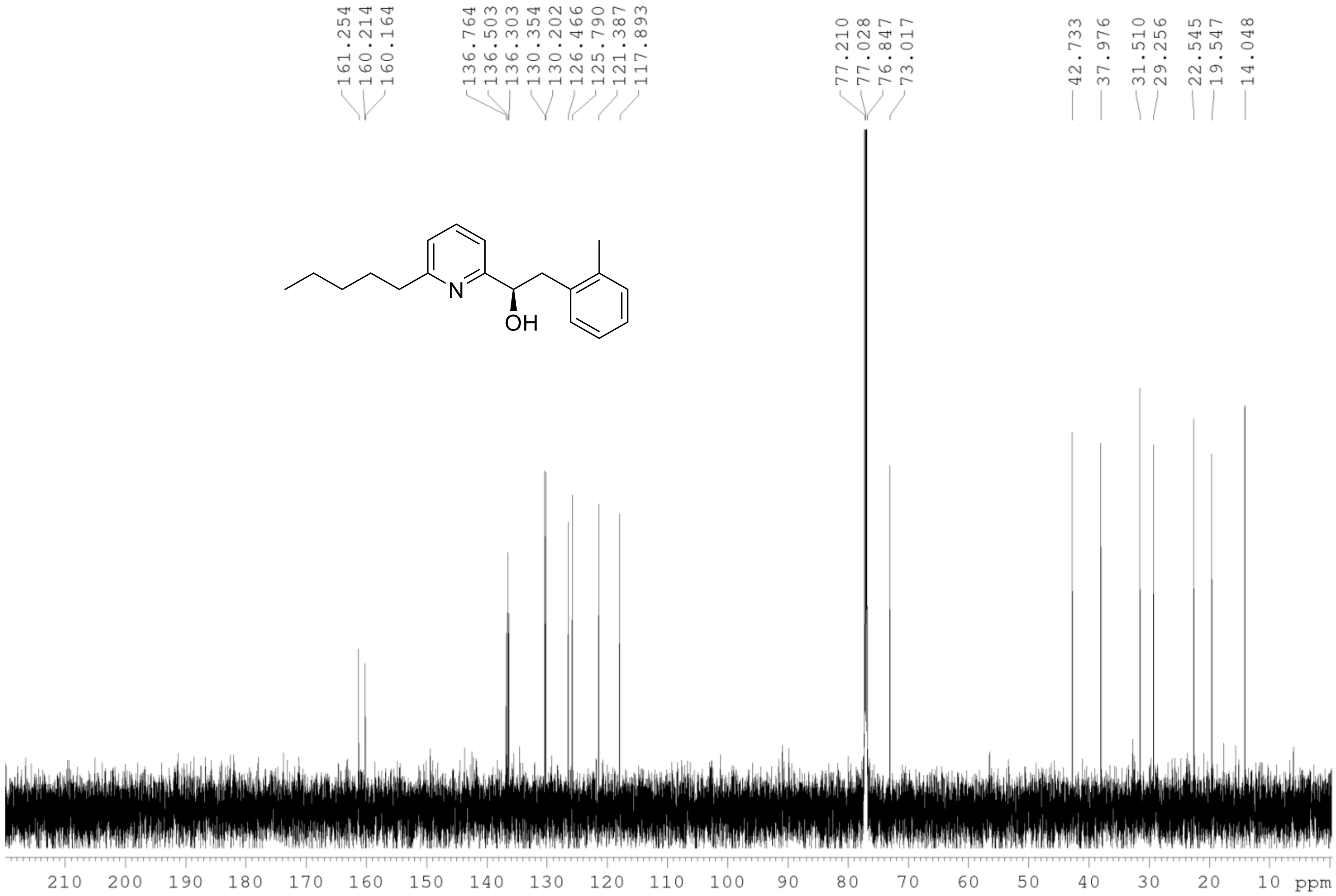

^{1}H NMR (400 MHz, CDCl$_3$) (*R*)-2-(4-Methoxyphenyl)-1-(6-pentylpyridin-2-yl)ethan-1-ol (6h)

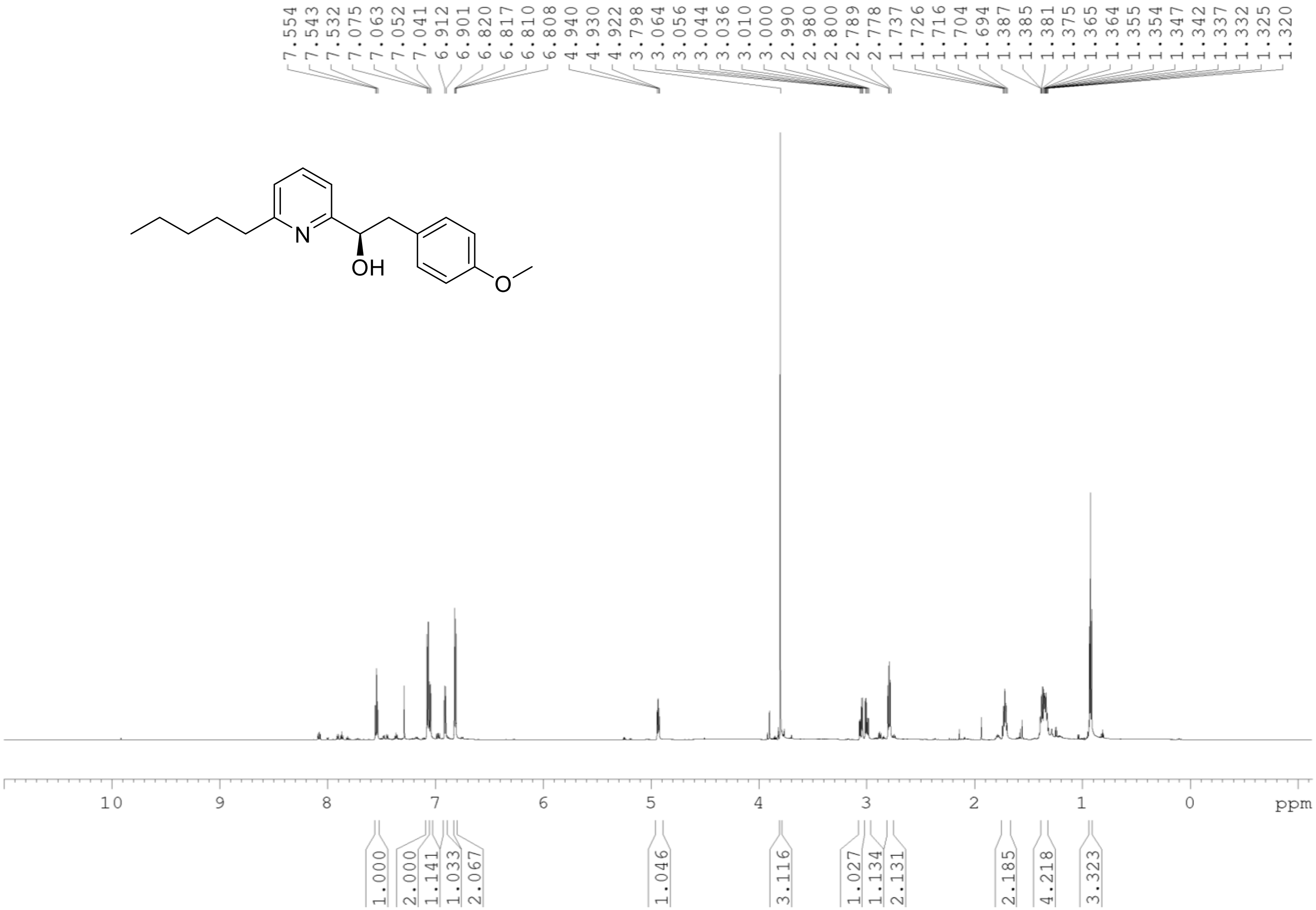

^{13}C NMR (101 MHz, CDCl$_3$) (*R*)-2-(4-Methoxyphenyl)-1-(6-pentylpyridin-2-yl)ethan-1-ol (6h)

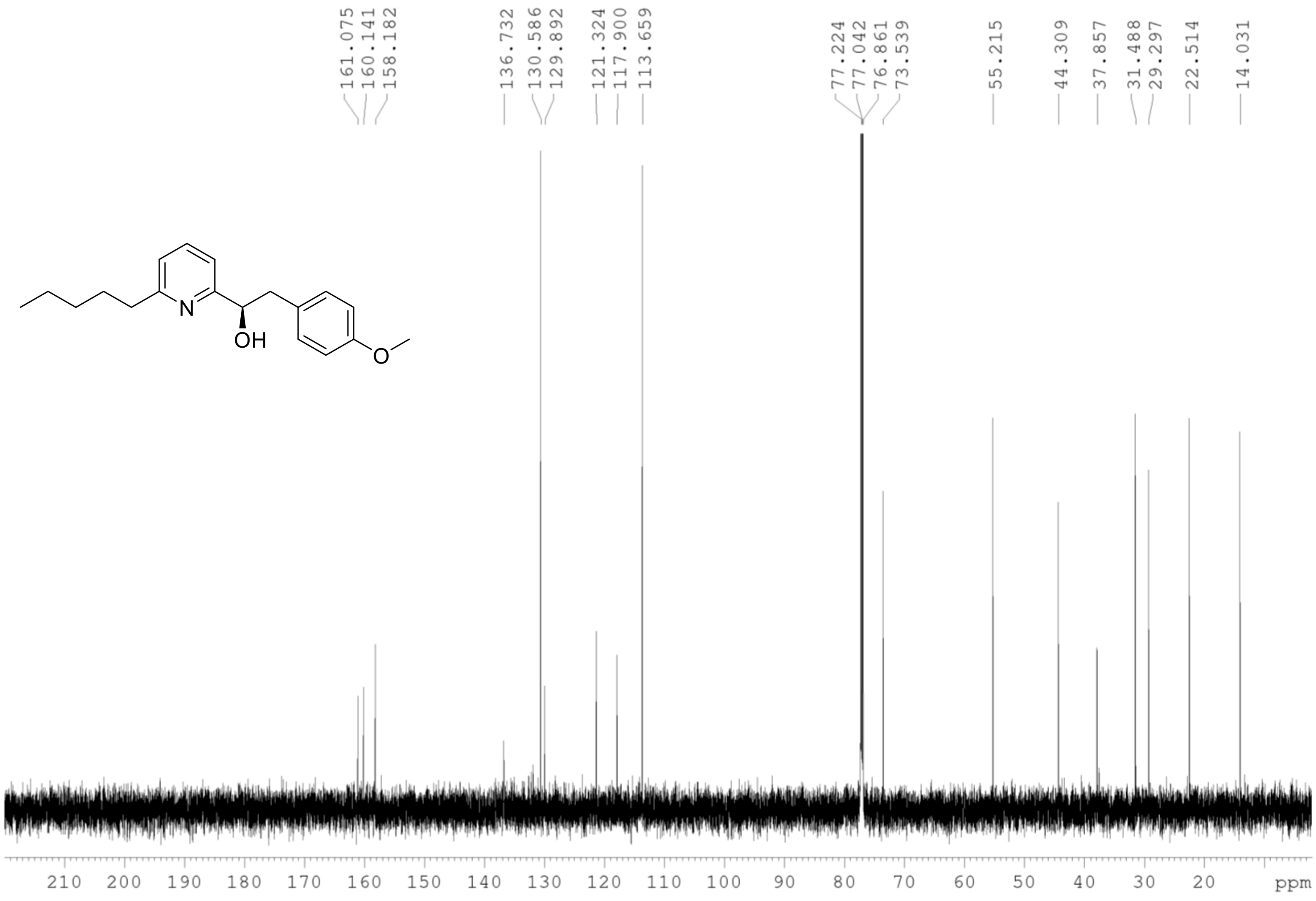

^1H NMR (400 MHz, $CDCl_3$) (*R*)-1-(6-pentylpyridin-2-yl)-4-phenylbutan-1-ol (6i)

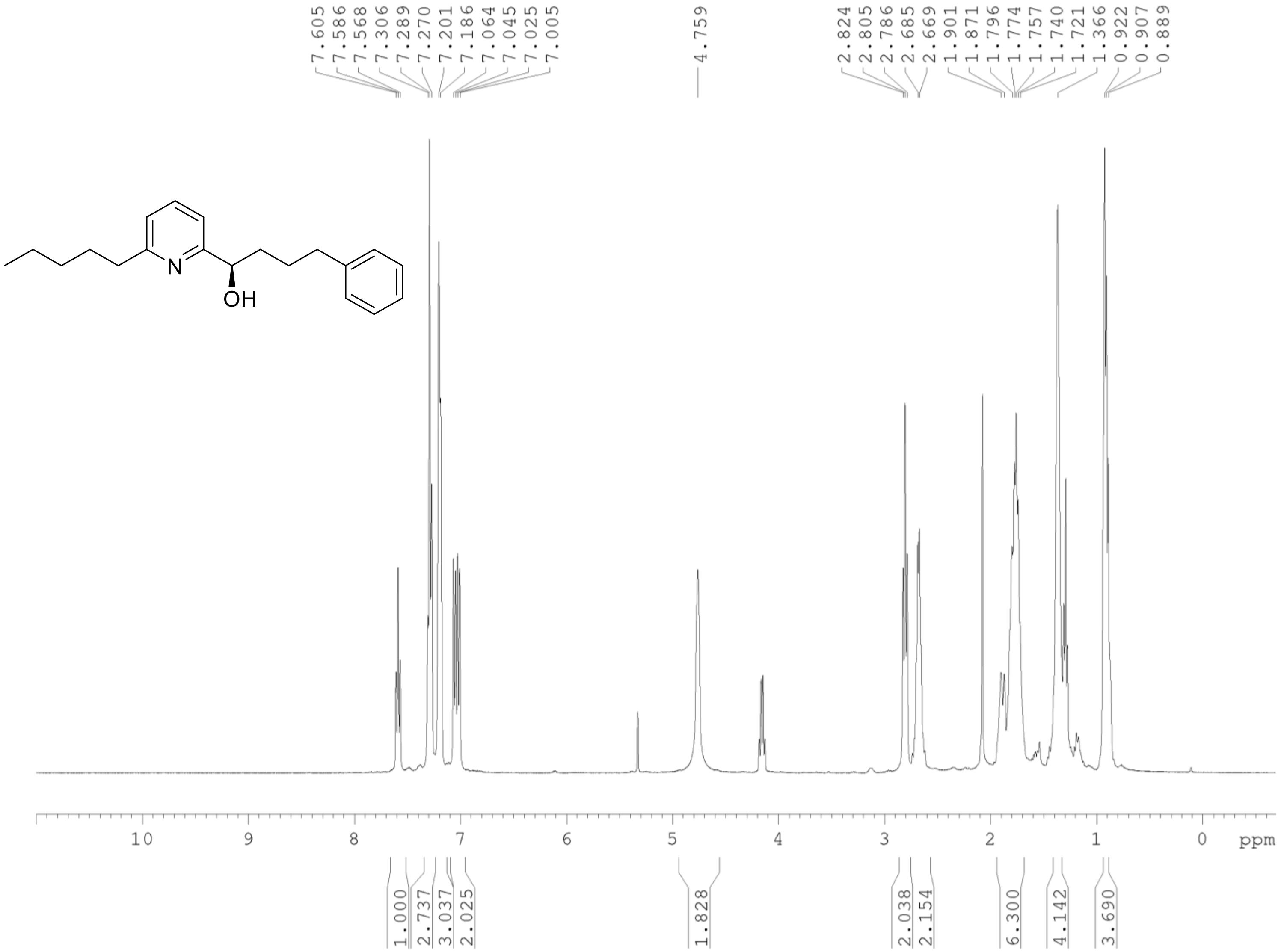

^{13}C NMR (101 MHz, $CDCl_3$) (*R*)-1-(6-pentylpyridin-2-yl)-4-phenylbutan-1-ol (6i)

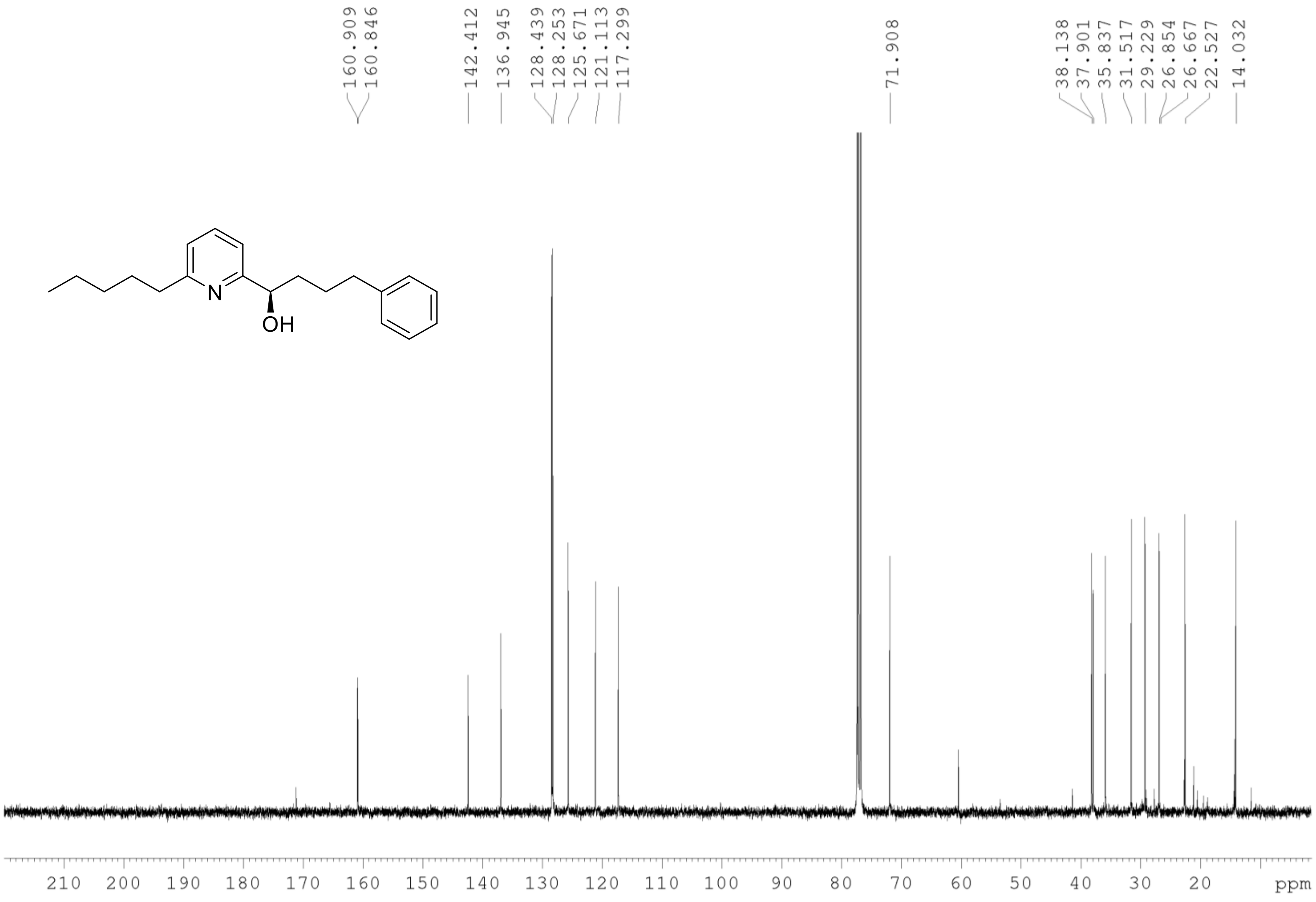

^{1}H NMR (400 MHz, $CDCl_3$) (*R*)-1-(6-pentylpyridin-2-yl)-4-phenylbutan-1-yl acetate (6ia)

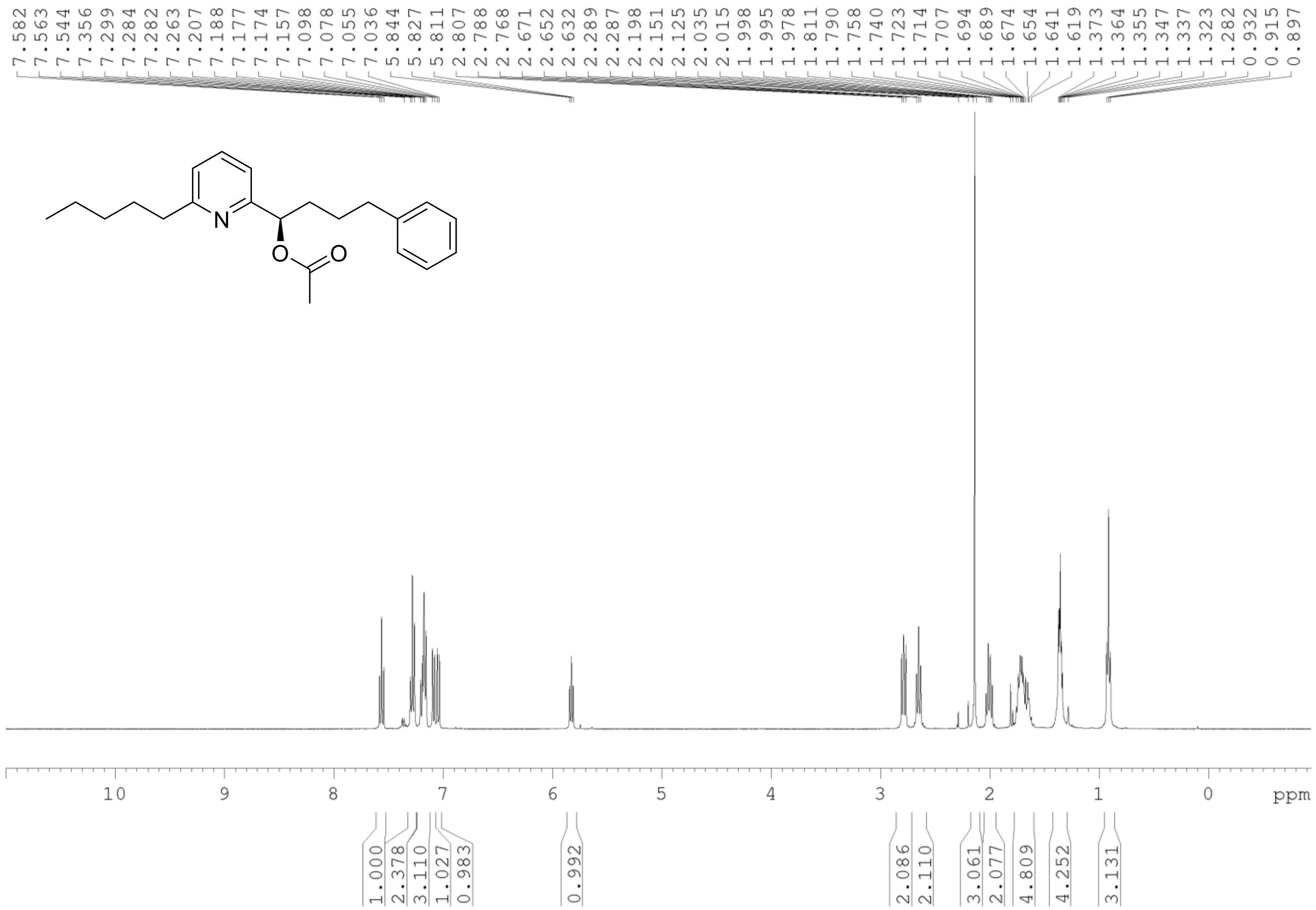

^{13}C NMR (101 MHz, $CDCl_3$) (*R*)-1-(6-pentylpyridin-2-yl)-4-phenylbutan-1-yl acetate (6ia)

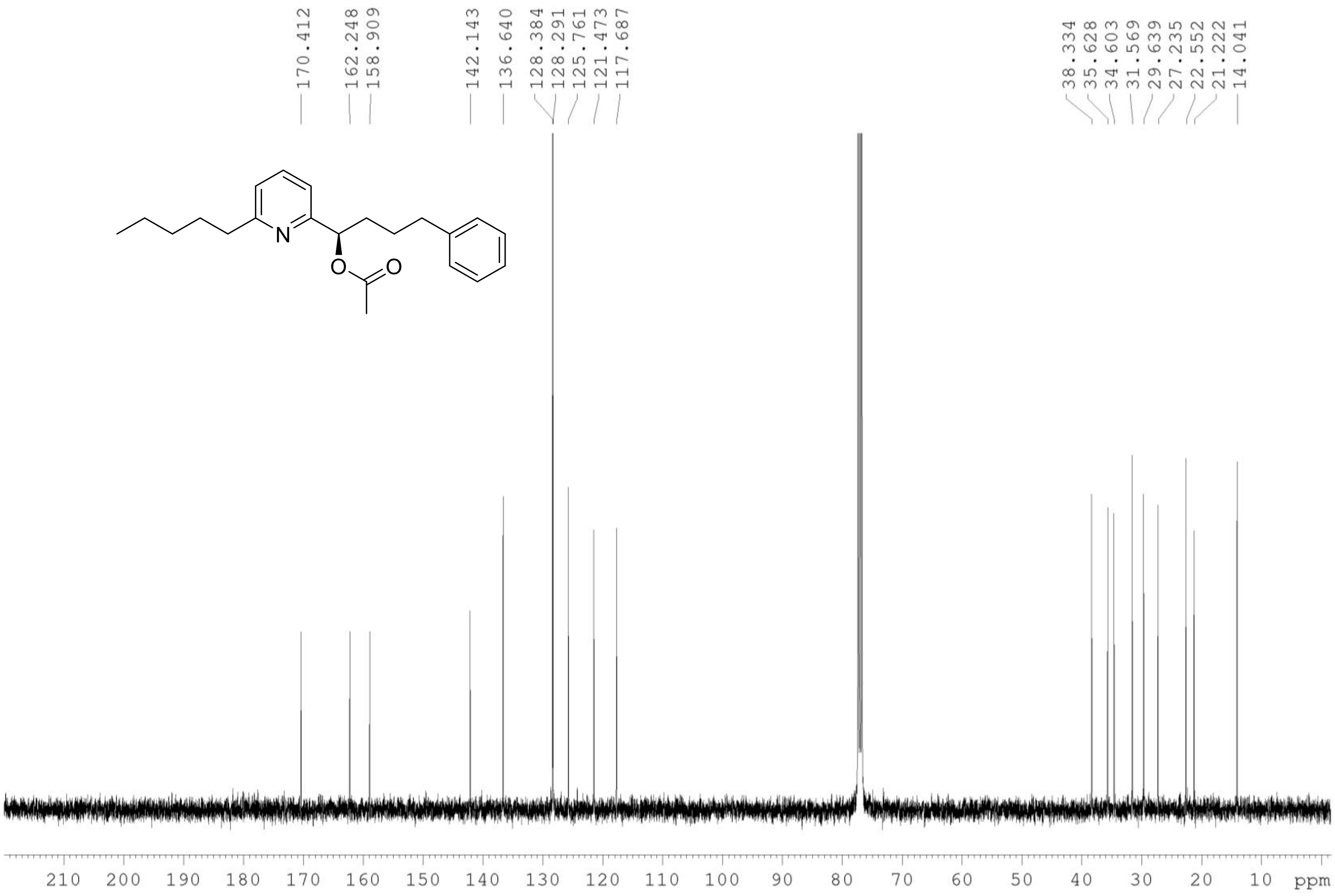

^{1}H NMR (400 MHz, CDCl$_3$) (*R*)-1-(6-pentylpyridin-2-yl)butan-1-ol (6j)

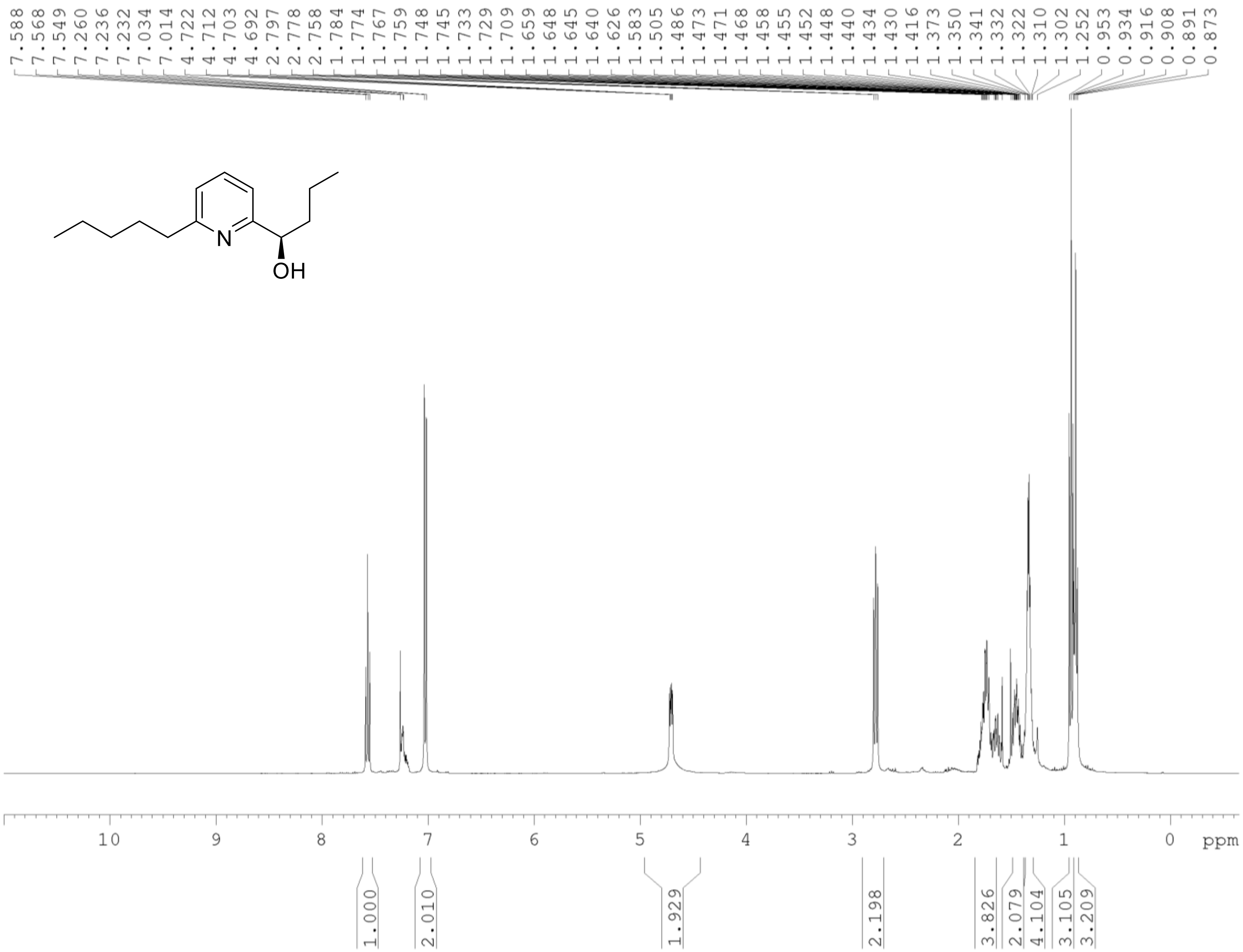

^{13}C NMR (101 MHz, $CDCl_3$) (*R*)-1-(6-pentylpyridin-2-yl)butan-1-ol (6j)

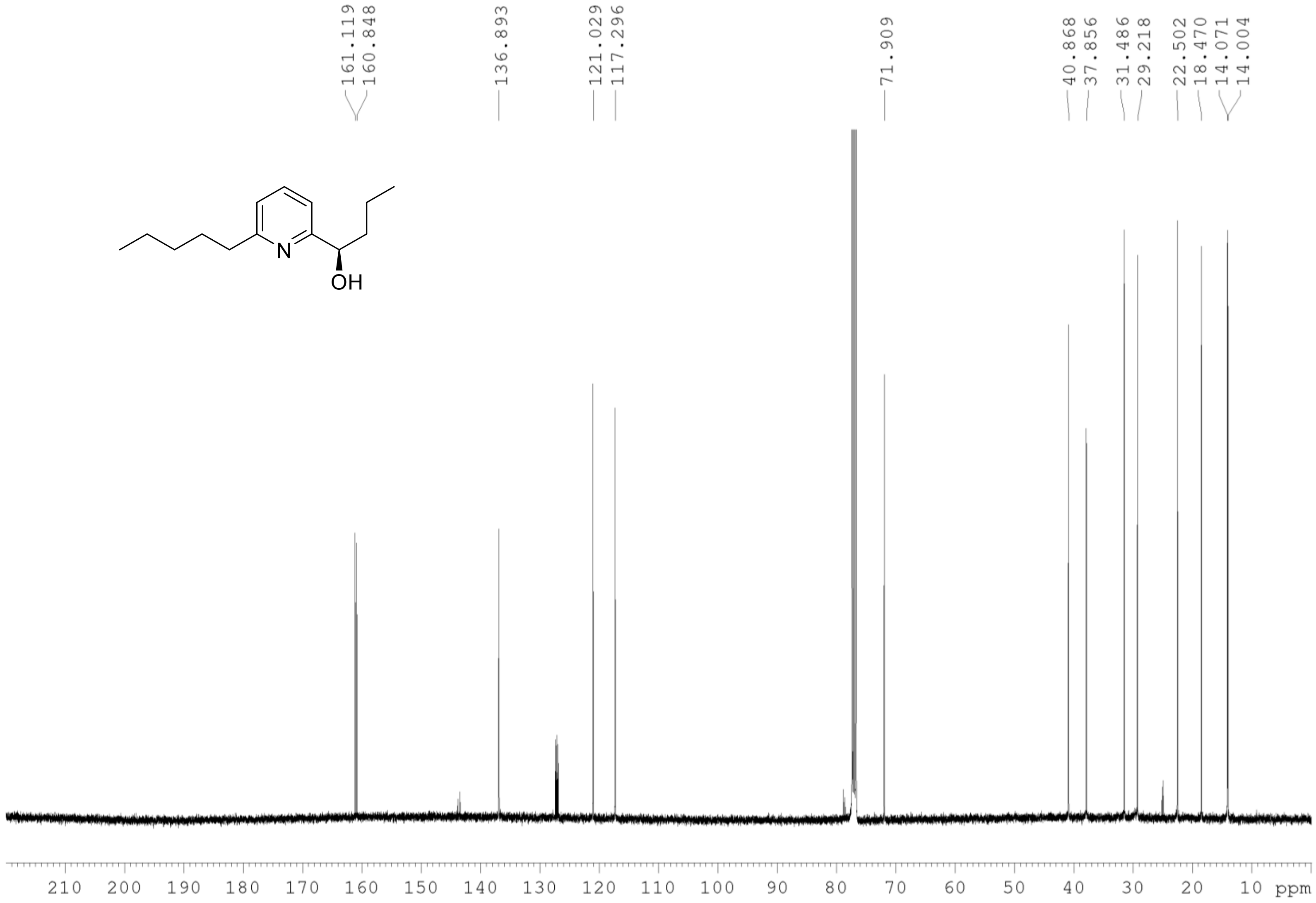

^{1}H NMR (400 MHz, $CDCl_3$) (*R*)-3-cyclohexyl-1-(6-pentylpyridin-2-yl)propan-1-ol (6k)

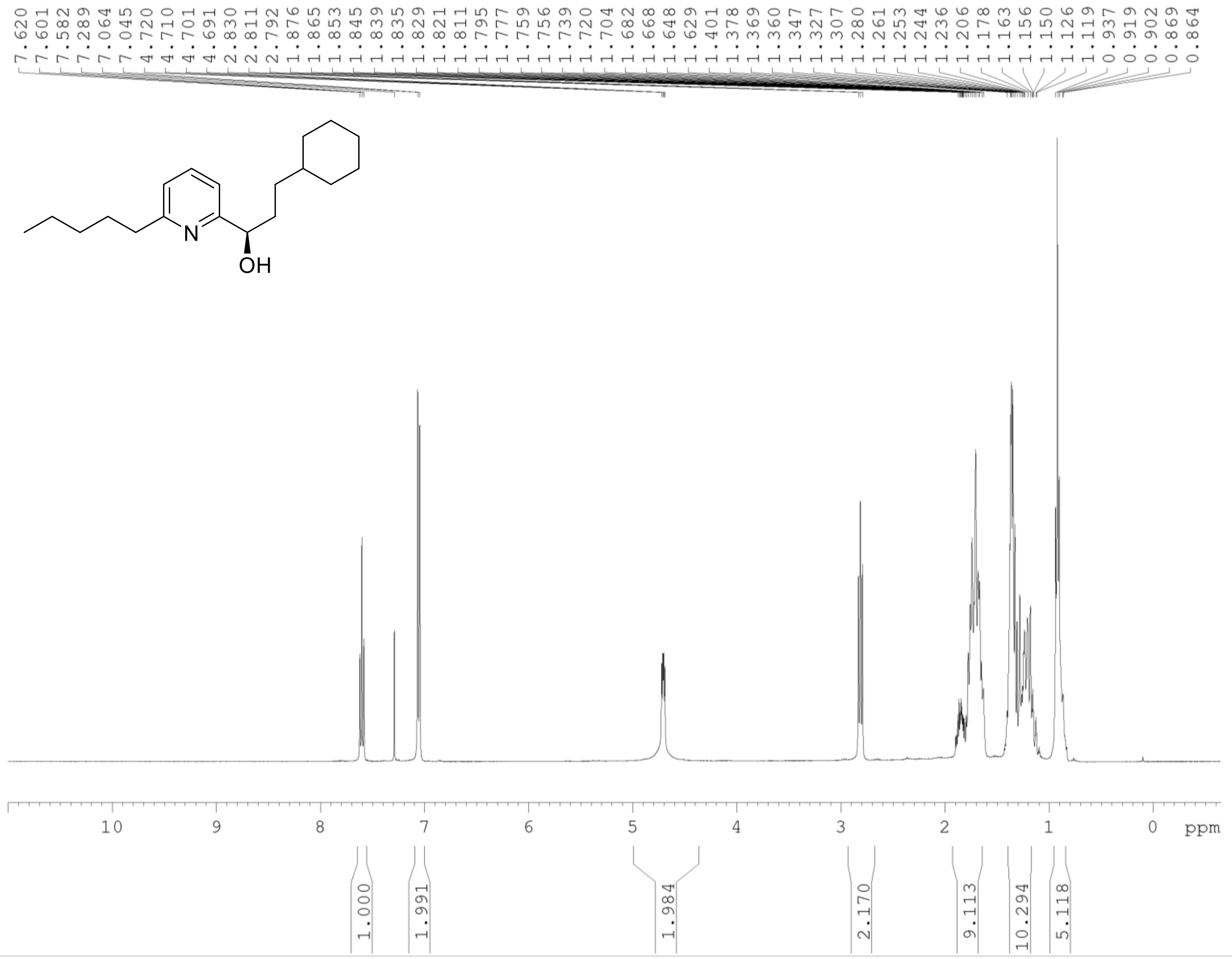

^{13}C NMR (101 MHz, $CDCl_3$) (*R*)-3-cyclohexyl-1-(6-pentylpyridin-2-yl)propan-1-ol (6k)

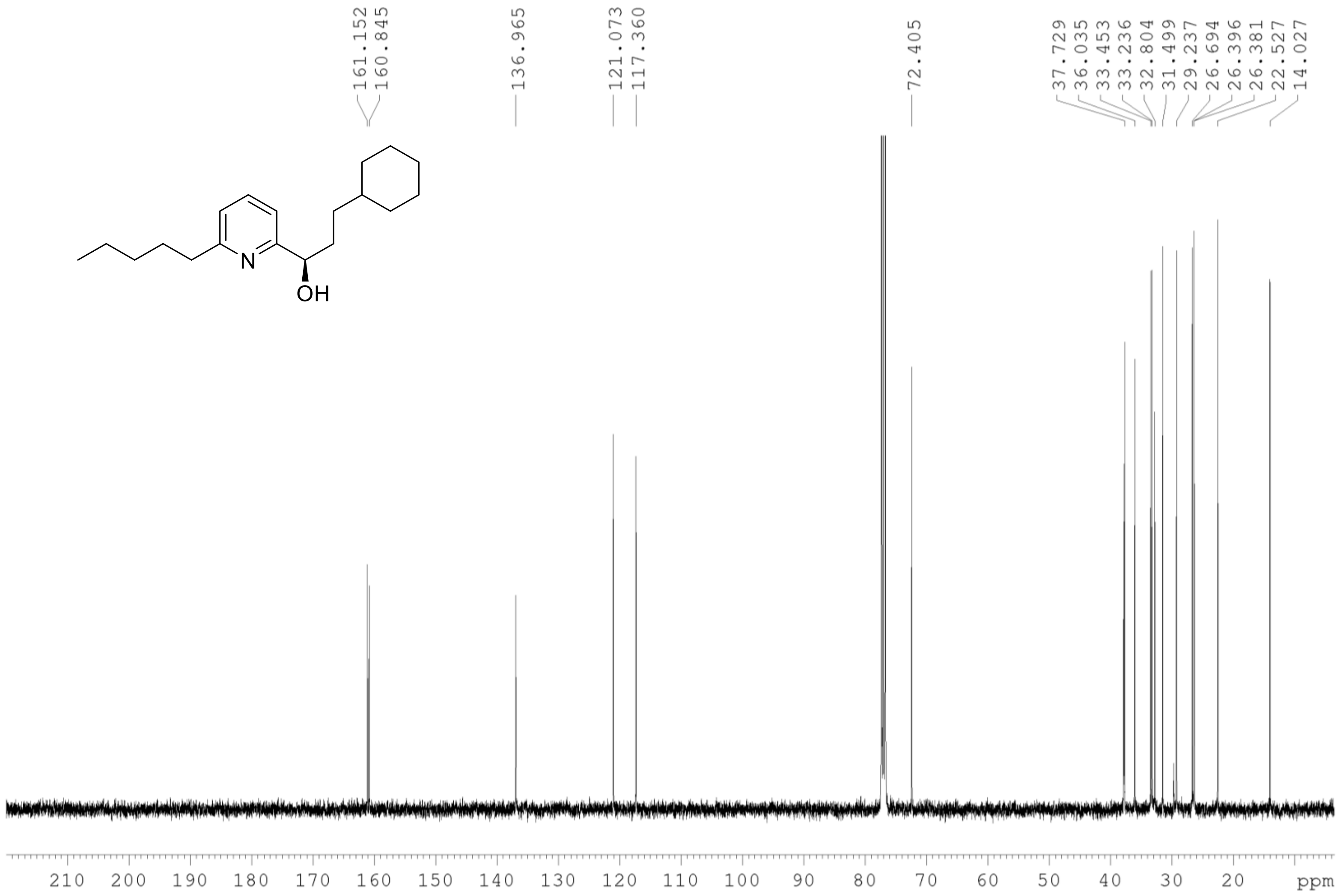

^{1}H NMR (400 MHz, $CDCl_3$) (*R*)-cyclobutyl(6-pentylpyridin-2-yl)methanol (6l)

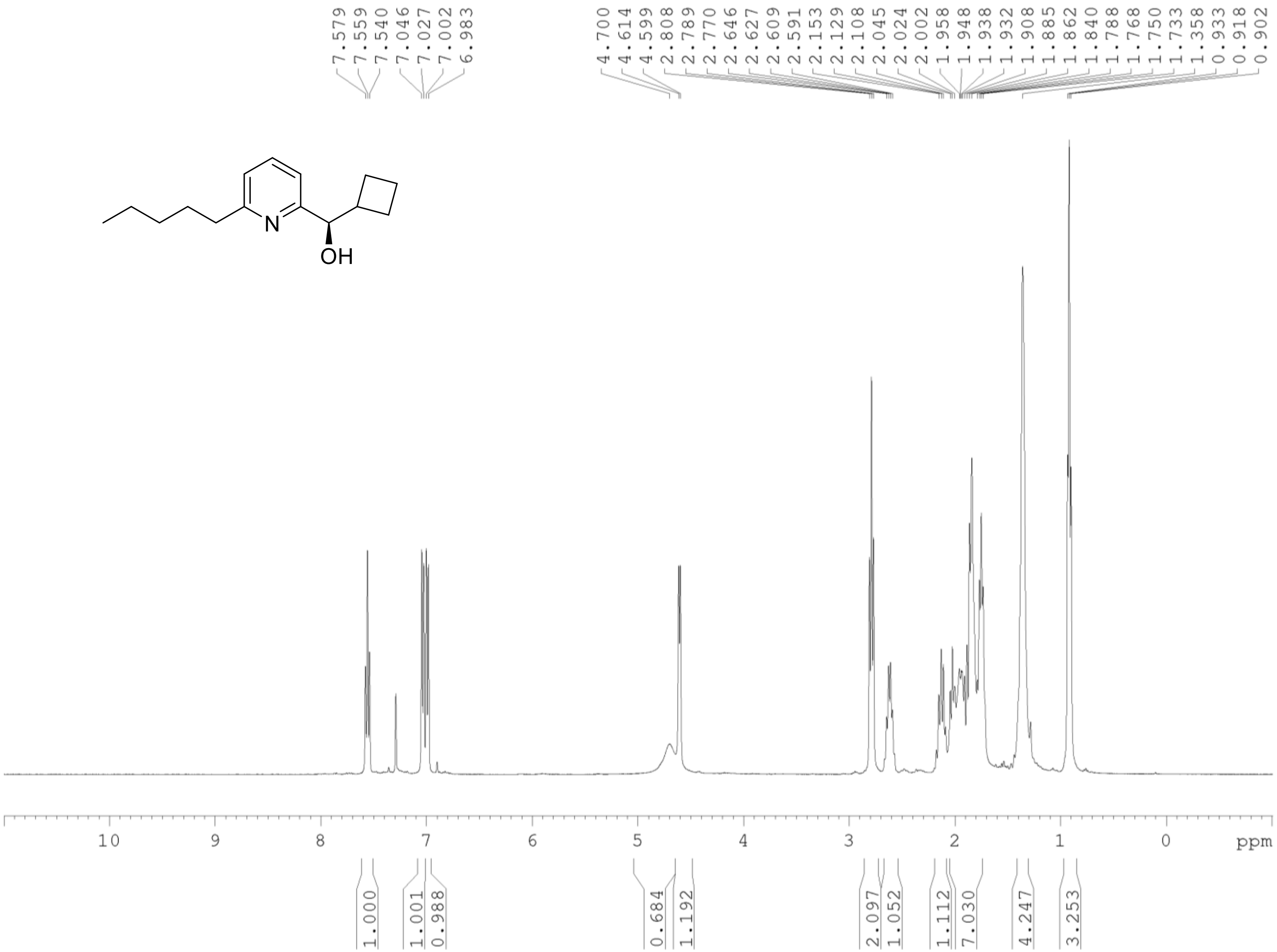

^{13}C NMR (101 MHz, $CDCl_3$) (*R*)-cyclobutyl(6-pentylpyridin-2-yl)methanol (6l)

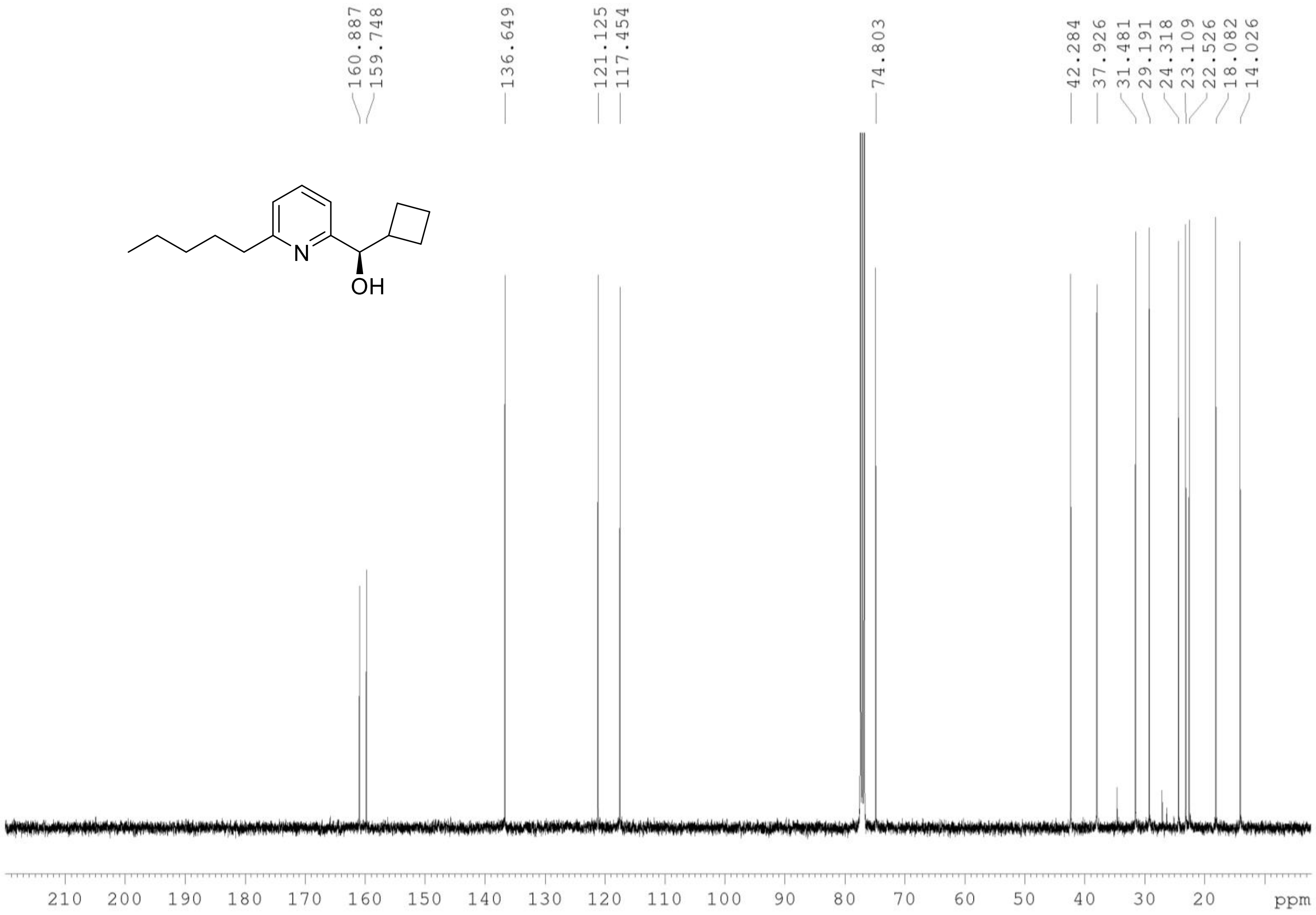

^{1}H NMR (400 MHz, $CDCl_3$) (*R*)-1-(6-pentylpyridin-2-yl)but-3-yn-1-ol (6m)

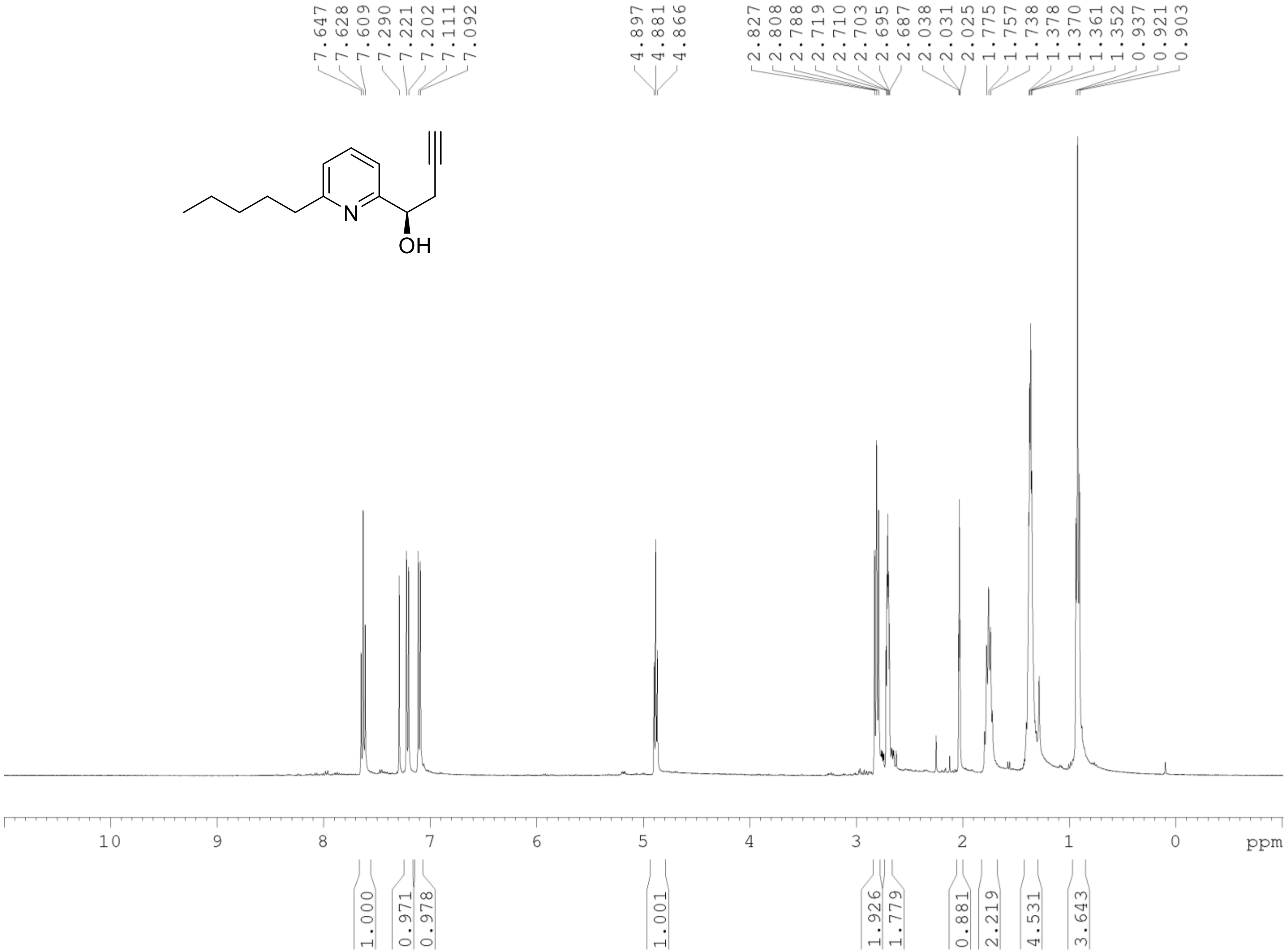

^{13}C NMR (101 MHz, $CDCl_3$) (*R*)-1-(6-pentylpyridin-2-yl)but-3-yn-1-ol (6m)

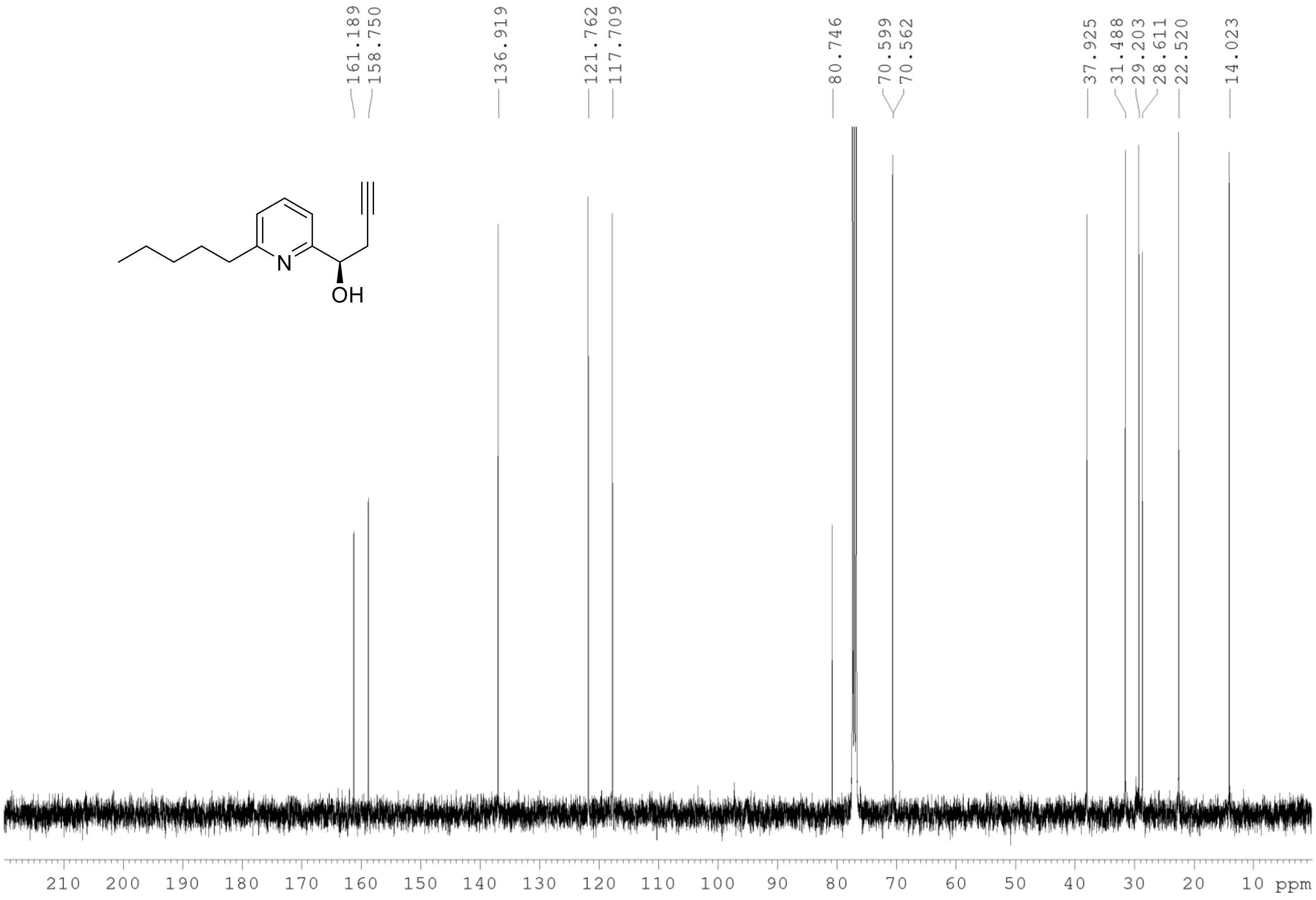

^{1}H NMR (400 MHz, $CDCl_3$) (*R*)-1-(6-pentylpyridin-2-yl)-4-(trimethylsilyl)but-3-yn-1-ol (6n)

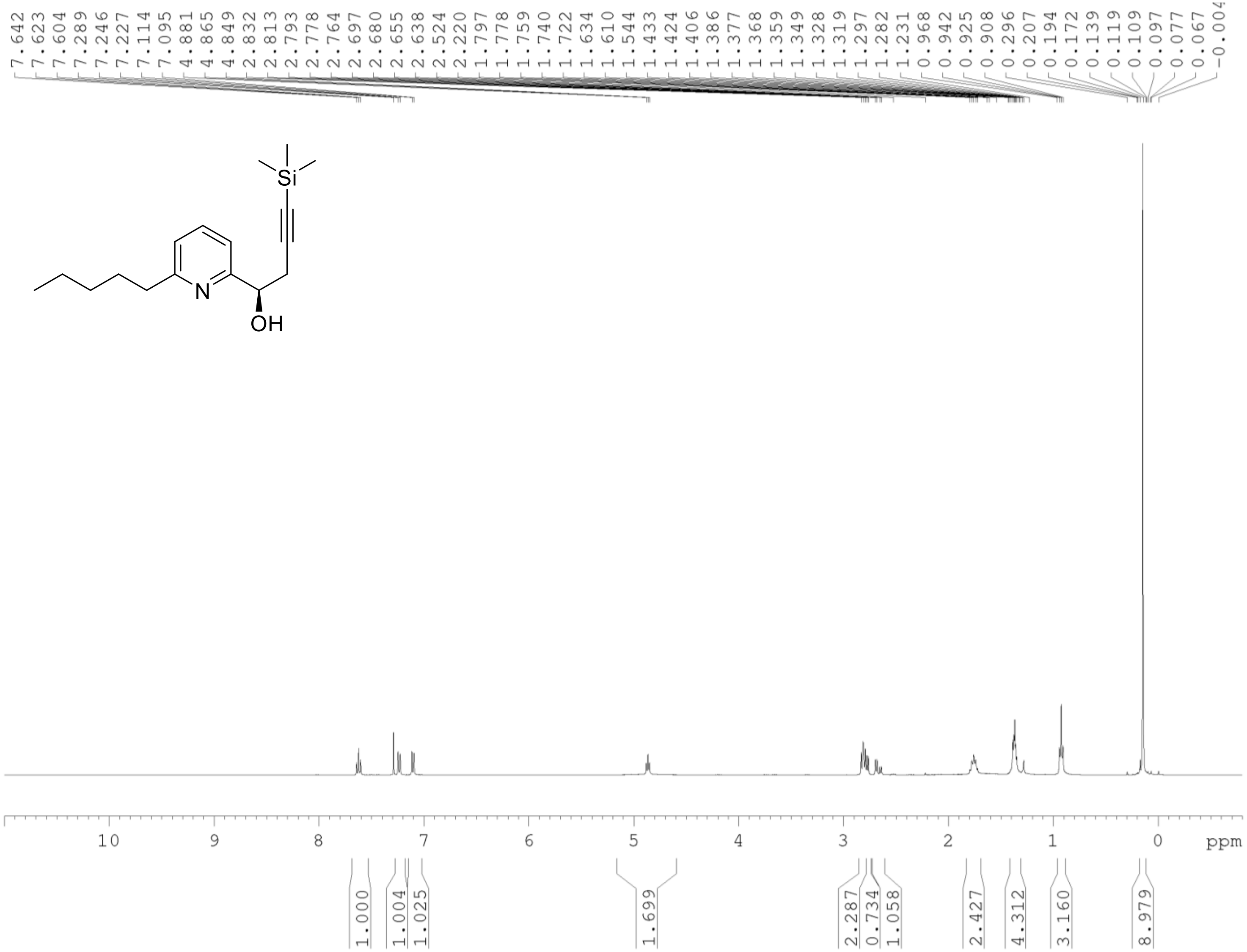

^{13}C NMR (101 MHz, CDCl$_3$) (*R*)-1-(6-pentylpyridin-2-yl)-4-(trimethylsilyl)but-3-yn-1-ol (6n)

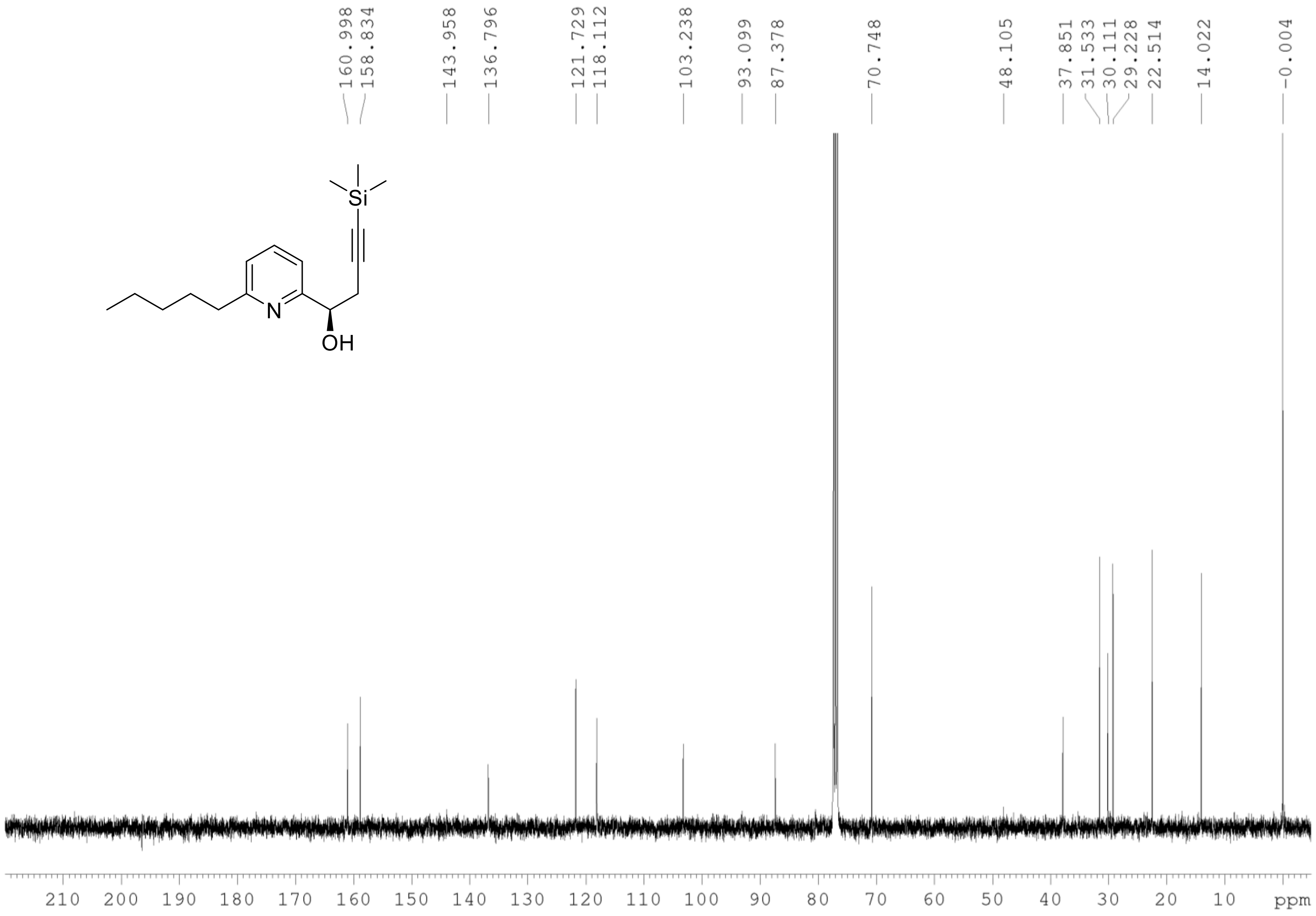

^{1}H NMR (400 MHz, $CDCl_3$) Methyl (*R*)-4-hydroxy-4-(6-pentylpyridin-2-yl)butanoate (6o)

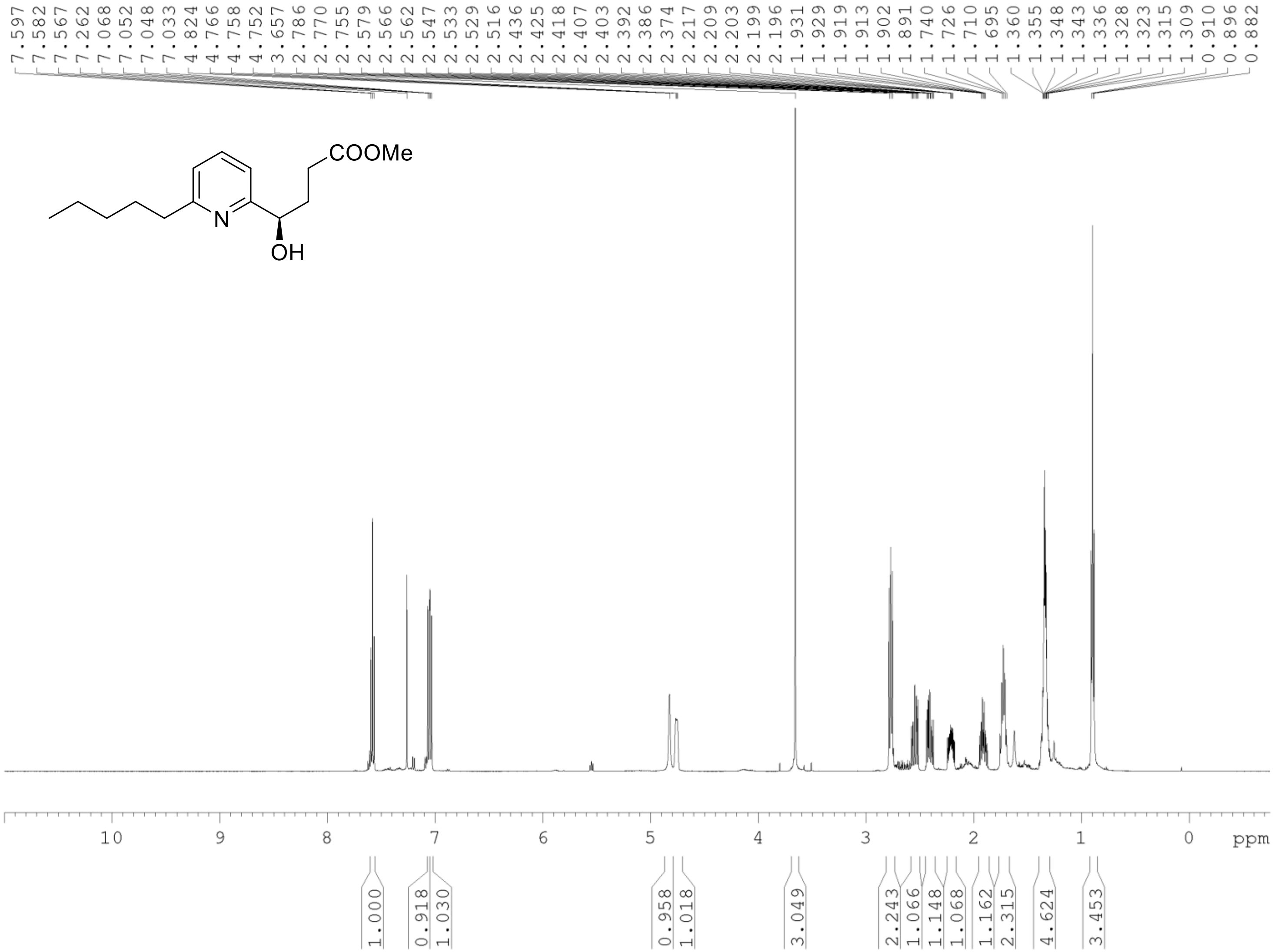

^{13}C NMR (101 MHz, CDCl$_3$) Methyl (*R*)-4-hydroxy-4-(6-pentylpyridin-2-yl)butanoate (6o)

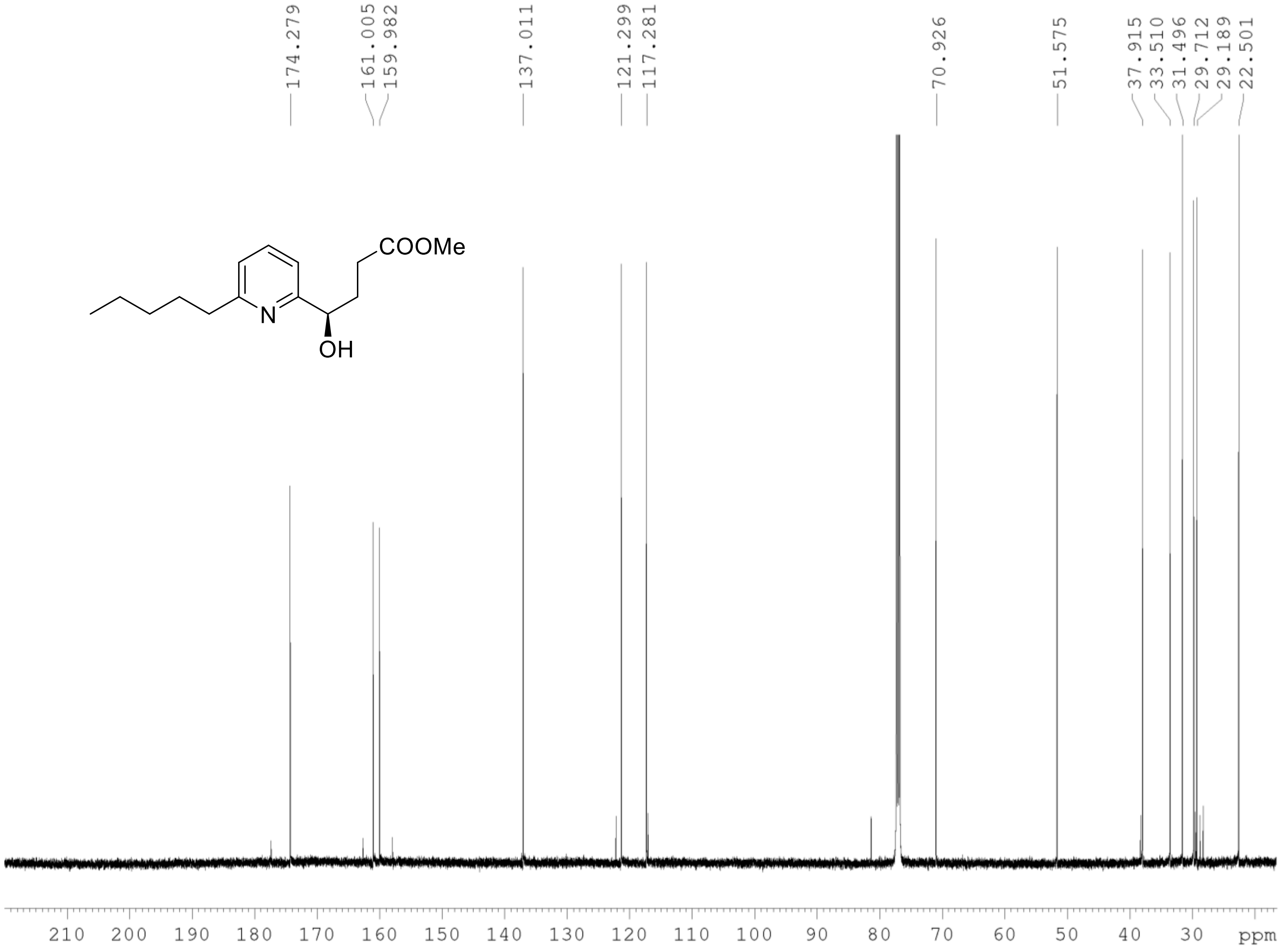

^{1}H NMR (400 MHz, CDCl3) (*R*)-3-((tert-butyldimethylsilyl)oxy)-1-(6-pentylpyridin-2-yl)propan-1-ol (6p)

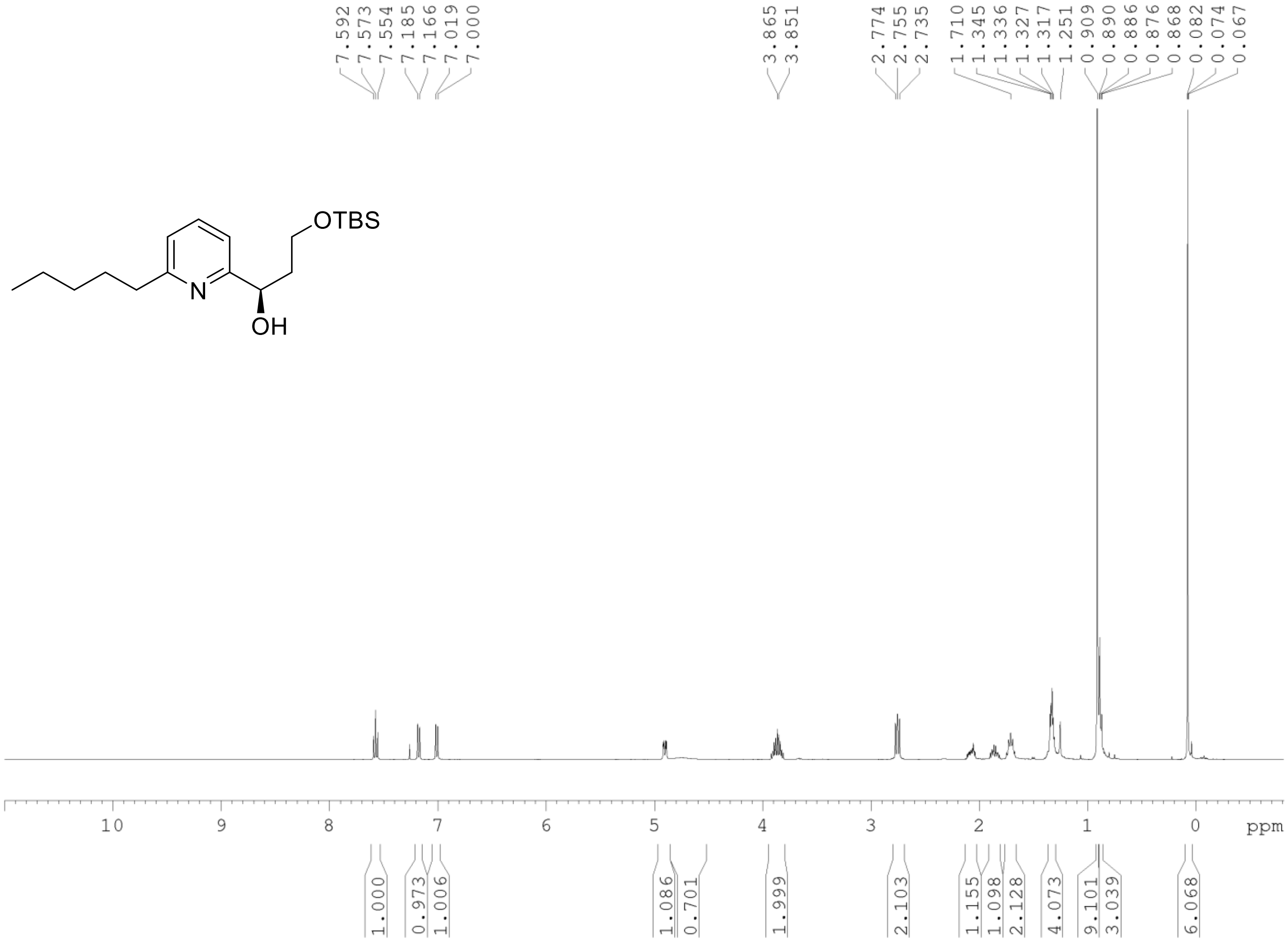

^{13}C NMR (101 MHz, CDCl3) (*R*)-3-((tert-butyldimethylsilyl)oxy)-1-(6-pentylpyridin-2-yl)propan-1-ol (6p)

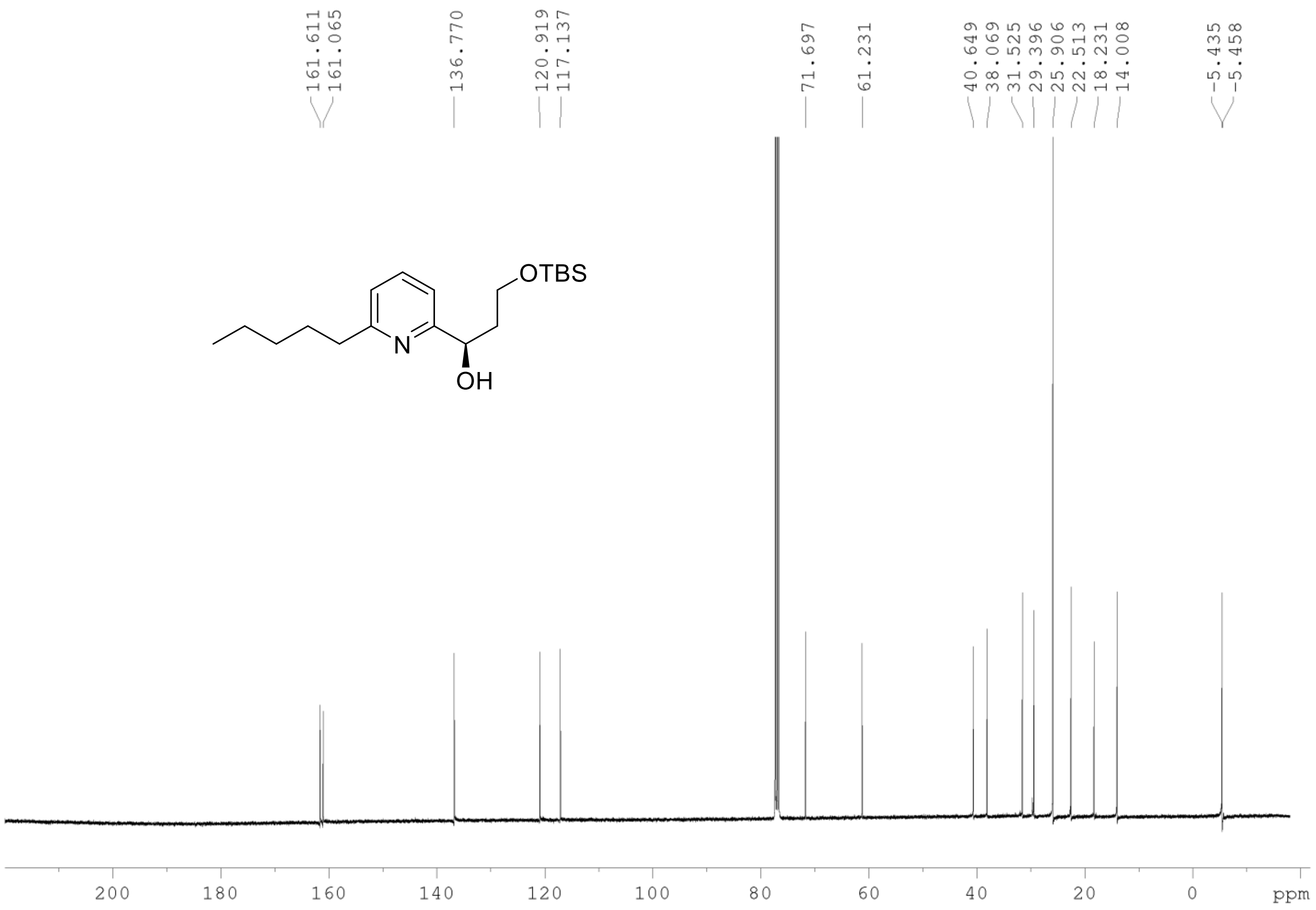

^{1}H NMR (400 MHz, $CDCl_3$) *tert*-Butyl (*R*)-(3-hydroxy-3-(6-pentylpyridin-2-yl)propyl)carbamate (6q)

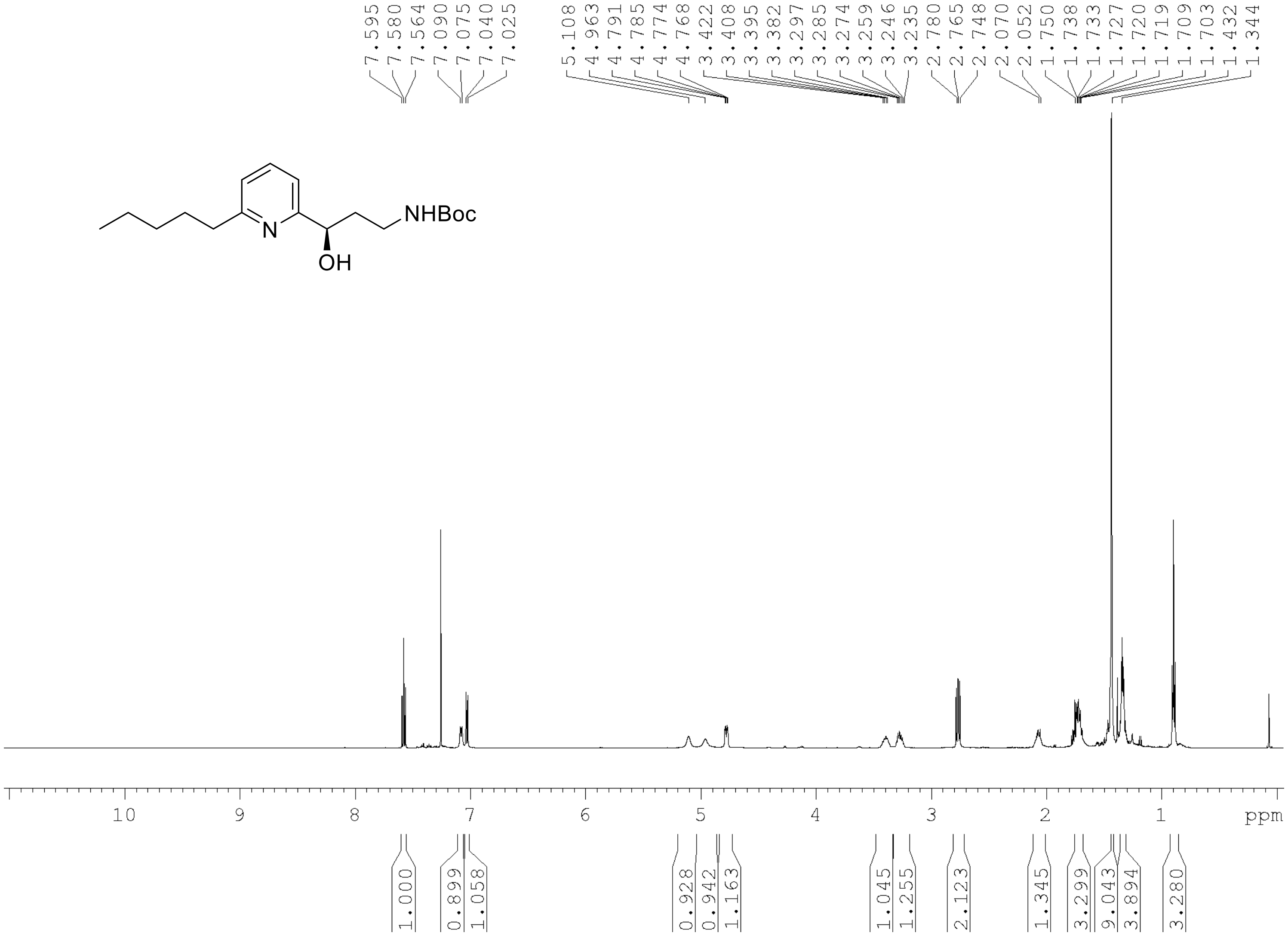

^{13}C NMR (126 MHz, $CDCl_3$) *tert*-butyl (*R*)-(3-hydroxy-3-(6-pentylpyridin-2-yl)propyl)carbamate (6q)

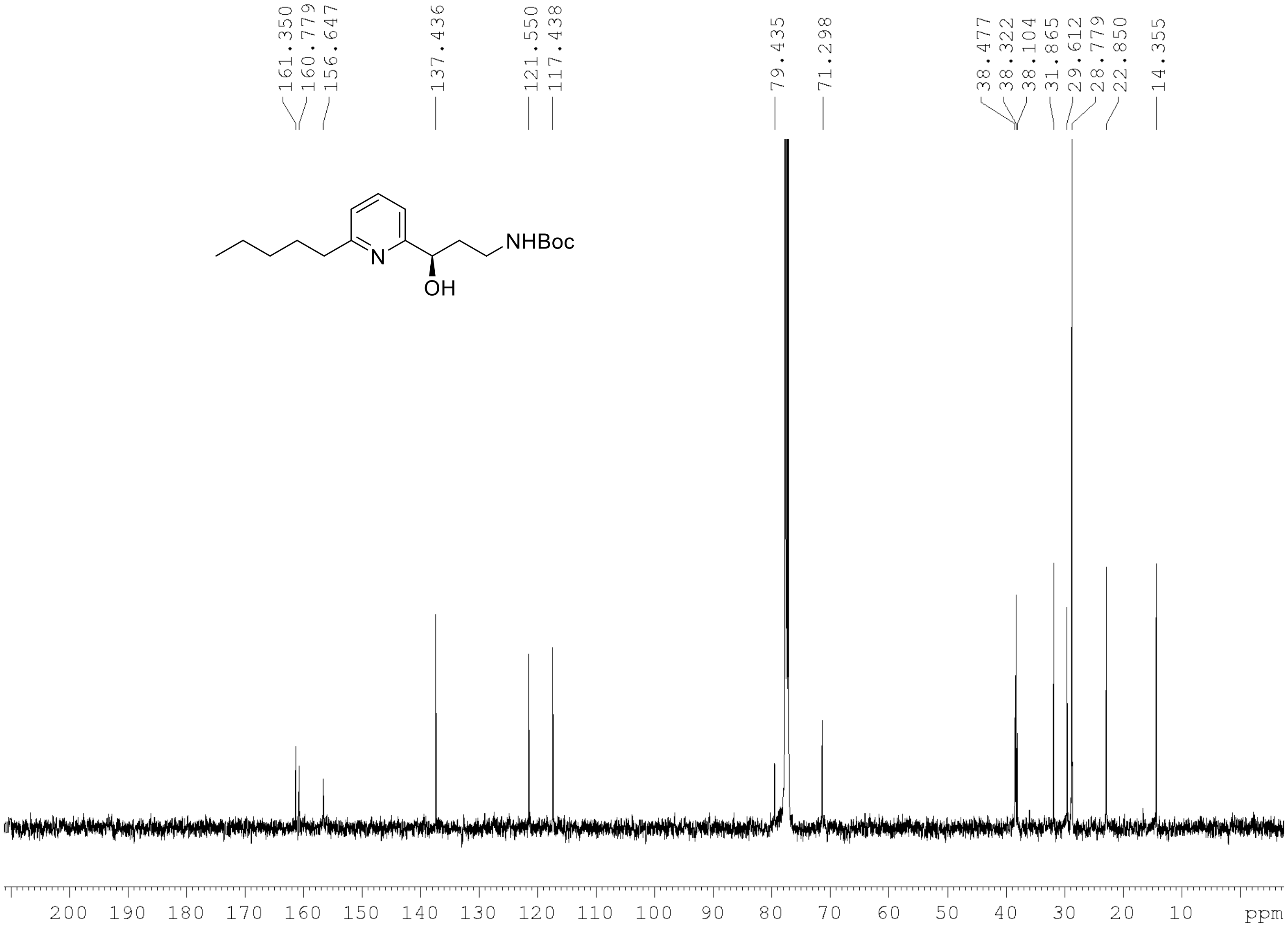

^{1}H NMR (500 MHz, $CDCl_3$) (*R*)-1-(pyridin-2-yl)ethyl-(*S*)-trifluoro-2-methoxy-2-phenylpropanoate

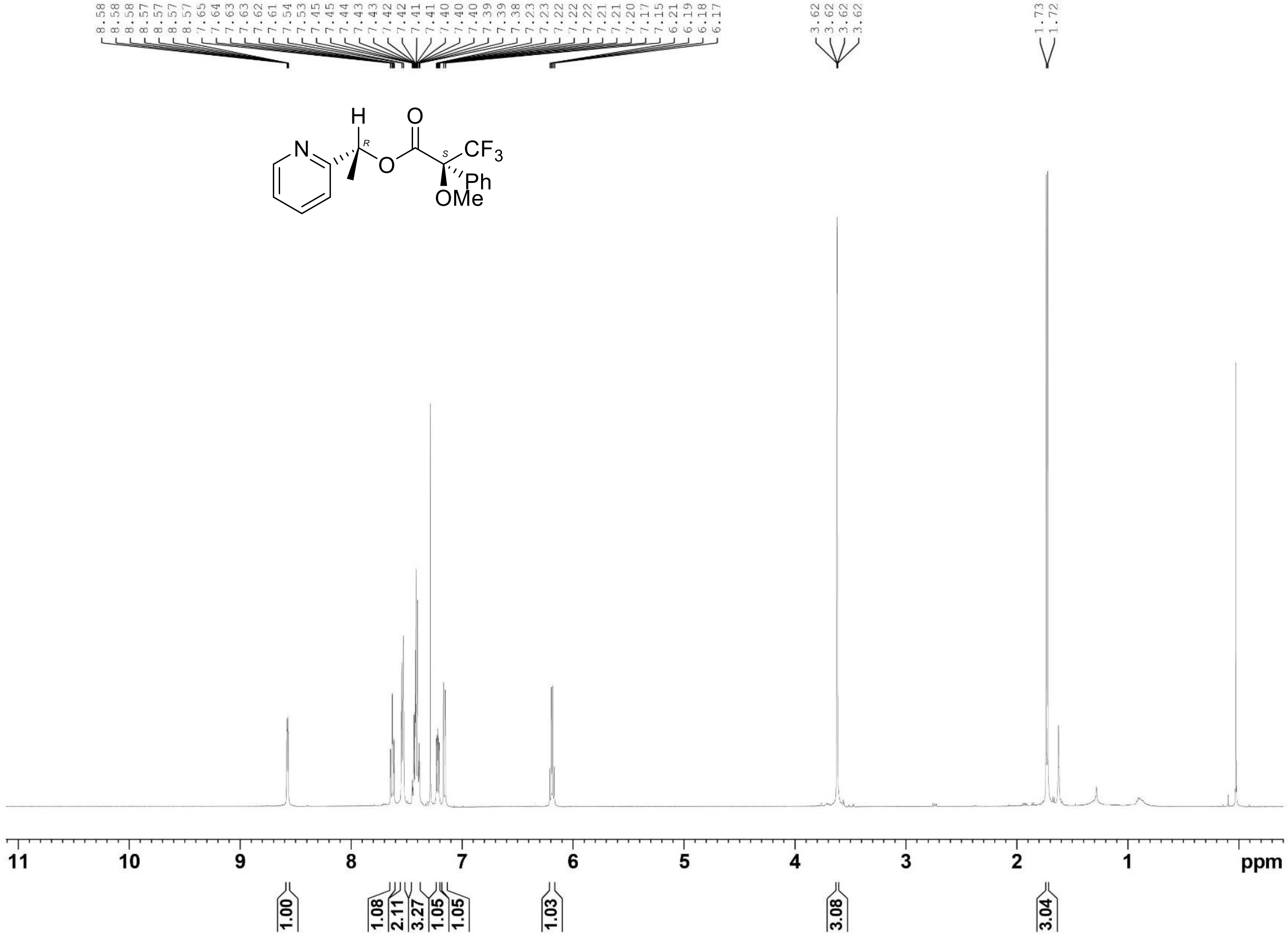

^{13}C NMR (126 MHz, $CDCl_3$) (*R*)-1-(pyridin-2-yl)ethyl-(*S*)-trifluoro-2-methoxy-2-phenylpropanoate

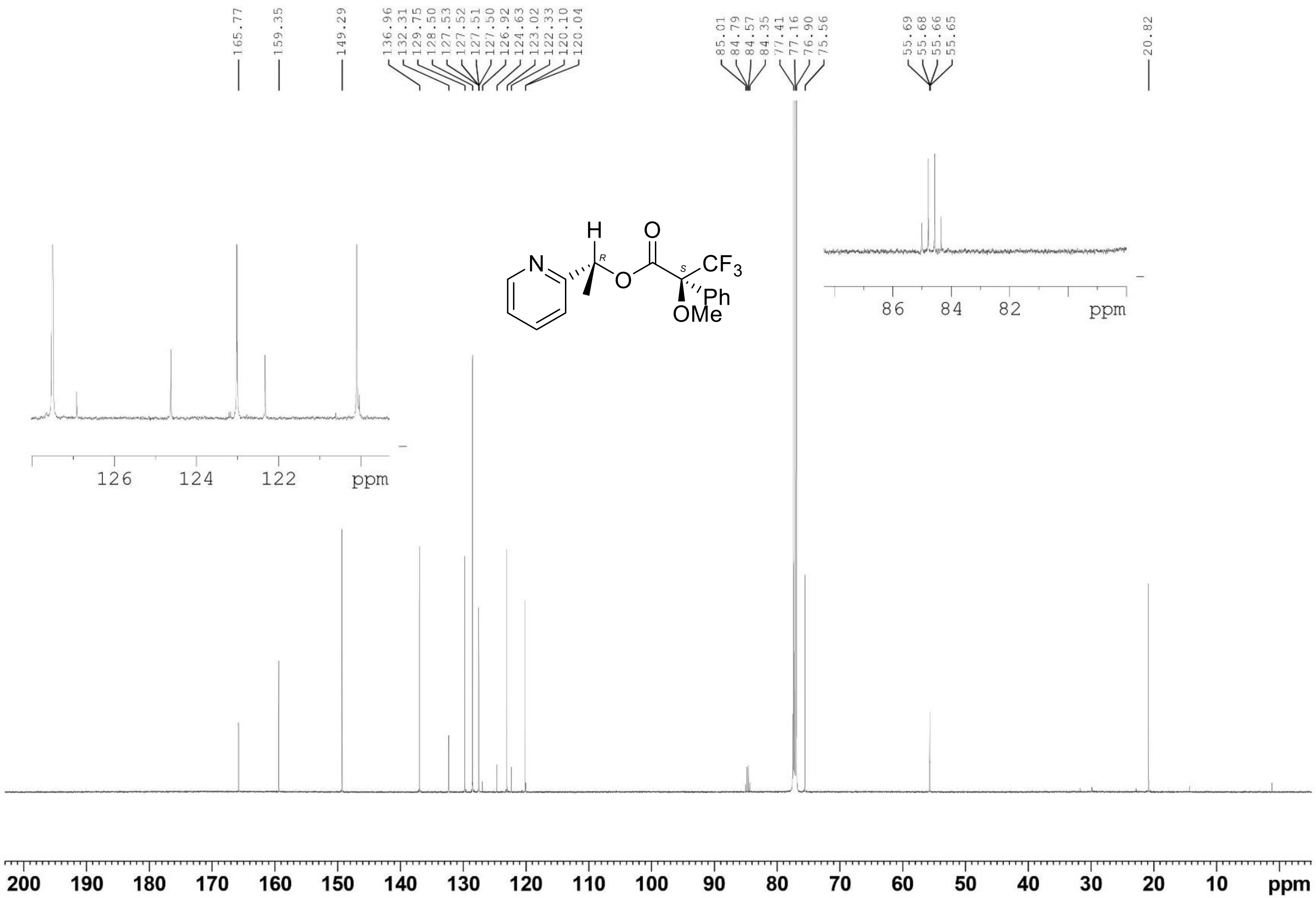

^{19}F NMR (471 MHz, $CDCl_3$) (*R*)-1-(pyridin-2-yl)ethyl-(*S*)-trifluoro-2-methoxy-2-phenylpropanoate

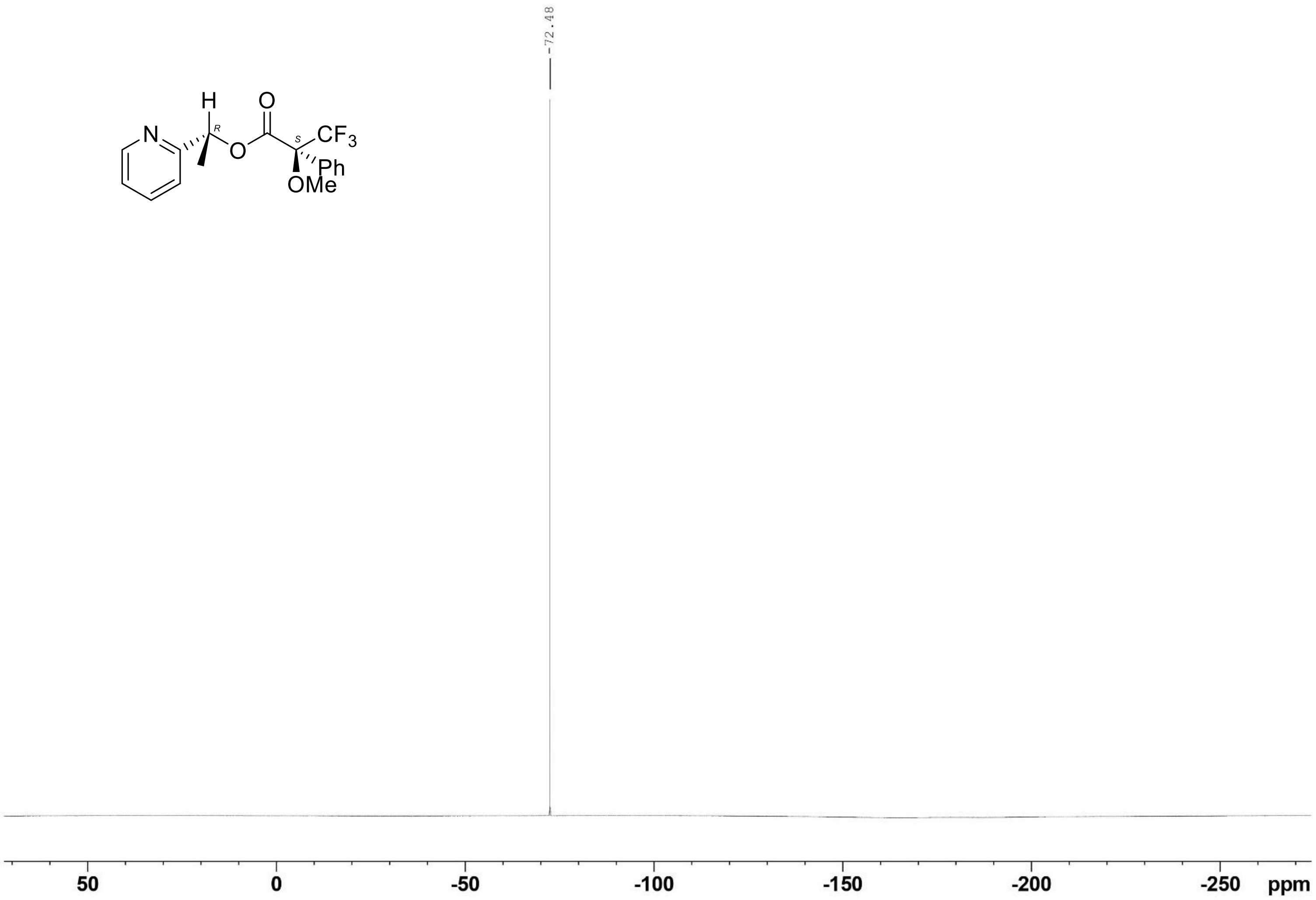

^1H NMR (500 MHz, $CDCl_3$) (*R*)-1-(pyridin-2-yl)ethyl-(*R*)-trifluoro-2-methoxy-2-phenylpropanoate

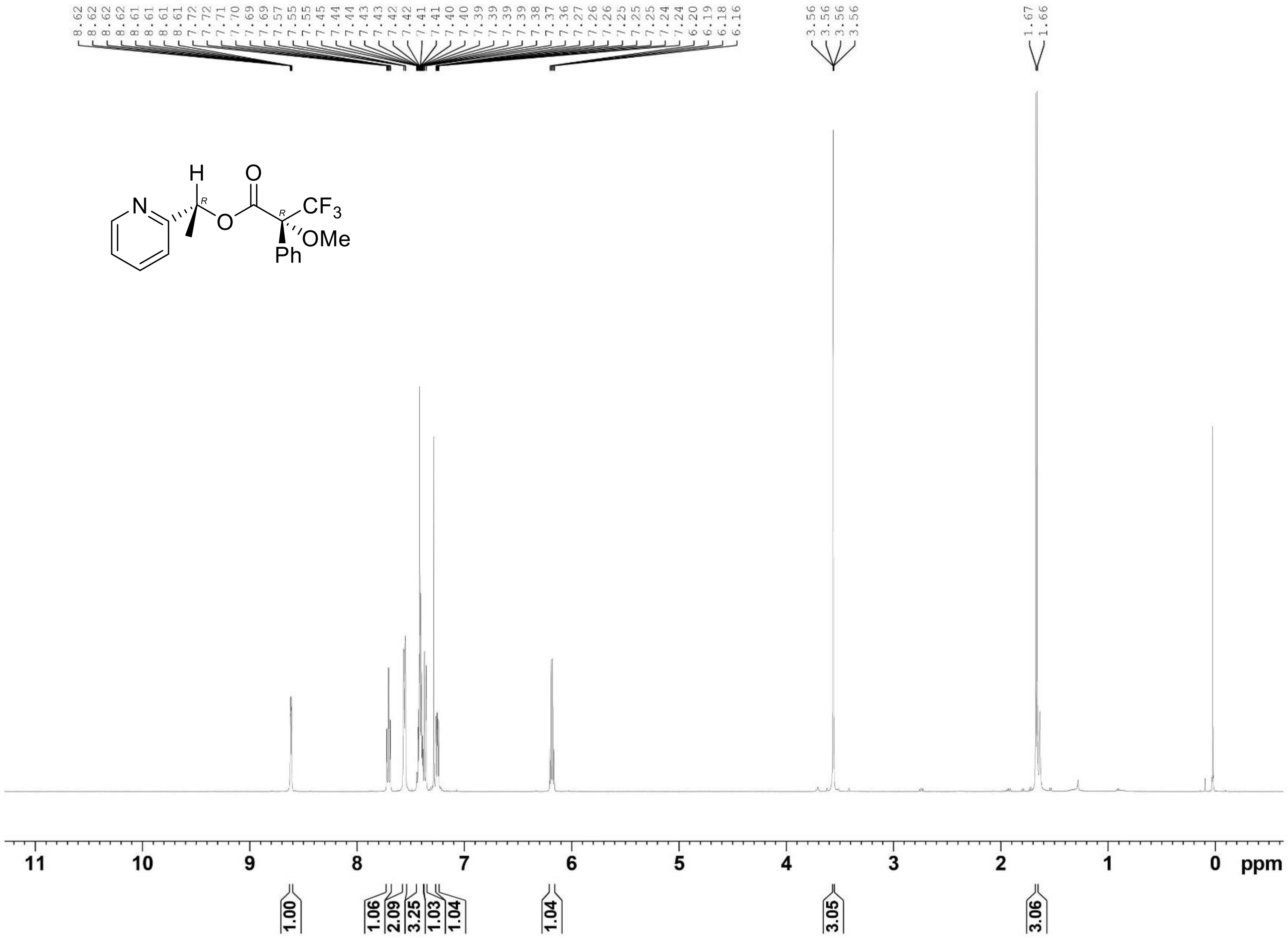

^{13}C NMR (126 MHz, $CDCl_3$) (*R*)-1-(pyridin-2-yl)ethyl-(*R*)-trifluoro-2-methoxy-2-phenylpropanoate

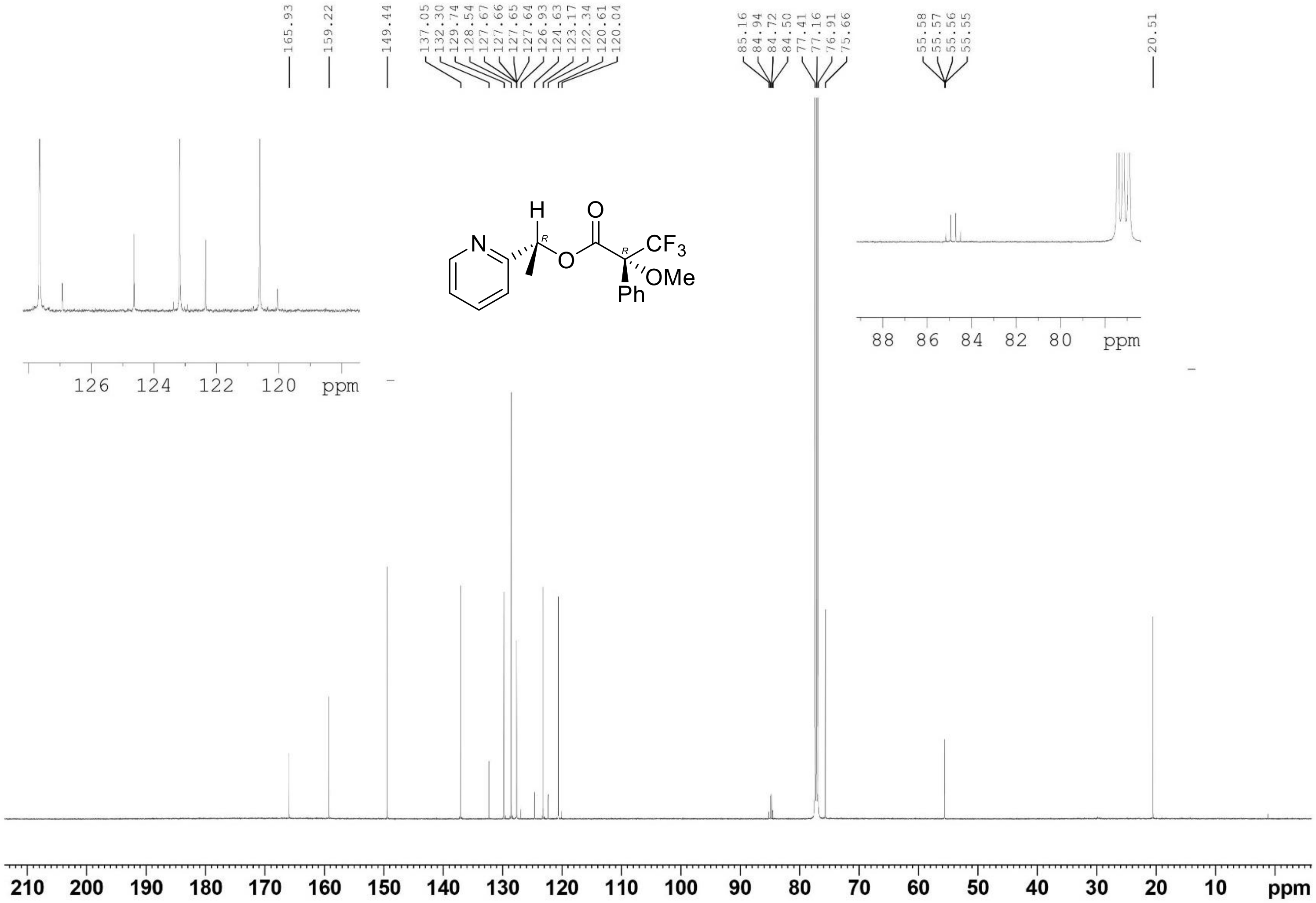

^{19}F NMR (471 MHz, CDCl$_3$) (*R*)-1-(pyridin-2-yl)ethyl-(*R*)-trifluoro-2-methoxy-2-phenylpropanoate

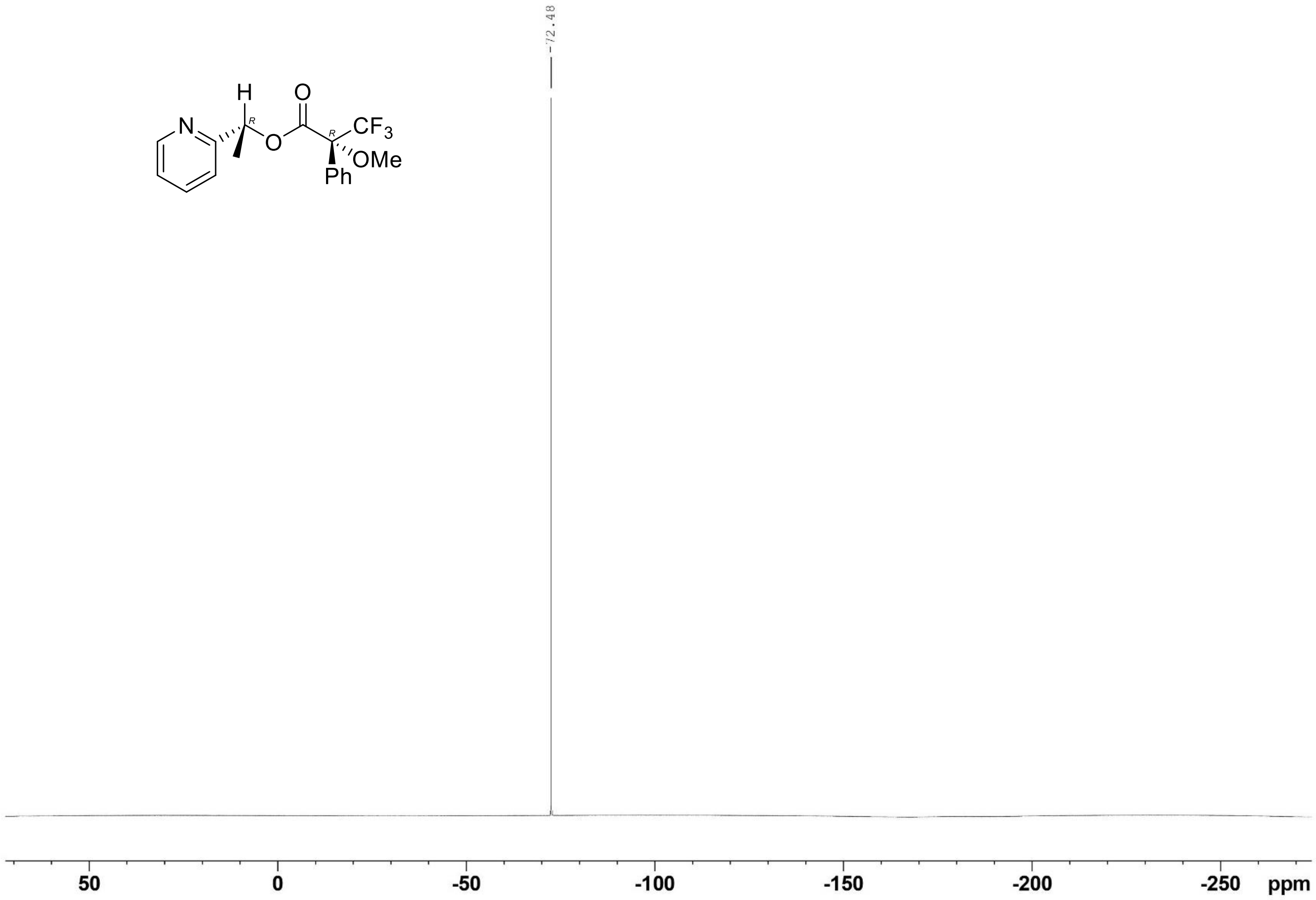

^{1}H NMR (500 MHz, $CDCl_3$) 1-(6-methylpyridin-2-yl)-3-phenylpropyl (2*S*)-trifluoro-2-methoxy-2-phenylpropanoate

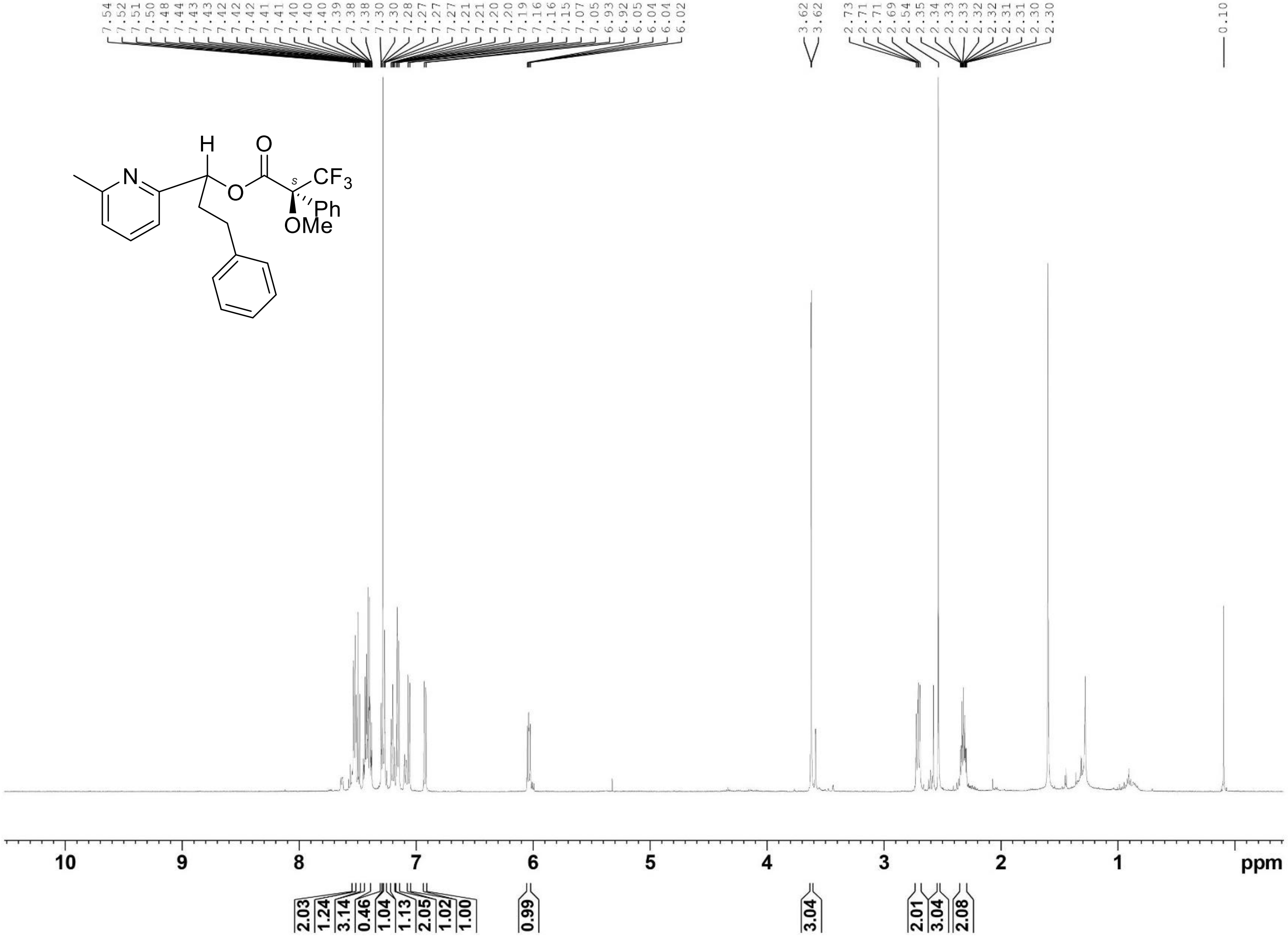

^{13}C NMR (126 MHz, CDCl$_3$) 1-(6-methylpyridin-2-yl)-3-phenylpropyl (2*S*)-trifluoro-2-methoxy-2-phenylpropanoate

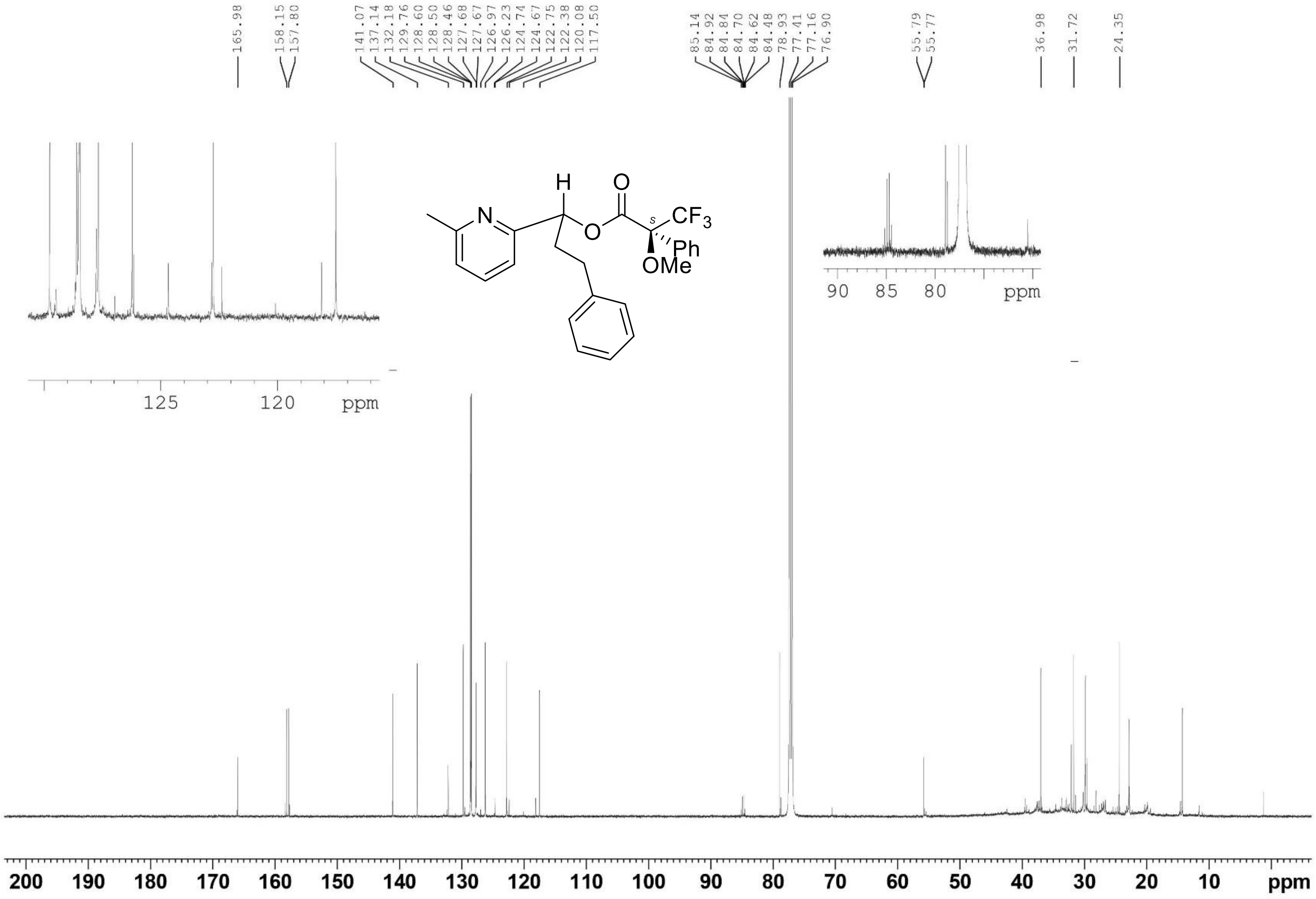

^{19}F NMR (471 MHz, $CDCl_3$) 1-(6-methylpyridin-2-yl)-3-phenylpropyl (2*S*)-trifluoro-2-methoxy-2-phenylpropanoate

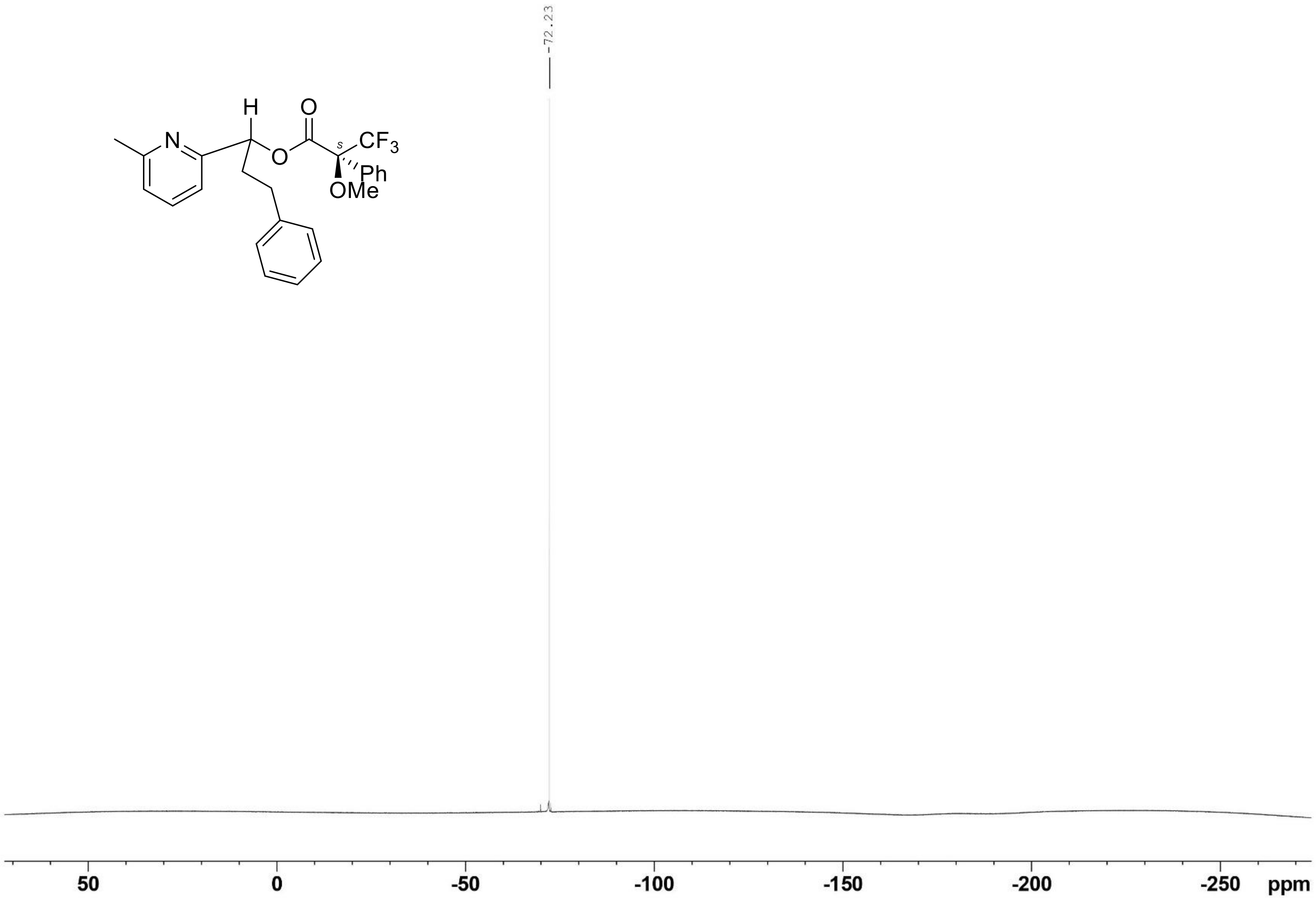

^{1}H NMR (400 MHz, $CDCl_3$) 1-(6-methylpyridin-2-yl)-3-phenylpropyl (2*R*)-trifluoro-2-methoxy-2-phenylpropanoate

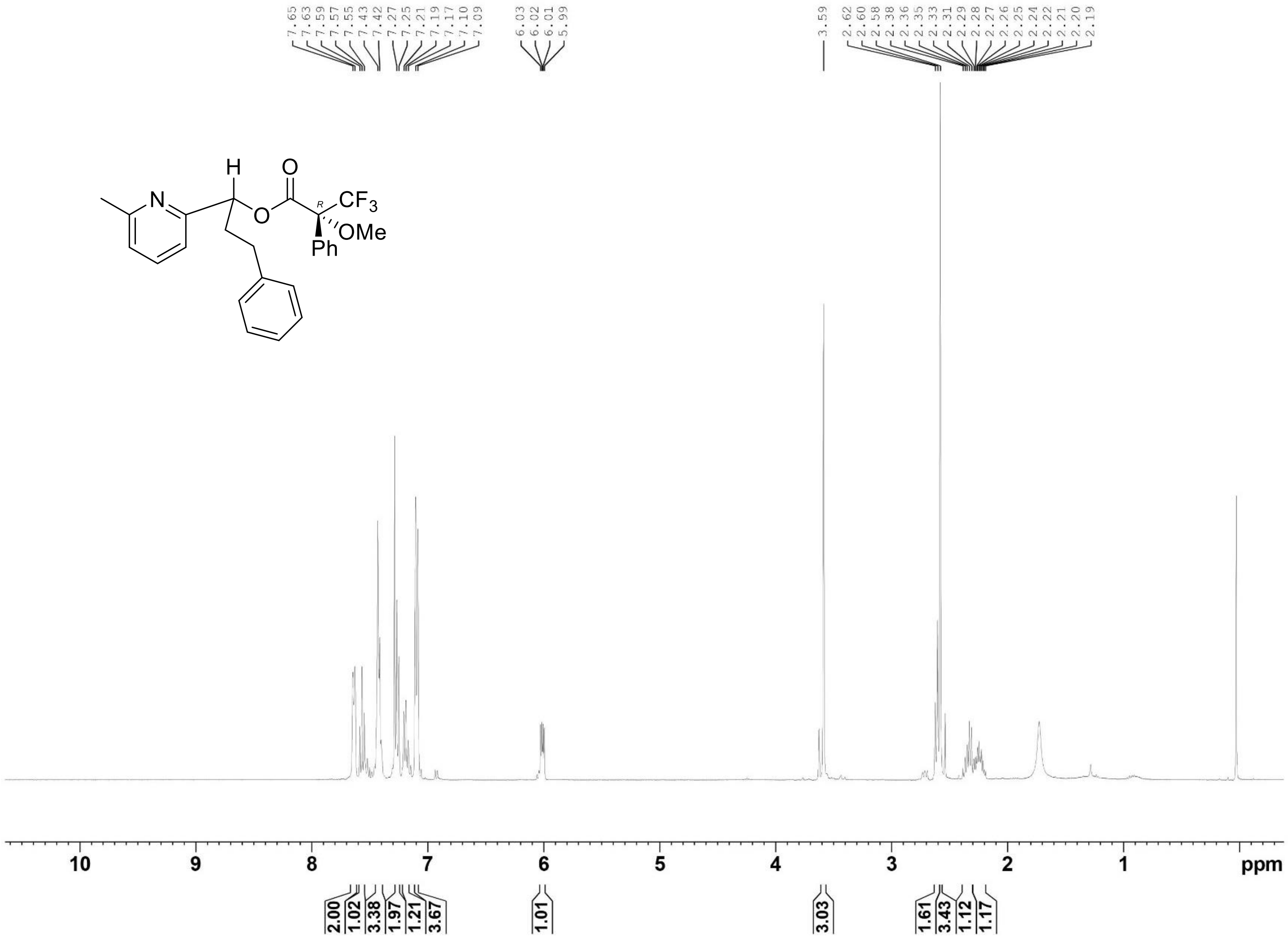

^{13}C NMR (101 MHz, $CDCl_3$) 1-(6-methylpyridin-2-yl)-3-phenylpropyl (2*R*)-trifluoro-2-methoxy-2-phenylpropanoate

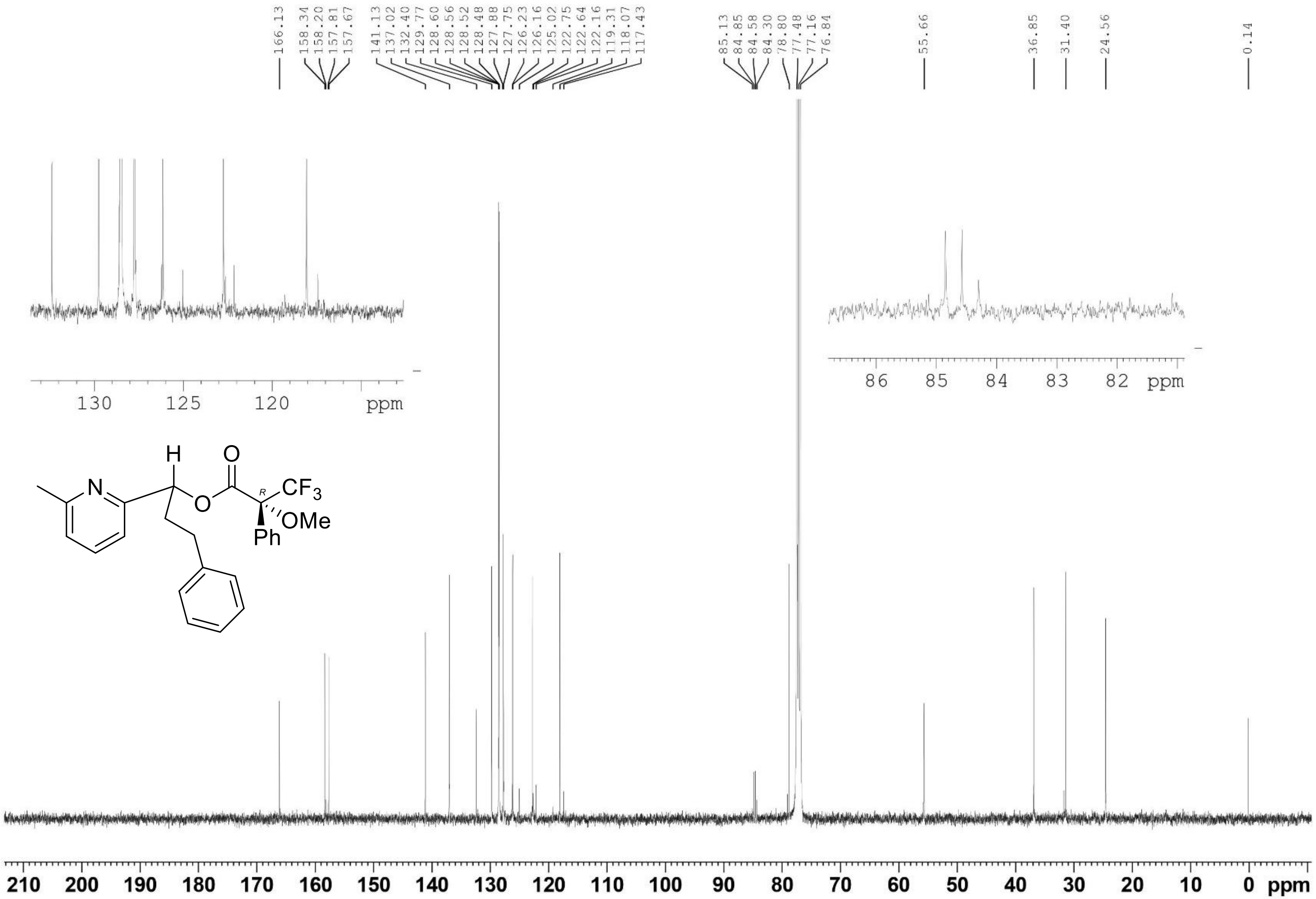

^{19}F NMR (471 MHz, CDCl$_3$) 1-(6-methylpyridin-2-yl)-3-phenylpropyl (2*R*)-trifluoro-2-methoxy-2-phenylpropanoate

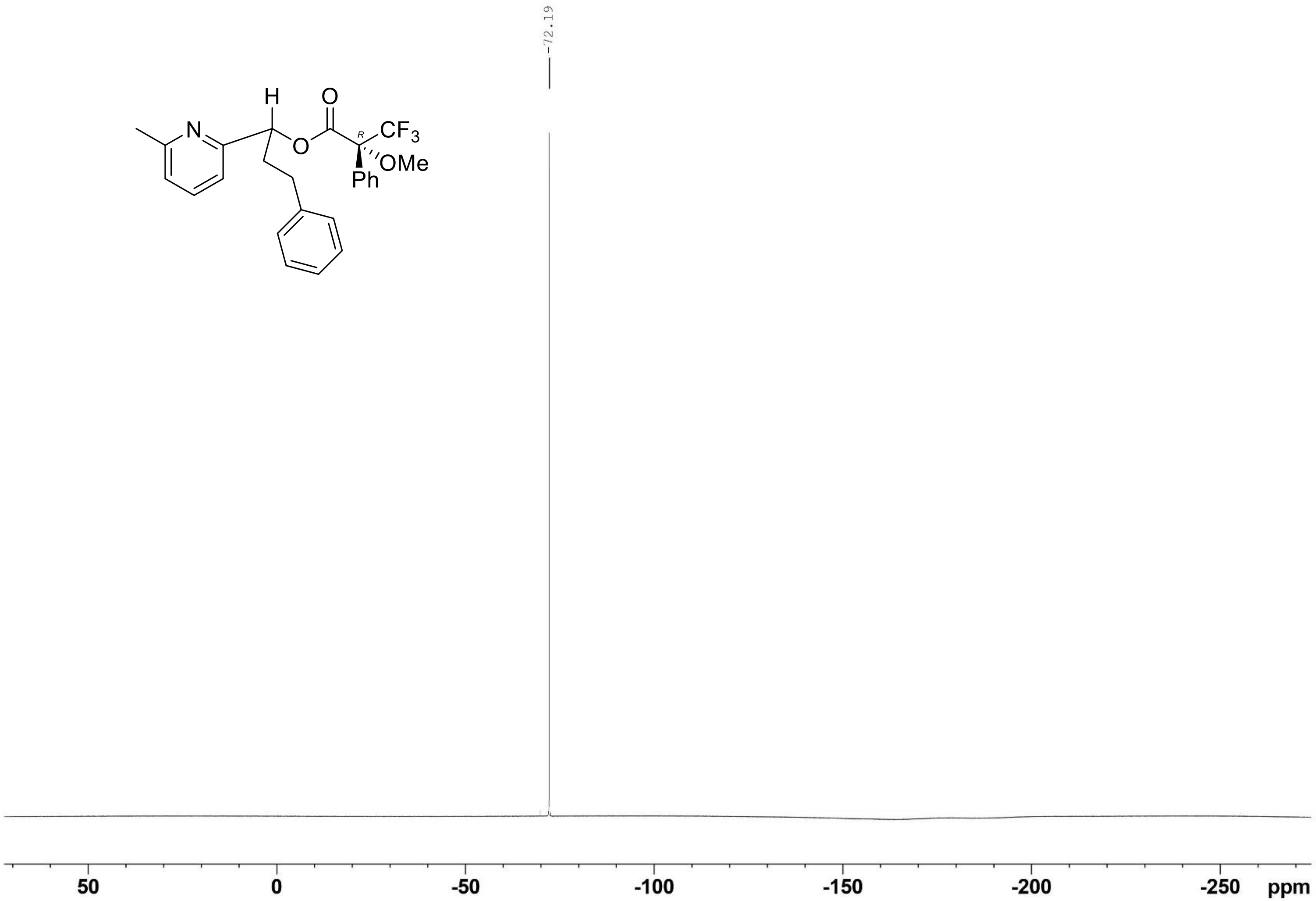

^{1}H NMR (500 MHz, $CDCl_3$) 3-phenyl-1-(5,6,7,8-tetrahydroquinolin-2-yl)propyl (2*S*)- trifluoro-2-methoxy-2-phenylpropanoate

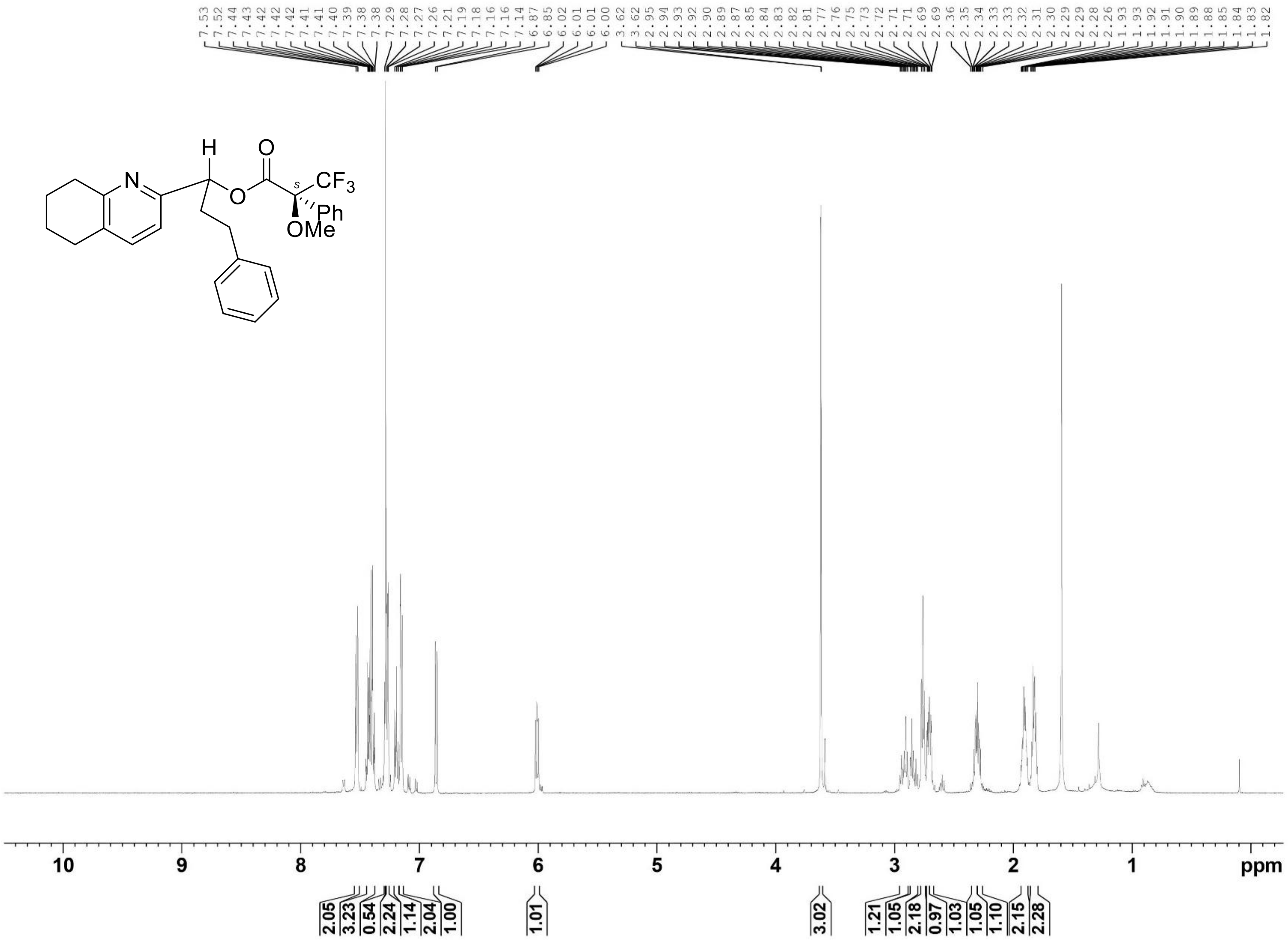

^{13}C NMR (126 MHz, $CDCl_3$) 3-phenyl-1-(5,6,7,8-tetrahydroquinolin-2-yl)propyl (2*S*)- trifluoro-2-methoxy-2-phenylpropanoate

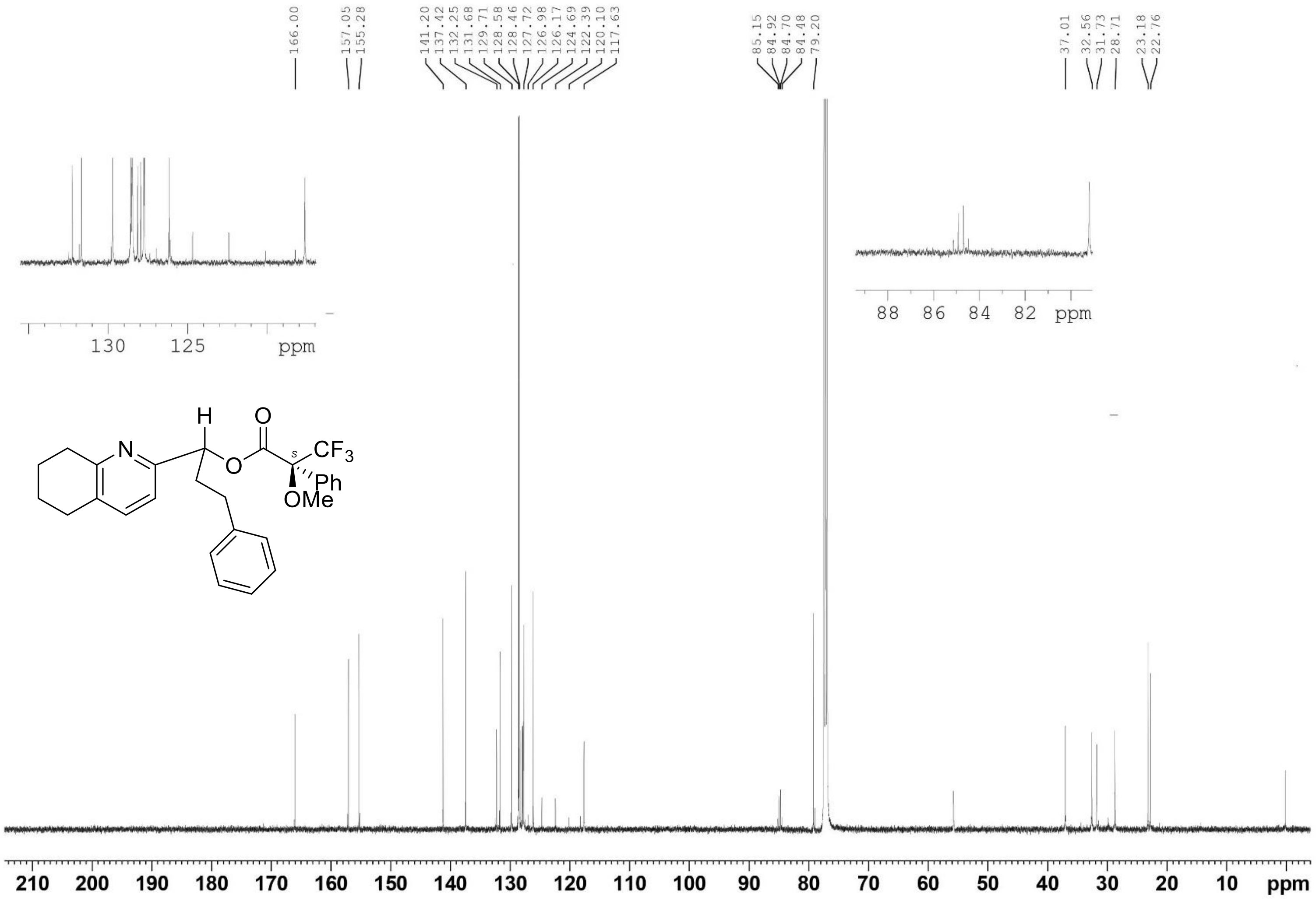

^{19}F NMR (471 MHz, $CDCl_3$) 3-phenyl-1-(5,6,7,8-tetrahydroquinolin-2-yl)propyl (2*S*)- trifluoro-2-methoxy-2-phenylpropanoate

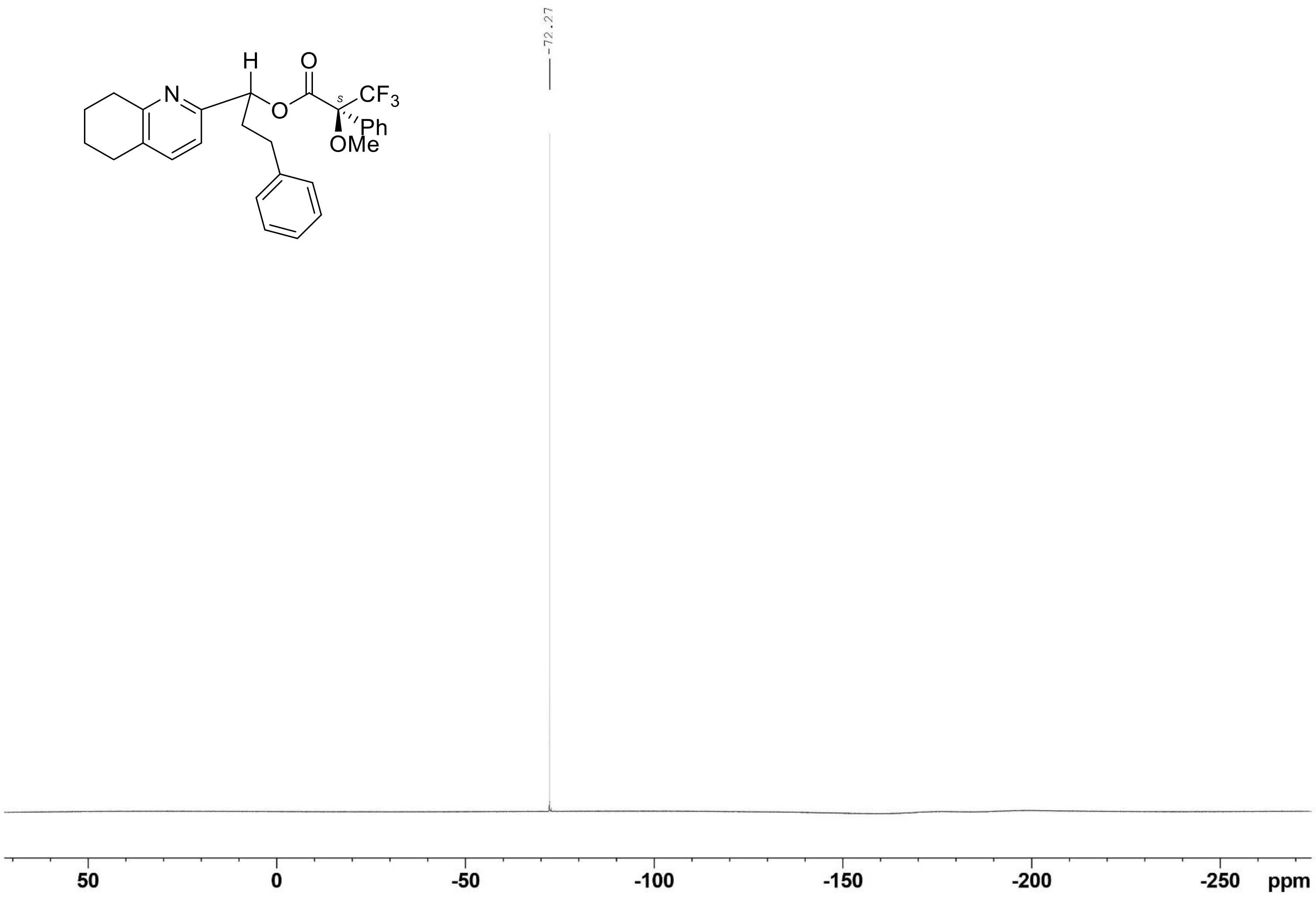

^{1}H NMR (500 MHz, $CDCl_3$) 3-phenyl-1-(5,6,7,8-tetrahydroquinolin-2-yl)propyl (2*R*)- trifluoro-2-methoxy-2-phenylpropanoate

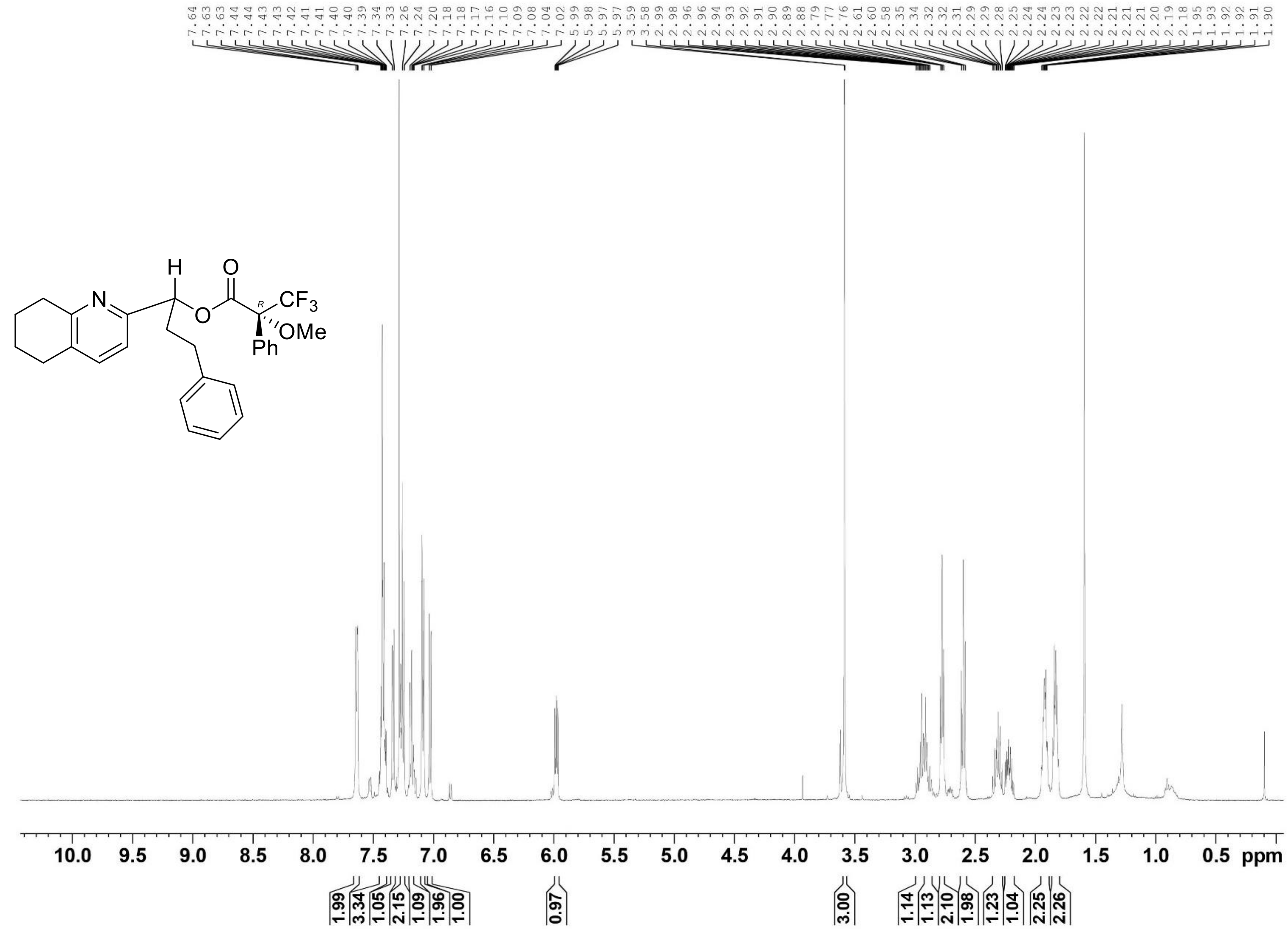

^{13}C NMR (126 MHz, CDCl₃) 3-phenyl-1-(5,6,7,8-tetrahydroquinolin-2-yl)propyl (2R)- trifluoro-2-methoxy-2-phenylpropanoate

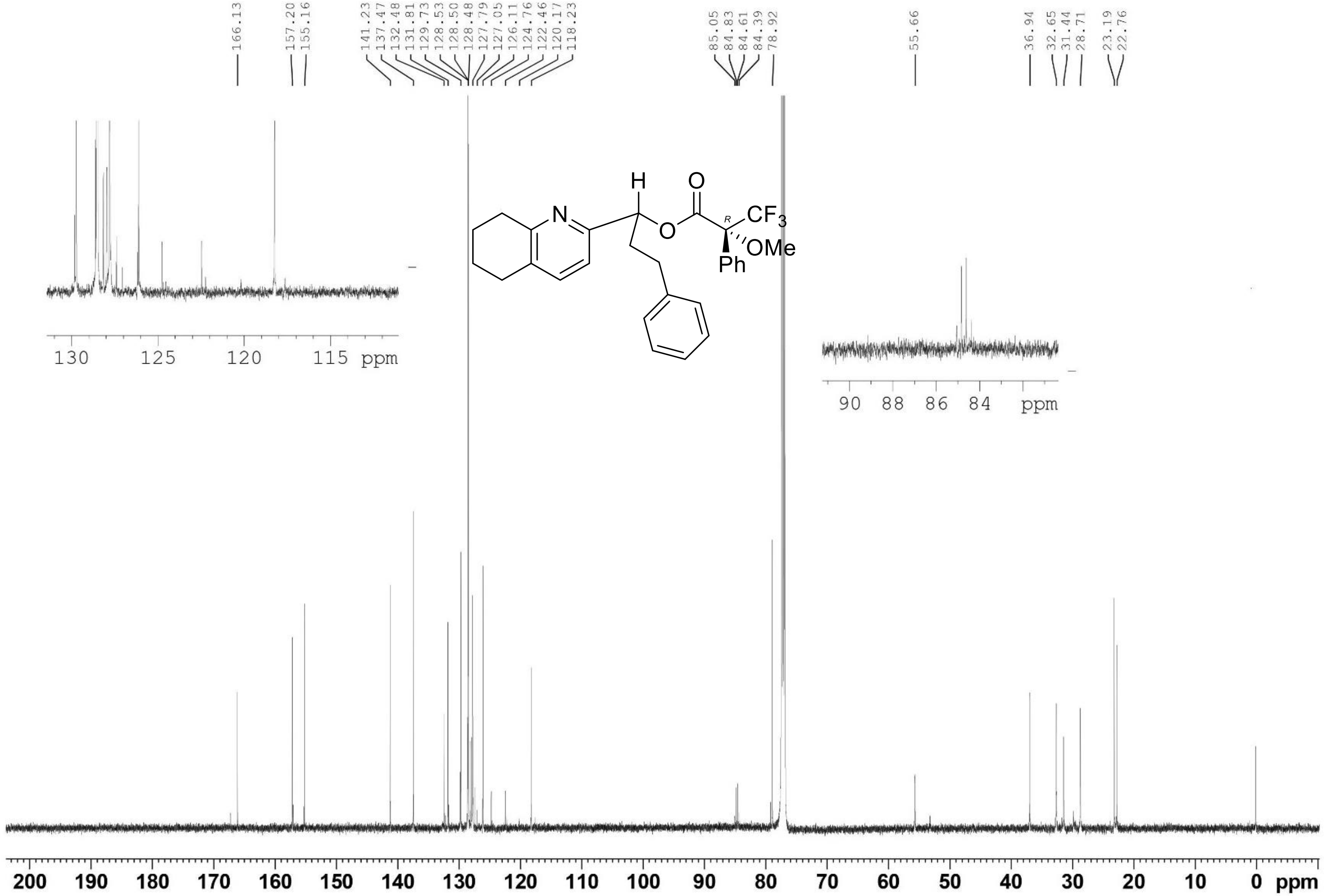

^{19}F NMR (471 MHz, $CDCl_3$) 3-phenyl-1-(5,6,7,8-tetrahydroquinolin-2-yl)propyl (2R)- trifluoro-2-methoxy-2-phenylpropanoate

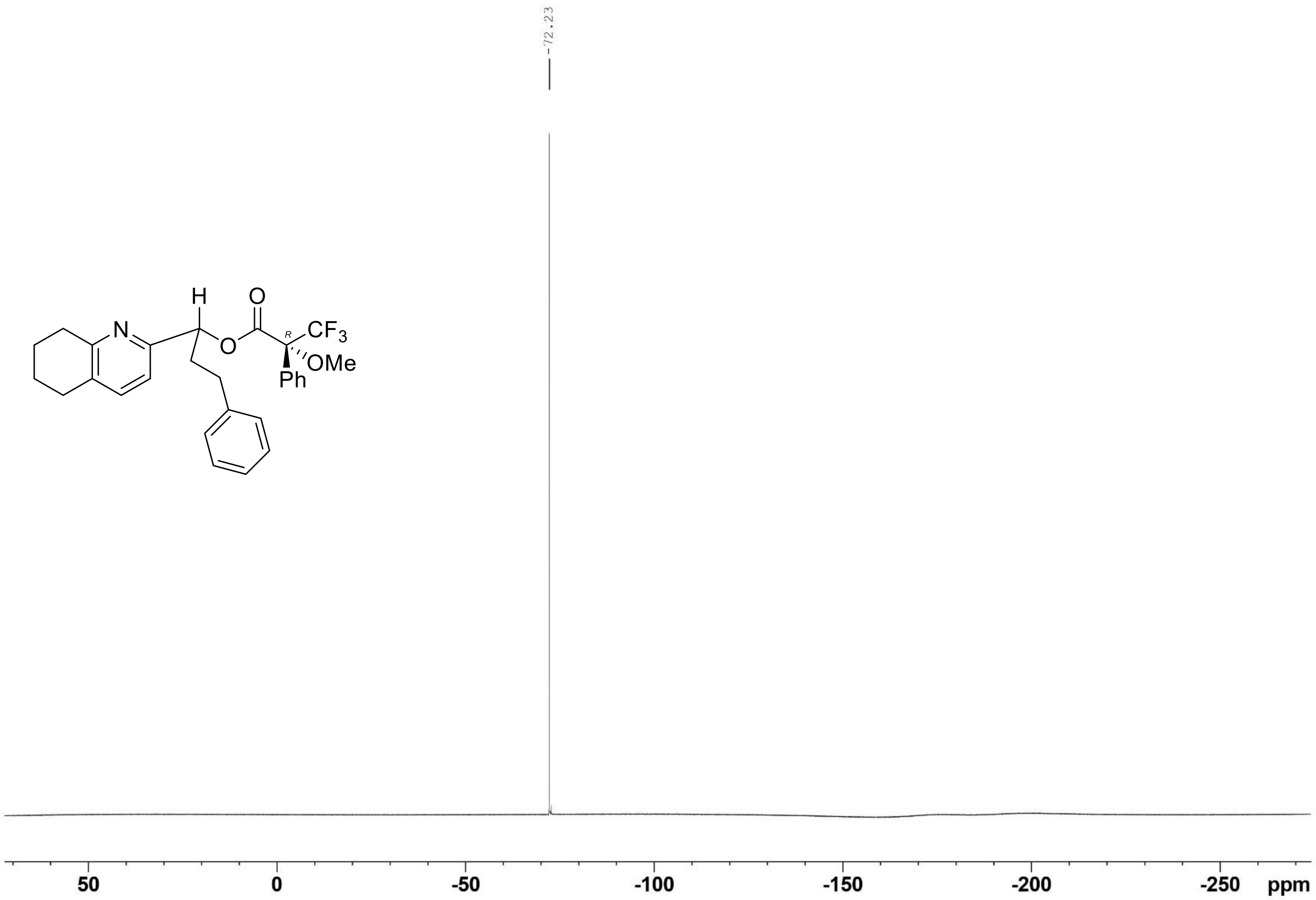

^{1}H NMR (400 MHz, $CDCl_3$) 2-(1-methoxy-3-phenylpropyl)-6-pentylpyridine (7a)

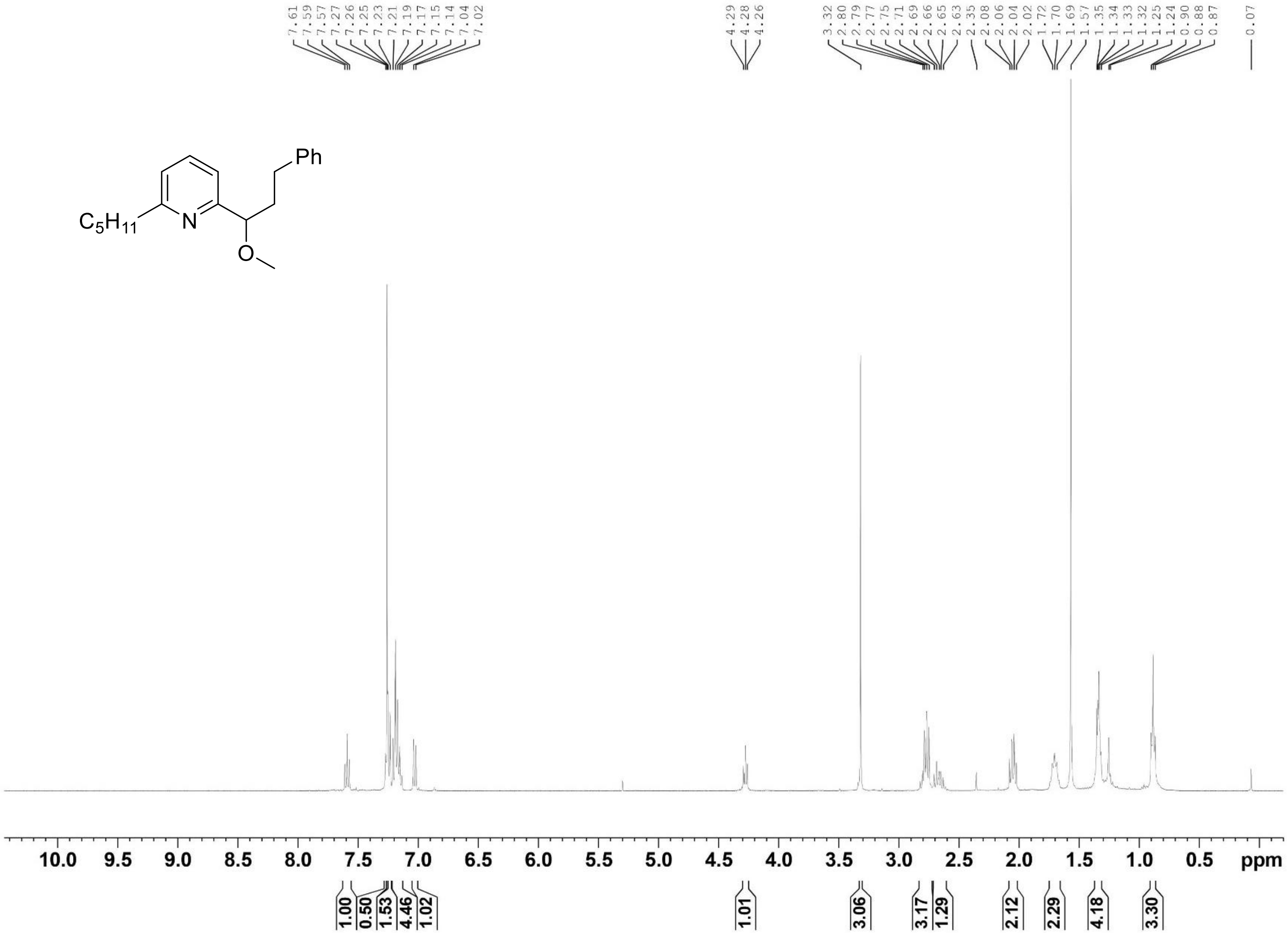

^{13}C NMR (101 MHz, $CDCl_3$) 2-(1-methoxy-3-phenylpropyl)-6-pentylpyridine (7a)

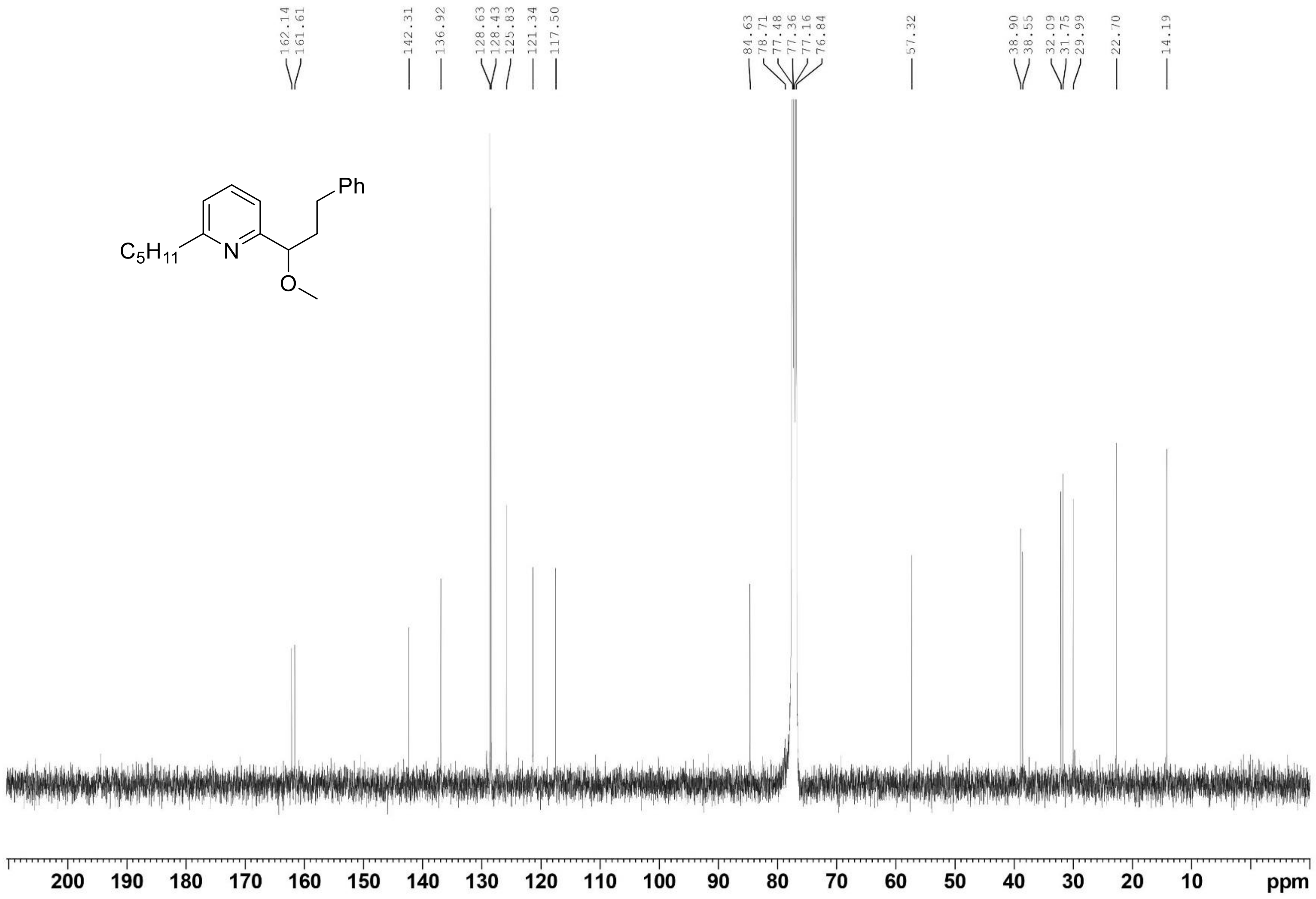

SFC Traces

(*R*)-1-(6-pentylpyridin-2-yl)-3-phenylpropan-1-ol (2a)

SFC Analysis: CHIRAL ART SC (CO_2/MeOH = 97/03, 2.5 mL min^{-1}, 40 °C, 262 nm); **<u>85% ee</u>**

t_R = 8.0 (minor), 8.5 (major) minutes

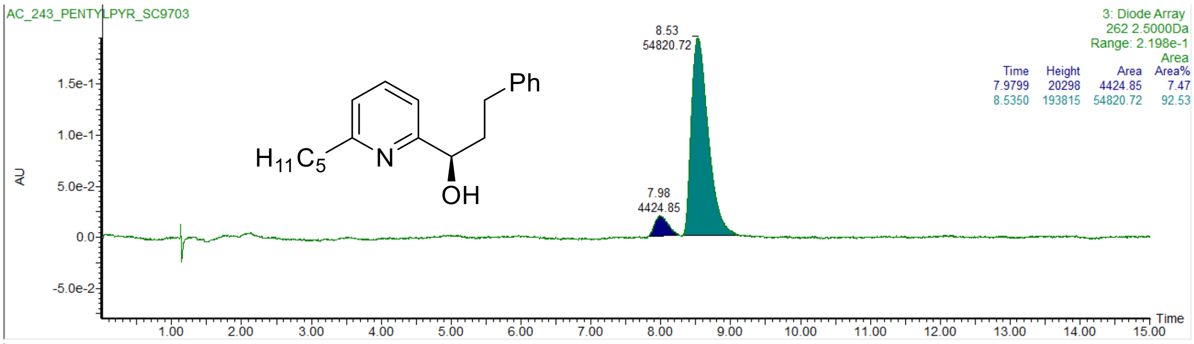

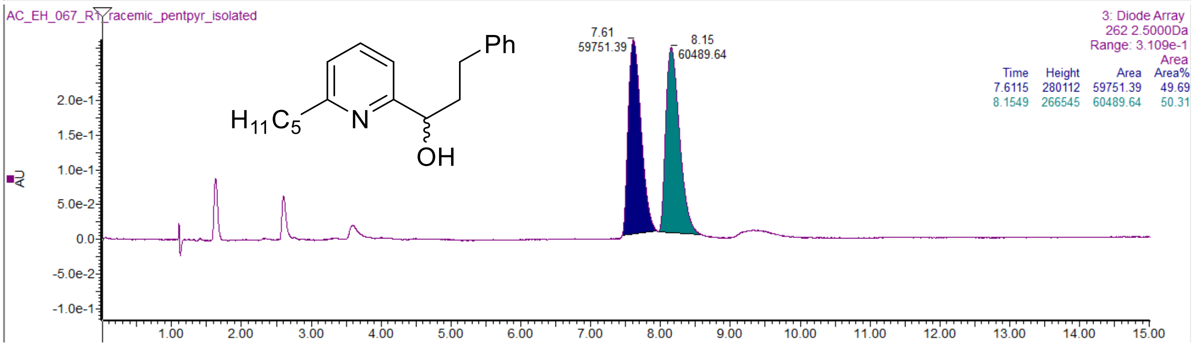

(*R*)-1-(6-methylpyridin-2-yl)-3-phenylpropan-1-ol (2b)

SFC Analysis: Chiralpak IG (CO_2/MeOH = 85/15, 2.5 mL min^{-1}, 40 °C, 263 nm); **<u>82% ee</u>**

t_R = 4.5 (minor), 4.8 (major) minutes

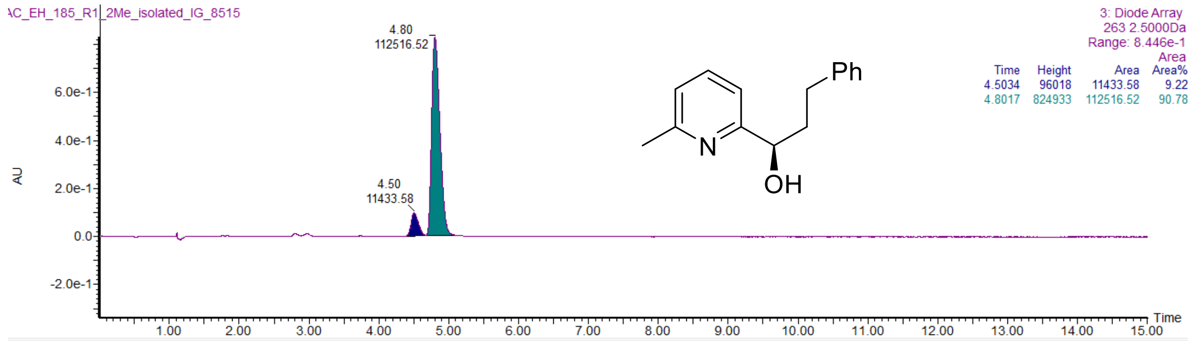

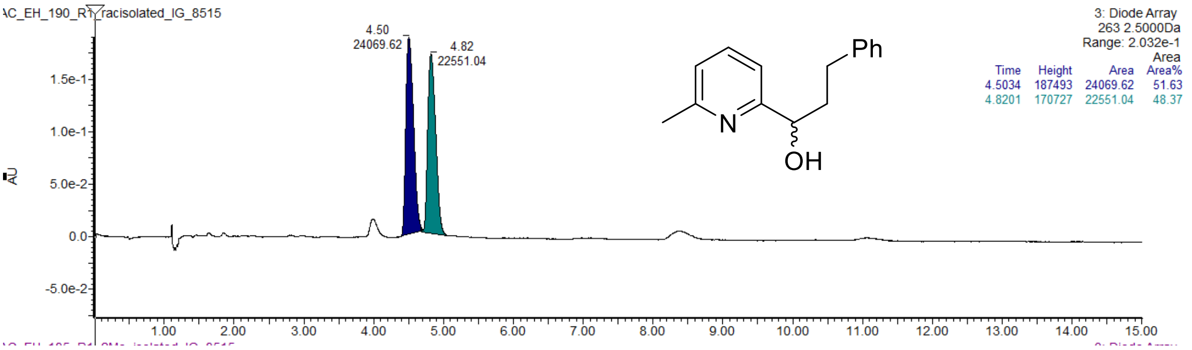

(*R*)-1-(6-isopropylpyridin-2-yl)-3-phenylpropan-1-ol (2c)

SFC Analysis: Chiralpak IE (CO_2/MeOH = 95/05, 2.5 mL min^{-1}, 40 °C, 262 nm); **<u>80% ee</u>**

t_R = 6.3 (minor), 6.8 (major) minutes

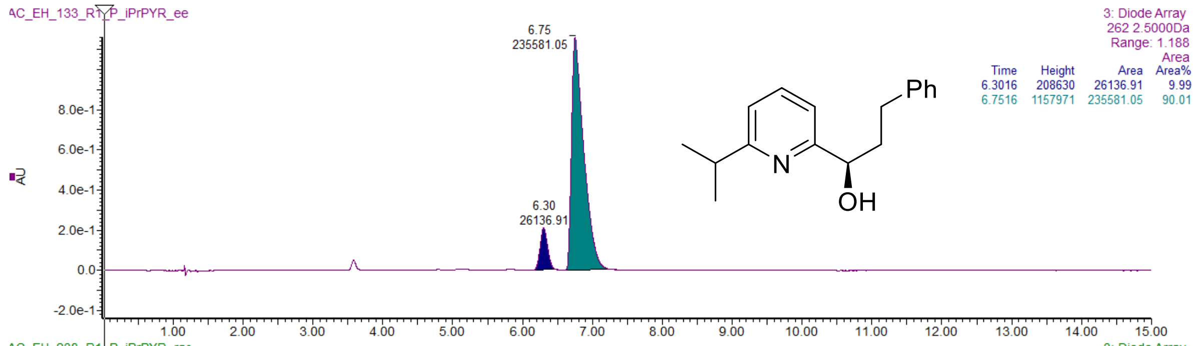

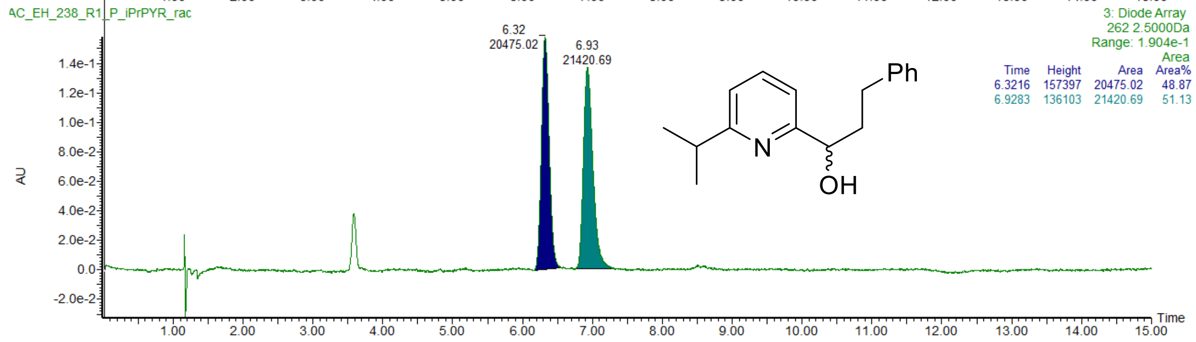

(*R*)-1-(6-cyclohexylpyridin-2-yl)-3-phenylpropan-1-ol (2d)

SFC Analysis: Chiralpak IE (CO_2/MeOH = 93/07, 2.5 mL min^{-1}, 40 °C, 262 nm); **<u>80% ee</u>**

t_R = 9.3 (minor), 10.7 (major) minutes

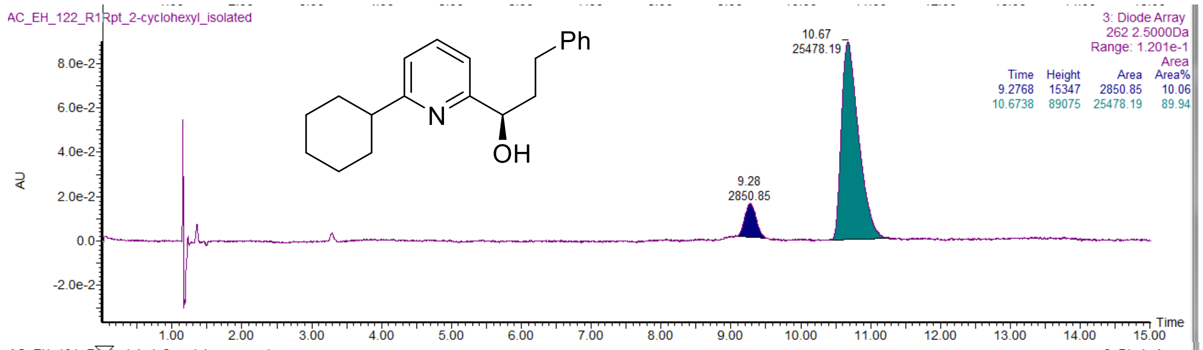

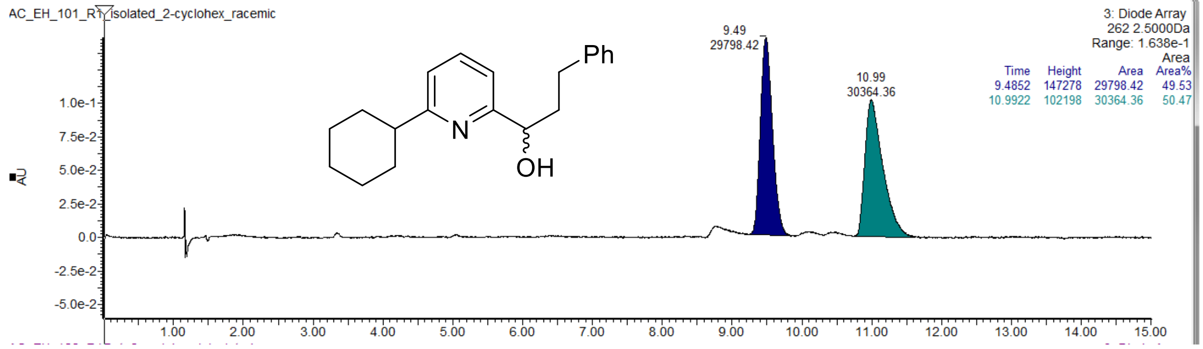

(*R*)-1-(6-(cyclohexylmethyl)pyridin-2-yl)-3-phenylpropan-1-ol (2e)

SFC Analysis: Chiralpak IE (CO_2/MeOH = 93/07, 2.5 mL min^{-1}, 40 °C, 263 nm); **<u>81% ee</u>**

t_R = 9.8 (minor), 10.2 (major) minutes

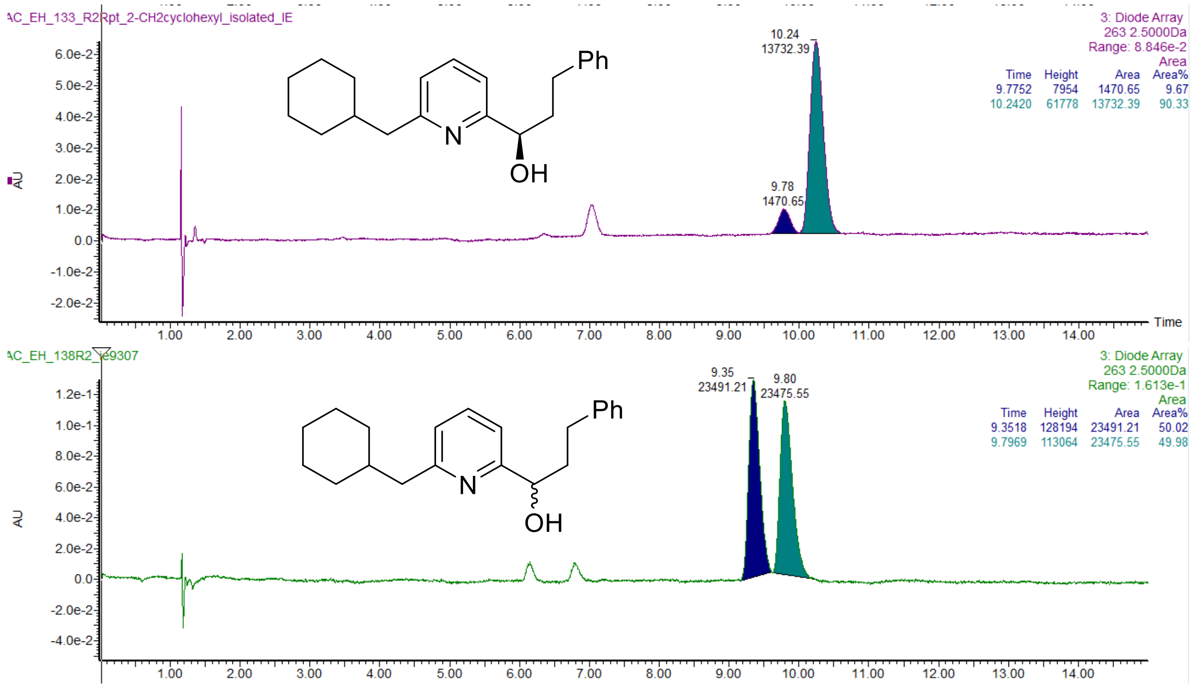

***tert*-butyl (*R*)-(2-(6-(1-hydroxy-3-phenylpropyl)pyridin-2-yl)ethyl)carbamate (2f)**

SFC Analysis: CHIRAL ART SC (CO_2/MeOH = 90/10, 2.5 mL min^{-1}, 40 °C, 262 nm); **<u>86% ee</u>**

t_R = 7.2 (minor), 7.6 (major) minutes

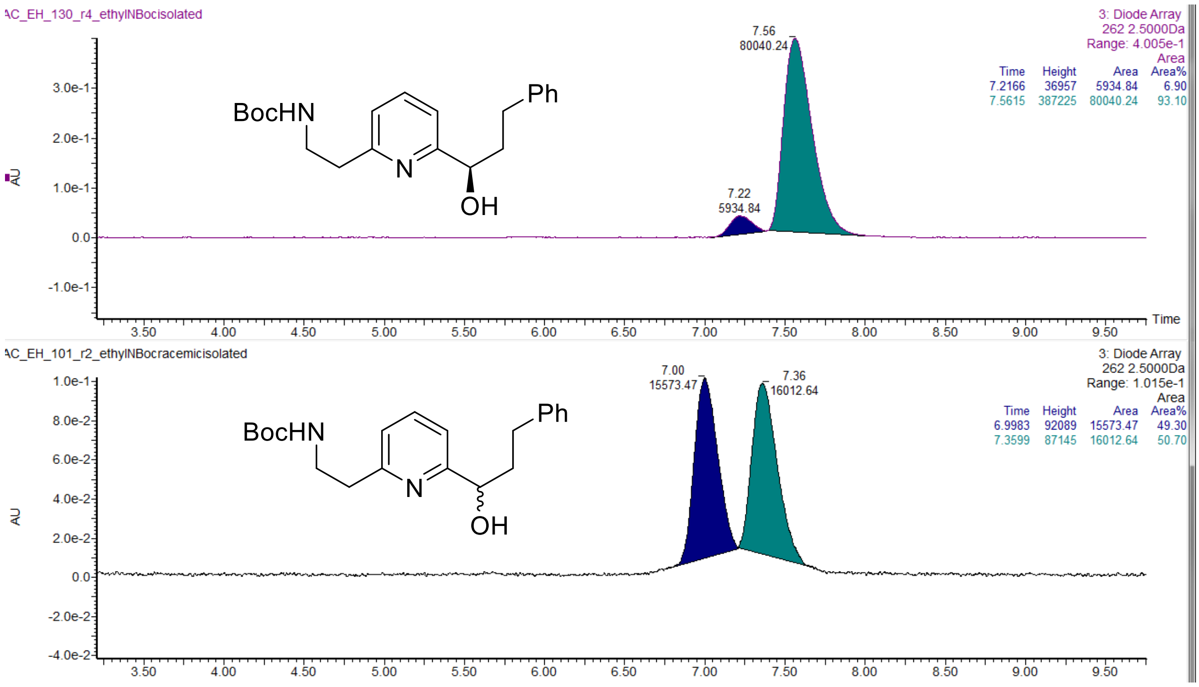

(*R*)-1-(6-(2-hydroxyethyl)pyridin-2-yl)-3-phenylpropan-1-ol (2ga)

SFC Analysis: Chiralpak IG (CO_2/MeOH = 80/20, 2.5 mL min^{-1}, 40 °C, 263 nm); **82% ee**

t_R = 7.8 (major), 10.0 (minor) minutes

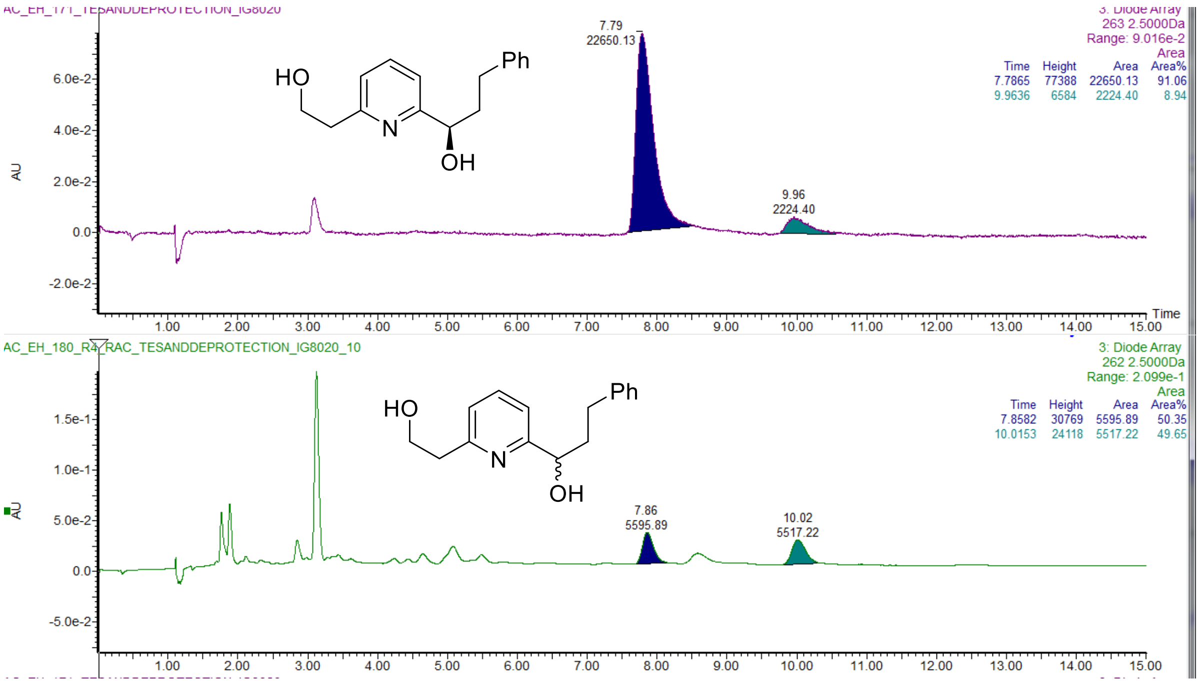

(*R*)-3-phenyl-1-(6-(3-phenylpropyl)pyridin-2-yl)propan-1-ol (2h)

SFC Analysis: Chiralpak IE (CO_2/MeOH = 90/10, 2.5 mL min^{-1}, 40 °C, 263 nm); **82% ee**

t_R = 10.2 (minor), 10.8 (major) minutes.

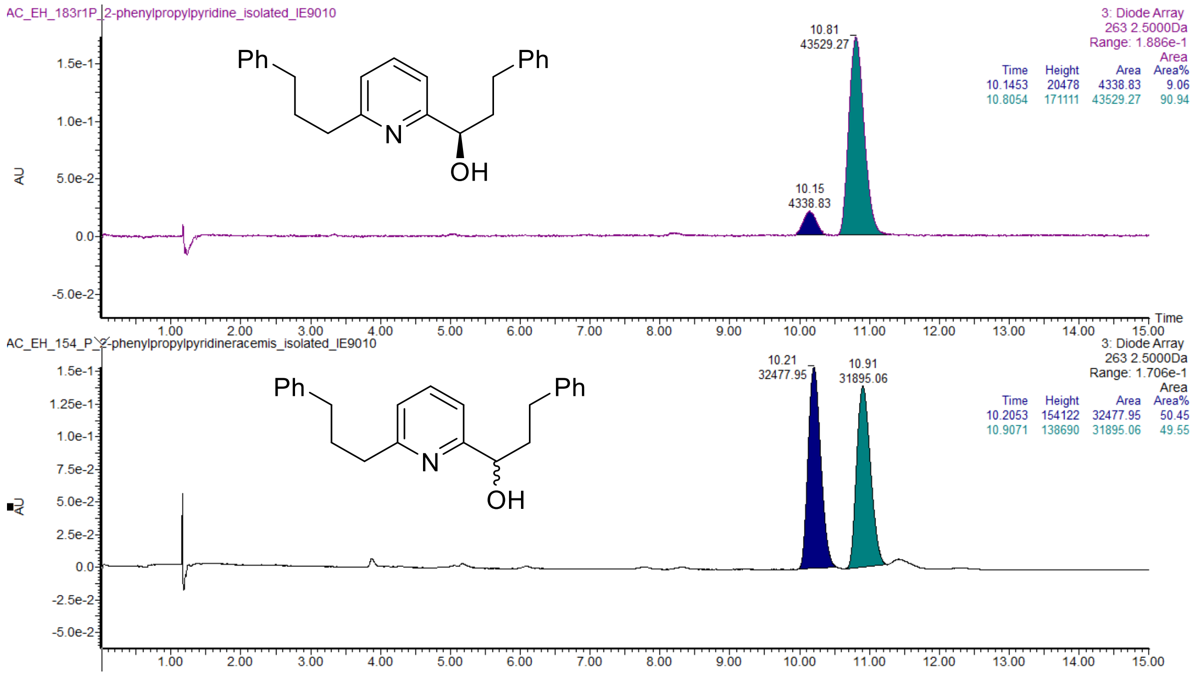

(*R*)-1-(5,6-dimethylpyridin-2-yl)-3-phenylpropan-1-ol (2i)

SFC Analysis: Chiralpak IE (CO_2/MeOH = 85/15, 2.5 mL min^{-1}, 40 °C, 267 nm), **<u>83% ee</u>**

t_R = 4.6 (minor), 4.8 (major) minutes

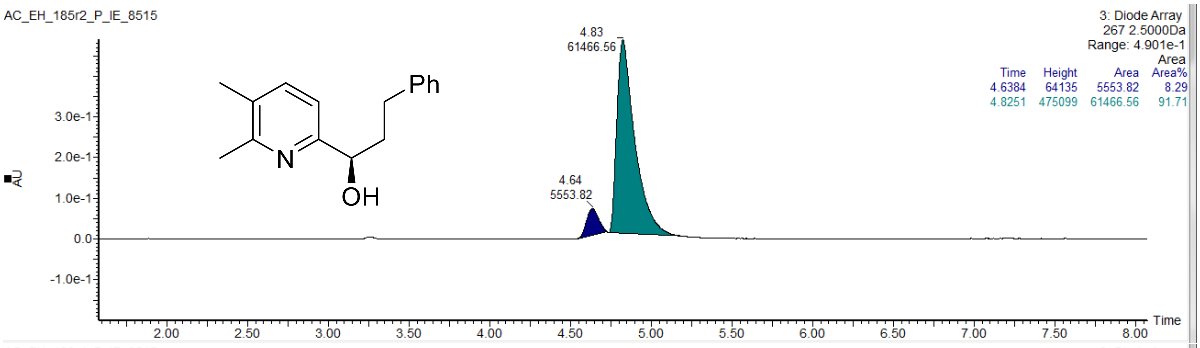

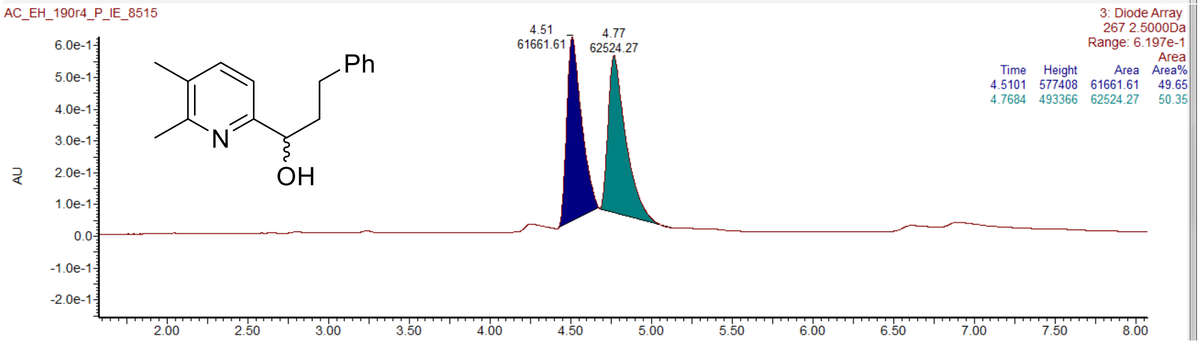

(*R*)-1-(6,7-dihydro-5H-cyclopenta-pyridin-2-yl)-3-phenylpropan-1-ol (2j)

SFC Analysis: Chiralpak IH (CO_2/MeOH = 93/07, 2.5 mL min^{-1}, 40 °C, 273 nm), **<u>88% ee</u>**

t_R = 6.1 (major), 7.5 (minor) minutes

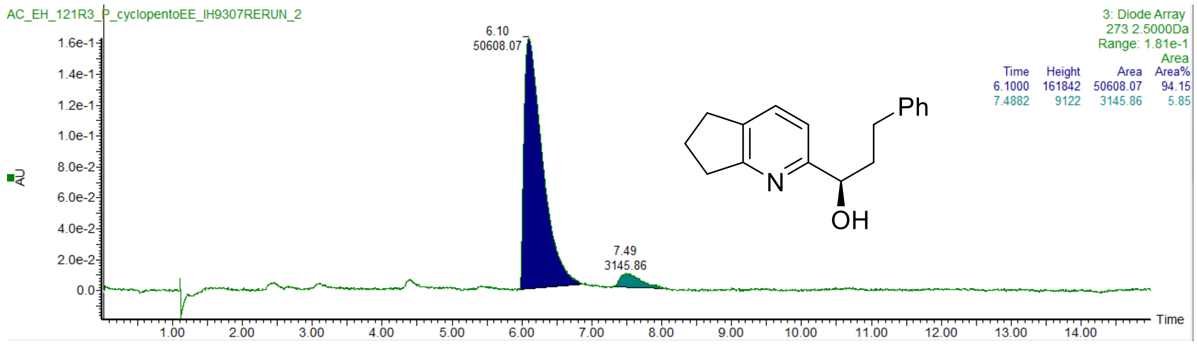

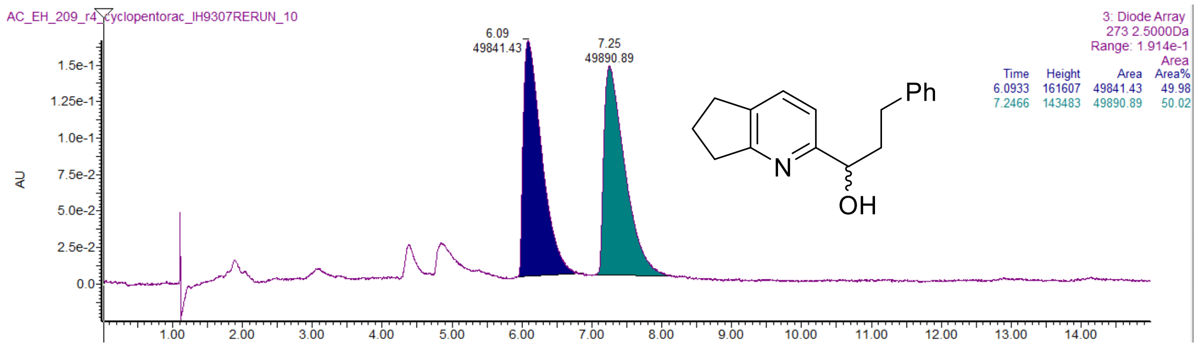

(*R*)-3-phenyl-1-(5,6,7,8-tetrahydroquinolin-2-yl)propan-1-ol (2k)

SFC Analysis: CHIRAL ART SC (CO_2/MeOH = 93/07, 2.5 mL min^{-1}, 40 °C, 270 nm); **88% ee**

t_R = 11.1 (minor), 11.7 (major) minutes

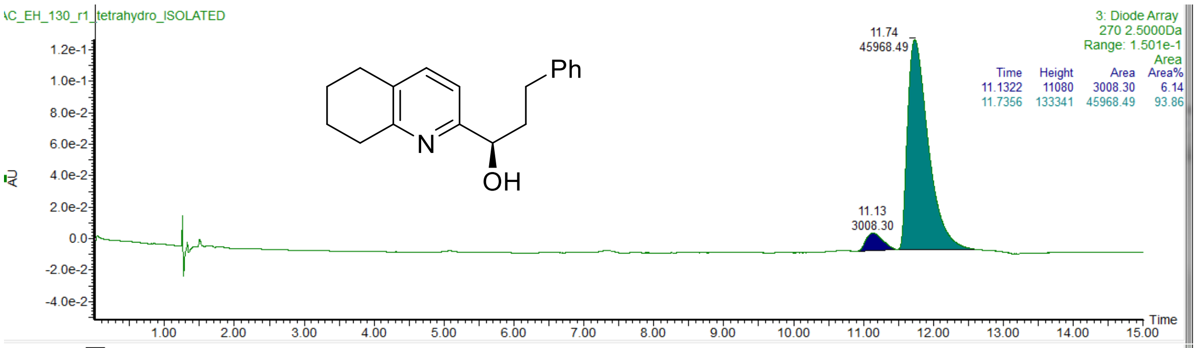

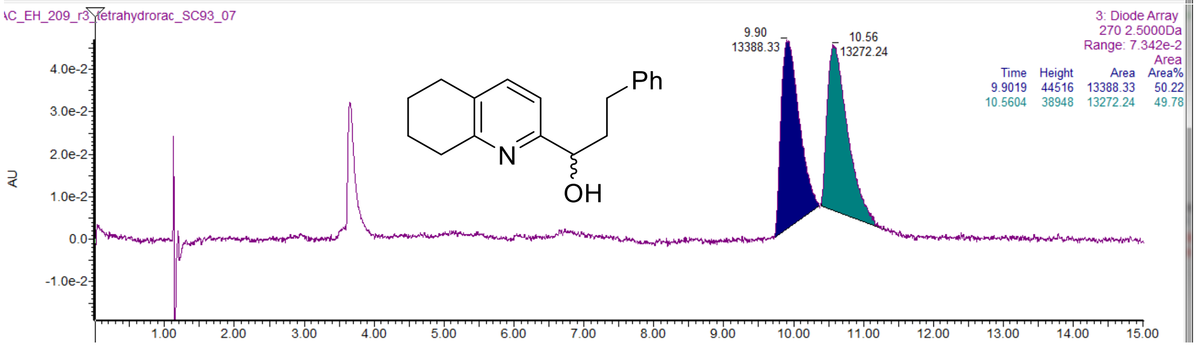

(*R*)-1-(5,6-dimethylpyridin-2-yl)-3-phenylpropan-1-ol (2l)

. **SFC Analysis:** Chiralpak IG(CO_2/MeOH = 90/10, 2.5 mL min^{-1}, 40 °C, 261 nm), **78% ee**

t_R = 6.8 (minor), 7.9 (major) minutes.

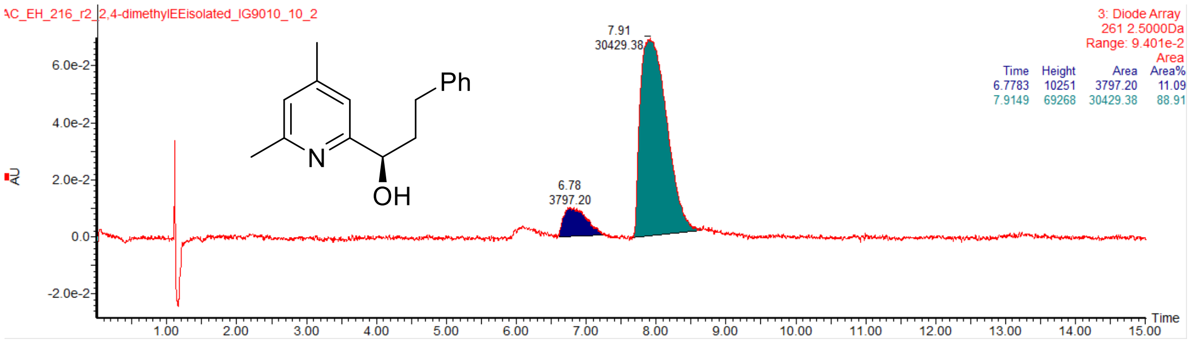

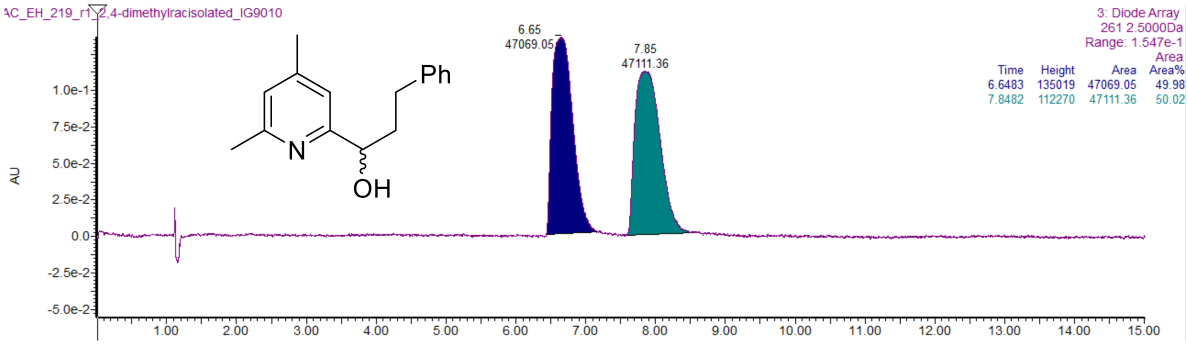

(*R*)-1-(3,5-dimethylpyridin-2-yl)-3-phenylpropan-1-ol (2m)

SFC Analysis: Chiralpak IG (CO_2/MeOH = 85/15, 2.5 mL min^{-1}, 40 °C, 267 nm); **94% ee**

t_R = 6.7 (major), 8.6 (minor) minutes

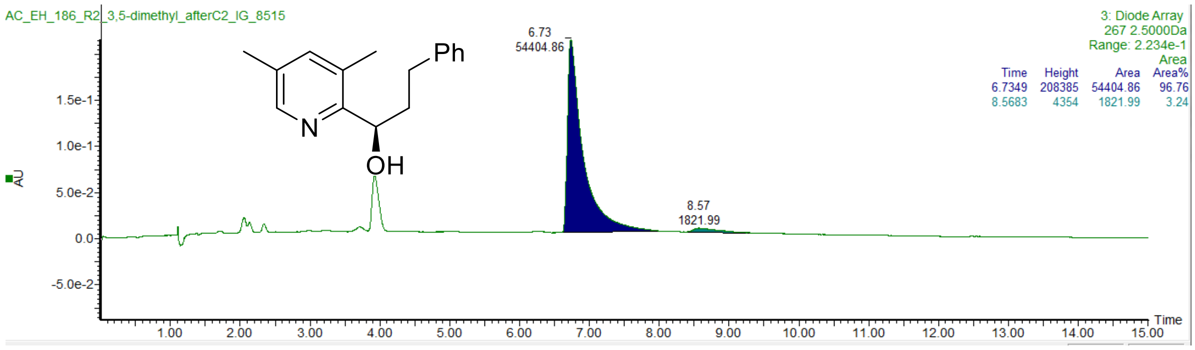

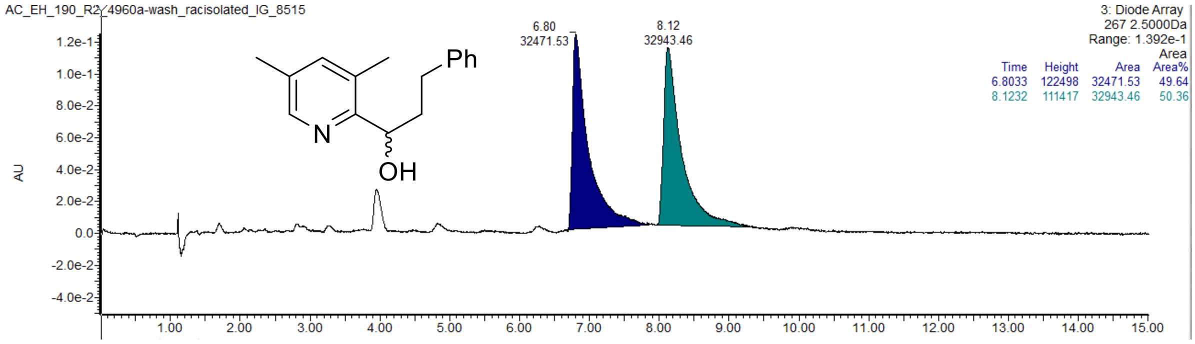

(*R*)-1-(3,6-dimethylpyridin-2-yl)-3-phenylpropan-1-ol (2n)

SFC Analysis: Chiralpak IG (CO_2/MeOH = 95/05, 2.5 mL min^{-1}, 40 °C, 267 nm), **91% ee**

t_R = 10.4 (minor), 11.0 (major) minutes.

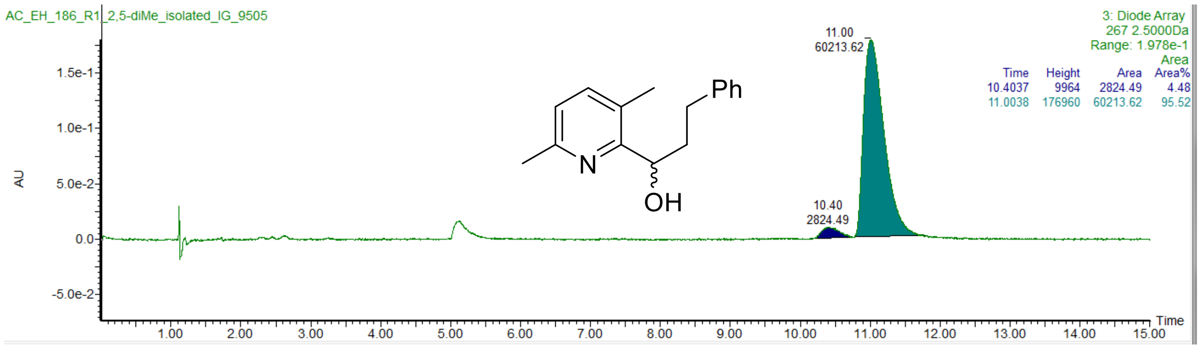

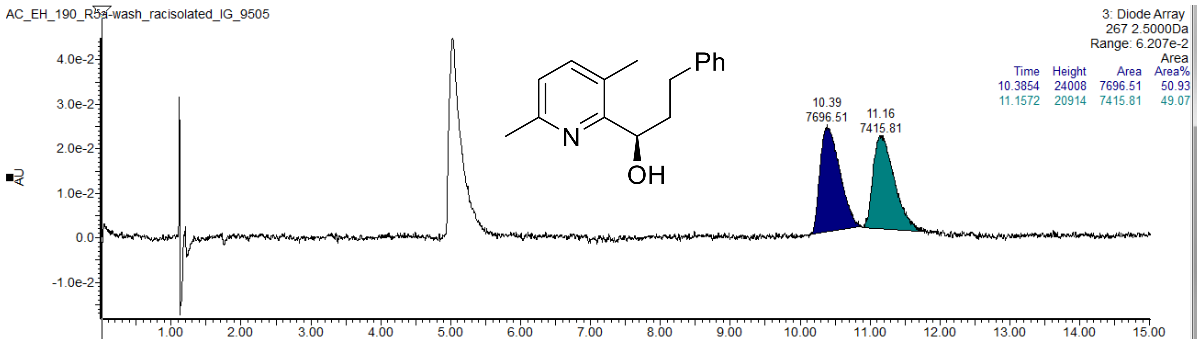

(*R*)-1-(3-methyl-5-phenylpyridin-2-yl)-3-phenylpropan-1-ol (2o)

SFC Analysis: Chiralpak IE (CO_2/MeOH = 75/25, 2.5 mL min^{-1}, 40 °C, 245); **<u>88% ee</u>**

t_R = 7.2 (major), 9.02 (minor) minutes.

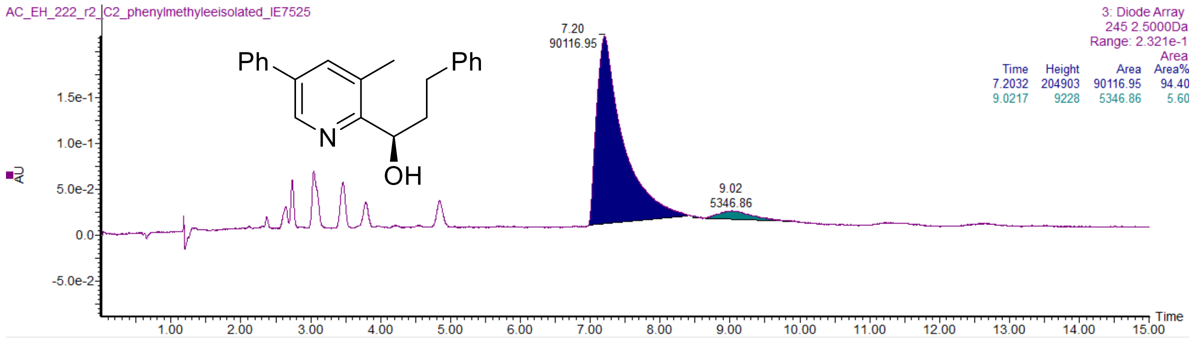

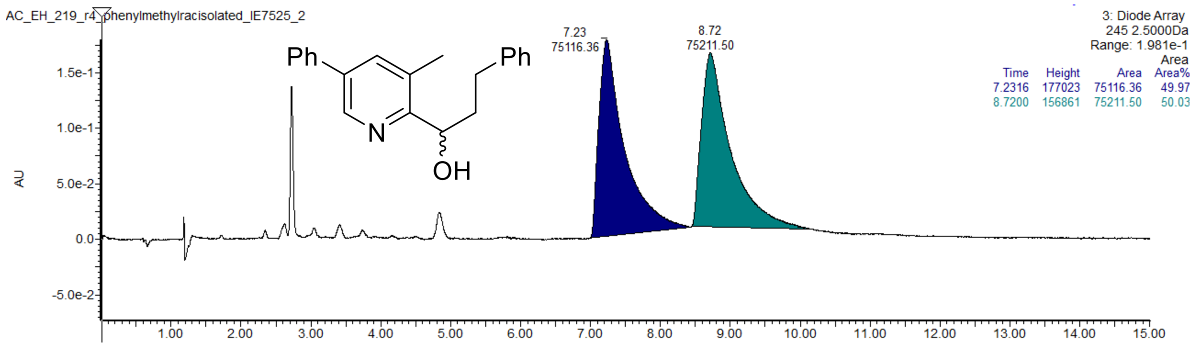

(*R*)-1-(3-methylpyridin-2-yl)-3-phenylpropan-1-ol (2pa)

SFC Analysis: Chiralpak IG (CO_2/MeOH = 85/15, 2.5 mL min^{-1}, 40 °C, 262 nm); **<u>90% ee</u>**

t_R = 5.3 (major), 6.9 (minor) minutes

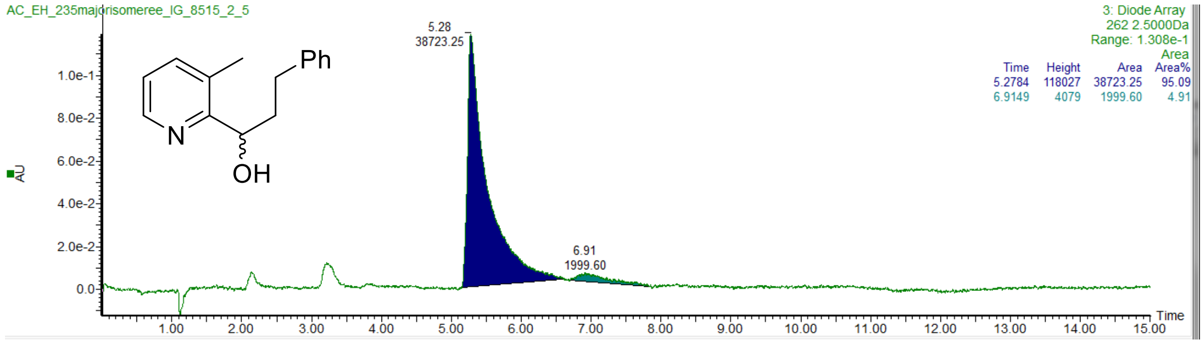

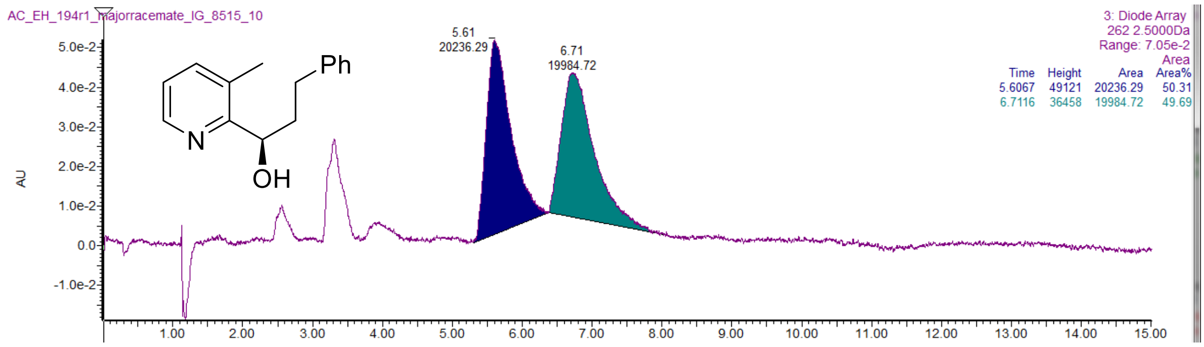

(*R*)-1-(5-methylpyridin-2-yl)-3-phenylpropan-1-ol (2pb)

SFC Analysis: Chiralpak IG (CO_2/MeOH = 85/15, 2.5 mL min^{-1}, 40 °C, 265 nm), **76% ee**

t_R = 8.0 (major), 9.5 (minor) minutes

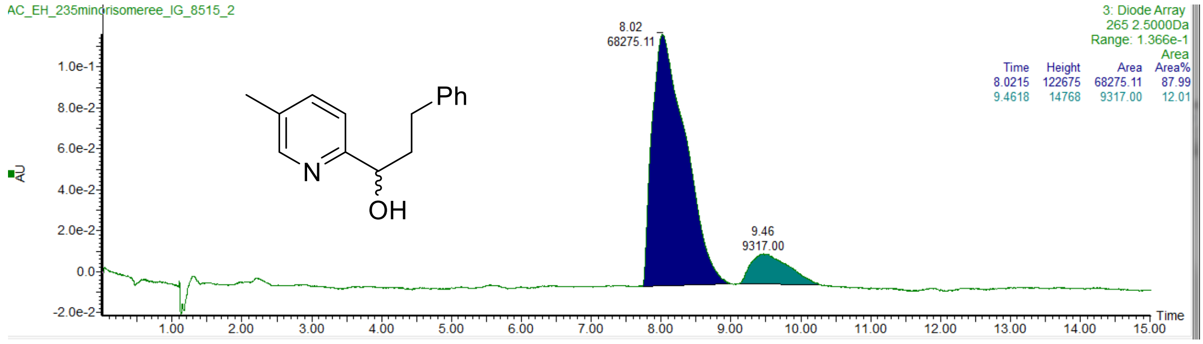

Time	Height	Area	Area%
8.0215	122675	68275.11	87.99
9.4618	14768	9317.00	12.01

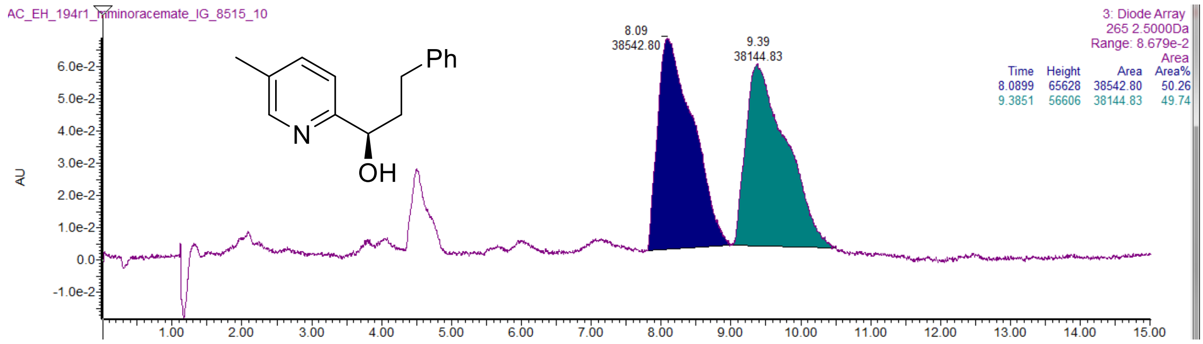

Time	Height	Area	Area%
8.0899	65628	38542.80	50.26
9.3851	56606	38144.83	49.74

***tert*-butyl (*R*)-((6-(1-hydroxy-3-phenylpropyl)-5-methylpyridin-2-yl)methyl)carbamate (2q)**

SFC Analysis: Chiralpak IE (CO_2/MeOH = 80/20, 2.5 mL min^{-1}, 40 °C, 266 nm); **90% ee**

t_R = 5.5 (major), 6.0 (minor) minutes

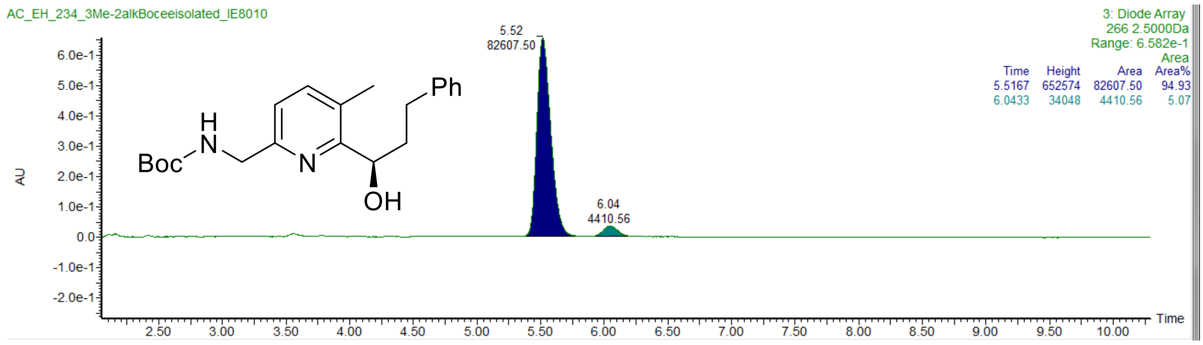

Time	Height	Area	Area%
5.5167	652574	82607.50	94.93
6.0433	34048	4410.56	5.07

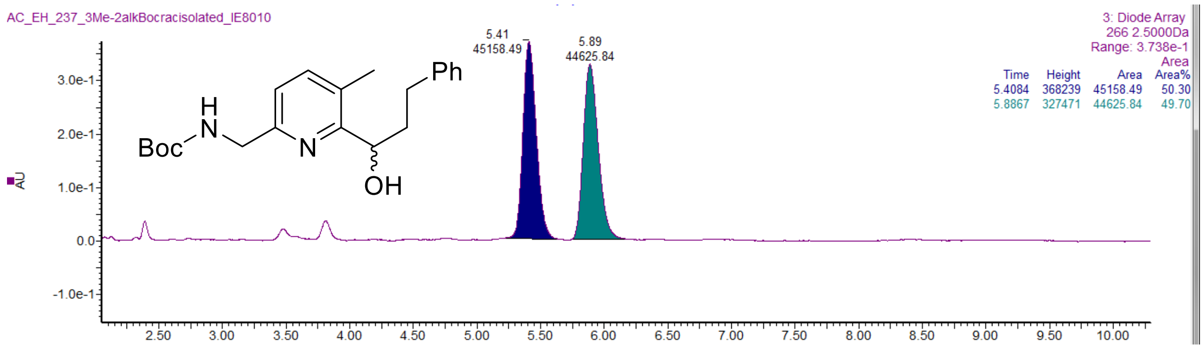

Time	Height	Area	Area%
5.4084	368239	45158.49	50.30
5.8867	327471	44625.84	49.70

(*R*)-1-(3-ethyl-6-methylpyridin-2-yl)-3-phenylpropyl acetate (2ra)

SFC Analysis: Chiral ART SC (CO_2/MeOH = 97/03, 1.5 mL min^{-1}, 40 °C. 267 nm); **<u>90% ee</u>**

t_R = 7.5 (minor), 8.0 (major) minutes.

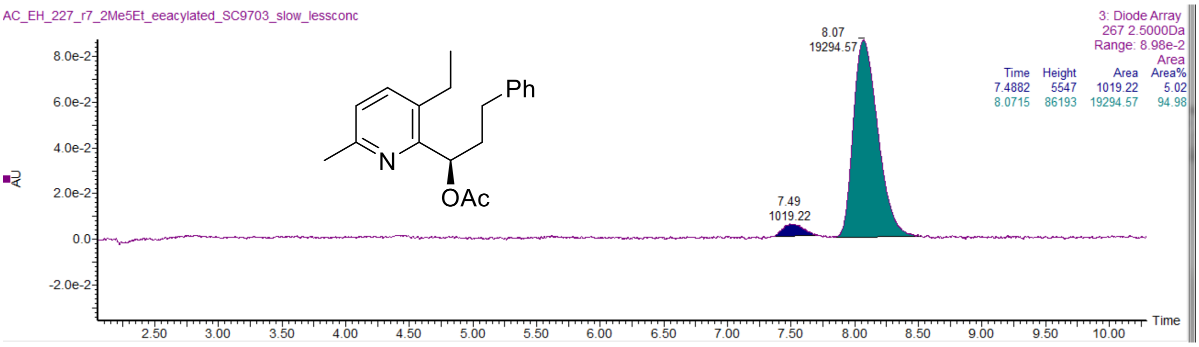

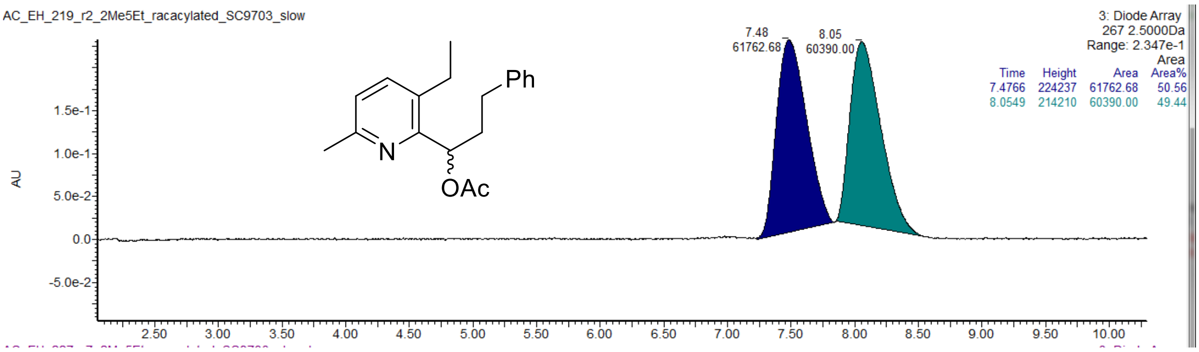

(*R*)-3-phenyl-1-(pyridin-2-yl)propyl acetate (2sa)

SFC Analysis: Chiralpak IE (CO_2/MeOH = 95/05, 2.5 mL min^{-1}, 40 °C, 258 nm); **<u>73% ee</u>**

t_R = 7.9 (major), 9.8 (minor) minutes.

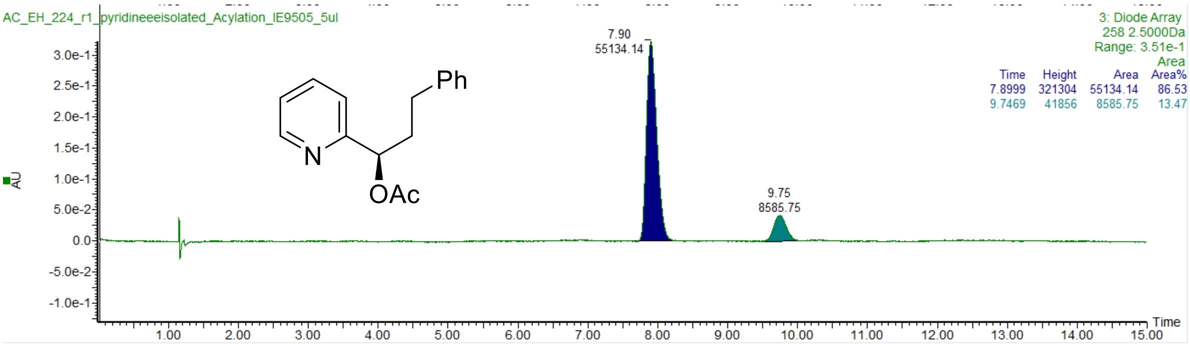

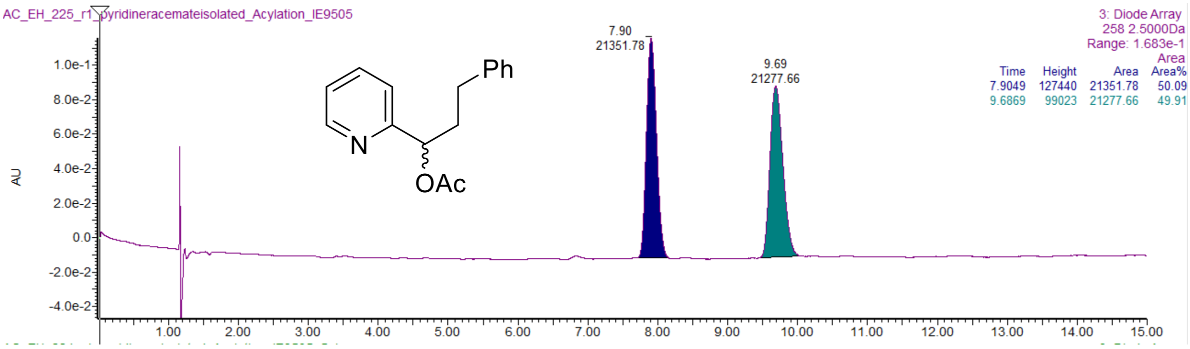

(1*R*,1'*R*)-pyridine-2,6-diylbis(3-phenylpropane-1,1-diyl) diacetate (2ua)

SFC Analysis: Chiralpak IE (CO_2/MeOH = 90/10, 2.5 mL min^{-1}, 40 °C, 262 nm); **95% ee**

t_R = 5.4 (major), 5.8 (minor) minutes.

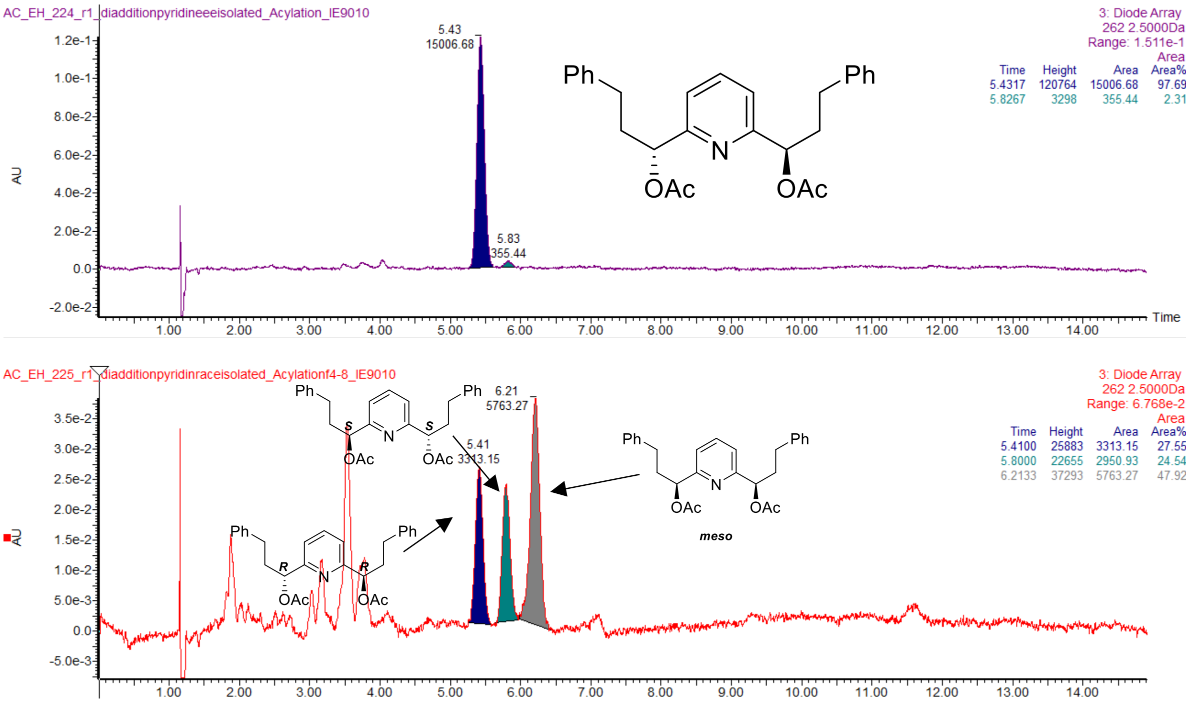

tert-butyl (2-(1-hydroxy-3-phenylpropyl)pyridin-3-yl)carbamate **2xa**

SFC Analysis: CHIRALCEL IG (CO_2/MeOH = *85/15*, 2.5 mL min^{-1}, 40 °C) t_R = *2.7* (minor), *3.0* (major) minutes.

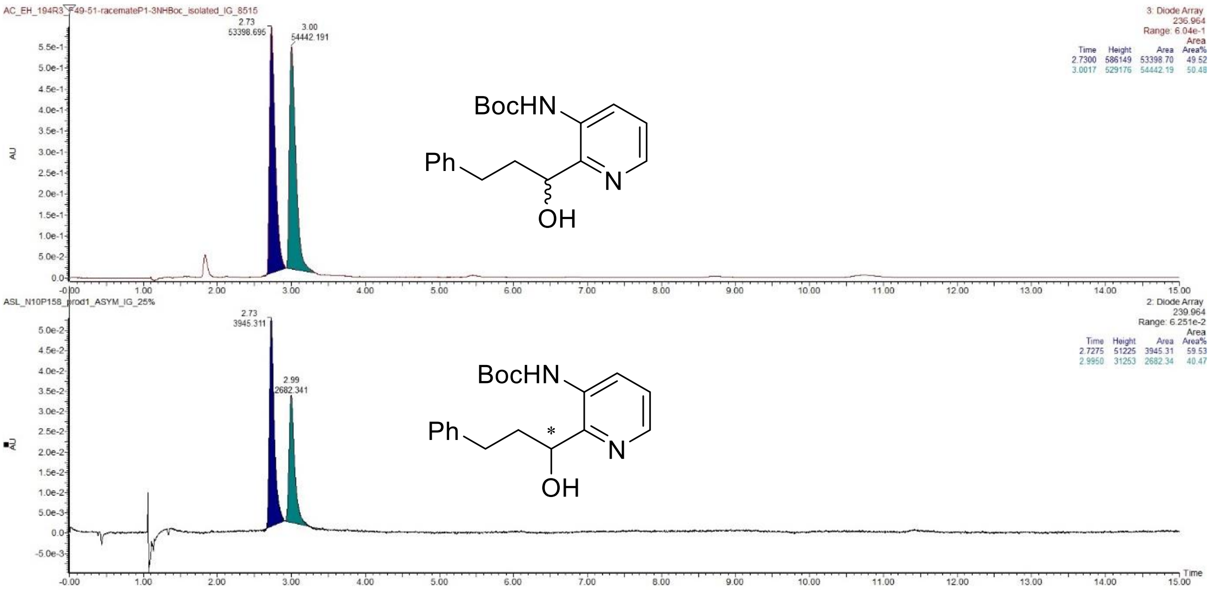

tert-Butyl (*R*)-(6-(1-hydroxy-3-phenylpropyl)pyridin-3-yl) **2x**

SFC Analysis: Chiralpak IE (CO_2/MeOH = 70/30, 2.5 mL min^{-1}, 40 °C) t_R = 3.5 (major), 4.6 (minor) minutes.

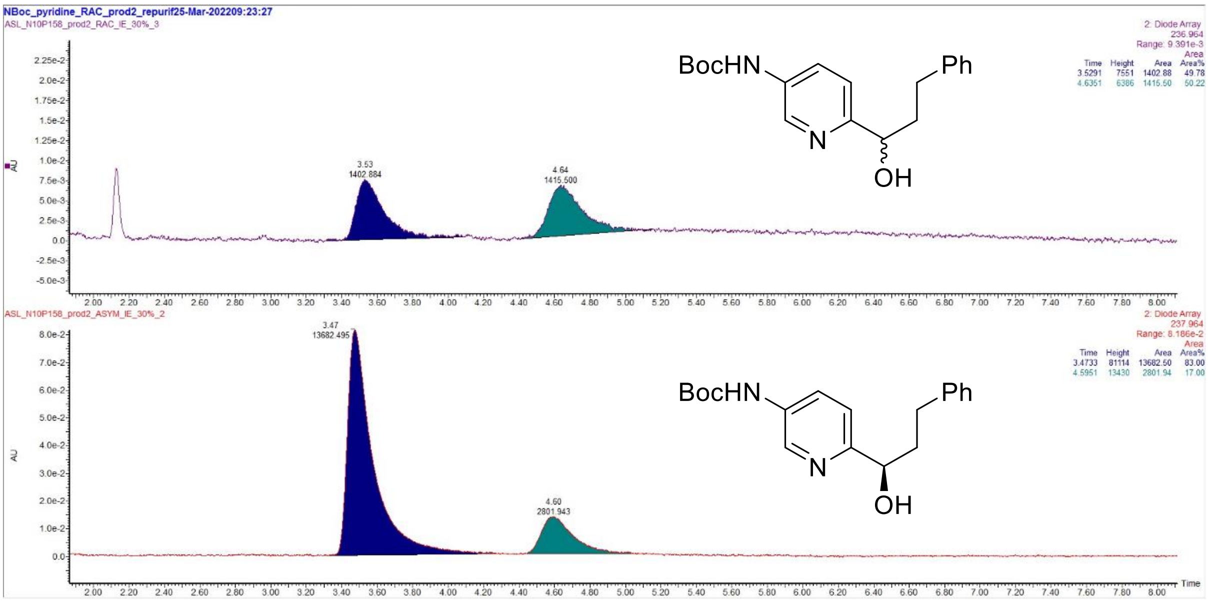

(*R*)-1-(4-(tert-butyl)pyridin-2-yl)-3-phenylpropyl acetate

SFC Analysis: Chiralpak SC (CO_2/MeOH = 95/05, 2.5 mL min^{-1}, 40 °C, 257 nm); <u>**84% ee**</u>

t_R = 4.2 (major), 4.7 (minor) minutes

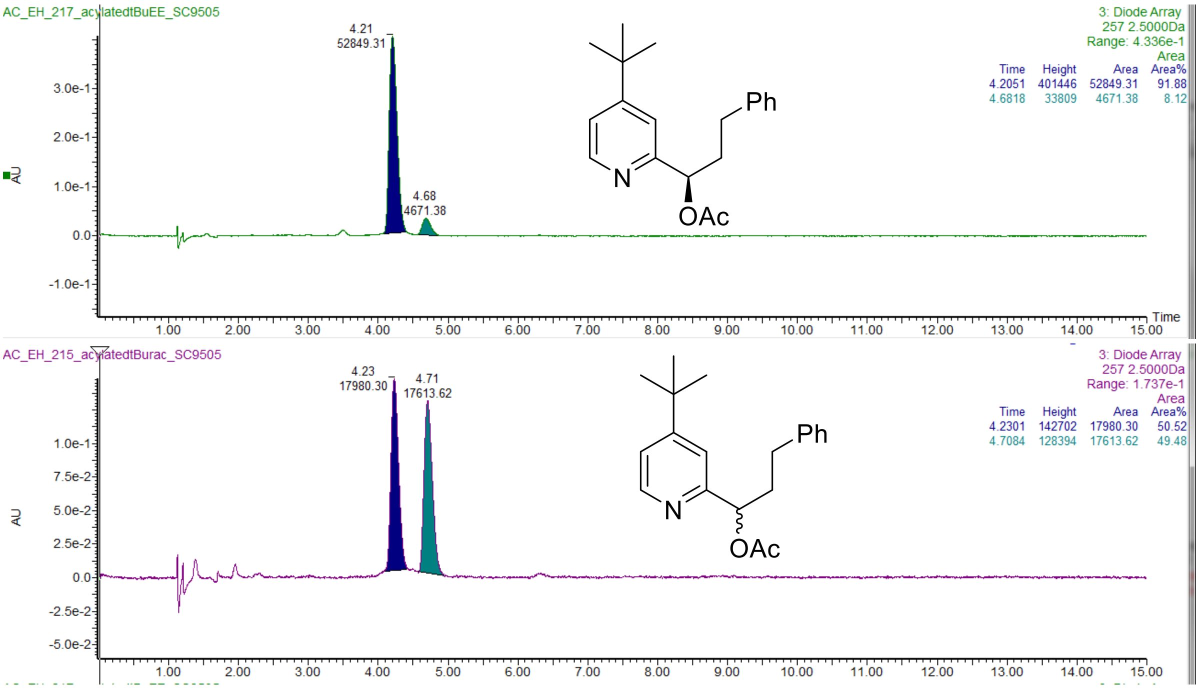

(*R*)-3-(2-Bromophenyl)-1-(6-pentylpyridin-2-yl)propan-1-ol (6a)

SFC Analysis: CHIRAL ART SC (CO_2/MeOH = 97/3, 2.5 ml min^{-1}, 40 °C, 263 nm); **84% ee**

t_R = 12.1 (major), 14.3 (minor) minutes

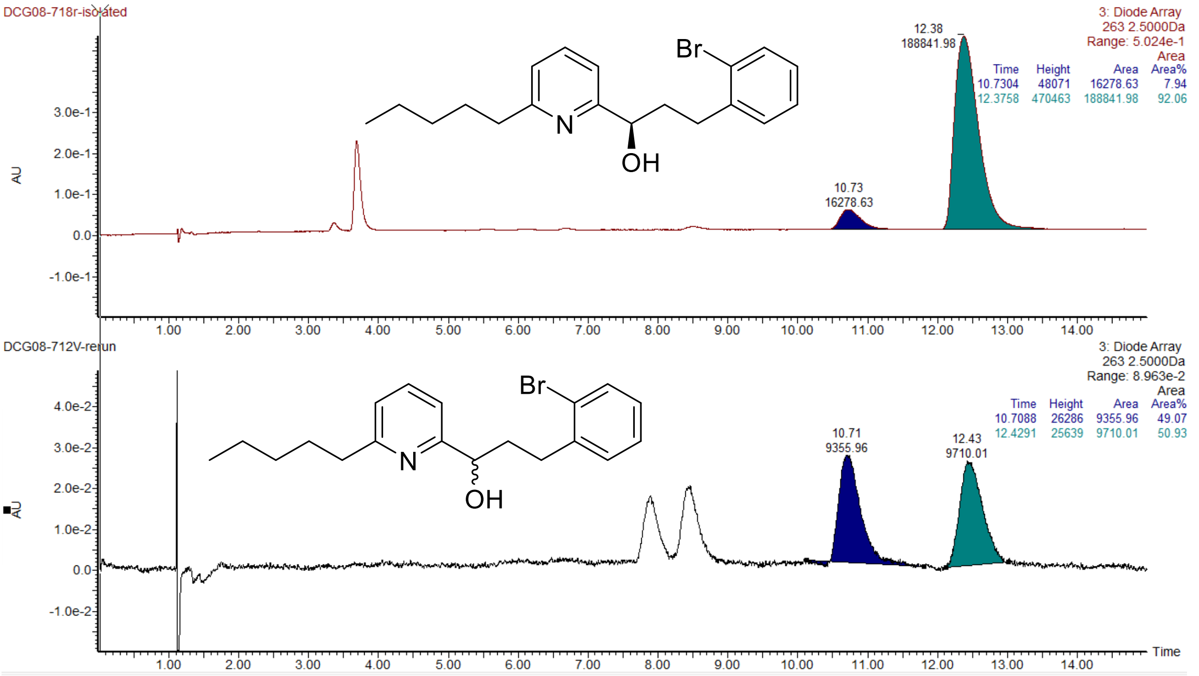

(*R*)-3-(2-methoxyphenyl)-1-(6-pentylpyridin-2-yl)propan-1-ol (6b)

SFC Analysis: CHIRAL ART SC (CO_2/MeOH = 93/7, 2.5 ml min^{-1}, 40 °C, 263 nm); **81% ee**

t_R = 7.1 (major), 7.9 (minor) minutes

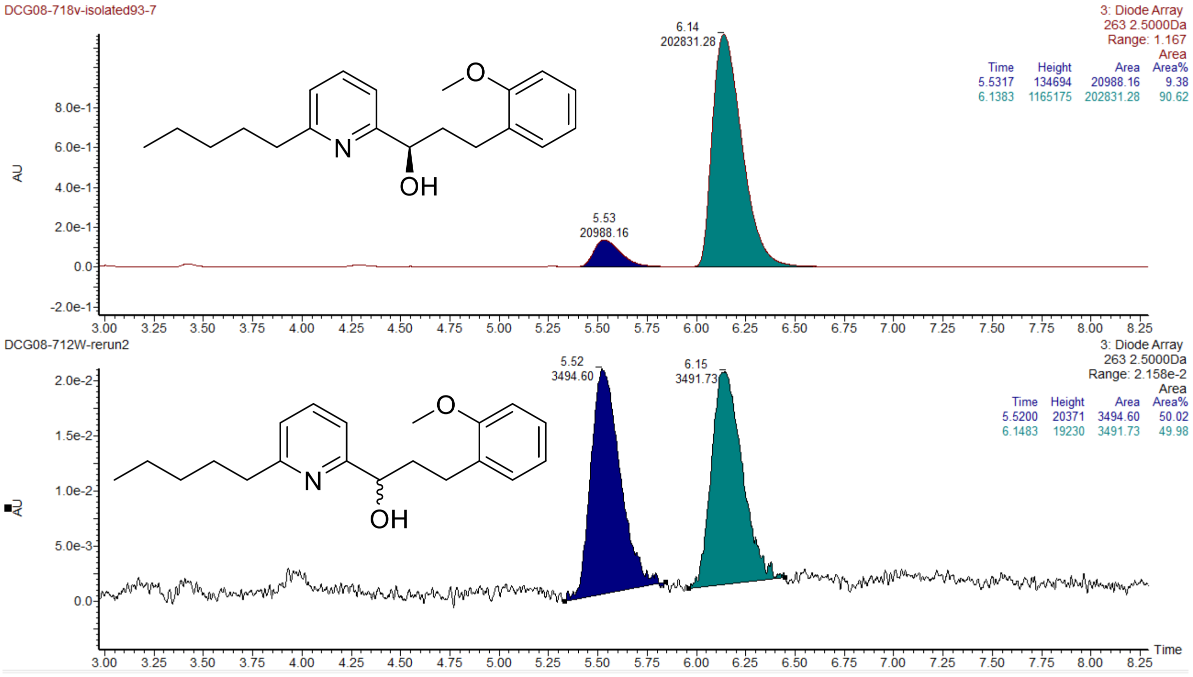

(*R*)-3-(4-bromophenyl)-1-(6-pentylpyridin-2-yl)propan-1-ol (6c)

SFC Analysis: CHIRAL ART SC (CO_2/MeOH = 97/3, 2.5 ml min^{-1}, 40 °C, 263 nm); **81% ee**

t_R = 11.3 (major), 12.4 (minor) minutes.

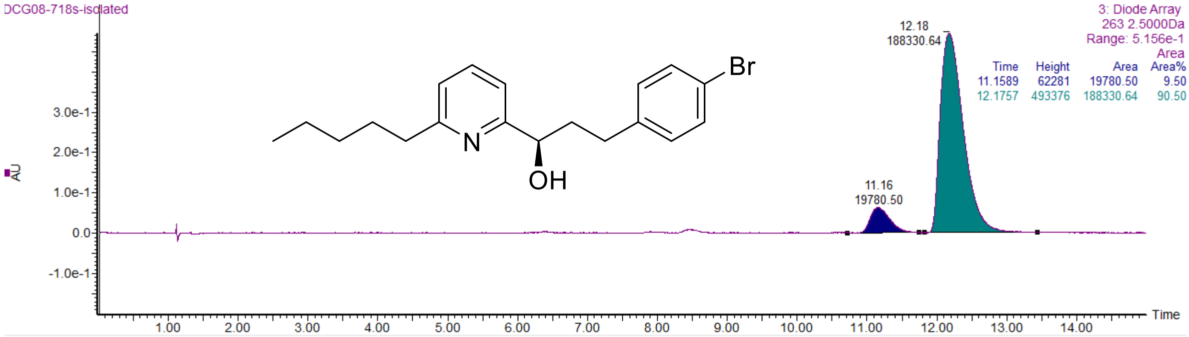

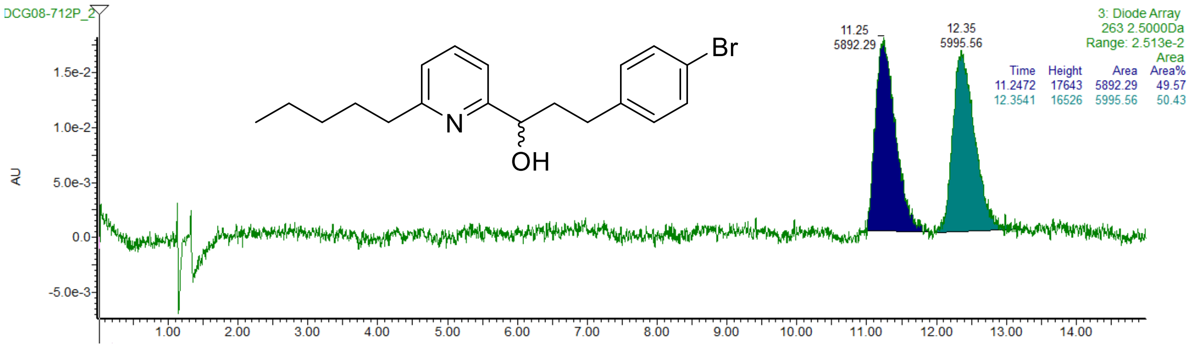

(*R*)-3-(2-fluorophenyl)-1-(6-pentylpyridin-2-yl)propan-1-ol (6d)

SFC Analysis: CHIRAL ART SC (CO_2/MeOH = 97/3, 2.5 ml min^{-1}, 40 °C, 200 nm); **86% ee**

t_R = 5.7 (minor), 6.2 (major) minutes

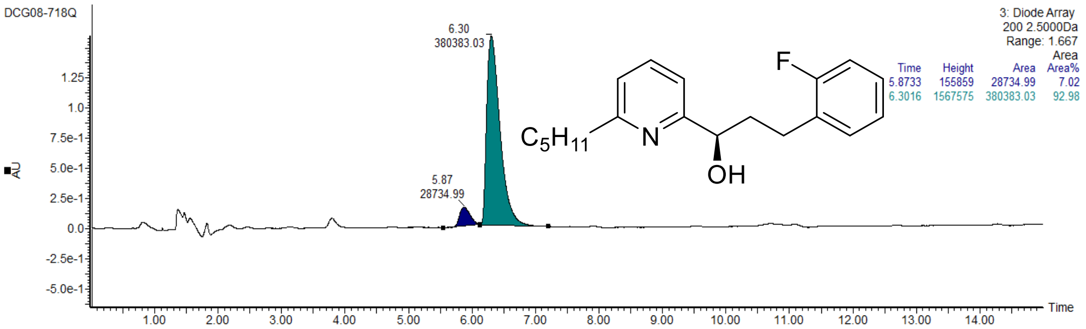

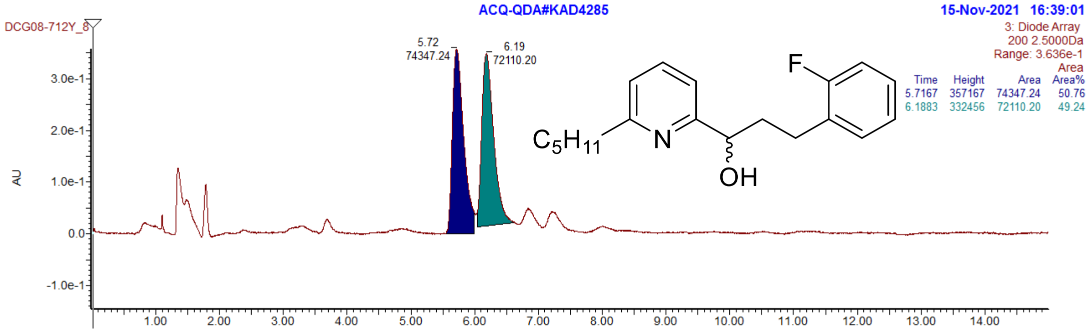

(*R*)-2-(2-fluorophenyl)-1-(6-pentylpyridin-2-yl)ethan-1-ol (6e)

SFC Analysis: CHIRALCEL IH (CO_2/MeOH = 98.5/1.5, 2.5 ml min^{-1}, 40 °C, 263 nm); **88% ee**

t_R = 5.6 (major), 6.3 (minor) minutes

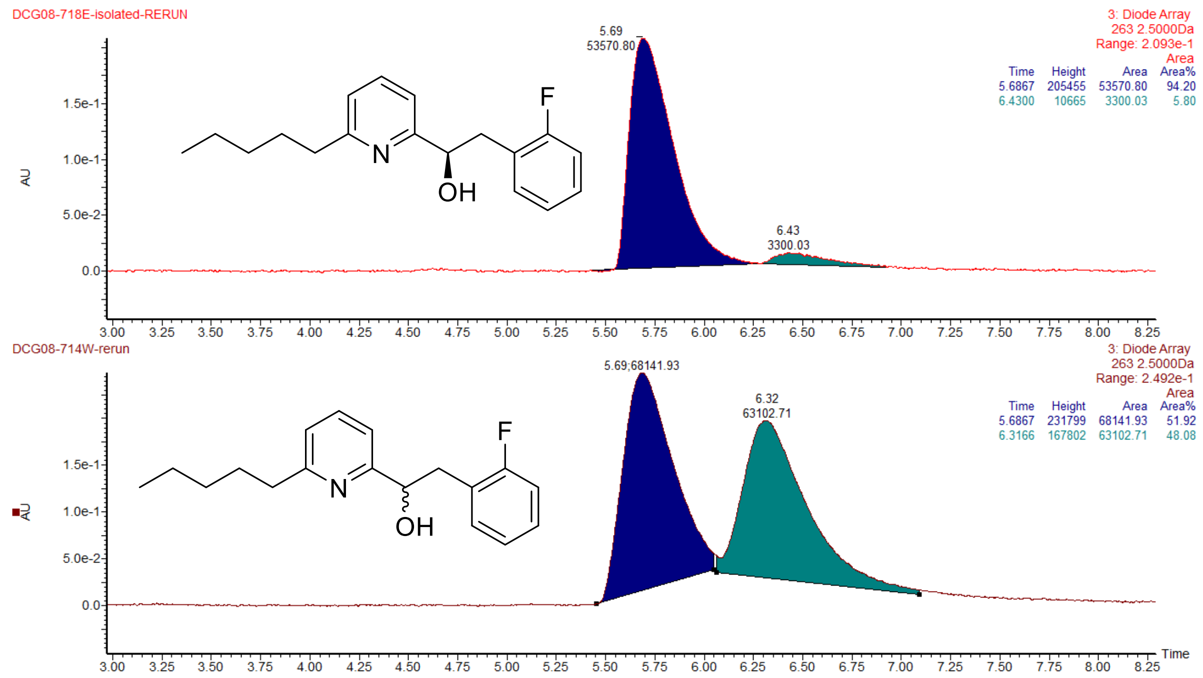

(*R*)-2-(4-Bromophenyl)-1-(6-pentylpyridin-2-yl)ethan-1-ol (6f)

SFC Analysis: CHIRAL ART SC (CO_2/MeOH = 95/5, 2.5 ml min^{-1}, 40 °C, 263 nm); **83% ee**

t_R = 5.7 (minor), 6.0 (major) minutes.

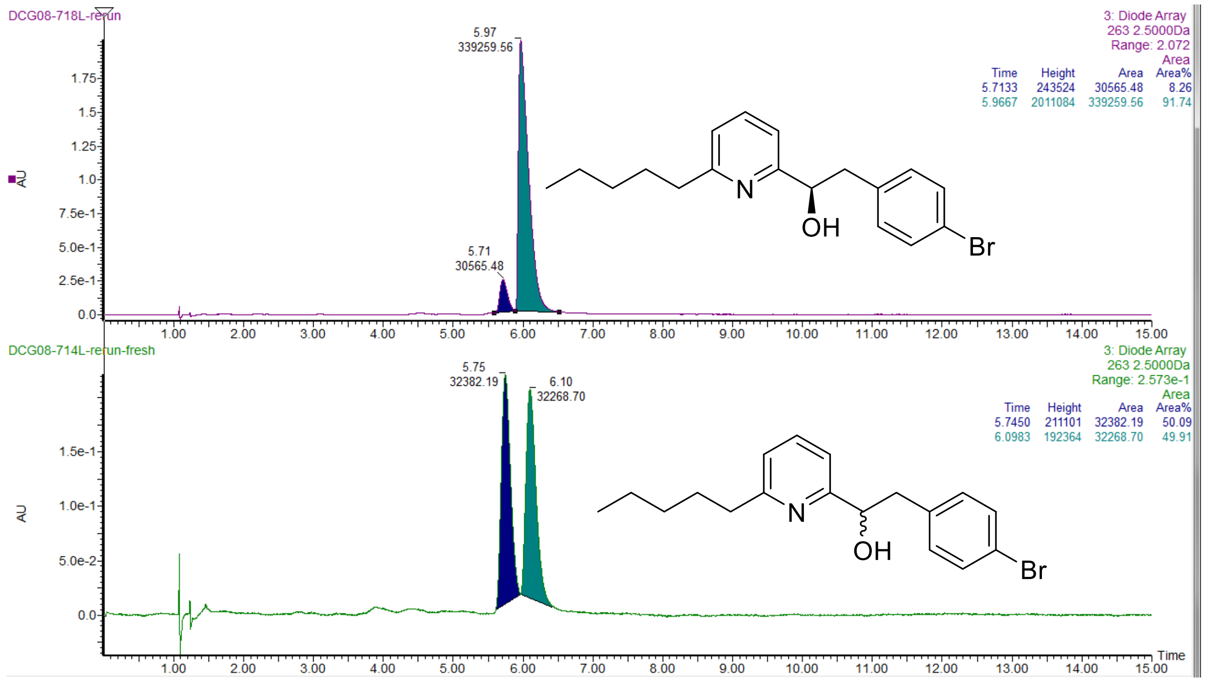

(*R*)-2-(2-tolyl)-1-(6-pentylpyridin-2-yl)ethan-1-ol (6g)

SFC Analysis: CHIRAL ART SJ (CO_2/MeOH = 99/1, 2.5 ml min^{-1}, 40 °C, 263 nm); **85% ee**

t_R = 6.1 (major), 6.8 (minor) minutes.

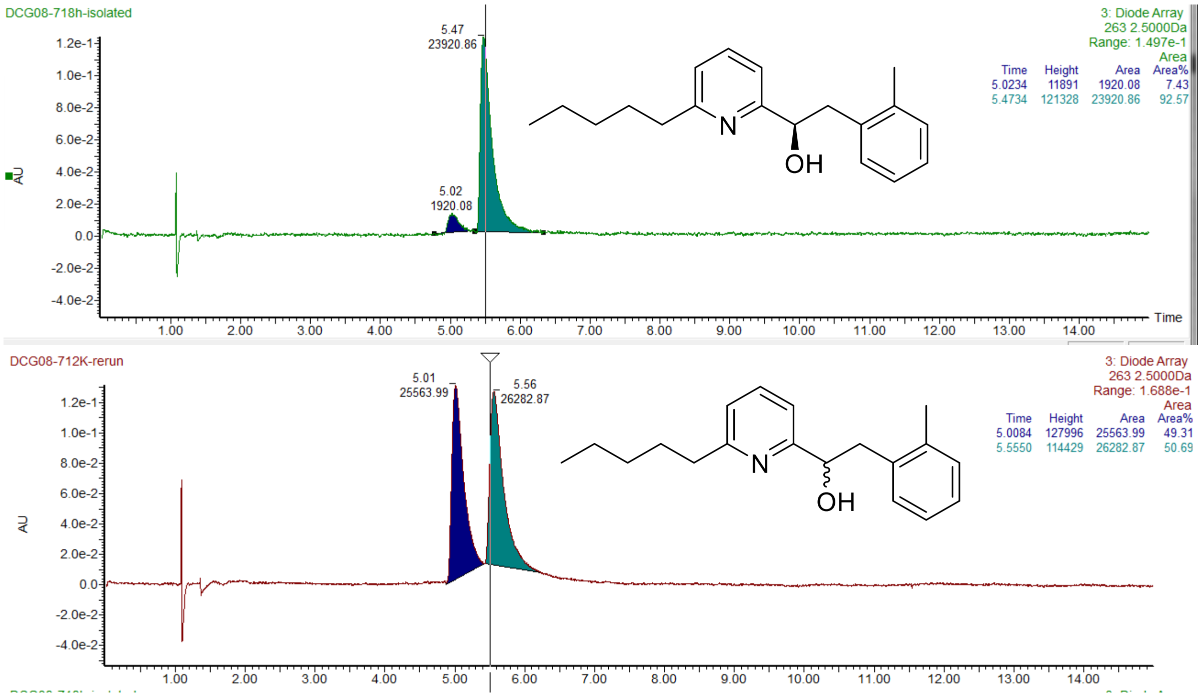

(*R*)-2-(4-Methoxyphenyl)-1-(6-pentylpyridin-2-yl)ethan-1-ol (6h)

SFC Analysis: CHIRAL ART SJ (CO_2/MeOH = 97/3, 2.5 ml min^{-1}, 40 °C, 263 nm); **84% ee**

t_R = 5.51 (minor), 5.9 (major) minutes.

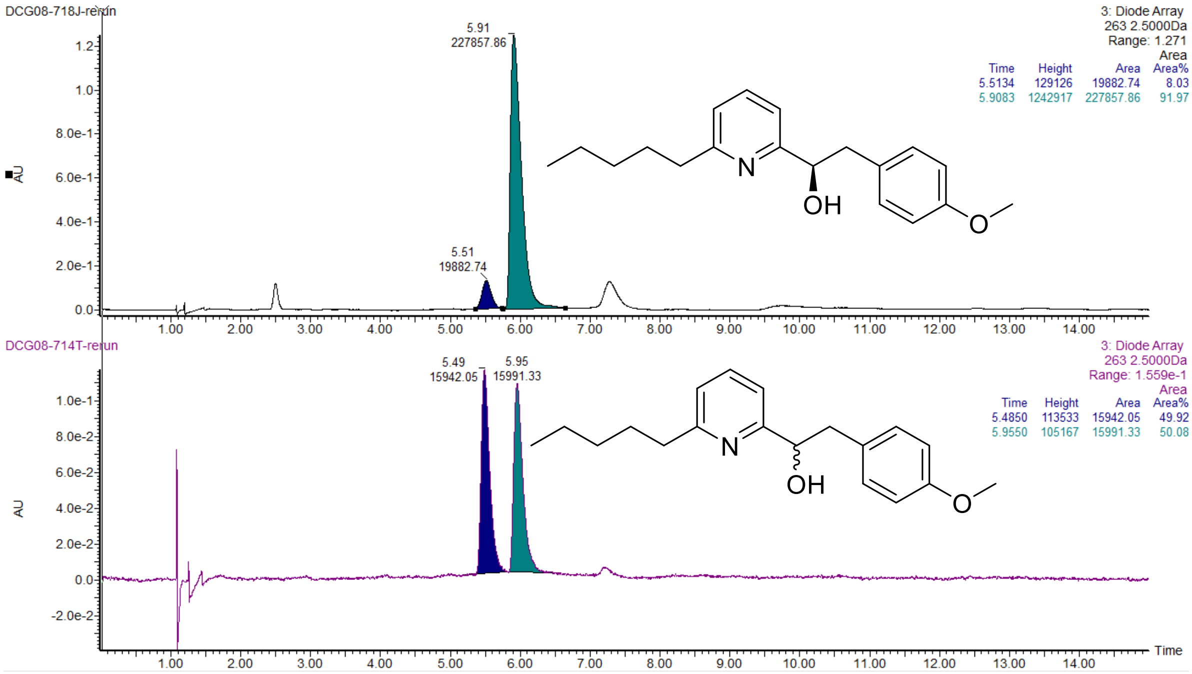

(*R*)-1-(6-pentylpyridin-2-yl)-4-phenylbutan-1-yl acetate (6ia)

SFC Analysis: CHIRALCEL IH (CO_2/MeOH = 98/2, 2.5 ml min^{-1}, 40 °C, 200 nm); **88% ee**

t_R = 3.8 (major), 4.3 (minor) minutes.

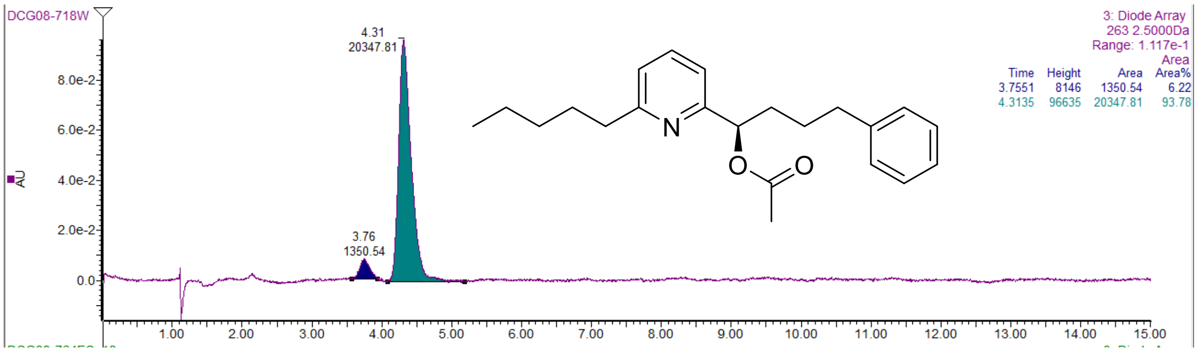

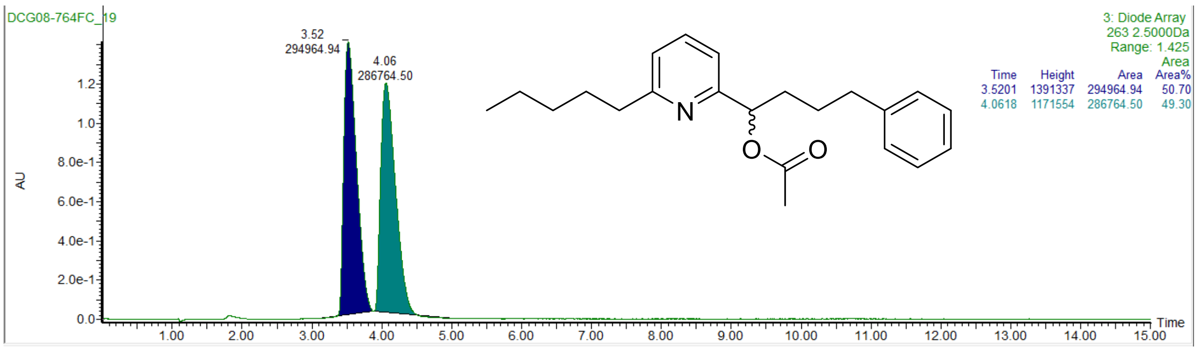

(*R*)-1-(6-pentylpyridin-2-yl)butan-1-ol (6j)

SFC Analysis: CHIRALCEL IG (CO_2/MeOH = 95/5, 2.5 ml min^{-1}, 40 °C, 263 nm); **84% ee**

t_R = 4.3 (major), 4.9 (minor) minutes.

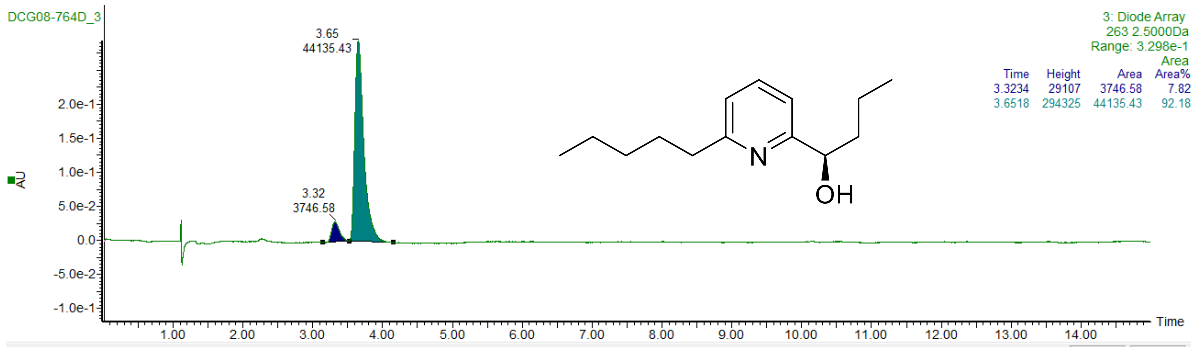

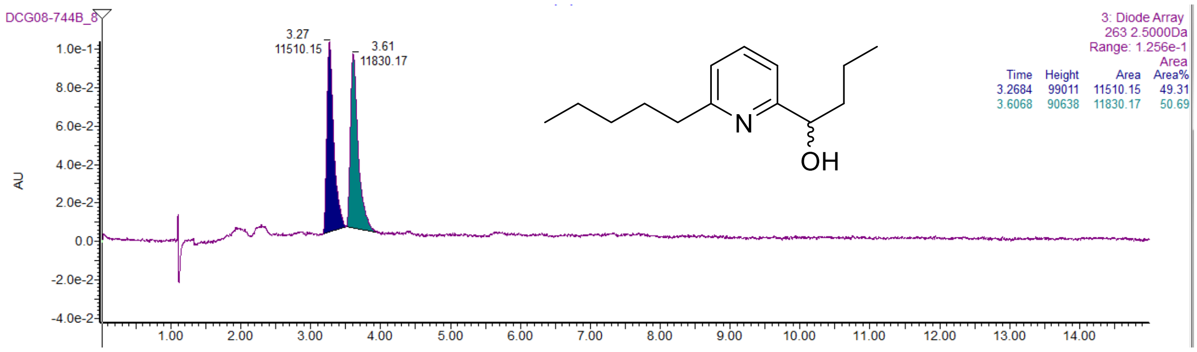

(*R*)-3-cyclohexyl-1-(6-pentylpyridin-2-yl)propan-1-ol (6k)

SFC Analysis: CHIRAL ART SC (CO_2/MeOH = 98/2, 2.5 ml min^{-1}, 40 °C, 263 nm); **<u>85% ee</u>**

t_R = 9.1 (major), 11.5 (minor) minutes.

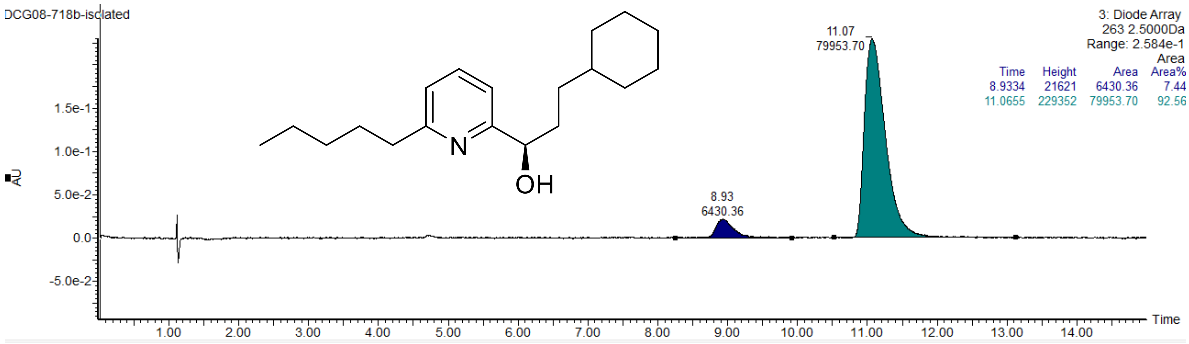

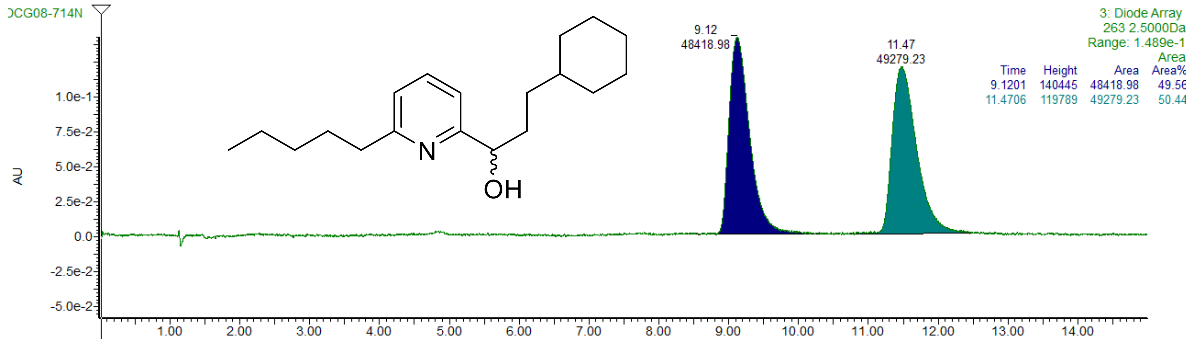

(*R*)-cyclobutyl(6-pentylpyridin-2-yl)methanol (6l)

SFC Analysis: CHIRAL ART SC (CO_2/MeOH = 98/2, 2.5 ml min^{-1}, 40 °C, 263 nm); **<u>88% ee</u>**

t_R = 6.4 (major), 7.2 (minor) minutes.

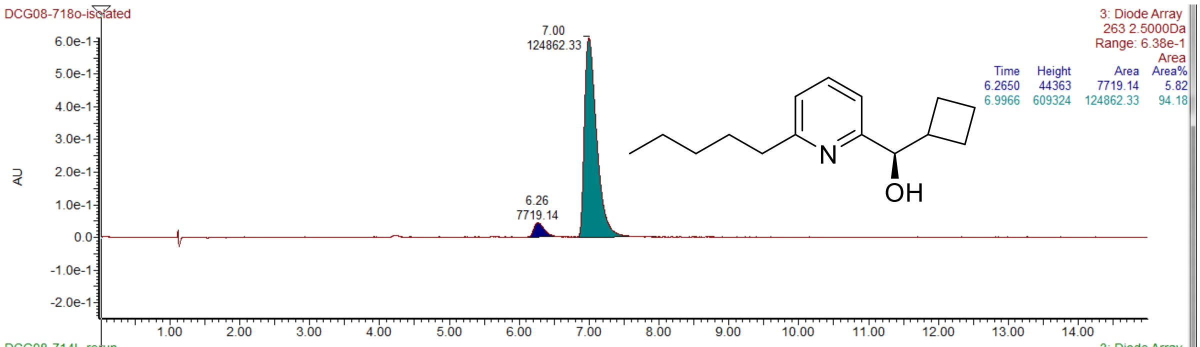

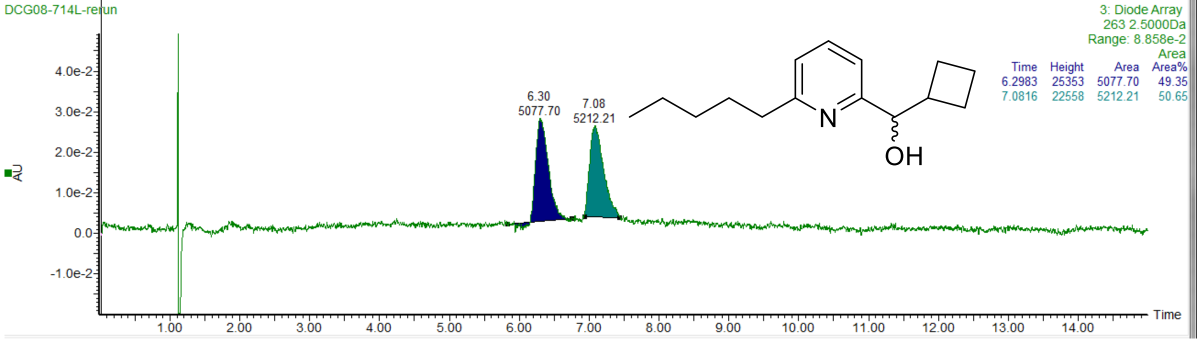

(*R*)-1-(6-pentylpyridin-2-yl)but-3-yn-1-ol (6m)

SFC Analysis: CHIRAL ART SC (CO_2/MeOH = 98/2, 2.5 ml min^{-1}, 40 °C, 263 nm); <u>**86% ee**</u>

t_R = 4.6 (major), 5.1 (minor) minutes.

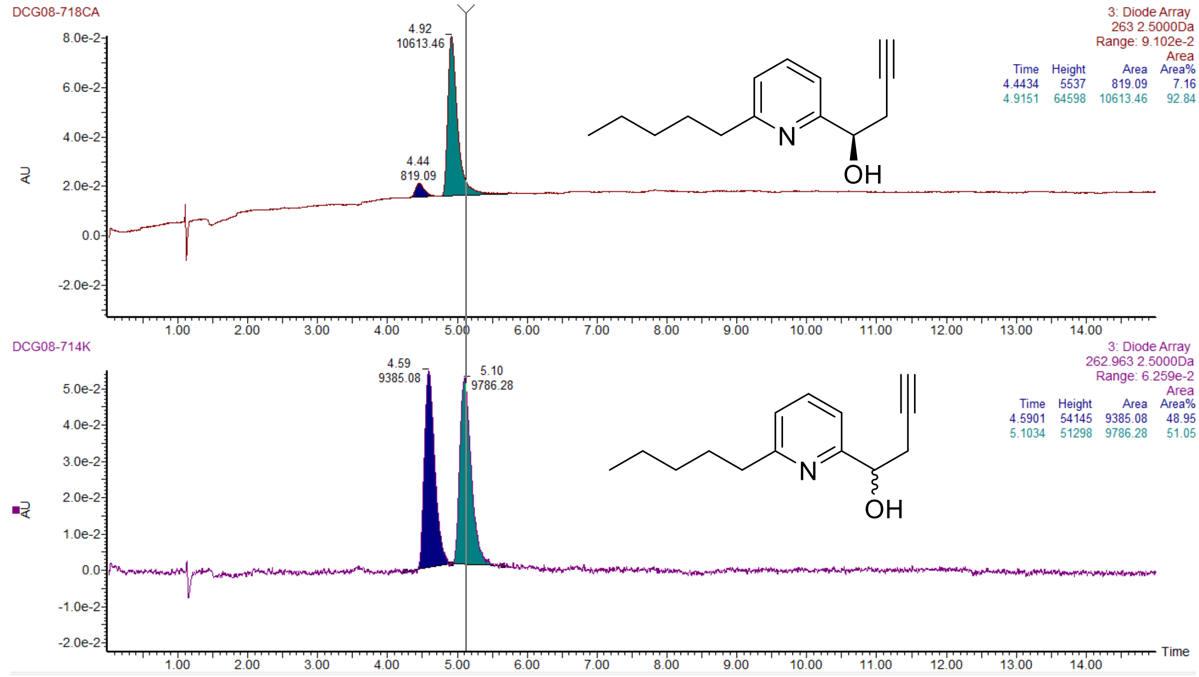

(*R*)-1-(6-pentylpyridin-2-yl)-4-(trimethylsilyl)but-3-yn-1-ol (6n)

SFC Analysis: CHIRAL ART SC (CO_2/MeOH = 99/1, 2.5 ml min^{-1}, 40 °C, 263 nm); <u>**90% ee**</u>

t_R = 3.7 (major), 4.3 (minor) minutes.

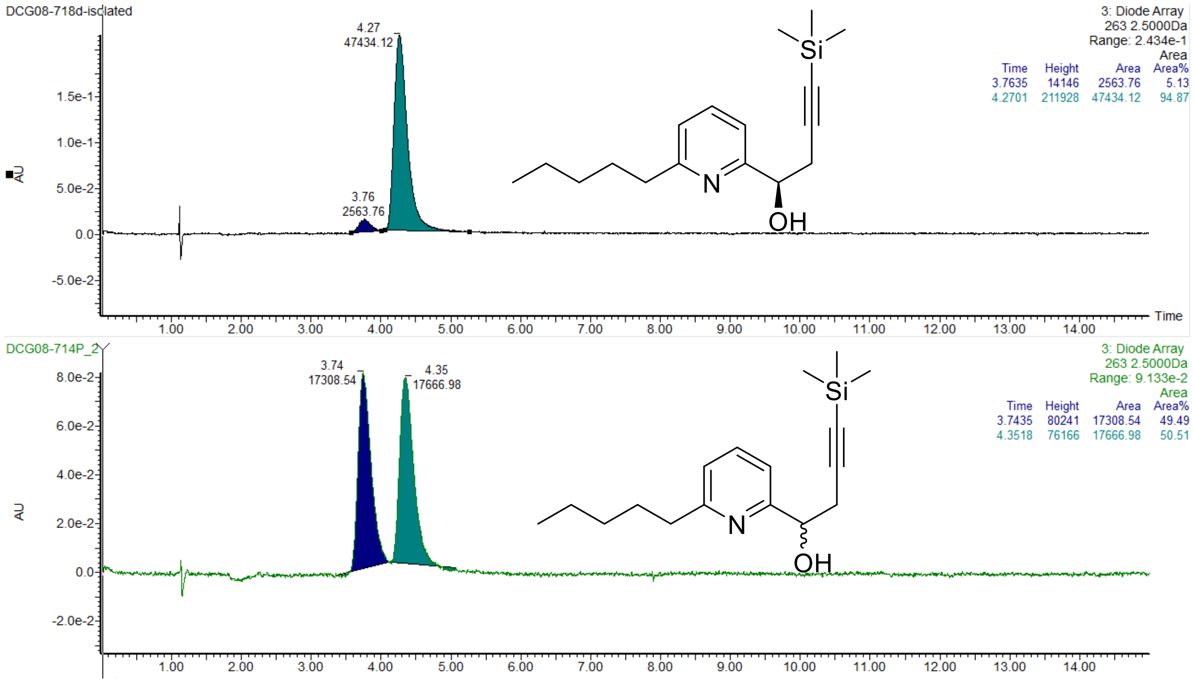

Methyl (*R*)-4-hydroxy-4-(6-pentylpyridin-2-yl)butanoate (6o)

SFC Analysis: CHIRALPAK SC (CO_2/MeOH = 95/5, 2.5 ml min^{-1}, 40 °C, 263 nm); **<u>80% ee</u>**

t_R = 5.2 (minor), 5.9 (major) minutes.

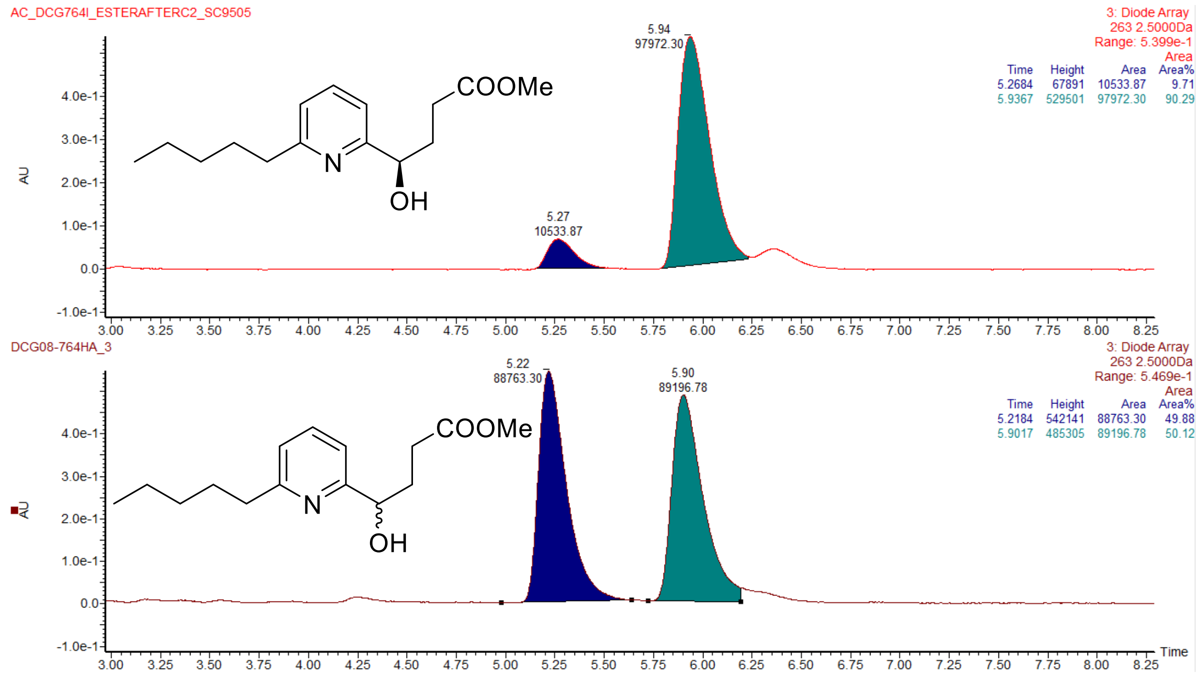

(*R*)-3-((tert-butyldimethylsilyl)oxy)-1-(6-pentylpyridin-2-yl)propan-1-ol (6p)

SFC Analysis: CHIRALPAK SC (CO_2/MeOH = 98/2, 2.5 ml min^{-1}, 40 °C, 263 nm); **<u>74% ee</u>**

t_R = 3.1 (minor), 3.8 (major) minutes.

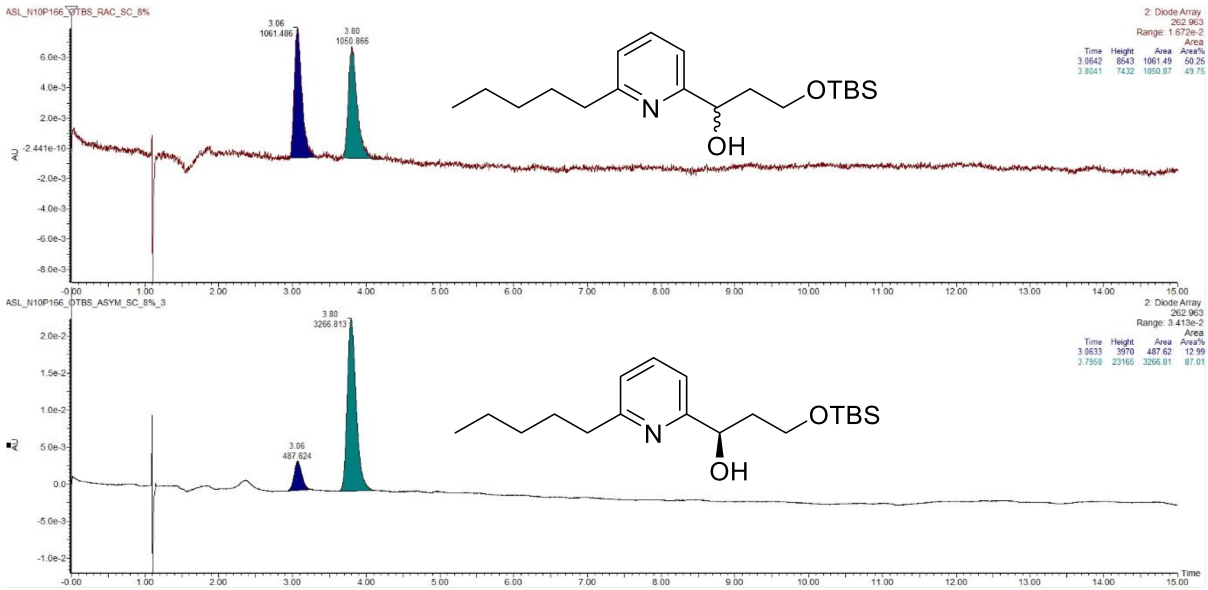

tert-butyl (_R_)-(3-hydroxy-3-(6-pentylpyridin-2-yl)propyl)carbamate (6q)

SFC Analysis: SFC Analysis: CHIRALCEL IG (CO_2/MeOH = 75/25, 2.5 mL min^{-1}, 40 °C); **74% ee** t_R = 7.8 (minor), 8.9 (major) minutes.

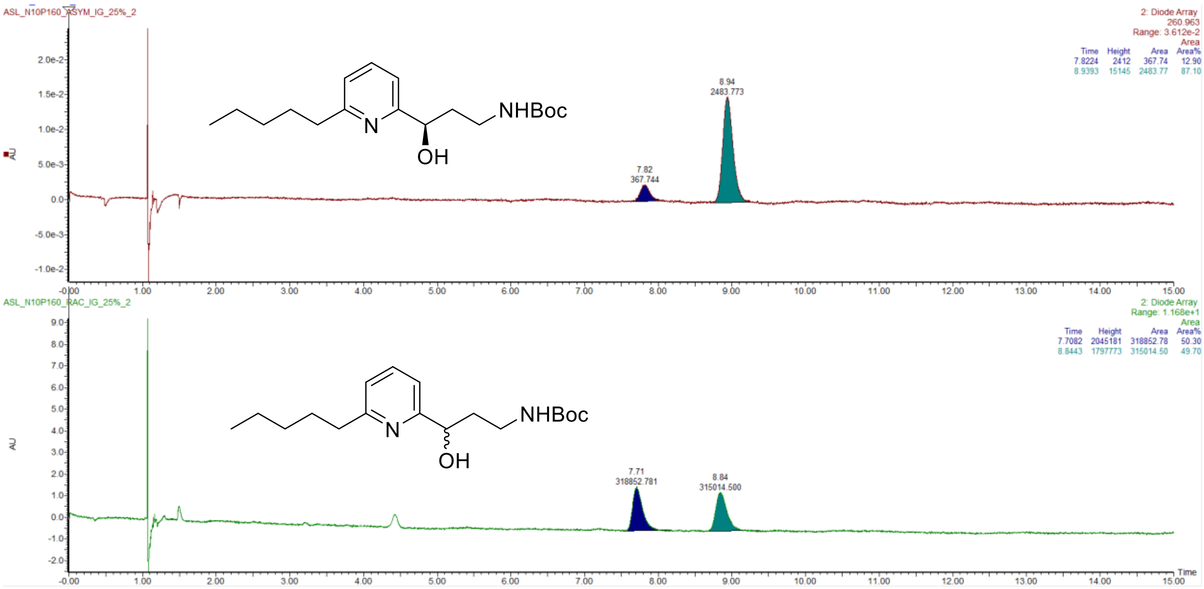

2-(1-methoxy-3-phenylpropyl)-6-pentylpyridine (7a)

SFC Analysis: Chiralpak IE (CO_2/MeOH = 95/05, 2.5 mL min^{-1}, 40 °C, 263 nm); **<u>20% ee</u>**

t_R = 4.3 (major), 4.6 (minor) minutes

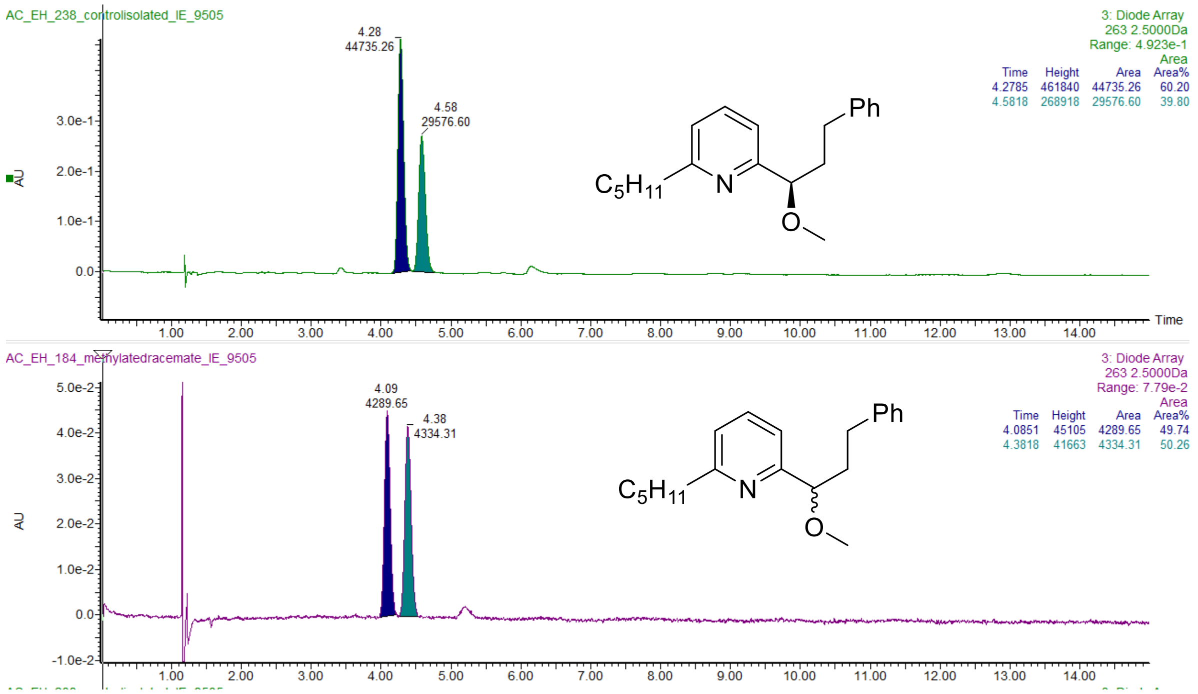

Supplement: Supplementary file 1 — Supporting Information [file ANIE-61-0-s001.pdf]
